# The Burden of Digestive Diseases in the United States Population

**DOI:** 10.1101/2023.08.16.23294166

**Published:** 2023-08-21

**Authors:** Aynur Unalp-Arida, Constance E. Ruhl

**Affiliations:** National Institute of Diabetes and Digestive and Kidney Diseases, National Institutes of Health, Department of Health and Human Services, Two Democracy Plaza, Room 6009, 6707 Democracy Blvd., Bethesda, MD 20892-5458; Social & Scientific Systems, Inc., a DLH Holdings Corp company, 8757 Georgia Avenue, 12^th^ floor, Silver Spring, MD 20910

## Abstract

**Background and rationale.:**

Digestive diseases are common and lead to significant morbidity, mortality, and health care utilization. We used national survey and claims databases to expand on earlier findings and investigate current trends in the digestive disease burden in the United States.

**Methods.:**

The National Ambulatory Medical Care Survey, Nationwide Emergency Department Sample, National Inpatient Sample, Vital Statistics of the U.S., Surveillance, Epidemiology, and End Results Program, Optum Clinformatics^®^ Data Mart, and Centers for Medicare and Medicaid Services Medicare 5% Sample databases were used to estimate medical care, mortality, cancer incidence, and claims-based prevalence with a digestive disease diagnosis. Rates were age-adjusted (for national databases) and shown per 100,000 population.

**Results.:**

For all digestive diseases, prevalence (claims-based, all-listed diagnoses) was 30.5% among commercial insurance enrollees (2020) and 53.1% among Medicare beneficiaries (2019). In the U.S. population, digestive diseases contributed to approximately 126 million ambulatory care visits (2015), 41 million emergency department visits (2018), 16 million hospital discharges (2018), and 472,000 deaths (2019) annually. Prevalence, medical care, and mortality rates with a digestive disease diagnosis were higher among children and younger adults (except for emergency department visits) and then increased with age. Women had higher prevalence and medical care rates with a digestive disease diagnosis, but mortality rates were higher among men. Prevalence and medical care rates with a digestive disease diagnosis were higher among Blacks, followed by Whites, then Hispanics, and lowest among Asians. Mortality rates were higher among Blacks compared with Whites and lower among Hispanics compared with non-Hispanics. Between 2004 and the most recent year, ambulatory care visit rates with a digestive disease diagnosis increased by 4%, hospital discharge rates decreased by 3%, and mortality rates decreased by 7%. Among commercial insurance enrollees, rates were higher compared with national data for ambulatory care visits and hospital discharges, but lower for emergency department visits. The medical care use and mortality burdens varied among individual digestive diseases.

**Conclusion.:**

The digestive disease burden in the United States is substantial, particularly among Blacks and older adults.

## INTRODUCTION

Digestive diseases affect multiple organs and systems including the alimentary tract, liver and biliary system, and pancreas. These disorders have diverse causes such as congenital and genetic anomalies, acute and chronic infections, environmental risk factors, and adverse effects of drugs and toxins. The burden of digestive diseases ranges from the inconvenience of a diarrheal disease to chronic debilitating illnesses requiring continuous medical care to fatal conditions such as pancreatic cancer. There have been significant changes in the prevalence and overall burden of digestive diseases in the 21^st^ century building on earlier public health gains from improved sanitation, food safety, and development of new drugs, vaccines, diagnostic tests, and minimally invasive procedures. The well-being of the population of the United States requires a continued investment in digestive disease research and public health initiatives supported by a health care system capable of providing these advances to all equitably.

As outlined in the National Institute of Diabetes and Digestive and Kidney Diseases (NIDDK) Strategic Plan for Research,(1) a better understanding of social determinants of health, such as access to healthy food and safe places to exercise, may help to reduce health disparities and achieve health equity which may in turn decrease the burden of digestive diseases among racial and ethnic minority and other underserved populations. In this descriptive epidemiology paper, we present essential health data to reduce the burden of digestive diseases and to inform health care providers, researchers, administrators, public officials, professional and patient-based organizations, and the public to act. Our work follows the tradition of National Institutes of Health (NIH) sponsored publications *Digestive diseases in the United States: epidemiology and impact* in 1994,(2) and *Opportunities and challenges in digestive diseases research: recommendations of the National Commission on Digestive Diseases* in 2009,(3) and its companion report on the burden of digestive diseases.(4) Close examination of these reports reveals interesting trends in various digestive diseases despite numerous limitations on the types of data that can be obtained in the diverse and decentralized U.S. health care system. Despite many limitations of available data sources, there are several certainties that may be gleaned, such as a century of progress in health care delivery, scientific advances, and a still staggering burden of digestive diseases in the United States.

## METHODS

Digestive disease morbidity was identified by an International Classification of Diseases (ICD), Tenth Revision, Clinical Modification (ICD-10-CM) codes and digestive disease mortality by ICD, Tenth Revision (ICD-10) codes (Appendix 1). The first-listed diagnosis was considered the primary diagnosis and all remaining diagnoses were considered secondary and included under ‘all-listed’. For national event-level data sources, diagnoses were counted only once under the all-listed category, irrespective of the number of actual diagnoses listed on a medical record or death certificate.

The National Ambulatory Medical Care Survey (NAMCS), Healthcare Cost and Utilization Project (HCUP) Nationwide Emergency Department Sample (NEDS), HCUP National Inpatient Sample (NIS), Vital Statistics of the U.S., Surveillance, Epidemiology, and End Results (SEER) Program, Optum Clinformatics^®^ Data Mart (CDM), and Centers for Medicare and Medicaid Services (CMS) Medicare 5% Sample databases were used to estimate medical care, mortality, years of potential life lost, cancer incidence, and claims-based prevalence with primary or other digestive disease diagnoses. These data sources are described in more detail in Appendix 2. Estimates for the total population and demographic groups were calculated for the most recent year of data available. Rates were age-adjusted (for national databases) and shown per 100,000 population. For national data sources, ambulatory care, emergency department, and hospital numbers and rates represent events, not persons with an event. Statistical methodology is described in more detail in Appendix 3.

There were an estimated 66 million ambulatory care visits to office-based physicians with a first-listed diagnosis of a digestive disease and 126 million visits with a digestive disease diagnosis listed in any position (2015) (Table 1). The ambulatory care visit rate (per 100,000 U.S. population) was 19,962 with a first-listed diagnosis and 37,173 with an all-listed diagnosis. In other words, for every 100 U.S. residents, there were 37 ambulatory care visits at which a digestive disease diagnosis was noted and 20 visits for which a digestive disease diagnosis was primary. All-listed diagnosis rates were higher among children compared with adolescents and younger adults and then increased with age. Age-adjusted rates of all-listed diagnoses were higher among women compared with men, Blacks compared with Whites, and Hispanics compared with non-Hispanics. Between 2004 and 2015, the number of ambulatory care visits with a primary diagnosis of a digestive disease decreased from an estimated 72 million to 66 million and the number with an all-listed diagnosis increased from 105 million to 126 million. Age-adjusted ambulatory care visit rates (per 100,000) with a primary digestive disease diagnosis decreased by 19% overall from 24,543 to 19,962 and decreased among sex and race groups.(4,5) All-listed diagnosis rates increased by 4% overall from 35,684 to 37,173 and increased among women, Whites, and Blacks, but decreased among men.

There were an estimated 19 million emergency department visits with a first-listed diagnosis of a digestive disease and 41 million visits with a digestive disease diagnosis listed in any position in 2018 (Table 2). The emergency department visit rate (per 100,000) was 5,697 with a first-listed diagnosis and 12,064 with an all-listed diagnosis. This equates to 6 emergency department visits with a first-listed digestive disease diagnosis per 100 U.S. residents and 12 visits where a digestive disease was listed in any position. All-listed diagnosis rates increased with age, especially among persons 75 years and over. Age-adjusted rates were higher among women compared with men. National emergency department visit rates were not included in The Burden of Digestive Diseases in the United States compendium so rate trends cannot be examined.(4)

There were an estimated 4.0 million hospital discharges with a first-listed diagnosis of a digestive disease and 16 million discharges with a digestive disease diagnosis listed in any position in 2018 (Table 3). The hospital discharge rate (per 100,000) was 1,098 with a first-listed diagnosis and 4,478 with an all-listed diagnosis. This equates to 1 overnight hospital stay per 100 U.S. residents with a first-listed digestive disease diagnosis and 4 visits that included a digestive disease as a discharge diagnosis in any position. All-listed diagnosis rates were much higher among children compared with adolescents and the youngest adults and then increased with age. Age-adjusted rates were higher among women compared with men, Blacks compared with Whites, and non-Hispanics compared with Hispanics. Between 2004 and 2018, the number of hospital discharges with a primary diagnosis of a digestive disease decreased from an estimated 4.6 million to 4.0 million and the number with an all-listed diagnosis increased from 14 million to 16 million. Age-adjusted hospital discharge rates (per 100,000) with a primary digestive disease diagnosis decreased by 30% overall from 1,563 to 1,098 and decreased among sex and race groups.(4,5) All-listed diagnosis rates decreased by only 3% overall from 4,608 to 4,478 and decreased among women, but increased among men, Whites, and Blacks.

A digestive disease was the underlying cause of 294,000 deaths and an underlying or contributing cause of 472,000 deaths in 2019 (Table 4). Mortality rates (per 100,000) were 72.4 as underlying cause and 116.3 as underlying or other cause. Mortality rates (underlying or other cause) were higher among children compared with adolescents and the youngest adults and then increased with age. In contrast to health care rates, age-adjusted mortality rates were higher among men compared with women. They were higher among Blacks compared with Whites and among non-Hispanics compared with Hispanics. Between 2004 and 2019, the number of deaths with a digestive disease as underlying cause increased from 236,000 to 294,000 and the number with a digestive disease as underlying or other cause increased from 366,000 to 472,000. Age-adjusted mortality rates (per 100,000) with a digestive disease as underlying cause decreased by 10% overall from 80.4 to 72.4 and decreased among sex and race groups.(4,5) Rates with a digestive disease as underlying or other cause decreased by 7% overall from 124.8 to 116.3 and decreased among sex and race groups.

In contrast to national data that represent events, Optum data are at the person level which enables calculation of claims-based prevalence. Among 13.7 million private insurance enrollees (2020), 2.2 million had a first-listed digestive disease diagnosis (claims-based prevalence of 16.1%) and 4.2 million had an all-listed diagnosis (claims-based prevalence of 30.5%) (Table 5). Prevalence (all-listed diagnoses) was higher among children compared with adolescents and the youngest adults and then increased with age until the oldest age group. Prevalence was higher among women compared with men. It was highest among Blacks, followed by Whites, then Hispanics, and lowest among Asians. Prevalence was higher in the Northeast and South compared with the Midwest and West.

Among commercial insurance enrollees, ambulatory care visit rates with both first-listed and all-listed digestive disease diagnoses were much higher compared with national data; however, availability of more recent data for commercial insurance enrollees (2020 vs. 2015) may limit comparability (Table 6). Like national data, ambulatory care visit rates (all-listed diagnoses) were higher among children compared with adolescents and the youngest adults and then increased with age and were higher among women compared with men. Optum data include information on ambulatory care visits among Asians that are not available in national data used in this paper. Among persons with known race-ethnicity, rates were highest among Blacks, followed by Whites and Hispanics, and lowest among Asians. Ambulatory care visit rates were higher in the South and Northeast compared with the Midwest and West.

Among commercial insurance enrollees, emergency department visit rates with both first-listed and all-listed digestive disease diagnoses were much lower compared with national data; however, availability of more recent data for commercial insurance enrollees (2020 vs. 2018) may limit comparability (Table 7). Like national data, emergency department visit rates (all-listed diagnoses) increased with age and were higher among women compared with men. Optum data include information on emergency department visits by race-ethnicity that are not available in national data used in this paper. Among persons with known race-ethnicity, rates were highest among Blacks, followed by Whites and Hispanics, and lowest among Asians. Emergency department visit rates were higher in the South and Northeast compared with the Midwest and West.

Among commercial insurance enrollees, hospital discharge rates with both first-listed and all-listed digestive disease diagnoses were higher compared with national data; however, availability of more recent data for commercial insurance enrollees (2020 vs. 2018) may limit comparability (Table 8). Like national data, hospital discharge rates (all-listed diagnoses) were higher among children compared with adolescents and the youngest adults and then increased with age and were higher among women compared with men. Optum data include information on hospital discharges among Asians that are not available in national data used in this paper. Among persons with known race-ethnicity, rates were highest among Blacks, followed by Whites, then Hispanics, and lowest among Asians. Hospital discharge rates were highest in the Northeast and South, intermediate in the Midwest, and lowest in the West.

Like Optum data, Medicare data are at the person level and enables calculation of claims-based prevalence. Among 27 million age-eligible Medicare beneficiaries (2019), 7.8 million had a first-listed digestive disease diagnosis (claims-based prevalence of 29.1%) and 14 million had an all-listed diagnosis (claims-based prevalence of 53.1%) (Table 9). Prevalence (all-listed diagnoses) increased with age and was higher among women compared with men and Whites compared with Blacks. Prevalence was highest in the South, followed by the Northeast, then the Midwest, and lowest in the West. Medicare claims data were not utilized in The Burden of Digestive Diseases in the United States compendium so rate trends cannot be examined.(4)

Among age-eligible Medicare beneficiaries, there were an estimated 19 million ambulatory care visits with a first-listed diagnosis of a digestive disease and 43 million visits with a digestive disease diagnosis listed in any position (2019) (Table 10). The ambulatory care visit rate (per 100,000 population) was 69,119 with a first-listed diagnosis and 161,206 with an all-listed diagnosis. All-listed diagnosis rates increased with age until 85 years and were higher among women compared with men and Blacks compared with Whites. Rates were higher in the South and Northeast compared with the West and Midwest.

Among age-eligible Medicare beneficiaries, there were an estimated 2.4 million emergency department visits with a first-listed diagnosis of a digestive disease and 7.1 million visits with a digestive disease diagnosis listed in any position (2019) (Table 11). The emergency department visit rate (per 100,000 population) was 9,135 with a first-listed diagnosis and 26,607 with an all-listed diagnosis. All-listed diagnosis rates increased with age and were higher among women compared with men and Blacks compared with Whites. Rates were highest in the South, followed by the Northeast and Midwest, and lowest in the West.

Among age-eligible Medicare beneficiaries, there were an estimated 915,000 hospital discharges with a first-listed diagnosis of a digestive disease and 17,000 discharges with a digestive disease diagnosis listed in any position (2019) (Table 12). The hospital discharge rate (per 100,000 population) was 3,413 with a first-listed diagnosis and 17,407 with an all-listed diagnosis. All-listed diagnosis rates increased with age and were higher among Blacks compared with Whites but differed little by sex. Rates were lower in the West compared with the South, Midwest, and Northeast.

Gastroesophageal reflux disease contributed to 26.7 million ambulatory visits (2015) (Table 13). Ambulatory care visit rates (all-listed diagnoses) were higher among children compared with adolescents and the youngest adults and then increased with age. Age-adjusted rates were higher among women compared with men, Blacks compared with Whites, and Hispanics compared with non-Hispanics.

Gastroesophageal reflux disease contributed to 9.5 million emergency department visits in 2018 (Table 14). Emergency department visit rates (all-listed diagnoses) increased with age. Age-adjusted rates were higher among women compared with men.

Gastroesophageal reflux disease contributed to 6.2 million hospital discharges in 2018 (Table 15). Hospital discharge rates (all-listed diagnoses) increased with age. Age-adjusted rates were higher among women compared with men, Blacks compared with Whites, and non-Hispanics compared with Hispanics. Between 2004 and 2018, age-adjusted hospital discharge rates (per 100,000) with an all-listed diagnosis increased by 50% from 1,086 to 1628.(4,5)

Gastroesophageal reflux disease contributed to 12,000 deaths in 2019 (Table 16). Mortality rates (underlying or other cause) were higher among children compared with adolescents and the youngest adults and then increased with age. Age-adjusted mortality rates were higher among men, Whites, and non-Hispanics. Between 2004 and 2019, age-adjusted mortality rates (per 100,000) with gastroesophageal reflux disease as underlying or other cause increased by 7% from 2.7 to 2.9.(4)

Among privately insured enrollees, the claims-based prevalence of gastroesophageal reflux disease (based on all-listed diagnoses) was 11.1% (Table 17). Prevalence was similar among children compared with adolescents and the youngest adults and then increased with age. It was higher among women. It was highest among Blacks, followed by Whites and Hispanics, and lowest among Asians. It was highest in the South, followed by the Northeast, then the Midwest, and lowest in the West.

Among commercial insurance enrollees, ambulatory care visit rates with gastroesophageal reflux disease (all-listed diagnoses) were higher among children compared with adolescents and the youngest adults and then increased with age and were higher among women compared with men (Table 18). Among persons with known race-ethnicity, rates were highest among Blacks, followed by Whites, then Hispanics, and lowest among Asians. Rates were highest in the South, followed by the Northeast, then the Midwest, and lowest in the West.

Among commercial insurance enrollees, emergency department visit rates with gastroesophageal reflux disease (all-listed diagnoses) increased with age and were higher among women compared with men (Table 19). Among persons with known race-ethnicity, rates were highest among Blacks, followed by Whites, then Hispanics, and lowest among Asians. Rates were highest in the Northeast, followed by the Midwest and South, and lowest in the West.

Among commercial insurance enrollees, hospital discharge rates with gastroesophageal reflux disease (all-listed diagnoses) increased with age and were higher among women compared with men (Table 20). Among persons with known race-ethnicity, rates were highest among Blacks, followed by Whites, then Hispanics, and lowest among Asians. Rates were highest in the South, followed by the Northeast, then the Midwest, and lowest in the West.

Among Medicare beneficiaries, the claims-based prevalence of gastroesophageal reflux disease (based on all-listed diagnoses) was 23.9% (Table 21). Prevalence increased with age until the oldest age group and was higher among women and Whites. It was highest in the South, followed by the Midwest and Northeast, and lowest in the West.

Among Medicare beneficiaries, ambulatory care visit rates with gastroesophageal reflux disease (all-listed diagnoses) increased with age and were higher among women compared with men and Blacks compared with Whites (Table 22). Rates were highest in the South, followed by the Northeast, then the Midwest, and lowest in the West.

Among Medicare beneficiaries, emergency department visit rates with gastroesophageal reflux disease (all-listed diagnoses) increased with age and were higher among women compared with men and Blacks compared with Whites (Table 23). Rates were lower in the West compared with the South, Midwest, and Northeast.

Among Medicare beneficiaries, hospital discharge rates with gastroesophageal reflux disease (all-listed diagnoses) increased with age and were higher among women compared with men and Blacks compared with Whites (Table 24). Rates were lower in the West compared with the South, Midwest, and Northeast.

Peptic ulcer disease contributed to 1.4 million ambulatory visits (2015) (Table 25). Ambulatory care visits were uncommon during childhood and then rates (all-listed diagnoses) increased with age until the oldest age group. Age-adjusted ambulatory care visit rates were higher among women compared with men, Blacks compared with Whites, and Hispanics compared with non-Hispanics.

Peptic ulcer disease contributed to 471,000 emergency department visits in 2018 (Table 26). Emergency department visit rates increased with age. Age-adjusted emergency department visit rates were higher among men compared with women.

Peptic ulcer disease contributed to 439,000 hospital discharges in 2018 (Table 27). Hospital discharge rates increased with age. Hospital discharge rates were higher among men compared with women, Blacks compared with Whites, and non-Hispanics compared with Hispanics. Between 2004 and 2018, age-adjusted hospital discharge rates (per 100,000) with an all-listed diagnosis decreased by 31% from 166 to 114.(4)

Peptic ulcer disease contributed to 7,000 deaths in 2019 (Table 28). Mortality was uncommon among the youngest age groups and then rates (underlying or other cause) increased with age. Age-adjusted mortality rates were higher among men, Whites, and non-Hispanics. Between 2004 and 2019, age-adjusted mortality rates (per 100,000) with peptic ulcer disease as underlying or other cause decreased by 39% from 2.8 to 1.7.(4)

Among privately insured enrollees, the claims-based prevalence of peptic ulcer disease (based on all-listed diagnoses) was 0.5% (Table 29). Prevalence increased with age and was higher among women. It was highest among Blacks, similar among Whites and Hispanics, and lowest among Asians. It was highest in the Northeast and South, and lowest in the West.

Among commercial insurance enrollees, ambulatory care visit rates with peptic ulcer disease (all-listed diagnoses) increased with age and were higher among women compared with men (Table 30). Among persons with known race-ethnicity, rates were highest among Blacks, followed by Whites, then Hispanics, and lowest among Asians. Rates were highest in the South, followed by the Northeast, then the West, and lowest in the Midwest.

Among commercial insurance enrollees, emergency department visit rates with peptic ulcer disease (all-listed diagnoses) increased with age and differed little by sex (Table 31). Among persons with known race-ethnicity, rates were highest among Blacks, followed by Whites, then Hispanics, and lowest among Asians. Rates were highest in the Northeast, followed by the South, and lowest in the Midwest and West.

Among commercial insurance enrollees, hospital discharge rates with peptic ulcer disease (all-listed diagnoses) increased with age and were higher among men compared with women (Table 32). Among persons with known race-ethnicity, rates were highest among Blacks, followed by Whites, then Hispanics, and lowest among Asians. Rates were higher in the Northeast and South compared with the Midwest and West.

Among Medicare beneficiaries, the claims-based prevalence of peptic ulcer disease (based on all-listed diagnoses) was 1.2% (Table 33). Prevalence increased with age until the oldest age group and was higher among women and Blacks. It differed little by region.

Among Medicare beneficiaries, ambulatory care visit rates with peptic ulcer disease (all-listed diagnoses) increased with age until 85 years and were higher among women compared with men but differed little by race (Table 34). Rates were highest in the West and South, followed by the Northeast, and lowest in the Midwest.

Among Medicare beneficiaries, emergency department visit rates with peptic ulcer disease (all-listed diagnoses) increased with age and were higher among men compared with women and Blacks compared with Whites (Table 35). Rates were higher in the South compared with the Northeast and Midwest and lowest in the West.

Among Medicare beneficiaries, hospital discharge rates with peptic ulcer disease (all-listed diagnoses) increased with age and were higher among men compared with women and Blacks compared with Whites (Table 36). Rates were highest in the South, followed by the Midwest, then the West, and lowest in the Northeast.

Functional disorders contributed to 12.2 million ambulatory visits (2015) (Table 37). Ambulatory care visit rates (all-listed diagnoses) were higher among children compared with adolescents and younger adults and then generally increased with age. Age-adjusted rates were higher among women compared with men, Blacks compared with Whites, and Hispanics compared with non-Hispanics.

Functional disorders contributed to 4.5 million emergency department visits in 2018 (Table 38). Emergency department visit rates (all-listed diagnoses) were higher among children compared with adolescents and younger adults and then generally increased with age. Age-adjusted rates were higher among women compared with men.

Functional disorders contributed to 2.6 million hospital discharges in 2018 (Table 39). Hospital discharge rates (all-listed diagnoses) increased with age. Age-adjusted rates were higher among women compared with men, Blacks compared with Whites, and non-Hispanics compared with Hispanics. Between 2004 and 2018, age-adjusted hospital discharge rates (per 100,000) with an all-listed diagnosis increased by 64% from 423 to 695.(4)

Functional disorders contributed to 4,000 deaths in 2019 (Table 40). Mortality rates (underlying or other cause) increased with age. Age-adjusted mortality rates were higher among women, Whites, and non-Hispanics. Between 2004 and 2019, age-adjusted mortality rates (per 100,000) with functional disorders as underlying or other cause increased by 57% from 0.7 to 1.1.(4)

Among privately insured enrollees, the claims-based prevalence of functional disorders (based on all-listed diagnoses) was 5.1% (Table 41). Prevalence was higher among children compared with adolescents and younger adults and then increased with age. It was higher among women. It was highest among Blacks, followed by Whites and Hispanics, and lowest among Asians. It was higher in the Northeast and South compared with the Midwest and West.

Among commercial insurance enrollees, ambulatory care visit rates with functional disorders (all-listed diagnoses) were higher among children compared with adolescents and younger adults and then increased with age and were higher among women compared with men (Table 42). Among persons with known race-ethnicity, rates were highest among Blacks, followed by Whites and Hispanics, and lowest among Asians. Rates were highest in the Northeast, followed by the South, and lowest in the West and Midwest.

Among commercial insurance enrollees, emergency department visit rates with functional disorders (all-listed diagnoses) increased with age and were higher among women compared with men (Table 43). Among persons with known race-ethnicity, rates were highest among Blacks, followed by Whites, then Hispanics, and lowest among Asians. Rates were highest in the South, followed by the Northeast, then the West, and lowest in the Midwest.

Among commercial insurance enrollees, hospital discharge rates with functional disorders (all-listed diagnoses) increased with age and were higher among women compared with men (Table 44). Among persons with known race-ethnicity, rates were highest among Blacks, followed by Whites, then Hispanics, and lowest among Asians. Rates were highest in the Northeast, followed by the South, then the Midwest, and lowest in the West.

Among Medicare beneficiaries, the claims-based prevalence of functional disorders (based on all-listed diagnoses) was 11.3% (Table 45). Prevalence increased with age and was higher among women and Blacks. It was highest in the South, followed by the Northeast, then the Midwest, and lowest in the West.

Among Medicare beneficiaries, ambulatory care visit rates with functional disorders (all-listed diagnoses) increased with age and were higher among women compared with men and Blacks compared with Whites (Table 46). Rates were highest in the Northeast, followed by the South, then the Midwest, and lowest in the West.

Among Medicare beneficiaries, emergency department visit rates with functional disorders (all-listed diagnoses) increased with age and were higher among women compared with men and Blacks compared with Whites (Table 47). Rates were highest in the Northeast and South, followed by the Midwest, and lowest in the West.

Among Medicare beneficiaries, hospital discharge rates with functional disorders (all-listed diagnoses) increased with age and were higher among women compared with men and Blacks compared with Whites (Table 48). Rates were lower in the West compared with the South, Northeast, and Midwest.

Appendicitis contributed to 739,000 ambulatory visits (2015) (Table 49). Ambulatory care visit rates (all-listed diagnoses) were highest among persons 55–64 years and lowest among those 75 years and over. Age-adjusted ambulatory care visit rates were higher among men compared with women, Whites compared with Blacks, and Hispanics compared with non-Hispanics.

Appendicitis contributed to 421,000 emergency department visits in 2018 (Table 50). Emergency department visit rates (all-listed diagnoses) peaked among persons 12–24 years and then decreased with age. Age-adjusted rates were higher among men compared with women.

Appendicitis contributed to 193,000 hospital discharges in 2018 (Table 51). Hospital discharge rates (all-listed diagnoses) peaked at 12–24 years and were similar for other age groups. Age-adjusted rates were higher among men compared with women, Whites compared with Blacks, and Hispanics compared with non-Hispanics. Between 2004 and 2018, age-adjusted hospital discharge rates (per 100,000) with an all-listed diagnosis decreased by 47% from 111 to 59. (4,6)

Appendicitis contributed to 1,000 deaths in 2019 (Table 52). Mortality was uncommon through young adulthood after which rates (underlying or other cause) increased with age. Age-adjusted mortality rates did not differ by sex, race, or ethnicity. Between 2004 and 2019, age-adjusted mortality rates (per 100,000) with appendicitis as underlying or other cause decreased by a third from 0.3 to 0.2.(4)

Among privately insured enrollees, the claims-based prevalence of appendicitis (based on all-listed diagnoses) was 0.1% (Table 53). Prevalence was highest among adolescents and younger adults and did not differ by sex. It was highest among Hispanics and similar among Whites, Blacks, and Asians. It did not differ by region.

Among commercial insurance enrollees, ambulatory care visit rates with appendicitis (all-listed diagnoses) peaked among adolescents and the youngest adults and then decreased with age and were higher among men compared with women (Table 54). Among persons with known race-ethnicity, rates were highest among Hispanics, followed by Whites, then Asians, and lowest among Blacks. Rates were highest in the Midwest, followed by the South and Northeast, and lowest in the West.

Among commercial insurance enrollees, emergency department visit rates with appendicitis (all-listed diagnoses) increased with age until 75 years and were higher among women compared with men (Table 55). Among persons with known race-ethnicity, rates were highest among Whites and Hispanics, followed by Blacks, and lowest among Asians. Rates were higher in the Midwest and West compared with the Northeast and South.

Among commercial insurance enrollees, hospital discharge rates with appendicitis (all-listed diagnoses) generally increased with age and were higher among men compared with women (Table 56). Among persons with known race-ethnicity, rates were highest among Hispanics, followed by Whites and Blacks, and lowest among Asians. Rates were highest in the Northeast, followed by the South and Midwest, and lowest in the West.

Among Medicare beneficiaries, the claims-based prevalence of appendicitis (based on all-listed diagnoses) was 0.1% (Table 57). Prevalence did not differ by age, sex, or race. It did not differ by region.

Among Medicare beneficiaries, ambulatory care visit rates with appendicitis (all-listed diagnoses) decreased with age, differed little by sex, and were higher among Whites compared with Blacks (Table 58). Rates were highest in the West, followed by the South, then the Midwest, and lowest in the Northeast.

Among Medicare beneficiaries, emergency department visit rates with appendicitis (all-listed diagnoses) decreased with age until 85 years and were higher among men compared with women and Whites compared with Blacks (Table 59). Rates were highest in the West, followed by the South and Northeast, and lowest in the Midwest.

Among Medicare beneficiaries, hospital discharge rates with appendicitis (all-listed diagnoses) peaked among persons 70 to 74 years and were higher among men compared with women and Whites compared with Blacks (Table 60). Rates were highest in the West, followed by the South, then the Northeast, and lowest in the Midwest.

Abdominal wall hernia contributed to 5.1 million ambulatory visits (2015) (Table 61). Ambulatory care visit rates (all-listed diagnoses) were higher among children compared with adolescents and the youngest adults and then increased with age. Age-adjusted rates were higher among men compared with women, Blacks compared with Whites, and Hispanics compared with non-Hispanics.

Abdominal wall hernia contributed to 761,000 emergency department visits in 2018 (Table 62). Emergency department visit rates (all-listed diagnoses) were higher among children compared with adolescents and the youngest adults and then increased with age. Age-adjusted rates were higher among men compared with women.

Abdominal wall hernia contributed to 452,000 hospital discharges in 2018 (Table 63). Hospital discharge rates (all-listed diagnoses) were higher among children compared with adolescents and the youngest adults and then increased with age. Age-adjusted rates were higher among men compared with women and Blacks compared with Whites but did not differ by ethnicity. Between 2004 and 2018, age-adjusted hospital discharge rates (per 100,000) with an all-listed diagnosis decreased by 5% from 127 to 121.(4,6)

Abdominal wall hernia contributed to 2,000 deaths in 2019 (Table 64). Mortality was uncommon among the youngest age groups after which rates (underlying or other cause) increased with age. Age-adjusted mortality rates were higher among men, Blacks, and non-Hispanics. Between 2004 and 2019, age-adjusted mortality rates (per 100,000) with abdominal hernia as underlying or other cause decreased by 14% from 0.7 to 0.6.(4)

Among privately insured enrollees, the claims-based prevalence of abdominal wall hernia (based on all-listed diagnoses) was 1.1% (Table 65). Prevalence was higher among children compared with adolescents and younger adults and then increased with age. It was higher among men. It was similar among Whites, Blacks, and Hispanics, and lowest among Asians. It differed little by region.

Among commercial insurance enrollees, ambulatory care visit rates with abdominal wall hernia (all-listed diagnoses) were higher among children compared with adolescents and younger adults and then increased with age until 75 years (Table 66). They were higher among men compared with women. Among persons with known race-ethnicity, rates were highest among Whites, followed by Blacks, then Hispanics, and lowest among Asians. Rates were highest in the Northeast, followed by the South, then the West, and lowest in the Midwest.

Among commercial insurance enrollees, emergency department visit rates with abdominal wall hernia (all-listed diagnoses) increased with age and were higher among men compared with women (Table 67). Among persons with known race-ethnicity, rates were highest among Blacks, followed by Hispanics and Whites, and much lower among Asians. Rates were highest in the Northeast, followed by the South, then the West, and lowest in the Midwest.

Among commercial insurance enrollees, hospital discharge rates with abdominal wall hernia (all-listed diagnoses) were higher among children compared with adolescents and younger adults and then increased with age and were higher among men compared with women (Table 68). Among persons with known race-ethnicity, rates were highest among Blacks, followed by Hispanics and Whites, and much lower among Asians. Rates were highest in the Northeast, followed by the South, and lowest in the Midwest and West.

Among Medicare beneficiaries, the claims-based prevalence of abdominal wall hernia (based on all-listed diagnoses) was 2.3% (Table 69). Prevalence was highest among persons 75–84 years and was higher among men and Whites. It was higher in the Northeast compared to other regions.

Among Medicare beneficiaries, ambulatory care visit rates with abdominal wall hernia (all-listed diagnoses) peaked among persons 75 to 79 years and were over twice as high among men compared with women and Whites compared with Blacks (Table 70). Rates were highest in the Northeast, followed by the West, then the South, and lowest in the Midwest.

Among Medicare beneficiaries, emergency department visit rates with abdominal wall hernia (all-listed diagnoses) increased with age and were higher among men compared with women and Blacks compared with Whites (Table 71). Rates were highest in the Northeast, followed by the South, and lowest in the Midwest and West.

Among Medicare beneficiaries, hospital discharge rates with abdominal wall hernia (all-listed diagnoses) increased with age and were higher among men compared with women and Blacks compared with Whites (Table 72). Rates were highest in the Northeast, followed by the South, then the Midwest, and lowest in the West.

Crohn’s disease contributed to 1.0 million ambulatory visits (2015) (Table 73). Ambulatory care visit rates (all-listed diagnoses) were highest among persons 65–74 years. Age-adjusted rates were higher among women compared with men, Whites compared with Blacks, and non-Hispanics compared with Hispanics.

Crohn’s disease contributed to 375,000 emergency department visits in 2018 (Table 74). Emergency department visit rates (all-listed diagnoses) were highest among persons 25–44 years. Age-adjusted rates were higher among women compared with men.

Crohn’s disease contributed to 207,000 hospital discharges in 2018 (Table 75). Hospital discharge rates (all-listed diagnoses) increased with age. Age-adjusted rates were higher among women compared with men, Whites compared with Blacks, and non-Hispanics compared with Hispanics. Between 2004 and 2018, age-adjusted hospital discharge rates (per 100,000) with an all-listed diagnosis increased by 23% from 48 to 59.(4)

Crohn’s disease contributed to 2,000 deaths in 2019 (Table 76). Mortality was uncommon among the youngest age groups after which rates (underlying or other cause) increased with age. Age-adjusted mortality rates were higher among men, Whites, and non-Hispanics. Between 2004 and 2019, age-adjusted mortality rates (per 100,000) with Crohn’s disease as underlying or other cause increased by 20% from 0.5 to 0.6.(4)

Among privately insured enrollees, the claims-based prevalence of Crohn’s disease (based on all-listed diagnoses) was 0.3% (Table 77). Prevalence was highest in middle age and did not differ by sex. It was highest among Whites and Blacks, followed by Hispanics, and lowest among Asians. It differed little by region.

Among commercial insurance enrollees, ambulatory care visit rates with Crohn’s disease (all-listed diagnoses) peaked among persons 55 to 64 years and were higher among women compared with men (Table 78). Among persons with known race-ethnicity, rates were highest among Whites, followed by Blacks, then Hispanics, and lowest among Asians. Rates were highest in the Northeast, followed by the South, then the Midwest, and lowest in the West.

Among commercial insurance enrollees, emergency department visit rates with Crohn’s disease (all-listed diagnoses) peaked among persons 45 to 54 years and were higher among women compared with men (Table 79). Among persons with known race-ethnicity, rates were highest among Whites and Blacks, followed by Hispanics, and lowest among Asians. Rates were highest in the Northeast, followed by the South and Midwest, and lowest in the West.

Among commercial insurance enrollees, hospital discharge rates with Crohn’s disease (all-listed diagnoses) increased with age until 65 years and were higher among women compared with men (Table 80). Among persons with known race-ethnicity, rates were highest among Blacks and Whites, followed by Hispanics, and lowest among Asians. Rates were highest in the Northeast, followed by the South, then the Midwest, and lowest in the West.

Among Medicare beneficiaries, the claims-based prevalence of Crohn’s disease (based on all-listed diagnoses) was 0.4% (Table 81). Prevalence differed little by age, did not differ by sex, and was higher among Whites. It was highest in the Northeast and lowest in the West.

Among Medicare beneficiaries, ambulatory care visit rates with Crohn’s disease (all-listed diagnoses) decreased with age and were higher among women compared with men and Whites compared with Blacks (Table 82). Rates were highest in the Northeast, followed by the South and Midwest, and lowest in the West.

Among Medicare beneficiaries, emergency department visit rates with Crohn’s disease (all-listed diagnoses) increased with age until 85 years and were higher among women compared with men and Whites compared with Blacks (Table 83). Rates were highest in the Northeast, followed by the Midwest, then the South, and lowest in the West.

Among Medicare beneficiaries, hospital discharge rates with Crohn’s disease (all-listed diagnoses) peaked among persons 75 to 79 years and were higher among women compared with men and Whites compared with Blacks (Table 84). Rates were highest in the Northeast, followed by the Midwest, then the South, and lowest in the West.

Ulcerative colitis contributed to 1.3 million ambulatory visits (2015) (Table 85). Ambulatory care visit rates (all-listed diagnoses) were highest among persons 65–74 years. Age-adjusted rates were higher among women compared with men, Blacks compared with Whites, and Hispanics compared with non-Hispanics.

Ulcerative colitis contributed to 164,000 emergency department visits in 2018 (Table 86). Emergency department visit rates (all-listed diagnoses) increased with age. Age-adjusted rates were higher among women compared with men.

Ulcerative colitis contributed to 128,000 hospital discharges in 2018 (Table 87). Hospital discharge rates (all-listed diagnoses) increased with age. Age-adjusted rates were higher among women compared with men, Whites compared with Blacks, and non-Hispanics compared with Hispanics. Between 2004 and 2018, age-adjusted hospital discharge rates (per 100,000) with an all-listed diagnosis increased by 29% from 28 to 36.(4)

Ulcerative colitis contributed to 1,000 deaths in 2019 (Table 88). Mortality was uncommon among the youngest age groups after which rates (underlying or other cause) increased with age. Age-adjusted mortality rates were higher among men, Whites, and non-Hispanics. Between 2004 and 2019, age-adjusted mortality rates (per 100,000) with ulcerative colitis as underlying or other cause increased by a third from 0.3 to 0.4.(4)

Among privately insured enrollees, the claims-based prevalence of ulcerative colitis (based on all-listed diagnoses) was 0.4% (Table 89). Prevalence increased with age until the oldest age group and was higher among women. It was highest among Whites, similar among Blacks and Hispanics, and lowest among Asians. It differed little by region.

Among commercial insurance enrollees, ambulatory care visit rates with ulcerative colitis (all-listed diagnoses) peaked among persons 55 to 74 years and were higher among women compared with men (Table 90). Among persons with known race-ethnicity, rates were highest among Whites, followed by Blacks, then Hispanics, and lowest among Asians. Rates were highest in the Northeast, followed by the South, then the Midwest, and lowest in the West.

Among commercial insurance enrollees, emergency department visit rates with ulcerative colitis (all-listed diagnoses) were highest among persons 75 years and older and were higher among women compared with men (Table 91). Among persons with known race-ethnicity, rates were highest among Whites, followed by Blacks, then Hispanics, and lowest among Asians. Rates were highest in the Northeast, followed by the South, and lowest in the Midwest and West.

Among commercial insurance enrollees, hospital discharge rates with ulcerative colitis (all-listed diagnoses) increased with age and were higher among women compared with men (Table 92). Among persons with known race-ethnicity, rates were highest among Whites, followed by Blacks, then Hispanics, and lowest among Asians. Rates were highest in the Northeast, followed by the South, then the Midwest, and lowest in the West.

Among Medicare beneficiaries, the claims-based prevalence of ulcerative colitis (based on all-listed diagnoses) was 0.6% (Table 93). Prevalence was highest among persons 65–79 years, did not differ by sex, and was higher among Whites. It was highest in the Northeast and lowest in the South and West.

Among Medicare beneficiaries, ambulatory care visit rates with ulcerative colitis (all-listed diagnoses) peaked among persons 70 to 74 years and were higher among men compared with women and Whites compared with Blacks (Table 94). Rates were highest in the Northeast, followed by the South, then the Midwest, and lowest in the West.

Among Medicare beneficiaries, emergency department visit rates with ulcerative colitis (all-listed diagnoses) increased with age and were higher among women compared with men and Whites compared with Blacks (Table 95). Rates were highest in the Northeast, followed by the Midwest, then the South, and lowest in the West.

Among Medicare beneficiaries, hospital discharge rates with ulcerative colitis (all-listed diagnoses) increased with age until 85 years and were higher among women compared with men and Whites compared with Blacks (Table 96). Rates were highest in the Northeast, followed by the Midwest, then the South, and lowest in the West.

Diverticular disease contributed to 3.6 million ambulatory visits (2015) (Table 97). Ambulatory care visits were uncommon among children and then rates (all-listed diagnoses) increased with age. Age-adjusted rates were higher among women compared with men, Whites compared with Blacks, and Hispanics compared with non-Hispanics.

Diverticular disease contributed to 1.5 million emergency department visits in 2018 (Table 98). Emergency department visits were uncommon among children and then rates (all-listed diagnoses) increased with age. Age-adjusted rates were higher among women compared with men.

Diverticular disease contributed to 880,000 hospital discharges in 2018 (Table 99). Hospitalizations were uncommon among children and then hospital discharge rates (all-listed diagnoses) increased with age. Age-adjusted rates were higher among women compared with men, Blacks compared with Whites, and non-Hispanics compared with Hispanics. Between 2004 and 2018, age-adjusted hospital discharge rates (per 100,000) with an all-listed diagnosis decreased by 18% from 278 to 227.(4,6)

Diverticular disease contributed to 5,000 deaths in 2019 (Table 100). Mortality was uncommon among the youngest age groups after which rates (underlying or other cause) increased with age. Age-adjusted mortality rates were higher among women, Whites, and non-Hispanics. Between 2004 and 2019, age-adjusted mortality rates (per 100,000) with diverticular disease as underlying or other cause decreased by 40% from 2.0 to 1.2.(4)

Among privately insured enrollees, the claims-based prevalence of diverticular disease (based on all-listed diagnoses) was 2.9% (Table 101). Diverticular disease was uncommon in childhood and adolescence and then prevalence increased with age until the oldest age group. Prevalence was higher among women. It was highest among Blacks, followed by Whites and Hispanics, and lowest among Asians. It was highest in the Northeast, followed by the South and Midwest, and lowest in the West.

Among commercial insurance enrollees, ambulatory care visit rates with diverticular disease (all-listed diagnoses) increased with age until 75 years and were higher among women compared with men (Table 102). Among persons with known race-ethnicity, rates were highest among Blacks, followed by Whites, then Hispanics, and lowest among Asians. Rates were highest in the Northeast, followed by the South, then the Midwest, and lowest in the West.

Among commercial insurance enrollees, emergency department visit rates with diverticular disease (all-listed diagnoses) increased with age and were higher among women compared with men (Table 103). Among persons with known race-ethnicity, rates were highest among Blacks, followed by Whites, then Hispanics, and lowest among Asians. Rates were highest in the Northeast, followed by the South, then the West, and lowest in the Midwest.

Among commercial insurance enrollees, hospital discharge rates with diverticular disease (all-listed diagnoses) increased with age and were higher among women compared with men (Table 104). Among persons with known race-ethnicity, rates were highest among Blacks, followed by Whites, then Hispanics, and lowest among Asians. Rates were highest in the Northeast, followed by the South, then the Midwest, and lowest in the West.

Among Medicare beneficiaries, the claims-based prevalence of diverticular disease (based on all-listed diagnoses) was 7.1% (Table 105). Prevalence peaked among persons 75–79 years and was higher among women and Whites. It was highest in the South and Northeast, followed by Midwest, and lowest in the West.

Among Medicare beneficiaries, ambulatory care visit rates with diverticular disease (all-listed diagnoses) peaked among persons 75 to 79 years and were higher among women compared with men and Whites compared with Blacks (Table 106). Rates were higher in the South and Northeast compared with the Midwest and West.

Among Medicare beneficiaries, emergency department visit rates with diverticular disease (all-listed diagnoses) increased with age and were higher among women compared with men and Blacks compared with Whites (Table 107). Rates were highest in the South, followed by the Northeast, then the Midwest, and lowest in the West.

Among Medicare beneficiaries, hospital discharge rates with diverticular disease (all-listed diagnoses) increased with age and were higher among women compared with men and Blacks compared with Whites (Table 108). Rates were lower in the West compared with the South, Northeast, and Midwest.

Hemorrhoids contributed to 4.0 million ambulatory visits (2015) (Table 109). Ambulatory care visit rates (all-listed diagnoses) were similar among middle-aged and older adults. Age-adjusted rates were higher among men compared with women, Blacks compared with Whites, and Hispanics compared with non-Hispanics.

Hemorrhoids contributed to 603,000 emergency department visits in 2018 (Table 110). Emergency department visit rates (all-listed diagnoses) increased with age. Age-adjusted rates were higher among men compared with women.

Hemorrhoids contributed to 347,000 hospital discharges in 2018 (Table 111). Hospital discharge rates (all-listed diagnoses) increased with age. Age-adjusted rates were higher among men compared with women, Blacks compared with Whites, and Hispanics compared with non-Hispanics. Between 2004 and 2018, age-adjusted hospital discharge rates (per 100,000) with an all-listed diagnosis decreased by 12% from 104 to 91.(4,6)

Hemorrhoids contributed to <1,000 deaths in 2019 (Table 112). Mortality from hemorrhoids was rare, but occasionally occurred among older adults. Between 2004 and 2019, mortality with hemorrhoids as underlying or other cause remained uncommon.(4)

Among privately insured enrollees, the claims-based prevalence of hemorrhoids (based on all-listed diagnoses) was 2.7% (Table 113). Prevalence increased with age until the oldest age group and was higher among women. It was highest among Blacks, followed by Whites and Hispanics, and lowest among Asians. It was highest in the Northeast, followed by the South, and lowest in the Midwest and West.

Among commercial insurance enrollees, ambulatory care visit rates with hemorrhoids (all-listed diagnoses) increased with age until 75 years and were higher among women compared with men (Table 114). Among persons with known race-ethnicity, rates were highest among Blacks, followed by Hispanics, then Whites, and lowest among Asians. Rates were highest in the Northeast, followed by the South, then the West, and lowest in the Midwest.

Among commercial insurance enrollees, emergency department visit rates with hemorrhoids (all-listed diagnoses) peaked among persons 55 to 64 years and were higher among women compared with men (Table 115). Among persons with known race-ethnicity, rates were highest among Blacks, followed by Whites, then Hispanics, and lowest among Asians. Rates were highest in the Northeast, followed by the South, then the Midwest, and lowest in the West.

Among commercial insurance enrollees, hospital discharge rates with hemorrhoids (all-listed diagnoses) increased with age and were higher among women compared with men (Table 116). Among persons with known race-ethnicity, rates were highest among Blacks, followed by Whites and Hispanics, and lowest among Asians. Rates were highest in the Northeast, followed by the South, then the Midwest, and lowest in the West.

Among Medicare beneficiaries, the claims-based prevalence of hemorrhoids (based on all-listed diagnoses) was 4.2% (Table 117). Prevalence peaked among persons 70–74 years and was higher among women and Blacks. It was highest in the Northeast, followed by the South, then the West, and lowest in the Midwest.

Among Medicare beneficiaries, ambulatory care visit rates with hemorrhoids (all-listed diagnoses) peaked among persons 70 to 74 years, were higher among women compared with men, and did not differ by race (Table 118). Rates were highest in the Northeast, followed by the South, then the West, and lowest in the Midwest.

Among Medicare beneficiaries, emergency department visit rates with hemorrhoids (all-listed diagnoses) increased with age and were higher among women compared with men and Blacks compared with Whites (Table 119). Rates were highest in the Northeast, followed by the South, then the Midwest, and lowest in the West.

Among Medicare beneficiaries, hospital discharge rates with hemorrhoids (all-listed diagnoses) increased with age and were higher among women compared with men and Blacks compared with Whites (Table 120). Rates were highest in the Northeast, followed by the South, then the Midwest, and lowest in the West.

Liver disease (acute and chronic) contributed to 4.2 million ambulatory visits (2015) (Table 121). Ambulatory care visit rates (all-listed diagnoses) were highest among persons 55–64 years. Age-adjusted ambulatory care visit rates were higher among women compared with men, Blacks compared with Whites, and non-Hispanics compared with Hispanics.

Liver disease (acute and chronic) contributed to 2.2 million emergency department visits in 2018 (Table 122). Emergency department visit rates (all-listed diagnoses) were highest among persons 55–64 years. Age-adjusted emergency department visit rates were higher among men compared with women.

Liver disease (acute and chronic) contributed to 1.7 million hospital discharges in 2018 (Table 123). Hospital discharge rates (all-listed diagnoses) increased with age until the oldest age group. Age-adjusted hospital discharge rates were higher among men compared with women, Whites compared with Blacks, and Hispanics compared with non-Hispanics. Between 2004 and 2018, age-adjusted hospital discharge rates (per 100,000) with an all-listed diagnosis increased by 72% from 259 to 446.(7)

Liver disease (acute and chronic) contributed to 107,000 deaths in 2019 (Table 124). Mortality rates (underlying or other cause) increased with age. Age-adjusted mortality rates were higher among men, Whites, and Hispanics. Between 2004 and 2019, age-adjusted mortality rates (per 100,000) with liver disease as underlying or other cause increased by 7% from 24.9 to 26.8.(7)

Among privately insured enrollees, the claims-based prevalence of liver disease (acute and chronic) (based on all-listed diagnoses) was 1.9% (Table 125). Prevalence increased with age until the oldest age groups and did not differ by sex. It was highest among Hispanics, followed by Blacks, Whites, and Asians. It was highest in the South, followed by the Northeast, then the West, and lowest in the Midwest.

Among commercial insurance enrollees, ambulatory care visit rates with liver disease (acute and chronic) (all-listed diagnoses) peaked among persons 55 to 64 years and were higher among women compared with men (Table 126). Among persons with known race-ethnicity, rates were much higher among Hispanics, followed by Blacks and Whites, and lowest among Asians. Rates were highest in the South, followed by the Northeast, then the West, and lowest in the Midwest.

Among commercial insurance enrollees, emergency department visit rates with liver disease (acute and chronic) (all-listed diagnoses) increased with age until 75 years and were higher among men compared with women (Table 127). Among persons with known race-ethnicity, rates were highest among Hispanics, followed by Blacks, then Whites, and much lower among Asians. Rates were highest in the South, followed by the Northeast, then the West, and lowest in the Midwest.

Among commercial insurance enrollees, hospital discharge rates with liver disease (acute and chronic) (all-listed diagnoses) increased with age until 75 years were higher among men compared with women (Table 128). Among persons with known race-ethnicity, rates were highest among Blacks, followed by Hispanics, then Whites, and much lower among Asians. Rates were highest in the South, followed by the Northeast, then the Midwest, and lowest in the West.

Among Medicare beneficiaries, the claims-based prevalence of liver disease (acute and chronic) (based on all-listed diagnoses) was 3.6% (Table 129). Prevalence decreased with age and did not differ by sex or race. It was highest in the Northeast and South, followed by the West, and lowest in the Midwest.

Among Medicare beneficiaries, ambulatory care visit rates with liver disease (acute and chronic) (all-listed diagnoses) decreased with age, differed little by sex, and were higher among Whites compared with Blacks (Table 130). Rates were lower in the Midwest compared with the Northeast, South, and West.

Among Medicare beneficiaries, emergency department visit rates with liver disease (acute and chronic) (all-listed diagnoses) were lowest among persons 85 years and over and were higher among men compared with women and Blacks compared with Whites (Table 131). Rates were highest in the South, followed by the West, then the Northeast, and lowest in the Midwest.

Among Medicare beneficiaries, hospital discharge rates with liver disease (acute and chronic) (all-listed diagnoses) were lowest among persons 85 years and over and were higher among men compared with women and Blacks compared with Whites (Table 132). Rates were higher in the South compared with the Northeast, Midwest, and West.

Hepatitis A contributed to 12,000 ambulatory visits (2015) (Table 133). Ambulatory care visits were uncommon, and rates (all-listed diagnoses) were highest among persons 55–64 years. Age-adjusted ambulatory care visit rates were higher among women compared with men, Whites compared with Blacks, and Hispanics compared with non-Hispanics.

Hepatitis A contributed to 19,000 emergency department visits in 2018 (Table 134). Emergency department visit rates (all-listed diagnoses) were uncommon among children and highest among persons 25–44 years. Age-adjusted emergency department visit rates were higher among men compared with women.

Hepatitis A contributed to 15,000 hospital discharges in 2018 (Table 135). Hospital discharges were uncommon among children and rates (all-listed diagnoses) were highest among persons 25–44 years. Age-adjusted hospital discharge rates were higher among men compared with women, Whites compared with Blacks, and non-Hispanics compared with Hispanics. Between 2004 and 2018, age-adjusted hospital discharge rates (per 100,000) with an all-listed diagnosis increased by a third from 3 to 4.(4)

Hepatitis A contributed to <1,000 deaths in 2019 (Table 136). Mortality was uncommon among children and young adults and rates (underlying or other cause) were highest among persons 75 years and over. Age-adjusted mortality rates were higher among men, Whites, and non-Hispanics. Between 2004 and 2019, mortality with hepatitis A as underlying or other cause remained uncommon.(4)

Among commercial insurance enrollees, the claims-based prevalence of hepatitis A (based on all-listed diagnoses) was <0.1% (Table 137).

Among commercial insurance enrollees, ambulatory care visit rates with hepatitis A (all-listed diagnoses) increased with age until 75 years and did not differ by sex (Table 138). Among persons with known race-ethnicity, rates were highest among Hispanics, followed by Asians, Blacks, and Whites. Rates were highest in the Northeast, followed by the South, then the West, and lowest in the Midwest.

Among commercial insurance enrollees, the emergency department visit rate with hepatitis A (all-listed diagnoses) was less than 1 per 100,000 (Table 139).

Among commercial insurance enrollees, hospital discharge rates with hepatitis A (all-listed diagnoses) increased with age and differed little by sex (Table 140). Among persons with known race-ethnicity, rates were highest among Blacks, followed by Whites and Hispanics, and lowest among Asians. Rates were highest in the South, followed by the Northeast, then the West, and lowest in the Midwest.

Among Medicare beneficiaries, the claims-based prevalence of hepatitis A (based on all-listed diagnoses) was <0.1% (Table 141).

Among Medicare beneficiaries, ambulatory care visits with hepatitis A were uncommon among persons over 80 years and Blacks (Table 142). Rates (all-listed diagnoses) were higher among men compared with women. Rates were much higher in the South, intermediate in the Northeast and West, and lowest in the Midwest.

Among Medicare beneficiaries, emergency department visits with hepatitis A were uncommon among Blacks (Table 143). Rates (all-listed diagnoses) were highest among persons 80 to 84 years and higher among men compared with women. Rates were higher in the South compared with Northeast, West, and Midwest.

Among Medicare beneficiaries, hospital discharges with hepatitis A were uncommon among persons over 80 years and Blacks (Table 144). Rates (all-listed diagnoses) were higher among men compared with women. Rates were higher in the South and West compared with the Northeast and Midwest.

Hepatitis B contributed to 764,000 ambulatory visits (2015) (Table 145). Ambulatory care visits were uncommon among children and rates (all-listed diagnoses) were much higher among persons 75 years and over compared to younger age groups. Age-adjusted ambulatory care visit rates were higher among men compared with women, Blacks compared with Whites, and non-Hispanics compared with Hispanics.

Hepatitis B contributed to 89,000 emergency department visits in 2018 (Table 146). Emergency department visits were uncommon among children and rates (all-listed diagnoses) peaked at 55–64 years. Age-adjusted rates were higher among men compared with women.

Hepatitis B contributed to 82,000 hospital discharges in 2018 (Table 147). Hospital discharges were uncommon among children and rates (all-listed diagnoses) peaked at 55–64 years. Age-adjusted rates were higher among men compared with women, Blacks compared with Whites, and non-Hispanics compared with Hispanics. Between 2004 and 2018, age-adjusted hospital discharge rates (per 100,000) with an all-listed diagnosis remained stable at 23.(4)

Hepatitis B contributed to 2,000 deaths in 2019 (Table 148). Mortality was uncommon among the youngest age groups after which rates (underlying or other cause) increased with age until the oldest age group. Like health care use rates, age-adjusted mortality rates were higher among men, Blacks, and non-Hispanics. Between 2004 and 2019, age-adjusted mortality rates (per 100,000) with hepatitis B as underlying or other cause decreased by a third from 0.6 to 0.4.(4)

Among privately insured enrollees, the claims-based prevalence of hepatitis B (based on all-listed diagnoses) was 0.1% (Table 149). Hepatitis B was uncommon among children and adolescents and the youngest adults and prevalence was highest among persons 55–74 years. Prevalence did not differ by sex and was highest among Asians, similar among Blacks and Hispanics, and lowest among Whites. It was highest in the Northeast and lowest in the Midwest.

Among commercial insurance enrollees, ambulatory care visit rates with hepatitis B (all-listed diagnoses) increased with age until 75 years and were higher among men compared with women (Table 150). Among persons with known race-ethnicity, rates were much higher among Asians, followed by Blacks, then Hispanics, and lowest among Whites. Rates were highest in the Northeast, followed by the West, then the South, and lowest in the Midwest.

Among commercial insurance enrollees, emergency department visit rates with hepatitis B (all-listed diagnoses) were highest among persons 25 to 44 years and differed little by sex (Table 151). Among persons with known race-ethnicity, rates were much higher among Asians compared with other race-ethnicities. Rates were higher in the Northeast and South compared with the Midwest and West.

Among commercial insurance enrollees, hospital discharge rates with hepatitis B (all-listed diagnoses) increased with age until 75 years and were higher among men compared with women (Table 152). Among persons with known race-ethnicity, rates were highest among Asians, followed by Blacks, and lowest among Whites and Hispanics. Rates were highest in the Northeast, followed by the South, then the West, and lowest in the Midwest.

Among Medicare beneficiaries, the claims-based prevalence of hepatitis B (based on all-listed diagnoses) was 0.1% (Table 153). Prevalence was highest among persons 65–74 years and was higher among men and Blacks. It was highest in the West, and lowest in the Midwest and South.

Among Medicare beneficiaries, ambulatory care visit rates with hepatitis B (all-listed diagnoses) generally decreased with age and were higher among men compared with women and over four times higher among Blacks compared with Whites (Table 154). Rates were highest in the West, followed by the Northeast, then the South, and lowest in the Midwest.

Among Medicare beneficiaries, emergency department visit rates with hepatitis B (all-listed diagnoses) were highest among persons 65 to 69 years and were higher among men compared with women and over six times higher among Blacks compared with Whites (Table 155). Rates were higher in the West compared with the South, Midwest, and Northeast.

Among Medicare beneficiaries, hospital discharge rates with hepatitis B (all-listed diagnoses) were highest among persons 65 to 69 years and were higher among men compared with women and five times higher among Blacks compared with Whites (Table 156). Rates were highest in the West, followed by the Northeast, then the South, and lowest in the Midwest.

Hepatitis C contributed to 2.1 million ambulatory visits (2015) (Table 157). Ambulatory care visit rates (all-listed diagnoses) peaked among persons 55–64 years. Age-adjusted rates were higher among men compared with women, Blacks compared with Whites, and Hispanics compared with non-Hispanics.

Hepatitis C contributed to 865,000 emergency department visits in 2018 (Table 158). Emergency department visit rates (all-listed diagnoses) peaked among persons 55–64 years. Age-adjusted rates were higher among men compared with women.

Hepatitis C contributed to 600,000 hospital discharges in 2018 (Table 159). Hospital discharge rates (all-listed diagnoses) peaked among persons 55–64 years. Age-adjusted rates were higher among men compared with women, Blacks compared with Whites, and non-Hispanics compared with Hispanics. Between 2004 and 2018, age-adjusted hospital discharge rates (per 100,000) with an all-listed diagnosis increased by 12% from 143 to 160.(7)

Hepatitis C contributed to 14,000 deaths in 2019 (Table 160). Mortality rates (underlying or other cause) peaked among persons 55–64 years. Age-adjusted mortality rates were higher among men, Blacks, and non-Hispanics. Between 2004 and 2019, age-adjusted mortality rates (per 100,000) with hepatitis C as underlying or other cause decreased by 13% from 3.8 to 3.3. (4)

Among privately insured enrollees, the claims-based prevalence of hepatitis C (based on all-listed diagnoses) was 0.3% (Table 161). Prevalence peaked among persons 55–74 years and was higher among men. It was highest among Blacks, followed by Hispanics, and lowest among Whites and Asians. It was highest in the South and lowest in the Midwest.

Among commercial insurance enrollees, ambulatory care visit rates with hepatitis C (all-listed diagnoses) peaked among persons 55 to 64 years and were higher among men compared with women (Table 162). Among persons with known race-ethnicity, rates were much higher among Blacks, followed by Hispanics, then Whites, and lowest among Asians. Rates were highest in the South, followed by the Northeast, then the West, and lowest in the Midwest.

Among commercial insurance enrollees, emergency department visit rates with hepatitis C (all-listed diagnoses) peaked among persons 55 to 64 years and were higher among men compared with women (Table 163). Among persons with known race-ethnicity, rates were highest among Blacks, followed by Whites and Hispanics, and lowest among Asians. Rates were higher in the Northeast and South compared with the West and Midwest.

Among commercial insurance enrollees, hospital discharge rates with hepatitis C (all-listed diagnoses) peaked among persons 55 to 64 years and were higher among men compared with women (Table 164). Among persons with known race-ethnicity, rates were highest among Blacks, followed by Hispanics, then Whites, and lowest among Asians. Rates were highest in the South, followed by the Northeast, then the West, and lowest in the Midwest.

Among Medicare beneficiaries, the claims-based prevalence of hepatitis C (based on all-listed diagnoses) was 0.5% (Table 165). Prevalence decreased with age and was higher among men and Blacks. It was highest in the West, and lowest in the Midwest.

Among Medicare beneficiaries, ambulatory care visit rates with hepatitis C (all-listed diagnoses) decreased with age and were twice as high among men compared with women and over five times higher among Blacks compared with Whites (Table 166). Rates were highest in the West, followed by the Northeast and South, and lowest in the Midwest.

Among Medicare beneficiaries, emergency department visit rates with hepatitis C (all-listed diagnoses) decreased with age and were over twice as high among men compared with women and seven times higher among Blacks compared with Whites (Table 167). Rates were highest in the West, followed by the Northeast, then the South, and lowest in the Midwest.

Among Medicare beneficiaries, hospital discharge rates with hepatitis C (all-listed diagnoses) decreased with age and were over twice as high among men compared with women and over six times higher among Blacks compared with Whites (Table 168). Rates were highest in the Northeast, followed by the West, then the South, and lowest in the Midwest.

Gallstones contributed to an estimated 2.2 million ambulatory visits (2015) (Table 169). Ambulatory care visits rates (all-listed diagnoses) were highest among persons 65–74 years. Age-adjusted rates were higher among women compared with men, Whites compared with Blacks, and Hispanics compared with non-Hispanics.

Gallstones contributed to an estimated 1.1 million emergency department visits in 2018 (Table 170). Emergency department visit rates (all-listed diagnoses) increased with age. Age-adjusted rates were higher among women compared with men.

Gallstones contributed to an estimated 615,000 hospital discharges in 2018 (Table 171). Hospital discharge rates (all-listed diagnoses) increased with age. Age-adjusted rates were higher among women compared with men, Whites compared with Blacks, and Hispanics compared with non-Hispanics. Between 2004 and 2018, age-adjusted hospital discharge rates (per 100,000) with an all-listed diagnosis decreased by 21% from 212 to 167.(7) Hospital discharge rates underestimate the actual burden because most hospitalizations with gallstones were for cholecystectomy and a high proportion of cholecystectomies were performed laparoscopically without an overnight stay, and therefore, were not included in hospitalization statistics.

Gallstones contributed to an estimated 2,000 deaths in 2019 (Table 172). Mortality was uncommon among the youngest age groups after which rates (underlying or other cause) increased with age. Age-adjusted mortality rates were higher among men and did not differ by race or ethnicity. Between 2004 and 2019, age-adjusted mortality rates (per 100,000) with gallstone disease as underlying or other cause decreased by 43% from 0.7 to 0.4.(4)

Among privately insured enrollees, the claims-based prevalence of gallstones (based on all-listed diagnoses) was 0.7% (Table 173). Prevalence increased with age and was higher among women. It was highest among Hispanics, similar among Whites and Blacks, and lowest among Asians. It differed little by region.

Among commercial insurance enrollees, ambulatory care visit rates with gallstones (all-listed diagnoses) increased with age and were higher among women compared with men (Table 174). Among persons with known race-ethnicity, rates were highest among Hispanics, followed by Blacks, then Whites, and lowest among Asians. Rates were highest in the South, followed by the Northeast, then the West, and lowest in the Midwest.

Among commercial insurance enrollees, emergency department visit rates with gallstones (all-listed diagnoses) increased with age and were higher among women compared with men (Table 175). Among persons with known race-ethnicity, rates were highest among Blacks and Hispanics, followed by Whites, and lowest among Asians. Rates were lower in the Midwest compared with the South, West, and Northeast.

Among commercial insurance enrollees, hospital discharge rates with gallstones (all-listed diagnoses) increased with age and differed little by sex (Table 176). Among persons with known race-ethnicity, rates were highest among Hispanics, followed by Blacks, then Whites, and lowest among Asians. Rates were highest in the Northeast, followed by the South, then the West, and lowest in the Midwest.

Among Medicare beneficiaries, the claims-based prevalence of gallstones (based on all-listed diagnoses) was 1.7% (Table 177). Prevalence increased with age, was higher among men, and did not differ by race. It was lower in the Midwest compared with other regions.

Among Medicare beneficiaries, ambulatory care visit rates with gallstones (all-listed diagnoses) increased with age until 85 years and were higher among women compared with men and Whites compared with Blacks (Table 178). Rates were highest in the Northeast, intermediate in the South and West, and lowest in the Midwest.

Among Medicare beneficiaries, emergency department visit rates with gallstones (all-listed diagnoses) increased with age and were higher among men compared with women and Blacks compared with Whites (Table 179). Rates were highest in the South, followed by the West, then the Northeast, and lowest in the Midwest.

Among Medicare beneficiaries, hospital discharge rates with gallstones (all-listed diagnoses) increased with age and were higher among men compared with women and Blacks compared with Whites (Table 180). Rates were higher in the South compared with the Northeast, West, and Midwest.

Acute pancreatitis contributed to 897,000 ambulatory visits (2015) (Table 181). Ambulatory care visit rates (all-listed diagnoses) peaked among persons 55–64 years. Age-adjusted ambulatory care visit rates were higher among women compared with men, Blacks compared with Whites and among non-Hispanics compared with Hispanics.

Acute pancreatitis contributed to 585,000 emergency department visits in 2018 (Table 182). Emergency department visit rates (all-listed diagnoses) were similar among middle-aged and older adults. Age-adjusted emergency department visit rates were higher among men.

Acute pancreatitis contributed to 448,000 hospital discharges in 2018 (Table 183). Hospital discharge rates (all-listed diagnoses) were highest among persons 75 years and over. Age-adjusted rates were higher among women compared with men, Blacks compared with Whites and among non-Hispanics compared with Hispanics.

Acute pancreatitis contributed to 6,000 deaths in 2019 (Table 184). Mortality was uncommon among the youngest age groups after which rates (underlying or other cause) increased with age. Age-adjusted mortality rates were higher among men, Blacks, and non-Hispanics.

Among privately insured enrollees, the claims-based prevalence of acute pancreatitis (based on all-listed diagnoses) was 0.2% (Table 185). Prevalence was uncommon among children and adolescents and the youngest adults and was highest among persons 55 years and over. It did not differ by sex and was highest among Blacks, similar among Whites and Hispanics, and lowest among Asians. It did not differ by region.

Among commercial insurance enrollees, ambulatory care visit rates with acute pancreatitis (all-listed diagnoses) peaked among persons 55 to 64 years and were higher among men compared with women (Table 186). Among persons with known race-ethnicity, rates were highest among Blacks, followed by Whites and Hispanics, and lowest among Asians. Rates were higher in the South compared with the West, Northeast, and Midwest.

Among commercial insurance enrollees, emergency department visit rates with acute pancreatitis (all-listed diagnoses) increased with age until 75 years and differed little by sex (Table 187). Among persons with known race-ethnicity, rates were highest among Blacks, followed by Hispanics and Whites, and lowest among Asians. Rates were highest in the South, followed by the Northeast, then the West, and lowest in the Midwest.

Among commercial insurance enrollees, hospital discharge rates with acute pancreatitis (all-listed diagnoses) increased with age until 65 years and were higher among men compared with women (Table 188). Among persons with known race-ethnicity, rates were highest among Blacks, followed by Hispanics, then Whites, and lowest among Asians. Rates were highest in the South, followed by the Northeast, then the Midwest, and lowest in the West.

Among Medicare beneficiaries, the claims-based prevalence of acute pancreatitis (based on all-listed diagnoses) was 0.4% (Table 189). Prevalence differed little with age and was higher among men and Blacks. It differed little by region.

Among Medicare beneficiaries, ambulatory care visit rates with acute pancreatitis (all-listed diagnoses) were relatively stable with age and were higher among men compared with women and Whites compared with Blacks (Table 190). Rates were higher in the Midwest and South compared with the Northeast and West.

Among Medicare beneficiaries, emergency department visit rates with acute pancreatitis (all-listed diagnoses) generally increased with age and were higher among men compared with women and Blacks compared with Whites (Table 191). Rates were highest in the South, followed by the West, then the Midwest, and lowest in the Northeast.

Among Medicare beneficiaries, hospital discharge rates with acute pancreatitis (all-listed diagnoses) generally increased with age and were higher among men compared with women and Blacks compared with Whites (Table 192). Rates were highest in the South and Midwest, followed by the West, and lowest in the Northeast.

Chronic pancreatitis contributed to 245,000 ambulatory visits (2015) (Table 193). Ambulatory care visit rates (all-listed diagnoses) peaked in middle age. Age-adjusted rates were higher among men compared with women, Whites compared with Blacks, and Hispanics compared with non-Hispanics.

Chronic pancreatitis contributed to 263,000 emergency department visits in 2018 (Table 194). Emergency department visit rates (all-listed diagnoses) peaked in middle age. Age-adjusted rates were higher among men compared with women.

Chronic pancreatitis contributed to 192,000 hospital discharges in 2018 (Table 195). Hospital discharge rates (all-listed diagnoses) peaked in middle age. Age-adjusted rates were higher among men compared with women, Blacks compared with Whites, and non-Hispanics compared with Hispanics.

Chronic pancreatitis contributed to 2,000 deaths in 2019 (Table 196). Mortality was uncommon among the youngest age groups after which rates (underlying or other cause) increased with age. Age-adjusted mortality rates were higher among men, Blacks, and non-Hispanics.

Among privately insured enrollees, the claims-based prevalence of chronic pancreatitis (based on all-listed diagnoses) was 0.1% (Table 197). Chronic pancreatitis was uncommon among children and adolescents and the youngest adults and prevalence was highest among persons 55 years and over. Prevalence did not differ by sex and was highest among Blacks and similar among Whites, Hispanics, and Asians. It did not differ by region.

Among commercial insurance enrollees, ambulatory care visit rates with chronic pancreatitis (all-listed diagnoses) peaked among persons 55 to 64 years and were higher among men compared with women (Table 198). Among persons with known race-ethnicity, rates were highest among Blacks, followed by Whites, then Hispanics, and lowest among Asians. Rates were higher in the South compared with the Northeast, West, and Midwest.

Among commercial insurance enrollees, emergency department visit rates with chronic pancreatitis (all-listed diagnoses) peaked among persons 55 to 64 years and were higher among men compared with women (Table 199). Among persons with known race-ethnicity, rates were highest among Blacks, followed by Whites, then Hispanics, and lowest among Asians. Rates were highest in the South, followed by the Northeast, and lowest in the Midwest and West.

Among commercial insurance enrollees, hospital discharge rates with chronic pancreatitis (all-listed diagnoses) peaked among persons 55 to 64 years and were higher among men compared with women (Table 200). Among persons with known race-ethnicity, rates were highest among Blacks, followed by Whites, then Hispanics, and lowest among Asians. Rates were highest in the South, followed by the Northeast and Midwest, and lowest in the West.

Among Medicare beneficiaries, the claims-based prevalence of chronic pancreatitis (based on all-listed diagnoses) was 0.2% (Table 201). Prevalence did not differ by age or sex and was higher among Blacks. It did not differ by region.

Among Medicare beneficiaries, ambulatory care visit rates with chronic pancreatitis (all-listed diagnoses) were generally stable before declining after 85 years and were higher among men compared with women and Blacks compared with Whites (Table 202). Rates were highest in the South, followed by the Midwest, and lowest in the West and Northeast.

Among Medicare beneficiaries, emergency department visit rates with chronic pancreatitis (all-listed diagnoses) were highest among persons 65 to 69 years and were higher among men compared with women and over twice as high among Blacks compared with Whites (Table 203). Rates were highest in the Midwest, followed by the South, then the West, and lowest in the Northeast.

Among Medicare beneficiaries, hospital discharge rates with chronic pancreatitis (all-listed diagnoses) were highest among persons 65 to 69 years and were higher among men compared with women and over twice as high among Blacks compared with Whites (Table 204). Rates were highest in the Midwest, followed by the South, then the Northeast, and lowest in the West.

Celiac disease contributed to 611,000 ambulatory visits (2015) (Table 205). Ambulatory care visit rates (all-listed diagnoses) were highest among persons 55–64 years and second highest among children. Age-adjusted rates were higher among women compared with men, Whites compared with Blacks, and Hispanics compared with non-Hispanics.

Celiac disease contributed to 68,000 emergency department visits in 2018 (Table 206). Emergency department visit rates (all-listed diagnoses) increased with age. Age-adjusted rates were higher among women compared with men.

Celiac disease contributed to 42,000 hospital discharges in 2018 (Table 207). Hospital discharge rates (all-listed diagnoses) increased with age. Age-adjusted rates were higher among women compared with men, Whites compared with Blacks, and non-Hispanics compared with Hispanics.

Celiac disease contributed to <1,000 deaths in 2019 (Table 208). Mortality was uncommon with the highest rate (underlying or other cause) among persons 75 years and over. Age-adjusted mortality rates were higher among women, Whites, and non-Hispanics.

Among privately insured enrollees, the claims-based prevalence of celiac disease (based on all-listed diagnoses) was 0.1% (Table 209). Prevalence was highest among adolescents and the youngest adults and among women. It was highest among Whites, similar among Blacks and Hispanics, and lowest among Asians. It differed little by region.

Among commercial insurance enrollees, ambulatory care visit rates with celiac disease (all-listed diagnoses) were higher among adolescents and the youngest adults compared with other age groups and were over twice as high among women compared with men (Table 210). Among persons with known race-ethnicity, rates were highest among Whites, followed by Hispanics, then Blacks, and lowest among Asians. Rates were highest in the Northeast, followed by the West and Midwest, and lowest in the South.

Among commercial insurance enrollees, emergency department visit rates with celiac disease (all-listed diagnoses) were highest among persons 25 to 44 years and differed little by sex (Table 211). Among persons with known race-ethnicity, rates were higher among Whites compared with other race-ethnicities. Rates were highest in the Northeast, followed by the Midwest and West, and lowest in the South.

Among commercial insurance enrollees, hospital discharge rates with celiac disease (all-listed diagnoses) were highest among persons 75 years and over and were twice as high among women compared with men (Table 212). Among persons with known race-ethnicity, rates were highest among Whites, followed by Blacks, then Hispanics, and lowest among Asians. Rates were highest in the Northeast, followed by the Midwest, then the West, and lowest in the South.

Among Medicare beneficiaries, the claims-based prevalence of celiac disease (based on all-listed diagnoses) was 0.2% (Table 213). Prevalence was lowest in the oldest age group and higher among women and Whites. It was highest in the Northeast, and lowest in the South.

Among Medicare beneficiaries, ambulatory care visit rates with celiac disease (all-listed diagnoses) increased with age until 85 years and were higher among women compared with men and almost five times higher among Whites compared with Blacks (Table 214). Rates were highest in the Northeast, followed by the Midwest, and lowest in the South and West.

Among Medicare beneficiaries, emergency department visits with celiac disease were uncommon among Blacks (Table 215). Rates (all-listed diagnoses) increased with age until 85 years and were twice as high among women compared with men. Rates were higher in the Northeast compared with the Midwest, South, and West.

Among Medicare beneficiaries, hospital discharges with celiac disease were uncommon among Blacks (Table 216). Rates (all-listed diagnoses) increased with age and were higher among women compared with men. Rates were higher in the Northeast compared with the South, Midwest, and West.

Gastrointestinal infections (excluding C. difficile) contributed to 1.6 million ambulatory visits (2015) (Table 217). Ambulatory care visit rates (all-listed diagnoses) were highest among children and adolescents. Age-adjusted rates were higher among women compared with men, Blacks compared with Whites, and Hispanics compared with non-Hispanics.

Gastrointestinal infections (excluding C. difficile) contributed to 698,000 emergency department visits in 2018 (Table 218). Emergency department visit rates (all-listed diagnoses) were highest among children and second highest among persons 75 years and over. Age-adjusted rates were higher among women compared with men.

Gastrointestinal infections (excluding C. difficile) contributed to 240,000 hospital discharges in 2018 (Table 219). Hospital discharge rates (all-listed diagnoses) were higher among children compared with adolescents and younger adults and then increased with age. Age-adjusted rates were higher among women compared with men, Blacks compared with Whites, and non-Hispanics compared with Hispanics.

Gastrointestinal infections (excluding C. difficile) contributed to 5,000 deaths in 2019 (Table 220). Mortality rates (underlying or other cause) were higher among children compared with adolescents and younger adults and then increased with age. Age-adjusted mortality rates were higher among women and non-Hispanics and did not differ by race.

Among privately insured enrollees, the claims-based prevalence of gastrointestinal infections (excluding C. difficile) (based on all-listed diagnoses) was 0.5% (Table 221). Prevalence was highest among children and higher among women. It was higher among Blacks and Hispanics compared with Whites and Asians. It was highest in the South, and lowest in the Midwest and West.

Among commercial insurance enrollees, ambulatory care visit rates with gastrointestinal infections (excluding C. difficile) (all-listed diagnoses) were highest among children and were higher among women compared with men (Table 222). Among persons with known race-ethnicity, rates were highest among Hispanics, followed by Blacks, then Asians, and lowest among Whites. Rates were highest in the South, followed by the Northeast, and lowest in the Midwest and West.

Among commercial insurance enrollees, emergency department visit rates with gastrointestinal infections (excluding C. difficile) (all-listed diagnoses) increased with age and were higher among women compared with men (Table 223). Among persons with known race-ethnicity, rates were highest among Blacks, followed by Hispanics, then Whites, and lowest among Asians. Rates were highest in the South, followed by the Midwest, then the Northeast, and lowest in the West.

Among commercial insurance enrollees, hospital discharge rates with gastrointestinal infections (excluding C. difficile) (all-listed diagnoses) were higher among children compared with adolescents and the youngest adults and then increased with age and were higher among women compared with men (Table 224). Among persons with known race-ethnicity, rates were highest among Blacks, followed by Whites and Hispanics, and lowest among Asians. Rates were highest in the Northeast, followed by the South, then the Midwest, and lowest in the West.

Among Medicare beneficiaries, the claims-based prevalence of gastrointestinal infections (excluding C. difficile) (based on all-listed diagnoses) was 0.8% (Table 225). Prevalence was highest among persons 75 years and over, higher among women, and did not differ by race. It was highest in the Northeast and South, and lowest in the Midwest.

Among Medicare beneficiaries, ambulatory care visit rates with gastrointestinal infections (excluding C. difficile) (all-listed diagnoses) increased with age and were higher among women compared with men and Whites compared with Blacks (Table 226). Rates were highest in the Northeast and South, followed by the West, and lowest in the Midwest.

Among Medicare beneficiaries, emergency department visit rates with gastrointestinal infections (excluding C. difficile) (all-listed diagnoses) increased with age and were higher among women compared with men and Blacks compared with Whites (Table 227). Rates were highest in the Northeast, followed by the Midwest, then the South, and lowest in the West.

Among Medicare beneficiaries, hospital discharge rates with gastrointestinal infections (excluding C. difficile) (all-listed diagnoses) increased with age and were higher among women compared with men but differed little by race (Table 228). Rates were highest in the Northeast, followed by the Midwest, then the South, and lowest in the West.

Clostridium difficile contributed to 249,000 ambulatory visits (2015) (Table 229). Ambulatory care visit rates (all-listed diagnoses) were higher among children compared with adolescents and younger adults and then increased with age. Age-adjusted rates were higher among women compared with men, Whites compared with Blacks, and non-Hispanics compared with Hispanics.

Clostridium difficile contributed to 293,000 emergency department visits in 2018 (Table 230). Emergency department visit rates (all-listed diagnoses) increased with age. Age-adjusted rates were higher among women compared with men.

Clostridium difficile contributed to 307,000 hospital discharges in 2018 (Table 231). Hospital discharge rates (all-listed diagnoses) increased with age. Age-adjusted rates were higher among women compared with men, Blacks compared with Whites, and non-Hispanics compared with Hispanics.

Clostridium difficile contributed to 8,000 deaths in 2019 (Table 232). Mortality was uncommon among the youngest age groups after which rates (underlying or other cause) increased with age. Age-adjusted mortality rates were higher among men, Whites, and non-Hispanics.

Among privately insured enrollees, the claims-based prevalence of Clostridium difficile (based on all-listed diagnoses) was 0.1% (Table 233). C. difficile was uncommon among children and adolescents and the youngest adults and then prevalence increased with age. Prevalence was higher among women and higher among Whites and Blacks compared with Hispanics and Asians. It differed little by region.

Among commercial insurance enrollees, ambulatory care visit rates with Clostridium difficile (all-listed diagnoses) increased with age and were higher among women compared with men (Table 234). Among persons with known race-ethnicity, rates were highest among Whites, followed by Blacks, then Hispanics, and lowest among Asians. Rates were highest in the Northeast, followed by the Midwest and West, and lowest in the South.

Among commercial insurance enrollees, emergency department visit rates with Clostridium difficile (all-listed diagnoses) increased with age and were higher among women compared with men (Table 235). Among persons with known race-ethnicity, rates were highest among Whites, followed by Blacks, then Hispanics, and lowest among Asians. Rates were higher in the Northeast and Midwest compared with the South and West.

Among commercial insurance enrollees, hospital discharge rates with Clostridium difficile (all-listed diagnoses) increased with age and were higher among women compared with men (Table 236). Among persons with known race-ethnicity, rates were highest among Blacks, followed by Whites, then Hispanics, and lowest among Asians. Rates were highest in the Northeast, followed by the South, then the Midwest, and lowest in the West.

Among Medicare beneficiaries, the claims-based prevalence of Clostridium difficile (based on all-listed diagnoses) was 0.5% (Table 237). Prevalence increased with age, was higher among women, and did not differ by race. It differed little by region.

Among Medicare beneficiaries, ambulatory care visit rates with Clostridium difficile (all-listed diagnoses) increased with age and were higher among women compared with men and Whites compared with Blacks (Table 238). Rates were highest in the Northeast, followed by the Midwest, then the South, and lowest in the West.

Among Medicare beneficiaries, emergency department visit rates with Clostridium difficile (all-listed diagnoses) increased with age and were higher among women compared with men and Blacks compared with Whites (Table 239). Rates were highest in the Northeast and Midwest, followed by the South, and lowest in the West.

Among Medicare beneficiaries, hospital discharge rates with Clostridium difficile (all-listed diagnoses) increased with age and were higher among women compared with men and Blacks compared with Whites (Table 240). Rates were highest in the Midwest, followed by the Northeast and South, and lowest in the West.

Esophageal cancer accounted for 16,000 incident cases in 2016 (Table 241). Esophageal cancer was rare among children and adolescents and incidence rates increased with age throughout adulthood. Age-adjusted incidence rates were much higher among men compared with women, and higher among Whites compared with Blacks, and non-Hispanics compared with Hispanics.

Esophageal cancer accounted contributed to 40,000 hospital discharges in 2018 (Table 242). Esophageal cancer hospitalization was rare among children and adolescents and hospital discharge rates increased with age throughout adulthood. Age-adjusted hospital discharge rates (all-listed diagnoses) were much higher among men compared with women, and higher among Whites compared with Blacks, and non-Hispanics compared with Hispanics.

Esophageal cancer contributed to 18,000 deaths in 2019 (Table 243). Esophageal cancer mortality was rare among children and adolescents and mortality rates increased with age throughout adulthood. Age-adjusted mortality rates (underlying or other cause) were much higher among men compared with women, and higher among Whites compared with Blacks, and non-Hispanics compared with Hispanics.

Gastric cancer accounted for 24,000 incident cases in 2016 (Table 244). Gastric cancer was rare among children and adolescents and incidence rates increased with age throughout adulthood. Age-adjusted incidence rates were higher among men compared with women, Blacks compared with Whites, and Hispanics compared with non-Hispanics.

Gastric cancer contributed to 41,000 hospital discharges in 2018 (Table 245). Gastric cancer hospitalization was rare among children and adolescents and hospital discharge rates increased with age throughout adulthood. Age-adjusted hospital discharges rates (all-listed diagnoses) were higher among men compared with women, Blacks compared with Whites, and Hispanics compared with non-Hispanics.

Gastric cancer contributed to 12,000 deaths in 2019 (Table 246). Gastric cancer mortality was rare among children and adolescents and mortality rates increased with age throughout adulthood. Age-adjusted mortality rates (underlying or other cause) were higher among men compared with women, Blacks compared with Whites, and Hispanics compared with non-Hispanics.

Cancer of the small intestine accounted for 9,000 incident cases in 2016 (Table 247). Small intestinal cancer was rare among children and adolescents and incidence rates increased with age throughout adulthood. Age-adjusted incidence rates were higher among men compared with women, Blacks compared with Whites, and among non-Hispanics compared with Hispanics. Between 2004 and 2016, age-adjusted incidence rates (per 100,000) increased by a third from 1.8 to 2.4.(4)

Cancer of the small intestine contributed to 9,000 hospital discharges in 2018 (Table 248). Small intestinal cancer hospitalization was rare among children and adolescents and hospital discharge rates increased with age throughout adulthood. Age-adjusted hospital discharge rates (all-listed diagnoses) were higher among men compared with women and Blacks compared with Whites but did not differ by ethnicity. Between 2004 and 2018, age-adjusted hospital discharge rates (per 100,000) with an all-listed diagnosis decreased by a third from 3 to 2.(4)

Cancer of the small intestine contributed to 2,000 deaths in 2019 (Table 249). Small intestinal cancer mortality was rare among children and adolescents and mortality rates increased with age throughout adulthood. Age-adjusted mortality rates (underlying or other cause) were higher among men compared with women, Blacks compared with Whites, and non-Hispanics compared with Hispanics. Between 2004 and 2018, age-adjusted hospital discharge rates (per 100,000) with an all-listed diagnosis increased by a fourth from 0.4 to 0.5.(4)

Colorectal cancer accounted for 132,000 incident cases in 2016 (Table 250). Colorectal cancer was rare among children and incidence rates increased with age throughout adulthood. Age-adjusted incidence rates were higher among men compared with women, Blacks compared with Whites, and non-Hispanics compared with Hispanics. Between 2004 and 2016, age-adjusted incidence rates (per 100,000) decreased by 26% from 47.5 to 35.0 (4)

Colorectal cancer contributed to 250,000 hospital discharges in 2018 (Table 251). Colorectal cancer hospitalization was rare among children and hospital discharge rates increased with age throughout adulthood. Age-adjusted hospital discharge rates (all-listed diagnoses) were higher among men compared with women, Blacks compared with Whites, and non-Hispanics compared with Hispanics. Between 2004 and 2018, age-adjusted hospital discharge rates (per 100,000) with an all-listed diagnosis decreased by 26% from 87 to 64.(4,6)

Colorectal cancer contributed to 60,000 deaths in 2019 (Table 252). Colorectal cancer mortality was rare among children and mortality rates increased with age throughout adulthood. Age-adjusted mortality rates (underlying or other cause) were higher among men compared with women, Blacks compared with Whites, and non-Hispanics compared with Hispanics. Between 2004 and 2019, age-adjusted mortality rates (per 100,000) with colorectal cancer as underlying or other cause decreased by 31% from 21.4 to 14.7.(4)

Primary liver cancer accounted for 26,000 incident cases in 2016 (Table 253). Primary liver cancer was uncommon in childhood through young adulthood and incidence rates peaked among persons 65–74 years. Age-adjusted incidence rates were higher among men compared with women, Blacks compared with Whites, and Hispanics compared with non-Hispanics.

Primary liver cancer contributed to 65,000 hospital discharges in 2018 (Table 254). Primary liver cancer hospitalization was uncommon in childhood through young adulthood and rates peaked among persons 65–74 years. Age-adjusted hospital discharge rates (all-listed diagnoses) were higher among men compared with women, Blacks compared with Whites, and Hispanics compared with non-Hispanics.

Primary liver cancer contributed to 23,000 deaths in 2019 (Table 255). Primary liver cancer mortality rates increased with age. Age-adjusted mortality rates (underlying or other cause) were higher among men compared with women, Blacks compared with Whites, and Hispanics compared with non-Hispanics.

Bile duct cancer accounted for 13,000 incident cases in 2016 (Table 256). Bile duct cancer was rare among children and adolescents and incidence rates increased with age throughout adulthood. Age-adjusted mortality rates (underlying or other cause) were higher among men compared with women, Whites compared with Blacks, and Hispanics compared with non-Hispanics. Between 2004 and 2016, age-adjusted incidence rates (per 100,000) increased by 43% from 2.3 to 3.3.(4)

Bile duct cancer contributed to 34,000 hospital discharges in 2018 (Table 257). Bile duct cancer hospitalization was rare among children and adolescents and hospital discharge rates increased with age throughout adulthood. Age-adjusted hospital discharge rates (all-listed diagnoses) were higher among men compared with women and Hispanics compared with non-Hispanics but did not differ by race. Between 2004 and 2018, age-adjusted hospital discharge rates (per 100,000) with an all-listed diagnosis increased by a third from 6 to 8.(4)

Bile duct cancer contributed to 11,000 deaths in 2019 (Table 258). Bile duct cancer mortality was rare among children and adolescents and mortality rates increased with age throughout adulthood. Age-adjusted mortality rates (underlying or other cause) were higher among men compared with women, Whites compared with Blacks, and non-Hispanics compared with Hispanics. Between 2004 and 2019, age-adjusted mortality rates (per 100,000) with bile duct cancer as underlying or other cause increased by 44% from 1.8 to 2.6.(4)

Gallbladder cancer accounted for an estimated 4,000 incident cases in 2016 (Table 259). Gallbladder cancer was rare among children, adolescents, and younger adults and incidence rates increased with age throughout middle and older age. In contrast to all other common digestive cancers, age-adjusted incidence rates were higher among women compared with men. Incidence rates were higher among Blacks compared with Whites and among Hispanics compared with non-Hispanics. Since 2004, age-adjusted rates (per 100,000) for incidence have remained stable at 1.1.(4)

Gallbladder cancer contributed to 8,000 hospital discharges in 2018 (Table 260). Gallbladder cancer hospitalization was rare among children, adolescents, and younger adults and hospital discharge rates increased with age throughout middle and older age. In contrast to all other common digestive cancers, age-adjusted hospital discharge rates (all-listed diagnoses) were higher among women compared with men. Hospital discharge rates were higher among Blacks compared with Whites and among Hispanics compared with non-Hispanics. Since 2004, age-adjusted hospital discharge rates have remained stable at 2.(4)

Gallbladder cancer contributed to 2,000 deaths in 2019 (Table 261). Gallbladder cancer mortality was rare among children, adolescents, and younger adults and mortality rates increased with age throughout middle and older age. In contrast to all other common digestive cancers, age-adjusted mortality rates (underlying or other cause) were higher among women compared with men. Mortality rates were higher among Blacks compared with Whites and among Hispanics compared with non-Hispanics. Between 2004 and 2019, age-adjusted mortality rates (per 100,000) with gallbladder cancer as underlying or other cause decreased by 14% from 0.7 to 0.6.(4)

Pancreatic cancer accounted for 52,000 incident cases in 2016 (Table 262). Pancreatic cancer was rare among children and adolescents and incidence rates increased with age throughout adulthood. Age-adjusted incidence rates were higher among men compared with women, Blacks compared with Whites, and non-Hispanics compared with Hispanics. Between 2004 and 2016, age-adjusted incidence rates (per 100,000) increased by 18% from 11.3 to 13.3 (4)

Pancreatic cancer contributed to 110,000 hospital discharges in 2018 (Table 263). Pancreatic cancer hospitalization was rare among children and adolescents and hospital discharge rates increased with age throughout adulthood. Age-adjusted hospital discharge rates (all-listed diagnoses) were higher among men compared with women, Blacks compared with Whites, and non-Hispanics compared with Hispanics. Between 2004 and 2018, age-adjusted hospital discharge rates (per 100,000) with an all-listed diagnosis increased by 17% from 23 to 27.(4)

Pancreatic cancer contributed to 48,000 deaths in 2019 (Table 264). Pancreatic cancer mortality was rare among children and adolescents and mortality rates increased with age throughout adulthood. Age-adjusted mortality rates (underlying or other cause) were higher among men compared with women, Blacks compared with Whites, and non-Hispanics compared with Hispanics. Between 2004 and 2019, age-adjusted mortality rates (per 100,000) with pancreatic cancer as underlying or other cause increased by 3% from 11.3 to 11.6.(4)

## Supplementary Material

Supplement 1

Supplement 2

Supplement 3

## Figures and Tables

**Table 1:** All Digestive Diseases: Ambulatory care visits with first-listed and all-listed diagnoses by age, race, ethnicity, and sex in the United States, 2015

Demographic Characteristics		First-Listed Diagnosis		All-Listed Diagnoses	
		Number in thousands	Rate per 100,000	Number in thousands	Rate per 100,000
**Age (years)**	**0 to 11**	8,190	16,838	13,617	27,996
	**12 to 24**	6,828	12,164	10,282	18,315
	**25 to 44**	13,571	16,063	21,969	26,003
	**45 to 54**	11,154	25,885	21,019	48,779
	**55 to 64**	10,279	25,190	22,758	55,772
	**65 to 74**	9,228	33,522	19,760	71,784
	**75 plus**	7,142	35,343	16,774	83,009
**Race**	**White**	52,719	20,005	99,203	36,526
	**Black**	10,033	22,585	18,614	42,558
	**Other**	3,640	15,718	8,362	36,790
**Ethnicity**	**Hispanic**	12,548	25,066	20,816	43,967
	**Not Hispanic**	53,844	19,278	105,363	36,594
**Sex**	**Female**	39,367	23,164	75,610	43,286
	**Male**	27,025	16,734	50,570	30,939
**Total**		66,392	19,962	126,180	37,173

Source: National Ambulatory Medical Care Survey (3-year average, 2014–2016).

Rates for age groups are unadjusted and all other rates are age-adjusted.

**Table 2:** All Digestive Diseases: Emergency department visits with first-listed and all-listed diagnoses by age and sex in the United States, 2018

Demographic Characteristics		First-Listed Diagnosis		All-Listed Diagnoses	
		Number in thousands	Rate per 100,000	Number in thousands	Rate per 100,000
**Age (years)**	**0 to 11**	2,540	5,248	3,966	8,195
	**12 to 24**	2,927	5,277	4,803	8,660
	**25 to 44**	5,253	6,040	9,985	11,480
	**45 to 54**	2,297	5,518	5,205	12,503
	**55 to 64**	2,191	5,183	5,761	13,627
	**65 to 74**	1,695	5,558	5,058	16,587
	**75 plus**	1,834	8,361	6,354	28,961
**Sex**	**Female**	10,976	6,592	24,423	14,027
	**Male**	7,760	4,809	16,706	10,109
**Total**		18,736	5,697	41,131	12,064

Source: Healthcare Cost and Utilization Project Nationwide Emergency Department Sample.

Rates for age groups are unadjusted and all other rates are age-adjusted.

**Table 3:** All Digestive Diseases: Hospital discharges with first-listed and all-listed diagnoses by age, race, ethnicity, and sex in the United States, 2018

Demographic Characteristics		First-Listed Diagnosis		All-Listed Diagnoses	
		Number in thousands	Rate per 100,000	Number in thousands	Rate per 100,000
**Age (years)**	**0 to 11**	154	318	1,185	2,449
	**12 to 24**	187	338	549	990
	**25 to 44**	674	775	2,193	2,521
	**45 to 54**	559	1,343	1,874	4,500
	**55 to 64**	774	1,830	2,995	7,085
	**65 to 74**	760	2,493	3,328	10,914
	**75 plus**	885	4,033	4,327	19,724
**Race**	**White**	3,233	1,102	13,188	4,407
	**Black**	531	1,205	2,331	5,280
	**Other**	267	1,044	1,029	4,121
**Ethnicity**	**Hispanic**	541	1,099	1,831	3,810
	**Not Hispanic**	3,490	1,111	14,716	4,628
**Sex**	**Female**	2,135	1,116	8,920	4,603
	**Male**	1,857	1,082	7,529	4,369
**Total**		3,993	1,098	16,450	4,478

Source: Healthcare Cost and Utilization Project National Inpatient Sample.

Rates for age groups are unadjusted and all other rates are age-adjusted.

**Table 4:** All Digestive Diseases: Deaths with underlying or underlying/other cause and lifetime years of life lost by age, race, ethnicity, and sex in the United States, 2019

Demographic Characteristics		Underlying Cause			Underlying/Other Cause		
		Number of Deaths	Rate per 100,000	Years of potential life lost in thousands	Number of Deaths	Rate per 100,000	Years of potential life lost in thousands
**Age (years)**	**0 to 11**	1,100	2.3	84.4	2,008	4.2	154.0
	**12 to 24**	434	0.8	25.4	1,091	2.0	64.0
	**25 to 44**	11,304	12.9	476.8	17,344	19.8	737.3
	**45 to 54**	24,968	61.1	769.7	35,111	85.9	1,084.0
	**55 to 64**	62,071	146.2	1,413.4	88,896	209.4	2,027.1
	**65 to 74**	77,372	245.8	1,221.8	116,459	369.9	1,835.6
	**75 plus**	117,207	519.2	871.6	211,447	936.6	1,506.9
**Race**	**White**	247,051	73.1	4,022.4	398,253	117.7	6,121.7
	**Black**	34,052	77.6	596.5	53,689	124.1	929.4
	**Other**	13,353	53.0	244.3	20,414	81.9	357.8
**Ethnicity**	**Hispanic**	24,799	58.9	530.5	36,812	89.0	768.8
	**Not Hispanic**	269,657	74.1	4,332.7	435,544	119.6	6,640.1
**Sex**	**Female**	130,904	58.3	2,125.7	218,140	96.3	3,366.3
	**Male**	163,552	88.7	2,737.5	254,216	140.0	4,042.5
**Total**		294,456	72.4	4,863.2	472,356	116.3	7,408.8

Source: Vital Statistics of the United States.

Rates for age groups are unadjusted and all other rates are age-adjusted.

Years of potential life lost is based on U.S. life Table 2006–2018 estimates of life expectancy at age of death.

**Table 5:** All Digestive Diseases: Claims-based prevalence with first-listed and all-listed diagnoses by age, race-ethnicity, sex and region among privately insured enrollees, 2020

Demographic Characteristics		Number of enrollees	First-Listed Diagnosis		All-Listed Diagnoses	
			Number of enrollees with disease	Percent of enrollees with disease	Number of enrollees with disease	Percent of enrollees with disease
**Age (years)**	**0 to 11**	1,101,978	93,661	8.5%	172,707	15.7%
	**12 to 24**	1,451,892	111,327	7.7%	176,361	12.1%
	**25 to 44**	2,656,672	284,972	10.7%	492,626	18.5%
	**45 to 54**	1,452,320	248,092	17.1%	464,662	32.0%
	**55 to 64**	1,640,216	341,348	20.8%	654,682	39.9%
	**65 to 74**	2,840,615	631,047	22.2%	1,188,635	41.8%
	**75 plus**	2,576,241	504,913	19.6%	1,030,999	40.0%
**Race-ethnicity**	**White**	8,657,702	1,449,645	16.7%	2,737,997	31.6%
	**Black**	1,242,477	228,501	18.4%	433,613	34.9%
	**Hispanic**	1,493,457	239,333	16.0%	446,788	29.9%
	**Asian**	607,284	74,540	12.3%	140,459	23.1%
	**Unknown**	1,719,014	223,341	13.0%	421,815	24.5%
**Sex**	**Female**	7,199,363	1,262,266	17.5%	2,396,094	33.3%
	**Male**	6,520,571	953,094	14.6%	1,784,578	27.4%
**Region**	**Northeast**	1,526,275	277,389	18.2%	500,914	32.8%
	**Midwest**	3,322,846	494,252	14.9%	942,800	28.4%
	**South**	5,619,435	985,884	17.5%	1,868,071	33.2%
	**West**	3,251,378	457,835	14.1%	868,887	26.7%
**Total**		13,719,934	2,215,360	16.1%	4,180,672	30.5%

Source: De-identified Optum Clinformatic ^®^ Data Mart.

Enrollees with full enrollment in a commercial health plan or Medicare during the year.

**Table 6:** All Digestive Diseases: Ambulatory care visits with first-listed and all-listed diagnoses by age, race-ethnicity, sex and region among privately insured enrollees, 2020

Demographic Characteristics		First-Listed Diagnosis		All-Listed Diagnoses	
		Number of events	Event rate per 100,000	Number of events	Event rate per 100,000
**Age (years)**	**0 to 11**	138,742	12,590	283,215	25,701
	**12 to 24**	194,991	13,430	322,026	22,180
	**25 to 44**	558,515	21,023	1,032,553	38,866
	**45 to 54**	513,234	35,339	1,073,396	73,909
	**55 to 64**	737,616	44,971	1,644,058	100,234
	**65 to 74**	1,380,964	48,615	3,081,539	108,481
	**75 plus**	1,124,690	43,656	2,911,397	113,009
**Race-ethnicity**	**White**	3,045,440	35,176	6,749,160	77,956
	**Black**	491,995	39,598	1,162,933	93,598
	**Hispanic**	521,380	34,911	1,139,865	76,324
	**Asian**	150,771	24,827	322,664	53,132
	**Unknown**	439,166	25,548	973,562	56,635
**Sex**	**Female**	2,657,694	36,916	6,082,612	84,488
	**Male**	1,991,058	30,535	4,265,572	65,417
**Region**	**Northeast**	598,611	39,220	1,289,784	84,505
	**Midwest**	969,127	29,166	2,153,490	64,809
	**South**	2,128,406	37,876	4,802,169	85,456
	**West**	952,608	29,299	2,102,741	64,672
**Total**		4,648,752	33,883	10,348,184	75,424

Source: De-identified Optum Clinformatic ^®^ Data Mart.

Enrollees with full enrollment in a commercial health plan or Medicare during the year.

**Table 7:** All Digestive Diseases: Emergency department visits with first-listed and all-listed diagnoses by age, race-ethnicity, sex and region among privately insured enrollees, 2020

Demographic Characteristics		First-Listed Diagnosis		All-Listed Diagnoses	
		Number of events	Event rate per 100,000	Number of events	Event rate per 100,000
**Age (years)**	**0 to 11**	965	88	1,415	128
	**12 to 24**	3,398	234	5,561	383
	**25 to 44**	18,980	714	32,916	1,239
	**45 to 54**	21,424	1,475	32,401	2,231
	**55 to 64**	42,370	2,583	67,205	4,097
	**65 to 74**	105,255	3,705	172,755	6,082
	**75 plus**	127,163	4,936	214,422	8,323
**Race-ethnicity**	**White**	202,814	2,343	335,012	3,870
	**Black**	47,209	3,800	78,914	6,351
	**Hispanic**	36,174	2,422	57,680	3,862
	**Asian**	6,270	1,032	10,296	1,695
	**Unknown**	27,088	1,576	44,773	2,605
**Sex**	**Female**	191,534	2,660	320,081	4,446
	**Male**	128,021	1,963	206,594	3,168
**Region**	**Northeast**	39,112	2,563	64,396	4,219
	**Midwest**	66,201	1,992	109,225	3,287
	**South**	149,266	2,656	246,329	4,384
	**West**	64,976	1,998	106,725	3,282
**Total**		319,555	2,329	526,675	3,839

Source: De-identified Optum Clinformatic ^®^ Data Mart.

Enrollees with full enrollment in a commercial health plan or Medicare during the year.

**Table 8:** All Digestive Diseases: Hospital discharges with first-listed and all-listed diagnoses by age, race-ethnicity, sex and region among privately insured enrollees, 2020

Demographic Characteristics		First-Listed Diagnosis		All-Listed Diagnoses	
		Number of events	Event rate per 100,000	Number of events	Event rate per 100,000
**Age (years)**	**0 to 11**	1,770	161	22,408	2,033
	**12 to 24**	2,258	156	9,403	648
	**25 to 44**	10,289	387	39,131	1,473
	**45 to 54**	11,494	791	44,452	3,061
	**55 to 64**	22,199	1,353	103,973	6,339
	**65 to 74**	47,950	1,688	233,696	8,227
	**75 plus**	62,345	2,420	335,855	13,037
**Race-ethnicity**	**White**	105,032	1,213	514,339	5,941
	**Black**	19,757	1,590	106,043	8,535
	**Hispanic**	16,777	1,123	74,156	4,965
	**Asian**	3,466	571	15,349	2,527
	**Unknown**	13,273	772	79,031	4,597
**Sex**	**Female**	87,500	1,215	434,432	6,034
	**Male**	70,805	1,086	354,486	5,436
**Region**	**Northeast**	20,735	1,359	101,404	6,644
	**Midwest**	35,011	1,054	176,590	5,314
	**South**	71,551	1,273	366,456	6,521
	**West**	31,008	954	144,468	4,443
**Total**		158,305	1,154	788,918	5,750

Source: De-identified Optum Clinformatic ^®^ Data Mart.

Enrollees with full enrollment in a commercial health plan or Medicare during the year.

**Table 9:** All Digestive Diseases: Claims-based prevalence with first-listed and all-listed diagnoses by age, race, sex and region among fee-for-service, age-eligible Medicare beneficiaries, 2019

Demographic Characteristics		Number of enrollees	First-Listed Diagnosis		All-Listed Diagnoses	
			Number of enrollees with disease	Percent of enrollees with disease	Number of enrollees with disease	Percent of enrollees with disease
**Age (years)**	**65 to 69**	8,066,320	2,262,980	28.1%	4,016,480	49.8%
	**70 to 74**	6,936,940	2,068,880	29.8%	3,674,040	53.0%
	**75 to 79**	4,894,060	1,494,340	30.5%	2,686,880	54.9%
	**80 to 84**	3,335,340	985,700	29.6%	1,847,460	55.4%
	**85+**	3,578,120	992,580	27.7%	2,003,400	56.0%
**Race**	**White**	22,023,800	6,446,940	29.3%	11,797,960	53.6%
	**Black**	1,861,660	527,600	28.3%	964,080	51.8%
	**Other**	2,925,320	829,940	28.4%	1,466,220	50.1%
**Sex**	**Female**	14,975,560	4,537,040	30.3%	8,328,400	55.6%
	**Male**	11,835,220	3,267,440	27.6%	5,899,860	49.9%
**Region**	**Northeast**	4,748,700	1,443,740	30.4%	2,549,620	53.7%
	**Midwest**	6,069,800	1,658,760	27.3%	3,158,060	52.0%
	**South**	10,595,900	3,210,640	30.3%	5,865,440	55.4%
	**West**	5,396,380	1,491,340	27.6%	2,655,140	49.2%
**Total**		26,810,780	7,804,480	29.1%	14,228,260	53.1%

Source: Centers for Medicare & Medicaid Services, 5% Denominator and Claims Files.

Beneficiaries aged 65+ years with continuous and full Parts A and B enrollment and no Health Maintenance Organization enrollment during the year.

Unweighted counts were multiplied by 20 to produce counts for this table.

**Table 10:** All Digestive Diseases: Ambulatory care visits with first-listed and all-listed diagnoses by age, race, sex and region among fee-for-service, age-eligible Medicare beneficiaries, 2019

Demographic Characteristics		First-Listed Diagnosis		All-Listed Diagnoses	
		Number of events	Event rate per 100,000	Number of events	Event rate per 100,000
**Age (years)**	**65 to 69**	5,348,520	66,307	11,581,900	143,583
	**70 to 74**	4,978,480	71,768	11,005,700	158,654
	**75 to 79**	3,683,640	75,268	8,447,660	172,610
	**80 to 84**	2,386,820	71,562	5,910,200	177,199
	**85+**	2,133,980	59,640	6,275,240	175,378
**Race**	**White**	15,277,320	69,367	35,437,700	160,906
	**Black**	1,208,800	64,931	3,083,400	165,626
	**Other**	2,045,320	69,918	4,699,600	160,653
**Sex**	**Female**	10,749,960	71,783	25,908,960	173,008
	**Male**	7,781,480	65,749	17,311,740	146,273
**Region**	**Northeast**	3,573,040	75,242	8,092,200	170,409
	**Midwest**	3,681,060	60,645	8,847,180	145,757
	**South**	7,763,360	73,268	18,370,940	173,378
	**West**	3,513,980	65,117	7,910,380	146,587
**Total**		18,531,440	69,119	43,220,700	161,206

Source: Centers for Medicare & Medicaid Services, 5% Denominator and Claims Files.

Beneficiaries aged 65+ years with continuous and full Parts A and B enrollment and no Health Maintenance Organization enrollment during the year.

Unweighted counts were multiplied by 20 to produce counts for this table.

**Table 11:** All Digestive Diseases: Emergency department visits with first-listed and all-listed diagnoses by age, race, sex and region among fee-for-service, age-eligible Medicare beneficiaries, 2019

Demographic Characteristics		First-Listed Diagnosis		All-Listed Diagnoses	
		Number of events	Event rate per 100,000	Number of events	Event rate per 100,000
**Age (years)**	**65 to 69**	562,200	6,970	1,476,080	18,299
	**70 to 74**	541,660	7,808	1,502,700	21,662
	**75 to 79**	471,960	9,644	1,374,280	28,081
	**80 to 84**	387,840	11,628	1,184,360	35,509
	**85+**	485,400	13,566	1,596,120	44,608
**Race**	**White**	1,937,860	8,799	5,768,000	26,190
	**Black**	244,400	13,128	687,380	36,923
	**Other**	266,800	9,120	678,160	23,182
**Sex**	**Female**	1,444,540	9,646	4,211,620	28,123
	**Male**	1,004,520	8,488	2,921,920	24,688
**Region**	**Northeast**	420,500	8,855	1,280,580	26,967
	**Midwest**	542,140	8,932	1,623,980	26,755
	**South**	1,015,940	9,588	3,012,260	28,429
	**West**	470,480	8,718	1,216,720	22,547
**Total**		2,449,060	9,135	7,133,540	26,607

Source: Centers for Medicare & Medicaid Services, 5% Denominator and Claims Files.

Beneficiaries aged 65+ years with continuous and full Parts A and B enrollment and no Health Maintenance Organization enrollment during the year.

Unweighted counts were multiplied by 20 to produce counts for this table.

**Table 12:** All Digestive Diseases: Hospital discharges with first-listed and all-listed diagnoses by age, race, sex and region among fee-for-service, age-eligible Medicare beneficiaries, 2019

Demographic Characteristics		First-Listed Diagnosis		All-Listed Diagnoses	
		Number of events	Event rate per 100,000	Number of events	Event rate per 100,000
**Age (years)**	**65 to 69**	202,620	2,512	934,600	11,586
	**70 to 74**	203,160	2,929	993,820	14,326
	**75 to 79**	179,100	3,660	919,520	18,788
	**80 to 84**	147,660	4,427	781,660	23,436
	**85+**	182,500	5,100	1,037,280	28,990
**Race**	**White**	740,160	3,361	3,839,000	17,431
	**Black**	82,120	4,411	407,000	21,862
	**Other**	92,760	3,171	420,880	14,387
**Sex**	**Female**	518,640	3,463	2,634,420	17,591
	**Male**	396,400	3,349	2,032,460	17,173
**Region**	**Northeast**	169,840	3,577	857,580	18,059
	**Midwest**	212,360	3,499	1,097,960	18,089
	**South**	373,640	3,526	1,952,780	18,430
	**West**	159,200	2,950	758,560	14,057
**Total**		915,040	3,413	4,666,880	17,407

Source: Centers for Medicare & Medicaid Services, 5% Denominator and Claims Files.

Beneficiaries aged 65+ years with continuous and full Parts A and B enrollment and no Health Maintenance Organization enrollment during the year.

Unweighted counts were multiplied by 20 to produce counts for this table.

**Table 13:** Gastroesophageal Reflux Disease: Ambulatory care visits with first-listed and all-listed diagnoses by age, race, ethnicity, and sex in the United States, 2015

Demographic Characteristics		First-Listed Diagnosis		All-Listed Diagnoses	
		Number in thousands	Rate per 100,000	Number in thousands	Rate per 100,000
**Age (years)**	**0 to 11**	362	745	1,195	2,457
	**12 to 24**	541	964	1,146	2,042
	**25 to 44**	1,065	1,260	3,463	4,098
	**45 to 54**	952	2,210	4,592	10,657
	**55 to 64**	985	2,414	5,635	13,810
	**65 to 74**	974	3,539	5,945	21,597
	**75 plus**	562	2,780	4,735	23,432
**Race**	**White**	4,387	1,638	21,392	7,488
	**Black**	797	1,835	3,561	8,328
	**Other**	257	1,087	1,759	7,906
**Ethnicity**	**Hispanic**	912	2,232	4,242	10,206
	**Not Hispanic**	4,529	1,568	22,469	7,340
**Sex**	**Female**	3,572	2,009	16,082	8,673
	**Male**	1,869	1,175	10,629	6,421
**Total**		5,441	1,609	26,711	7,578

Source: National Ambulatory Medical Care Survey (3-year average, 2014–2016).

Rates for age groups are unadjusted and all other rates are age-adjusted.

**Table 14:** Gastroesophageal Reflux Disease: Emergency department visits with first-listed and all-listed diagnoses by age and sex in the United States, 2018

Demographic Characteristics		First-Listed Diagnosis		All-Listed Diagnoses	
		Number in thousands	Rate per 100,000	Number in thousands	Rate per 100,000
**Age (years)**	**0 to 11**	44	91	226	466
	**12 to 24**	60	109	326	589
	**25 to 44**	123	141	1,447	1,663
	**45 to 54**	61	147	1,335	3,208
	**55 to 64**	58	138	1,791	4,236
	**65 to 74**	45	147	1,841	6,037
	**75 plus**	46	211	2,518	11,478
**Sex**	**Female**	245	144	5,657	2,913
	**Male**	192	118	3,826	2,228
**Total**		438	131	9,484	2,582

Source: Healthcare Cost and Utilization Project Nationwide Emergency Department Sample.

Rates for age groups are unadjusted and all other rates are age-adjusted.

**Table 15:** Gastroesophageal Reflux Disease: Hospital discharges with first-listed and all-listed diagnoses by age, race, ethnicity, and sex in the United States, 2018

Demographic Characteristics		First-Listed Diagnosis		All-Listed Diagnoses	
		Number in thousands	Rate per 100,000	Number in thousands	Rate per 100,000
**Age (years)**	**0 to 11**	6	12	78	162
	**12 to 24**	2	4	104	188
	**25 to 44**	10	12	628	722
	**45 to 54**	11	27	704	1,691
	**55 to 64**	17	40	1,233	2,917
	**65 to 74**	16	52	1,510	4,953
	**75 plus**	20	90	1,967	8,967
**Race**	**White**	65	22	5,207	1,653
	**Black**	13	30	800	1,841
	**Other**	5	20	274	1,112
**Ethnicity**	**Hispanic**	10	21	476	1,103
	**Not Hispanic**	73	23	5,806	1,726
**Sex**	**Female**	41	21	3,599	1,759
	**Male**	41	24	2,626	1,489
**Total**		82	22	6,225	1,628

Source: Healthcare Cost and Utilization Project National Inpatient Sample.

Rates for age groups are unadjusted and all other rates are age-adjusted.

**Table 16:** Gastroesophageal Reflux Disease: Deaths with underlying or underlying/other cause and lifetime years of life lost by age, race, ethnicity, and sex in the United States, 2019

Demographic Characteristics		Underlying Cause			Underlying/Other Cause		
		Number of Deaths	Rate per 100,000	Years of potential life lost in thousands	Number of Deaths	Rate per 100,000	Years of potential life lost in thousands
**Age (years)**	**0 to 11**	10	0.0	0.8	43	0.1	3.2
	**12 to 24**	2	0.0	0.1	27	0.0	1.6
	**25 to 44**	33	0.0	1.4	194	0.2	8.2
	**45 to 54**	45	0.1	1.4	449	1.1	13.8
	**55 to 64**	117	0.3	2.7	1,269	3.0	28.8
	**65 to 74**	194	0.6	3.0	2,216	7.0	34.4
	**75 plus**	824	3.7	4.8	7,515	33.3	47.8
**Race**	**White**	1,107	0.3	12.3	10,412	3.0	118.8
	**Black**	81	0.2	1.3	1,008	2.5	14.9
	**Other**	37	0.2	0.6	293	1.2	4.1
**Ethnicity**	**Hispanic**	64	0.2	1.2	524	1.4	8.5
	**Not Hispanic**	1,161	0.3	13.0	11,189	3.0	129.3
**Sex**	**Female**	603	0.3	6.7	6,312	2.7	70.0
	**Male**	622	0.4	7.5	5,401	3.1	67.9
**Total**		1,225	0.3	14.2	11,713	2.9	137.9

Source: Vital Statistics of the United States.

Rates for age groups are unadjusted and all other rates are age-adjusted.

Years of potential life lost is based on U.S. life Table 2006–2018 estimates of life expectancy at age of death.

**Table 17:** Gastroesophageal Reflux Disease: Claims-based prevalence with first-listed and all-listed diagnoses by age, race-ethnicity, sex and region among privately insured enrollees, 2020

Demographic Characteristics		Number of enrollees	First-Listed Diagnosis		All-Listed Diagnoses	
			Number of enrollees with disease	Percent of enrollees with disease	Number of enrollees with disease	Percent of enrollees with disease
**Age (years)**	**0 to 11**	1,101,978	9,567	0.9%	20,842	1.9%
	**12 to 24**	1,451,892	10,511	0.7%	25,914	1.8%
	**25 to 44**	2,656,672	43,031	1.6%	132,690	5.0%
	**45 to 54**	1,452,320	36,937	2.5%	145,474	10.0%
	**55 to 64**	1,640,216	52,413	3.2%	241,952	14.8%
	**65 to 74**	2,840,615	103,549	3.6%	492,699	17.3%
	**75 plus**	2,576,241	80,437	3.1%	470,137	18.2%
**Race-ethnicity**	**White**	8,657,702	219,262	2.5%	1,039,266	12.0%
	**Black**	1,242,477	37,054	3.0%	177,232	14.3%
	**Hispanic**	1,493,457	37,219	2.5%	150,528	10.1%
	**Asian**	607,284	10,905	1.8%	40,131	6.6%
	**Unknown**	1,719,014	32,005	1.9%	122,551	7.1%
**Sex**	**Female**	7,199,363	204,551	2.8%	912,394	12.7%
	**Male**	6,520,571	131,894	2.0%	617,314	9.5%
**Region**	**Northeast**	1,526,275	44,716	2.9%	181,214	11.9%
	**Midwest**	3,322,846	65,408	2.0%	339,807	10.2%
	**South**	5,619,435	165,939	3.0%	738,811	13.1%
	**West**	3,251,378	60,382	1.9%	269,876	8.3%
**Total**		13,719,934	336,445	2.5%	1,529,708	11.1%

Source: De-identified Optum Clinformatic ^®^ Data Mart.

Enrollees with full enrollment in a commercial health plan or Medicare during the year.

**Table 18:** Gastroesophageal Reflux Disease: Ambulatory care visits with first-listed and all-listed diagnoses by age, race-ethnicity, sex and region among privately insured enrollees, 2020

Demographic Characteristics		First-Listed Diagnosis		All-Listed Diagnoses	
		Number of events	Event rate per 100,000	Number of events	Event rate per 100,000
**Age (years)**	**0 to 11**	14,041	1,274	39,452	3,580
	**12 to 24**	14,641	1,008	40,683	2,802
	**25 to 44**	60,273	2,269	228,478	8,600
	**45 to 54**	50,487	3,476	268,369	18,479
	**55 to 64**	71,490	4,359	460,324	28,065
	**65 to 74**	141,710	4,989	928,025	32,670
	**75 plus**	110,129	4,275	932,876	36,211
**Race-ethnicity**	**White**	299,725	3,462	1,928,520	22,275
	**Black**	51,036	4,108	365,372	29,407
	**Hispanic**	51,726	3,464	293,130	19,628
	**Asian**	15,100	2,486	73,076	12,033
	**Unknown**	45,184	2,628	238,109	13,851
**Sex**	**Female**	282,690	3,927	1,783,786	24,777
	**Male**	180,081	2,762	1,114,421	17,091
**Region**	**Northeast**	63,293	4,147	345,225	22,619
	**Midwest**	86,607	2,606	598,397	18,009
	**South**	230,753	4,106	1,467,869	26,121
	**West**	82,118	2,526	486,716	14,970
**Total**		462,771	3,373	2,898,207	21,124

Source: De-identified Optum Clinformatic ^®^ Data Mart.

Enrollees with full enrollment in a commercial health plan or Medicare during the year.

**Table 19:** Gastroesophageal Reflux Disease: Emergency department visits with first-listed and all-listed diagnoses by age, race-ethnicity, sex and region among privately insured enrollees, 2020

Demographic Characteristics		First-Listed Diagnosis		All-Listed Diagnoses	
		Number of events	Event rate per 100,000	Number of events	Event rate per 100,000
**Age (years)**	**0 to 11**	32	3	73	7
	**12 to 24**	139	10	371	26
	**25 to 44**	688	26	2,394	90
	**45 to 54**	536	37	2,071	143
	**55 to 64**	907	55	3,921	239
	**65 to 74**	1,776	63	7,832	276
	**75 plus**	1,882	73	8,846	343
**Race-ethnicity**	**White**	3,595	42	16,654	192
	**Black**	1,051	85	4,064	327
	**Hispanic**	662	44	2,141	143
	**Asian**	105	17	467	77
	**Unknown**	547	32	2,182	127
**Sex**	**Female**	3,576	50	15,940	221
	**Male**	2,384	37	9,568	147
**Region**	**Northeast**	743	49	3,910	256
	**Midwest**	1,249	38	6,771	204
	**South**	3,055	54	11,272	201
	**West**	913	28	3,555	109
**Total**		5,960	43	25,508	186

Source: De-identified Optum Clinformatic ^®^ Data Mart.

Enrollees with full enrollment in a commercial health plan or Medicare during the year.

**Table 20:** Gastroesophageal Reflux Disease: Hospital discharges with first-listed and all-listed diagnoses by age, race-ethnicity, sex and region among privately insured enrollees, 2020

Demographic Characteristics		First-Listed Diagnosis		All-Listed Diagnoses	
		Number of events	Event rate per 100,000	Number of events	Event rate per 100,000
**Age (years)**	**0 to 11**	55	5	933	85
	**12 to 24**	25	2	1,570	108
	**25 to 44**	108	4	11,259	424
	**45 to 54**	143	10	17,165	1,182
	**55 to 64**	405	25	44,429	2,709
	**65 to 74**	750	26	99,583	3,506
	**75 plus**	1,130	44	143,001	5,551
**Race-ethnicity**	**White**	1,709	20	221,785	2,562
	**Black**	385	31	43,846	3,529
	**Hispanic**	247	17	24,559	1,644
	**Asian**	42	7	4,729	779
	**Unknown**	233	14	23,021	1,339
**Sex**	**Female**	1,390	19	185,026	2,570
	**Male**	1,226	19	132,914	2,038
**Region**	**Northeast**	312	20	39,742	2,604
	**Midwest**	554	17	75,875	2,283
	**South**	1,185	21	152,116	2,707
	**West**	565	17	50,207	1,544
**Total**		2,616	19	317,940	2,317

Source: De-identified Optum Clinformatic ^®^ Data Mart.

Enrollees with full enrollment in a commercial health plan or Medicare during the year.

**Table 21:** Gastroesophageal Reflux Disease: Claims-based prevalence with first-listed and all-listed diagnoses by age, race, sex and region among fee-for-service, age-eligible Medicare beneficiaries, 2019

Demographic Characteristics		Number of enrollees	First-Listed Diagnosis		All-Listed Diagnoses	
			Number of enrollees with disease	Percent of enrollees with disease	Number of enrollees with disease	Percent of enrollees with disease
**Age (years)**	**65 to 69**	8,066,320	392,040	4.9%	1,659,120	20.6%
	**70 to 74**	6,936,940	368,140	5.3%	1,629,540	23.5%
	**75 to 79**	4,894,060	268,220	5.5%	1,273,780	26.0%
	**80 to 84**	3,335,340	174,080	5.2%	893,240	26.8%
	**85+**	3,578,120	155,280	4.3%	940,280	26.3%
**Race**	**White**	22,023,800	1,127,640	5.1%	5,374,440	24.4%
	**Black**	1,861,660	86,000	4.6%	424,540	22.8%
	**Other**	2,925,320	144,120	4.9%	596,980	20.4%
**Sex**	**Female**	14,975,560	862,780	5.8%	3,926,880	26.2%
	**Male**	11,835,220	494,980	4.2%	2,469,080	20.9%
**Region**	**Northeast**	4,748,700	253,520	5.3%	1,099,980	23.2%
	**Midwest**	6,069,800	256,180	4.2%	1,429,680	23.6%
	**South**	10,595,900	614,400	5.8%	2,851,060	26.9%
	**West**	5,396,380	233,660	4.3%	1,015,240	18.8%
**Total**		26,810,780	1,357,760	5.1%	6,395,960	23.9%

Source: Centers for Medicare & Medicaid Services, 5% Denominator and Claims Files.

Beneficiaries aged 65+ years with continuous and full Parts A and B enrollment and no Health Maintenance Organization enrollment during the year.

Unweighted counts were multiplied by 20 to produce counts for this table.

**Table 22:** Gastroesophageal Reflux Disease: Ambulatory care visits with first-listed and all-listed diagnoses by age, race, sex and region among fee-for-service, age-eligible Medicare beneficiaries, 2019

Demographic Characteristics		First-Listed Diagnosis		All-Listed Diagnoses	
		Number of events	Event rate per 100,000	Number of events	Event rate per 100,000
**Age (years)**	**65 to 69**	557,060	6,906	3,213,040	39,833
	**70 to 74**	532,500	7,676	3,213,460	46,324
	**75 to 79**	378,820	7,740	2,518,320	51,457
	**80 to 84**	247,340	7,416	1,801,280	54,006
	**85+**	234,320	6,549	1,979,600	55,325
**Race**	**White**	1,606,080	7,292	10,517,200	47,754
	**Black**	130,000	6,983	930,960	50,007
	**Other**	213,960	7,314	1,277,540	43,672
**Sex**	**Female**	1,259,740	8,412	8,152,640	54,440
	**Male**	690,300	5,833	4,573,060	38,639
**Region**	**Northeast**	371,400	7,821	2,241,740	47,207
	**Midwest**	348,820	5,747	2,533,080	41,733
	**South**	894,920	8,446	6,014,200	56,760
	**West**	334,900	6,206	1,936,680	35,889
**Total**		1,950,040	7,273	12,725,700	47,465

Source: Centers for Medicare & Medicaid Services, 5% Denominator and Claims Files.

Beneficiaries aged 65+ years with continuous and full Parts A and B enrollment and no Health Maintenance Organization enrollment during the year.

Unweighted counts were multiplied by 20 to produce counts for this table.

**Table 23:** Gastroesophageal Reflux Disease: Emergency department visits with first-listed and all-listed diagnoses by age, race, sex and region among fee-for-service, age-eligible Medicare beneficiaries, 2019

Demographic Characteristics		First-Listed Diagnosis		All-Listed Diagnoses	
		Number of events	Event rate per 100,000	Number of events	Event rate per 100,000
**Age (years)**	**65 to 69**	13,720	170	515,880	6,395
	**70 to 74**	12,660	183	562,800	8,113
	**75 to 79**	11,500	235	537,080	10,974
	**80 to 84**	9,880	296	462,380	13,863
	**85+**	11,580	324	623,620	17,429
**Race**	**White**	45,860	208	2,251,100	10,221
	**Black**	7,660	411	247,140	13,275
	**Other**	5,820	199	203,520	6,957
**Sex**	**Female**	35,000	234	1,657,340	11,067
	**Male**	24,340	206	1,044,420	8,825
**Region**	**Northeast**	9,040	190	475,160	10,006
	**Midwest**	14,360	237	653,820	10,772
	**South**	26,040	246	1,200,480	11,330
	**West**	9,900	183	372,300	6,899
**Total**		59,340	221	2,701,760	10,077

Source: Centers for Medicare & Medicaid Services, 5% Denominator and Claims Files.

Beneficiaries aged 65+ years with continuous and full Parts A and B enrollment and no Health Maintenance Organization enrollment during the year.

Unweighted counts were multiplied by 20 to produce counts for this table.

**Table 24:** Gastroesophageal Reflux Disease: Hospital discharges with first-listed and all-listed diagnoses by age, race, sex and region among fee-for-service, age-eligible Medicare beneficiaries, 2019

Demographic Characteristics		First-Listed Diagnosis		All-Listed Diagnoses	
		Number of events	Event rate per 100,000	Number of events	Event rate per 100,000
**Age (years)**	**65 to 69**	3,560	44	431,460	5,349
	**70 to 74**	3,940	57	484,000	6,977
	**75 to 79**	3,580	73	444,260	9,078
	**80 to 84**	3,120	94	362,980	10,883
	**85+**	4,240	118	468,520	13,094
**Race**	**White**	14,700	67	1,859,960	8,445
	**Black**	2,020	109	167,660	9,006
	**Other**	1,720	59	163,600	5,593
**Sex**	**Female**	10,220	68	1,293,460	8,637
	**Male**	8,220	69	897,760	7,585
**Region**	**Northeast**	3,440	72	391,460	8,244
	**Midwest**	4,400	72	539,980	8,896
	**South**	7,200	68	951,340	8,978
	**West**	3,400	63	308,440	5,716
**Total**		18,440	69	2,191,220	8,173

Source: Centers for Medicare & Medicaid Services, 5% Denominator and Claims Files.

Beneficiaries aged 65+ years with continuous and full Parts A and B enrollment and no Health Maintenance Organization enrollment during the year.

Unweighted counts were multiplied by 20 to produce counts for this table.

**Table 25:** Peptic Ulcer Disease: Ambulatory care visits with first-listed and all-listed diagnoses by age, race, ethnicity, and sex in the United States, 2015

Demographic Characteristics		First-Listed Diagnosis		All-Listed Diagnoses	
		Number in thousands	Rate per 100,000	Number in thousands	Rate per 100,000
**Age (years)**	**0 to 11**	.	.	.	.
	**12 to 24**	5	8	32	56
	**25 to 44**	85	101	128	152
	**45 to 54**	43	100	230	533
	**55 to 64**	105	257	236	577
	**65 to 74**	155	563	499	1,812
	**75 plus**	38	189	241	1,191
**Race**	**White**	235	92	965	329
	**Black**	154	319	263	588
	**Other**	42	188	137	548
**Ethnicity**	**Hispanic**	60	157	333	871
	**Not Hispanic**	371	113	1,032	331
**Sex**	**Female**	288	148	779	404
	**Male**	144	83	586	339
**Total**		431	117	1,365	372

Source: National Ambulatory Medical Care Survey (3-year average, 2014–2016).

Rates for age groups are unadjusted and all other rates are age-adjusted.

**Table 26:** Peptic Ulcer Disease: Emergency department visits with first-listed and all-listed diagnoses by age and sex in the United States, 2018

Demographic Characteristics		First-Listed Diagnosis		All-Listed Diagnoses	
		Number in thousands	Rate per 100,000	Number in thousands	Rate per 100,000
**Age (years)**	**0 to 11**	0	1	1	2
	**12 to 24**	6	10	12	22
	**25 to 44**	26	30	65	75
	**45 to 54**	22	53	63	152
	**55 to 64**	33	77	94	222
	**65 to 74**	34	113	100	328
	**75 plus**	47	213	135	615
**Sex**	**Female**	83	42	240	121
	**Male**	84	48	231	132
**Total**		168	45	471	126

Source: Healthcare Cost and Utilization Project Nationwide Emergency Department Sample.

Rates for age groups are unadjusted and all other rates are age-adjusted.

**Table 27:** Peptic Ulcer Disease: Hospital discharges with first-listed and all-listed diagnoses by age, race, ethnicity, and sex in the United States, 2018

Demographic Characteristics		First-Listed Diagnosis		All-Listed Diagnoses	
		Number in thousands	Rate per 100,000	Number in thousands	Rate per 100,000
**Age (years)**	**0 to 11**	0	0	1	2
	**12 to 24**	2	4	6	10
	**25 to 44**	15	17	43	49
	**45 to 54**	18	44	52	124
	**55 to 64**	31	74	90	214
	**65 to 74**	36	117	105	346
	**75 plus**	49	225	142	645
**Race**	**White**	122	39	349	110
	**Black**	20	46	63	145
	**Other**	11	46	31	127
**Ethnicity**	**Hispanic**	13	31	44	105
	**Not Hispanic**	140	41	399	118
**Sex**	**Female**	74	36	217	105
	**Male**	78	44	222	126
**Total**		152	40	439	114

Source: Healthcare Cost and Utilization Project National Inpatient Sample.

Rates for age groups are unadjusted and all other rates are age-adjusted.

**Table 28:** Peptic Ulcer Disease: Deaths with underlying or underlying/other cause and lifetime years of life lost by age, race, ethnicity, and sex in the United States, 2019

Demographic Characteristics		Underlying Cause			Underlying/Other Cause		
		Number of Deaths	Rate per 100,000	Years of potential life lost in thousands	Number of Deaths	Rate per 100,000	Years of potential life lost in thousands
**Age (years)**	**0 to 11**	1	0.0	0.1	2	0.0	0.1
	**12 to 24**	5	0.0	0.3	12	0.0	0.7
	**25 to 44**	122	0.1	5.1	202	0.2	8.5
	**45 to 54**	237	0.6	7.2	437	1.1	13.4
	**55 to 64**	584	1.4	13.4	1,136	2.7	26.0
	**65 to 74**	758	2.4	12.0	1,607	5.1	25.3
	**75 plus**	1,848	8.2	12.9	3,662	16.2	25.7
**Race**	**White**	3,022	0.9	42.4	6,009	1.8	83.1
	**Black**	353	0.8	5.9	677	1.6	11.1
	**Other**	180	0.8	2.7	372	1.5	5.6
**Ethnicity**	**Hispanic**	227	0.6	3.8	482	1.3	8.2
	**Not Hispanic**	3,328	0.9	47.2	6,576	1.8	91.6
**Sex**	**Female**	1,751	0.8	23.8	3,336	1.5	45.5
	**Male**	1,804	1.0	27.2	3,722	2.1	54.3
**Total**		3,555	0.9	51.0	7,058	1.7	99.8

Source: Vital Statistics of the United States.

Rates for age groups are unadjusted and all other rates are age-adjusted.

Years of potential life lost is based on U.S. life Table 2006–2018 estimates of life expectancy at age of death.

**Table 29:** Peptic Ulcer Disease: Claims-based prevalence with first-listed and all-listed diagnoses by age, race-ethnicity, sex and region among privately insured enrollees, 2020

Demographic Characteristics		Number of enrollees	First-Listed Diagnosis		All-Listed Diagnoses	
			Number of enrollees with disease	Percent of enrollees with disease	Number of enrollees with disease	Percent of enrollees with disease
**Age (years)**	**0 to 11**	1,101,978	39	0.0%	105	0.0%
	**12 to 24**	1,451,892	355	0.0%	895	0.1%
	**25 to 44**	2,656,672	1,652	0.1%	4,242	0.2%
	**45 to 54**	1,452,320	1,735	0.1%	5,241	0.4%
	**55 to 64**	1,640,216	3,369	0.2%	10,402	0.6%
	**65 to 74**	2,840,615	7,852	0.3%	23,619	0.8%
	**75 plus**	2,576,241	9,206	0.4%	27,042	1.0%
**Race-ethnicity**	**White**	8,657,702	16,044	0.2%	47,287	0.5%
	**Black**	1,242,477	2,988	0.2%	8,857	0.7%
	**Hispanic**	1,493,457	2,460	0.2%	7,378	0.5%
	**Asian**	607,284	894	0.1%	2,554	0.4%
	**Unknown**	1,719,014	1,822	0.1%	5,470	0.3%
**Sex**	**Female**	7,199,363	13,993	0.2%	40,459	0.6%
	**Male**	6,520,571	10,215	0.2%	31,087	0.5%
**Region**	**Northeast**	1,526,275	3,075	0.2%	9,058	0.6%
	**Midwest**	3,322,846	5,054	0.2%	15,022	0.5%
	**South**	5,619,435	11,073	0.2%	33,199	0.6%
	**West**	3,251,378	5,006	0.2%	14,267	0.4%
**Total**		13,719,934	24,208	0.2%	71,546	0.5%

Source: De-identified Optum Clinformatic ^®^ Data Mart.

Enrollees with full enrollment in a commercial health plan or Medicare during the year.

**Table 30:** Peptic Ulcer Disease: Ambulatory care visits with first-listed and all-listed diagnoses by age, race-ethnicity, sex and region among privately insured enrollees, 2020

Demographic Characteristics		First-Listed Diagnosis		All-Listed Diagnoses	
		Number of events	Event rate per 100,000	Number of events	Event rate per 100,000
**Age (years)**	**0 to 11**	46	4	130	12
	**12 to 24**	444	31	1,210	83
	**25 to 44**	1,962	74	5,714	215
	**45 to 54**	2,090	144	7,129	491
	**55 to 64**	3,817	233	13,880	846
	**65 to 74**	8,754	308	31,724	1,117
	**75 plus**	9,500	369	37,610	1,460
**Race-ethnicity**	**White**	17,436	201	64,734	748
	**Black**	3,433	276	11,898	958
	**Hispanic**	2,804	188	10,039	672
	**Asian**	1,038	171	3,648	601
	**Unknown**	1,902	111	7,078	412
**Sex**	**Female**	16,007	222	57,474	798
	**Male**	10,606	163	39,923	612
**Region**	**Northeast**	3,337	219	12,003	786
	**Midwest**	5,311	160	19,397	584
	**South**	12,513	223	45,431	808
	**West**	5,452	168	20,566	633
**Total**		26,613	194	97,397	710

Source: De-identified Optum Clinformatic ^®^ Data Mart.

Enrollees with full enrollment in a commercial health plan or Medicare during the year.

**Table 31:** Peptic Ulcer Disease: Emergency department visits with first-listed and all-listed diagnoses by age, race-ethnicity, sex and region among privately insured enrollees, 2020

Demographic Characteristics		First-Listed Diagnosis		All-Listed Diagnoses	
		Number of events	Event rate per 100,000	Number of events	Event rate per 100,000
**Age (years)**	**0 to 11**	1	0	3	0
	**12 to 24**	4	0	22	2
	**25 to 44**	50	2	130	5
	**45 to 54**	51	4	157	11
	**55 to 64**	153	9	366	22
	**65 to 74**	381	13	787	28
	**75 plus**	439	17	939	36
**Race-ethnicity**	**White**	768	9	1,629	19
	**Black**	132	11	314	25
	**Hispanic**	79	5	208	14
	**Asian**	21	3	57	9
	**Unknown**	79	5	196	11
**Sex**	**Female**	601	8	1,326	18
	**Male**	478	7	1,078	17
**Region**	**Northeast**	158	10	334	22
	**Midwest**	283	9	539	16
	**South**	433	8	1,039	18
	**West**	205	6	492	15
**Total**		1,079	8	2,404	18

Source: De-identified Optum Clinformatic ^®^ Data Mart.

Enrollees with full enrollment in a commercial health plan or Medicare during the year.

**Table 32:** Peptic Ulcer Disease: Hospital discharges with first-listed and all-listed diagnoses by age, race-ethnicity, sex and region among privately insured enrollees, 2020

Demographic Characteristics		First-Listed Diagnosis		All-Listed Diagnoses	
		Number of events	Event rate per 100,000	Number of events	Event rate per 100,000
**Age (years)**	**0 to 11**	3	0	18	2
	**12 to 24**	24	2	100	7
	**25 to 44**	192	7	694	26
	**45 to 54**	337	23	1,230	85
	**55 to 64**	919	56	3,360	205
	**65 to 74**	2,523	89	8,812	310
	**75 plus**	4,211	163	13,342	518
**Race-ethnicity**	**White**	5,725	66	18,415	213
	**Black**	941	76	3,651	294
	**Hispanic**	710	48	2,621	175
	**Asian**	240	40	722	119
	**Unknown**	593	34	2,147	125
**Sex**	**Female**	4,213	59	14,152	197
	**Male**	3,996	61	13,404	206
**Region**	**Northeast**	978	64	3,431	225
	**Midwest**	1,866	56	5,958	179
	**South**	3,516	63	12,581	224
	**West**	1,849	57	5,586	172
**Total**		8,209	60	27,556	201

Source: De-identified Optum Clinformatic ^®^ Data Mart.

Enrollees with full enrollment in a commercial health plan or Medicare during the year.

**Table 33:** Peptic Ulcer Disease: Claims-based prevalence with first-listed and all-listed diagnoses by age, race, sex and region among fee-for-service, age-eligible Medicare beneficiaries, 2019

Demographic Characteristics		Number of enrollees	First-Listed Diagnosis		All-Listed Diagnoses	
			Number of enrollees with disease	Percent of enrollees with disease	Number of enrollees with disease	Percent of enrollees with disease
**Age (years)**	**65 to 69**	8,066,320	26,060	0.3%	77,660	1.0%
	**70 to 74**	6,936,940	27,640	0.4%	79,920	1.2%
	**75 to 79**	4,894,060	23,420	0.5%	68,360	1.4%
	**80 to 84**	3,335,340	18,240	0.5%	50,660	1.5%
	**85+**	3,578,120	18,260	0.5%	53,480	1.5%
**Race**	**White**	22,023,800	90,720	0.4%	264,140	1.2%
	**Black**	1,861,660	8,340	0.4%	25,460	1.4%
	**Other**	2,925,320	14,560	0.5%	40,480	1.4%
**Sex**	**Female**	14,975,560	65,180	0.4%	188,600	1.3%
	**Male**	11,835,220	48,440	0.4%	141,480	1.2%
**Region**	**Northeast**	4,748,700	19,620	0.4%	56,500	1.2%
	**Midwest**	6,069,800	24,540	0.4%	72,540	1.2%
	**South**	10,595,900	45,840	0.4%	135,980	1.3%
	**West**	5,396,380	23,620	0.4%	65,060	1.2%
**Total**		26,810,780	113,620	0.4%	330,080	1.2%

Source: Centers for Medicare & Medicaid Services, 5% Denominator and Claims Files.

Beneficiaries aged 65+ years with continuous and full Parts A and B enrollment and no Health Maintenance Organization enrollment during the year.

Unweighted counts were multiplied by 20 to produce counts for this table.

**Table 34:** Peptic Ulcer Disease: Ambulatory care visits with first-listed and all-listed diagnoses by age, race, sex and region among fee-for-service, age-eligible Medicare beneficiaries, 2019

Demographic Characteristics		First-Listed Diagnosis		All-Listed Diagnoses	
		Number of events	Event rate per 100,000	Number of events	Event rate per 100,000
**Age (years)**	**65 to 69**	28,320	351	108,400	1,344
	**70 to 74**	30,300	437	116,640	1,681
	**75 to 79**	24,660	504	96,200	1,966
	**80 to 84**	18,900	567	75,600	2,267
	**85+**	17,480	489	74,560	2,084
**Race**	**White**	94,780	430	374,340	1,700
	**Black**	8,280	445	33,320	1,790
	**Other**	16,600	567	63,740	2,179
**Sex**	**Female**	72,060	481	281,780	1,882
	**Male**	47,600	402	189,620	1,602
**Region**	**Northeast**	21,620	455	81,420	1,715
	**Midwest**	24,320	401	96,520	1,590
	**South**	48,400	457	194,020	1,831
	**West**	25,320	469	99,440	1,843
**Total**		119,660	446	471,400	1,758

Source: Centers for Medicare & Medicaid Services, 5% Denominator and Claims Files.

Beneficiaries aged 65+ years with continuous and full Parts A and B enrollment and no Health Maintenance Organization enrollment during the year.

Unweighted counts were multiplied by 20 to produce counts for this table.

**Table 35:** Peptic Ulcer Disease: Emergency department visits with first-listed and all-listed diagnoses by age, race, sex and region among fee-for-service, age-eligible Medicare beneficiaries, 2019

Demographic Characteristics		First-Listed Diagnosis		All-Listed Diagnoses	
		Number of events	Event rate per 100,000	Number of events	Event rate per 100,000
**Age (years)**	**65 to 69**	9,420	117	25,540	317
	**70 to 74**	10,260	148	27,980	403
	**75 to 79**	9,420	192	27,380	559
	**80 to 84**	8,500	255	22,740	682
	**85+**	10,520	294	29,060	812
**Race**	**White**	38,440	175	104,440	474
	**Black**	4,320	232	12,980	697
	**Other**	5,360	183	15,280	522
**Sex**	**Female**	25,880	173	71,940	480
	**Male**	22,240	188	60,760	513
**Region**	**Northeast**	8,160	172	22,860	481
	**Midwest**	10,560	174	29,140	480
	**South**	19,260	182	55,060	520
	**West**	10,140	188	25,640	475
**Total**		48,120	179	132,700	495

Source: Centers for Medicare & Medicaid Services, 5% Denominator and Claims Files.

Beneficiaries aged 65+ years with continuous and full Parts A and B enrollment and no Health Maintenance Organization enrollment during the year.

Unweighted counts were multiplied by 20 to produce counts for this table.

**Table 36:** Peptic Ulcer Disease: Hospital discharges with first-listed and all-listed diagnoses by age, race, sex and region among fee-for-service, age-eligible Medicare beneficiaries, 2019

Demographic Characteristics		First-Listed Diagnosis		All-Listed Diagnoses	
		Number of events	Event rate per 100,000	Number of events	Event rate per 100,000
**Age (years)**	**65 to 69**	9,360	116	26,920	334
	**70 to 74**	10,200	147	29,580	426
	**75 to 79**	9,440	193	29,580	604
	**80 to 84**	8,700	261	24,140	724
	**85+**	10,760	301	30,000	838
**Race**	**White**	39,420	179	111,460	506
	**Black**	3,900	209	12,700	682
	**Other**	5,140	176	16,060	549
**Sex**	**Female**	25,600	171	74,320	496
	**Male**	22,860	193	65,900	557
**Region**	**Northeast**	8,020	169	23,420	493
	**Midwest**	10,980	181	31,920	526
	**South**	19,380	183	57,940	547
	**West**	10,080	187	26,940	499
**Total**		48,460	181	140,220	523

Source: Centers for Medicare & Medicaid Services, 5% Denominator and Claims Files.

Beneficiaries aged 65+ years with continuous and full Parts A and B enrollment and no Health Maintenance Organization enrollment during the year.

Unweighted counts were multiplied by 20 to produce counts for this table.

**Table 37:** Functional Disorders: Ambulatory care visits with first-listed and all-listed diagnoses by age, race, ethnicity, and sex in the United States, 2015

Demographic Characteristics		First-Listed Diagnosis		All-Listed Diagnoses	
		Number in thousands	Rate per 100,000	Number in thousands	Rate per 100,000
**Age (years)**	**0 to 11**	871	1,790	1,815	3,732
	**12 to 24**	444	791	991	1,765
	**25 to 44**	577	683	2,304	2,727
	**45 to 54**	444	1,030	1,921	4,459
	**55 to 64**	299	732	1,770	4,336
	**65 to 74**	612	2,224	1,571	5,707
	**75 plus**	664	3,287	1,862	9,215
**Race**	**White**	2,837	1,128	9,633	3,626
	**Black**	772	1,882	1,833	4,388
	**Other**	301	1,488	768	3,504
**Ethnicity**	**Hispanic**	874	1,923	2,434	5,097
	**Not Hispanic**	3,036	1,125	9,800	3,498
**Sex**	**Female**	2,298	1,407	8,394	4,921
	**Male**	1,613	1,069	3,841	2,479
**Total**		3,910	1,230	12,234	3,707

Source: National Ambulatory Medical Care Survey (3-year average, 2014–2016).

Rates for age groups are unadjusted and all other rates are age-adjusted.

**Table 38:** Functional Disorders: Emergency department visits with first-listed and all-listed diagnoses by age and sex in the United States, 2018

Demographic Characteristics		First-Listed Diagnosis		All-Listed Diagnoses	
		Number in thousands	Rate per 100,000	Number in thousands	Rate per 100,000
**Age (years)**	**0 to 11**	356	736	588	1,214
	**12 to 24**	174	313	416	750
	**25 to 44**	205	236	791	909
	**45 to 54**	108	258	494	1,186
	**55 to 64**	124	292	612	1,448
	**65 to 74**	126	414	614	2,014
	**75 plus**	190	864	952	4,341
**Sex**	**Female**	727	440	2,713	1,522
	**Male**	554	348	1,753	1,062
**Total**		1,282	393	4,467	1,294

Source: Healthcare Cost and Utilization Project Nationwide Emergency Department Sample.

Rates for age groups are unadjusted and all other rates are age-adjusted.

**Table 39:** Functional Disorders: Hospital discharges with first-listed and all-listed diagnoses by age, race, ethnicity, and sex in the United States, 2018

Demographic Characteristics		First-Listed Diagnosis		All-Listed Diagnoses	
		Number in thousands	Rate per 100,000	Number in thousands	Rate per 100,000
**Age (years)**	**0 to 11**	10	22	82	170
	**12 to 24**	9	16	114	205
	**25 to 44**	21	24	356	409
	**45 to 54**	17	40	298	716
	**55 to 64**	23	54	466	1,103
	**65 to 74**	24	77	520	1,704
	**75 plus**	27	125	736	3,354
**Race**	**White**	103	36	2,023	668
	**Black**	20	46	419	957
	**Other**	8	31	150	604
**Ethnicity**	**Hispanic**	15	31	263	571
	**Not Hispanic**	116	38	2,329	725
**Sex**	**Female**	76	41	1,509	771
	**Male**	54	32	1,062	614
**Total**		130	37	2,571	695

Source: Healthcare Cost and Utilization Project National Inpatient Sample.

Rates for age groups are unadjusted and all other rates are age-adjusted.

**Table 40:** Functional Disorders: Deaths with underlying or underlying/other cause and lifetime years of life lost by age, race, ethnicity, and sex in the United States, 2019

Demographic Characteristics		Underlying Cause			Underlying/Other Cause		
		Number of Deaths	Rate per 100,000	Years of potential life lost in thousands	Number of Deaths	Rate per 100,000	Years of potential life lost in thousands
**Age (years)**	**0 to 11**	13	0.0	1.0	39	0.1	2.9
	**12 to 24**	11	0.0	0.6	41	0.1	2.5
	**25 to 44**	43	0.0	1.9	179	0.2	8.0
	**45 to 54**	59	0.1	1.8	252	0.6	7.9
	**55 to 64**	127	0.3	2.9	598	1.4	13.8
	**65 to 74**	207	0.7	3.3	924	2.9	14.6
	**75 plus**	493	2.2	3.3	2,259	10.0	15.3
**Race**	**White**	835	0.2	12.5	3,717	1.1	54.3
	**Black**	99	0.2	2.1	436	1.0	8.3
	**Other**	19	0.1	0.3	139	0.6	2.3
**Ethnicity**	**Hispanic**	48	0.1	1.2	254	0.6	6.0
	**Not Hispanic**	905	0.3	13.7	4,038	1.1	59.0
**Sex**	**Female**	589	0.3	8.6	2,536	1.1	36.8
	**Male**	364	0.2	6.3	1,756	1.0	28.2
**Total**		953	0.2	14.9	4,292	1.1	64.9

Source: Vital Statistics of the United States.

Rates for age groups are unadjusted and all other rates are age-adjusted.

Years of potential life lost is based on U.S. life Table 2006–2018 estimates of life expectancy at age of death.

**Table 41:** Functional Disorders: Claims-based prevalence with first-listed and all-listed diagnoses by age, race-ethnicity, sex and region among privately insured enrollees, 2020

Demographic Characteristics		Number of enrollees	First-Listed Diagnosis		All-Listed Diagnoses	
			Number of enrollees with disease	Percent of enrollees with disease	Number of enrollees with disease	Percent of enrollees with disease
**Age (years)**	**0 to 11**	1,101,978	17,221	1.6%	41,010	3.7%
	**12 to 24**	1,451,892	12,495	0.9%	31,145	2.1%
	**25 to 44**	2,656,672	24,790	0.9%	71,710	2.7%
	**45 to 54**	1,452,320	18,234	1.3%	59,554	4.1%
	**55 to 64**	1,640,216	26,001	1.6%	89,930	5.5%
	**65 to 74**	2,840,615	53,853	1.9%	179,930	6.3%
	**75 plus**	2,576,241	65,723	2.6%	227,352	8.8%
**Race-ethnicity**	**White**	8,657,702	135,735	1.6%	444,685	5.1%
	**Black**	1,242,477	25,978	2.1%	84,337	6.8%
	**Hispanic**	1,493,457	23,554	1.6%	74,925	5.0%
	**Asian**	607,284	6,968	1.1%	21,398	3.5%
	**Unknown**	1,719,014	26,082	1.5%	75,286	4.4%
**Sex**	**Female**	7,199,363	142,418	2.0%	458,945	6.4%
	**Male**	6,520,571	75,899	1.2%	241,686	3.7%
**Region**	**Northeast**	1,526,275	30,308	2.0%	90,664	5.9%
	**Midwest**	3,322,846	43,513	1.3%	146,587	4.4%
	**South**	5,619,435	102,125	1.8%	325,473	5.8%
	**West**	3,251,378	42,371	1.3%	137,907	4.2%
**Total**		13,719,934	218,317	1.6%	700,631	5.1%

Source: De-identified Optum Clinformatic ^®^ Data Mart.

Enrollees with full enrollment in a commercial health plan or Medicare during the year.

**Table 42:** Functional Disorders: Ambulatory care visits with first-listed and all-listed diagnoses by age, race-ethnicity, sex and region among privately insured enrollees, 2020

Demographic Characteristics		First-Listed Diagnosis		All-Listed Diagnoses	
		Number of events	Event rate per 100,000	Number of events	Event rate per 100,000
**Age (years)**	**0 to 11**	22,308	2,024	57,585	5,226
	**12 to 24**	16,865	1,162	45,951	3,165
	**25 to 44**	33,288	1,253	112,940	4,251
	**45 to 54**	24,548	1,690	98,347	6,772
	**55 to 64**	34,877	2,126	148,806	9,072
	**65 to 74**	71,228	2,507	288,154	10,144
	**75 plus**	89,635	3,479	393,339	15,268
**Race-ethnicity**	**White**	182,846	2,112	726,991	8,397
	**Black**	35,038	2,820	141,648	11,400
	**Hispanic**	31,103	2,083	122,070	8,174
	**Asian**	8,907	1,467	33,771	5,561
	**Unknown**	34,855	2,028	120,642	7,018
**Sex**	**Female**	193,110	2,682	775,604	10,773
	**Male**	99,639	1,528	369,518	5,667
**Region**	**Northeast**	42,798	2,804	155,976	10,219
	**Midwest**	56,744	1,708	225,756	6,794
	**South**	136,425	2,428	537,446	9,564
	**West**	56,782	1,746	225,944	6,949
**Total**		292,749	2,134	1,145,122	8,346

Source: De-identified Optum Clinformatic ^®^ Data Mart.

Enrollees with full enrollment in a commercial health plan or Medicare during the year.

**Table 43:** Functional Disorders: Emergency department visits with first-listed and all-listed diagnoses by age, race-ethnicity, sex and region among privately insured enrollees, 2020

Demographic Characteristics		First-Listed Diagnosis		All-Listed Diagnoses	
		Number of events	Event rate per 100,000	Number of events	Event rate per 100,000
**Age (years)**	**0 to 11**	114	10	206	19
	**12 to 24**	131	9	393	27
	**25 to 44**	815	31	2,277	86
	**45 to 54**	1,028	71	2,428	167
	**55 to 64**	2,300	140	4,773	291
	**65 to 74**	6,666	235	13,039	459
	**75 plus**	10,824	420	21,223	824
**Race-ethnicity**	**White**	13,484	156	27,644	319
	**Black**	4,044	325	7,784	626
	**Hispanic**	2,057	138	4,333	290
	**Asian**	425	70	831	137
	**Unknown**	1,868	109	3,747	218
**Sex**	**Female**	12,091	168	25,602	356
	**Male**	9,787	150	18,737	287
**Region**	**Northeast**	3,077	202	5,609	367
	**Midwest**	4,506	136	8,761	264
	**South**	10,174	181	21,045	375
	**West**	4,121	127	8,924	274
**Total**		21,878	159	44,339	323

Source: De-identified Optum Clinformatic ^®^ Data Mart.

Enrollees with full enrollment in a commercial health plan or Medicare during the year.

**Table 44:** Functional Disorders: Hospital discharges with first-listed and all-listed diagnoses by age, race-ethnicity, sex and region among privately insured enrollees, 2020

Demographic Characteristics		First-Listed Diagnosis		All-Listed Diagnoses	
		Number of events	Event rate per 100,000	Number of events	Event rate per 100,000
**Age (years)**	**0 to 11**	81	7	1,222	111
	**12 to 24**	120	8	2,601	179
	**25 to 44**	318	12	6,990	263
	**45 to 54**	387	27	8,714	600
	**55 to 64**	684	42	19,259	1,174
	**65 to 74**	1,219	43	40,825	1,437
	**75 plus**	1,517	59	60,455	2,347
**Race-ethnicity**	**White**	2,894	33	92,269	1,066
	**Black**	553	45	20,808	1,675
	**Hispanic**	405	27	12,692	850
	**Asian**	93	15	2,846	469
	**Unknown**	381	22	11,451	666
**Sex**	**Female**	2,521	35	81,522	1,132
	**Male**	1,805	28	58,544	898
**Region**	**Northeast**	611	40	18,619	1,220
	**Midwest**	919	28	30,462	917
	**South**	1,985	35	66,446	1,182
	**West**	811	25	24,539	755
**Total**		4,326	32	140,066	1,021

Source: De-identified Optum Clinformatic ^®^ Data Mart.

Enrollees with full enrollment in a commercial health plan or Medicare during the year.

**Table 45:** Functional Disorders: Claims-based prevalence with first-listed and all-listed diagnoses by age, race, sex and region among fee-for-service, age-eligible Medicare beneficiaries, 2019

Demographic Characteristics		Number of enrollees	First-Listed Diagnosis		All-Listed Diagnoses	
			Number of enrollees with disease	Percent of enrollees with disease	Number of enrollees with disease	Percent of enrollees with disease
**Age (years)**	**65 to 69**	8,066,320	200,600	2.5%	651,060	8.1%
	**70 to 74**	6,936,940	211,120	3.0%	672,680	9.7%
	**75 to 79**	4,894,060	185,700	3.8%	584,700	11.9%
	**80 to 84**	3,335,340	149,220	4.5%	487,200	14.6%
	**85+**	3,578,120	192,180	5.4%	644,840	18.0%
**Race**	**White**	22,023,800	760,060	3.5%	2,477,760	11.3%
	**Black**	1,861,660	78,480	4.2%	242,680	13.0%
	**Other**	2,925,320	100,280	3.4%	320,040	10.9%
**Sex**	**Female**	14,975,560	609,300	4.1%	1,981,680	13.2%
	**Male**	11,835,220	329,520	2.8%	1,058,800	8.9%
**Region**	**Northeast**	4,748,700	183,080	3.9%	565,260	11.9%
	**Midwest**	6,069,800	185,320	3.1%	648,220	10.7%
	**South**	10,595,900	399,780	3.8%	1,286,480	12.1%
	**West**	5,396,380	170,640	3.2%	540,520	10.0%
**Total**		26,810,780	938,820	3.5%	3,040,480	11.3%

Source: Centers for Medicare & Medicaid Services, 5% Denominator and Claims Files.

Beneficiaries aged 65+ years with continuous and full Parts A and B enrollment and no Health Maintenance Organization enrollment during the year.

Unweighted counts were multiplied by 20 to produce counts for this table.

**Table 46:** Functional Disorders: Ambulatory care visits with first-listed and all-listed diagnoses by age, race, sex and region among fee-for-service, age-eligible Medicare beneficiaries, 2019

Demographic Characteristics		First-Listed Diagnosis		All-Listed Diagnoses	
		Number of events	Event rate per 100,000	Number of events	Event rate per 100,000
**Age (years)**	**65 to 69**	246,240	3,053	1,048,200	12,995
	**70 to 74**	263,460	3,798	1,102,520	15,893
	**75 to 79**	233,340	4,768	979,480	20,014
	**80 to 84**	190,220	5,703	834,440	25,018
	**85+**	250,060	6,989	1,238,160	34,604
**Race**	**White**	962,360	4,370	4,196,640	19,055
	**Black**	94,800	5,092	431,000	23,151
	**Other**	126,160	4,313	575,160	19,661
**Sex**	**Female**	801,320	5,351	3,560,620	23,776
	**Male**	382,000	3,228	1,642,180	13,875
**Region**	**Northeast**	245,840	5,177	1,012,540	21,322
	**Midwest**	221,640	3,652	1,048,280	17,270
	**South**	506,440	4,780	2,235,620	21,099
	**West**	209,400	3,880	906,360	16,796
**Total**		1,183,320	4,414	5,202,800	19,406

Source: Centers for Medicare & Medicaid Services, 5% Denominator and Claims Files.

Beneficiaries aged 65+ years with continuous and full Parts A and B enrollment and no Health Maintenance Organization enrollment during the year.

Unweighted counts were multiplied by 20 to produce counts for this table.

**Table 47:** Functional Disorders: Emergency department visits with first-listed and all-listed diagnoses by age, race, sex and region among fee-for-service, age-eligible Medicare beneficiaries, 2019

Demographic Characteristics		First-Listed Diagnosis		All-Listed Diagnoses	
		Number of events	Event rate per 100,000	Number of events	Event rate per 100,000
**Age (years)**	**65 to 69**	43,220	536	181,220	2,247
	**70 to 74**	44,820	646	191,960	2,767
	**75 to 79**	44,900	917	184,600	3,772
	**80 to 84**	38,840	1,164	169,820	5,092
	**85+**	54,680	1,528	261,520	7,309
**Race**	**White**	173,280	787	789,220	3,583
	**Black**	28,860	1,550	106,400	5,715
	**Other**	24,320	831	93,500	3,196
**Sex**	**Female**	123,240	823	599,640	4,004
	**Male**	103,220	872	389,480	3,291
**Region**	**Northeast**	38,840	818	185,980	3,916
	**Midwest**	49,440	815	219,240	3,612
	**South**	94,520	892	414,480	3,912
	**West**	43,660	809	169,420	3,140
**Total**		226,460	845	989,120	3,689

Source: Centers for Medicare & Medicaid Services, 5% Denominator and Claims Files.

Beneficiaries aged 65+ years with continuous and full Parts A and B enrollment and no Health Maintenance Organization enrollment during the year.

Unweighted counts were multiplied by 20 to produce counts for this table.

**Table 48:** Functional Disorders: Hospital discharges with first-listed and all-listed diagnoses by age, race, sex and region among fee-for-service, age-eligible Medicare beneficiaries, 2019

Demographic Characteristics		First-Listed Diagnosis		All-Listed Diagnoses	
		Number of events	Event rate per 100,000	Number of events	Event rate per 100,000
**Age (years)**	**65 to 69**	7,560	94	159,320	1,975
	**70 to 74**	6,840	99	168,820	2,434
	**75 to 79**	6,080	124	153,340	3,133
	**80 to 84**	4,220	127	137,960	4,136
	**85+**	5,900	165	201,980	5,645
**Race**	**White**	23,540	107	669,340	3,039
	**Black**	3,660	197	77,220	4,148
	**Other**	3,400	116	74,860	2,559
**Sex**	**Female**	16,700	112	489,400	3,268
	**Male**	13,900	117	332,020	2,805
**Region**	**Northeast**	5,960	126	154,940	3,263
	**Midwest**	6,540	108	187,240	3,085
	**South**	12,400	117	348,160	3,286
	**West**	5,700	106	131,080	2,429
**Total**		30,600	114	821,420	3,064

Source: Centers for Medicare & Medicaid Services, 5% Denominator and Claims Files.

Beneficiaries aged 65+ years with continuous and full Parts A and B enrollment and no Health Maintenance Organization enrollment during the year.

Unweighted counts were multiplied by 20 to produce counts for this table.

**Table 49:** Appendicitis: Ambulatory care visits with first-listed and all-listed diagnoses by age, race, ethnicity, and sex in the United States, 2015

Demographic Characteristics		First-Listed Diagnosis		All-Listed Diagnoses	
		Number in thousands	Rate per 100,000	Number in thousands	Rate per 100,000
**Age (years)**	**0 to 11**	35	72	40	82
	**12 to 24**	135	240	155	277
	**25 to 44**	125	148	166	197
	**45 to 54**	65	151	74	172
	**55 to 64**	204	501	223	546
	**65 to 74**	75	274	77	281
	**75 plus**	3	17	3	17
**Race**	**White**	539	194	601	220
	**Black**	39	83	70	152
	**Other**	65	258	67	269
**Ethnicity**	**Hispanic**	230	464	245	490
	**Not Hispanic**	412	151	494	181
**Sex**	**Female**	296	170	325	189
	**Male**	346	195	414	236
**Total**		643	182	739	212

Source: National Ambulatory Medical Care Survey (3-year average, 2014–2016).

Rates for age groups are unadjusted and all other rates are age-adjusted.

**Table 50:** Appendicitis: Emergency department visits with first-listed and all-listed diagnoses by age and sex in the United States, 2018

Demographic Characteristics		First-Listed Diagnosis		All-Listed Diagnoses	
		Number in thousands	Rate per 100,000	Number in thousands	Rate per 100,000
**Age (years)**	**0 to 11**	52	108	55	113
	**12 to 24**	108	196	115	208
	**25 to 44**	112	129	123	141
	**45 to 54**	43	104	48	115
	**55 to 64**	35	83	41	96
	**65 to 74**	21	68	25	83
	**75 plus**	10	47	14	66
**Sex**	**Female**	177	111	198	123
	**Male**	205	131	223	142
**Total**		382	121	421	133

Source: Healthcare Cost and Utilization Project Nationwide Emergency Department Sample.

Rates for age groups are unadjusted and all other rates are age-adjusted.

**Table 51:** Appendicitis: Hospital discharges with first-listed and all-listed diagnoses by age, race, ethnicity, and sex in the United States, 2018

Demographic Characteristics		First-Listed Diagnosis		All-Listed Diagnoses	
		Number in thousands	Rate per 100,000	Number in thousands	Rate per 100,000
**Age (years)**	**0 to 11**	22	46	24	50
	**12 to 24**	36	64	40	72
	**25 to 44**	41	47	49	56
	**45 to 54**	20	48	25	60
	**55 to 64**	19	45	25	58
	**65 to 74**	14	45	18	60
	**75 plus**	8	38	12	56
**Race**	**White**	131	53	159	63
	**Black**	12	27	16	36
	**Other**	16	63	19	74
**Ethnicity**	**Hispanic**	41	66	48	79
	**Not Hispanic**	119	45	147	54
**Sex**	**Female**	73	44	91	54
	**Male**	86	54	103	64
**Total**		159	49	193	59

Source: Healthcare Cost and Utilization Project National Inpatient Sample.

Rates for age groups are unadjusted and all other rates are age-adjusted.

**Table 52:** Appendicitis: Deaths with underlying or underlying/other cause and lifetime years of life lost by age, race, ethnicity, and sex in the United States, 2019

Demographic Characteristics		Underlying Cause			Underlying/Other Cause		
		Number of Deaths	Rate per 100,000	Years of potential life lost in thousands	Number of Deaths	Rate per 100,000	Years of potential life lost in thousands
**Age (years)**	**0 to 11**	6	0.0	0.4	10	0.0	0.7
	**12 to 24**	7	0.0	0.4	14	0.0	0.9
	**25 to 44**	24	0.0	1.0	38	0.0	1.6
	**45 to 54**	22	0.1	0.7	54	0.1	1.7
	**55 to 64**	54	0.1	1.3	126	0.3	2.9
	**65 to 74**	77	0.2	1.2	153	0.5	2.4
	**75 plus**	201	0.9	1.4	336	1.5	2.4
**Race**	**White**	328	0.1	5.1	619	0.2	10.2
	**Black**	42	0.1	1.0	73	0.2	1.7
	**Other**	21	0.1	0.3	39	0.2	0.7
**Ethnicity**	**Hispanic**	33	0.1	0.9	70	0.2	1.9
	**Not Hispanic**	358	0.1	5.6	661	0.2	10.7
**Sex**	**Female**	177	0.1	2.7	336	0.2	5.8
	**Male**	214	0.1	3.7	395	0.2	6.8
**Total**		391	0.1	6.4	731	0.2	12.6

Source: Vital Statistics of the United States.

Rates for age groups are unadjusted and all other rates are age-adjusted.

Years of potential life lost is based on U.S. life Table 2006–2018 estimates of life expectancy at age of death.

**Table 53:** Appendicitis: Claims-based prevalence with first-listed and all-listed diagnoses by age, race-ethnicity, sex and region among privately insured enrollees, 2020

Demographic Characteristics		Number of enrollees	First-Listed Diagnosis		All-Listed Diagnoses	
			Number of enrollees with disease	Percent of enrollees with disease	Number of enrollees with disease	Percent of enrollees with disease
**Age (years)**	**0 to 11**	1,101,978	847	0.1%	892	0.1%
	**12 to 24**	1,451,892	2,688	0.2%	2,879	0.2%
	**25 to 44**	2,656,672	4,184	0.2%	4,672	0.2%
	**45 to 54**	1,452,320	1,873	0.1%	2,144	0.1%
	**55 to 64**	1,640,216	1,842	0.1%	2,236	0.1%
	**65 to 74**	2,840,615	2,387	0.1%	3,119	0.1%
	**75 plus**	2,576,241	1,510	0.1%	2,173	0.1%
**Race-ethnicity**	**White**	8,657,702	10,198	0.1%	12,007	0.1%
	**Black**	1,242,477	1,000	0.1%	1,298	0.1%
	**Hispanic**	1,493,457	1,941	0.1%	2,280	0.2%
	**Asian**	607,284	541	0.1%	623	0.1%
	**Unknown**	1,719,014	1,651	0.1%	1,907	0.1%
**Sex**	**Female**	7,199,363	7,551	0.1%	9,171	0.1%
	**Male**	6,520,571	7,780	0.1%	8,944	0.1%
**Region**	**Northeast**	1,526,275	1,556	0.1%	1,908	0.1%
	**Midwest**	3,322,846	4,273	0.1%	4,873	0.1%
	**South**	5,619,435	6,246	0.1%	7,488	0.1%
	**West**	3,251,378	3,256	0.1%	3,846	0.1%
**Total**		13,719,934	15,331	0.1%	18,115	0.1%

Source: De-identified Optum Clinformatic ^®^ Data Mart.

Enrollees with full enrollment in a commercial health plan or Medicare during the year.

**Table 54:** Appendicitis: Ambulatory care visits with first-listed and all-listed diagnoses by age, race-ethnicity, sex and region among privately insured enrollees, 2020

Demographic Characteristics		First-Listed Diagnosis		All-Listed Diagnoses	
		Number of events	Event rate per 100,000	Number of events	Event rate per 100,000
**Age (years)**	**0 to 11**	1,105	100	1,191	108
	**12 to 24**	3,575	246	3,879	267
	**25 to 44**	5,492	207	6,210	234
	**45 to 54**	2,503	172	2,938	202
	**55 to 64**	2,313	141	2,821	172
	**65 to 74**	2,725	96	3,445	121
	**75 plus**	1,750	68	2,517	98
**Race-ethnicity**	**White**	12,963	150	15,250	176
	**Black**	1,274	103	1,576	127
	**Hispanic**	2,447	164	2,924	196
	**Asian**	689	113	809	133
	**Unknown**	2,090	122	2,442	142
**Sex**	**Female**	9,717	135	11,752	163
	**Male**	9,746	149	11,249	173
**Region**	**Northeast**	2,113	138	2,566	168
	**Midwest**	5,385	162	6,135	185
	**South**	7,960	142	9,527	170
	**West**	4,005	123	4,773	147
**Total**		19,463	142	23,001	168

Source: De-identified Optum Clinformatic ^®^ Data Mart.

Enrollees with full enrollment in a commercial health plan or Medicare during the year.

**Table 55:** Appendicitis: Emergency department visits with first-listed and all-listed diagnoses by age, race-ethnicity, sex and region among privately insured enrollees, 2020

Demographic Characteristics		First-Listed Diagnosis		All-Listed Diagnoses	
		Number of events	Event rate per 100,000	Number of events	Event rate per 100,000
**Age (years)**	**0 to 11**	36	3	39	4
	**12 to 24**	151	10	173	12
	**25 to 44**	257	10	306	12
	**45 to 54**	174	12	214	15
	**55 to 64**	302	18	388	24
	**65 to 74**	1,382	49	1,805	64
	**75 plus**	896	35	1,209	47
**Race-ethnicity**	**White**	2,196	25	2,822	33
	**Black**	222	18	335	27
	**Hispanic**	381	26	496	33
	**Asian**	121	20	137	23
	**Unknown**	278	16	344	20
**Sex**	**Female**	1,737	24	2,267	31
	**Male**	1,461	22	1,867	29
**Region**	**Northeast**	375	25	440	29
	**Midwest**	917	28	1,087	33
	**South**	1,109	20	1,549	28
	**West**	797	25	1,058	33
**Total**		3,198	23	4,134	30

Source: De-identified Optum Clinformatic ^®^ Data Mart.

Enrollees with full enrollment in a commercial health plan or Medicare during the year.

**Table 56:** Appendicitis: Hospital discharges with first-listed and all-listed diagnoses by age, race-ethnicity, sex and region among privately insured enrollees, 2020

Demographic Characteristics		First-Listed Diagnosis		All-Listed Diagnoses	
		Number of events	Event rate per 100,000	Number of events	Event rate per 100,000
**Age (years)**	**0 to 11**	256	23	287	26
	**12 to 24**	409	28	504	35
	**25 to 44**	586	22	873	33
	**45 to 54**	394	27	609	42
	**55 to 64**	524	32	873	53
	**65 to 74**	899	32	1,572	55
	**75 plus**	751	29	1,368	53
**Race-ethnicity**	**White**	2,486	29	3,980	46
	**Black**	289	23	552	44
	**Hispanic**	506	34	766	51
	**Asian**	155	26	207	34
	**Unknown**	383	22	581	34
**Sex**	**Female**	1,847	26	3,029	42
	**Male**	1,972	30	3,057	47
**Region**	**Northeast**	493	32	769	50
	**Midwest**	918	28	1,450	44
	**South**	1,538	27	2,501	45
	**West**	870	27	1,366	42
**Total**		3,819	28	6,086	44

Source: De-identified Optum Clinformatic ^®^ Data Mart.

Enrollees with full enrollment in a commercial health plan or Medicare during the year.

**Table 57:** Appendicitis: Claims-based prevalence with first-listed and all-listed diagnoses by age, race, sex and region among fee-for-service, age-eligible Medicare beneficiaries, 2019

Demographic Characteristics		Number of enrollees	First-Listed Diagnosis		All-Listed Diagnoses	
			Number of enrollees with disease	Percent of enrollees with disease	Number of enrollees with disease	Percent of enrollees with disease
**Age (years)**	**65 to 69**	8,066,320	8,880	0.1%	10,440	0.1%
	**70 to 74**	6,936,940	6,960	0.1%	8,720	0.1%
	**75 to 79**	4,894,060	4,160	0.1%	5,560	0.1%
	**80 to 84**	3,335,340	2,220	0.1%	2,900	0.1%
	**85+**	3,578,120	2,100	0.1%	2,940	0.1%
**Race**	**White**	22,023,800	20,360	0.1%	25,260	0.1%
	**Black**	1,861,660	1,060	0.1%	1,500	0.1%
	**Other**	2,925,320	2,900	0.1%	3,800	0.1%
**Sex**	**Female**	14,975,560	13,420	0.1%	16,720	0.1%
	**Male**	11,835,220	10,900	0.1%	13,840	0.1%
**Region**	**Northeast**	4,748,700	4,060	0.1%	5,040	0.1%
	**Midwest**	6,069,800	5,280	0.1%	6,560	0.1%
	**South**	10,595,900	9,680	0.1%	12,300	0.1%
	**West**	5,396,380	5,300	0.1%	6,660	0.1%
**Total**		26,810,780	24,320	0.1%	30,560	0.1%

Source: Centers for Medicare & Medicaid Services, 5% Denominator and Claims Files.

Beneficiaries aged 65+ years with continuous and full Parts A and B enrollment and no Health Maintenance Organization enrollment during the year.

Unweighted counts were multiplied by 20 to produce counts for this table.

**Table 58:** Appendicitis: Ambulatory care visits with first-listed and all-listed diagnoses by age, race, sex and region among fee-for-service, age-eligible Medicare beneficiaries, 2019

Demographic Characteristics		First-Listed Diagnosis		All-Listed Diagnoses	
		Number of events	Event rate per 100,000	Number of events	Event rate per 100,000
**Age (years)**	**65 to 69**	8,880	110	11,680	145
	**70 to 74**	7,320	106	10,020	144
	**75 to 79**	4,140	85	6,100	125
	**80 to 84**	1,980	59	3,500	105
	**85+**	2,380	67	3,660	102
**Race**	**White**	21,080	96	29,600	134
	**Black**	1,140	61	1,700	91
	**Other**	2,480	85	3,660	125
**Sex**	**Female**	13,720	92	19,500	130
	**Male**	10,980	93	15,460	131
**Region**	**Northeast**	4,380	92	6,000	126
	**Midwest**	5,240	86	7,780	128
	**South**	10,100	95	13,780	130
	**West**	4,980	92	7,400	137
**Total**		24,700	92	34,960	130

Source: Centers for Medicare & Medicaid Services, 5% Denominator and Claims Files.

Beneficiaries aged 65+ years with continuous and full Parts A and B enrollment and no Health Maintenance Organization enrollment during the year.

Unweighted counts were multiplied by 20 to produce counts for this table.

**Table 59:** Appendicitis: Emergency department visits with first-listed and all-listed diagnoses by age, race, sex and region among fee-for-service, age-eligible Medicare beneficiaries, 2019

Demographic Characteristics		First-Listed Diagnosis		All-Listed Diagnoses	
		Number of events	Event rate per 100,000	Number of events	Event rate per 100,000
**Age (years)**	**65 to 69**	8,040	100	9,080	113
	**70 to 74**	6,020	87	7,500	108
	**75 to 79**	3,640	74	4,600	94
	**80 to 84**	1,760	53	2,220	67
	**85+**	1,940	54	2,600	73
**Race**	**White**	17,780	81	21,300	97
	**Black**	1,000	54	1,360	73
	**Other**	2,620	90	3,340	114
**Sex**	**Female**	11,680	78	13,980	93
	**Male**	9,720	82	12,020	102
**Region**	**Northeast**	3,760	79	4,640	98
	**Midwest**	4,280	71	5,220	86
	**South**	8,640	82	10,500	99
	**West**	4,720	87	5,640	105
**Total**		21,400	80	26,000	97

Source: Centers for Medicare & Medicaid Services, 5% Denominator and Claims Files.

Beneficiaries aged 65+ years with continuous and full Parts A and B enrollment and no Health Maintenance Organization enrollment during the year.

Unweighted counts were multiplied by 20 to produce counts for this table.

**Table 60:** Appendicitis: Hospital discharges with first-listed and all-listed diagnoses by age, race, sex and region among fee-for-service, age-eligible Medicare beneficiaries, 2019

Demographic Characteristics		First-Listed Diagnosis		All-Listed Diagnoses	
		Number of events	Event rate per 100,000	Number of events	Event rate per 100,000
**Age (years)**	**65 to 69**	4,240	53	5,360	66
	**70 to 74**	3,460	50	4,940	71
	**75 to 79**	2,020	41	3,280	67
	**80 to 84**	1,360	41	1,820	55
	**85+**	1,040	29	1,920	54
**Race**	**White**	9,820	45	13,920	63
	**Black**	760	41	1,100	59
	**Other**	1,540	53	2,300	79
**Sex**	**Female**	6,600	44	9,180	61
	**Male**	5,520	47	8,140	69
**Region**	**Northeast**	2,160	45	3,000	63
	**Midwest**	2,540	42	3,560	59
	**South**	4,840	46	6,960	66
	**West**	2,580	48	3,800	70
**Total**		12,120	45	17,320	65

Source: Centers for Medicare & Medicaid Services, 5% Denominator and Claims Files.

Beneficiaries aged 65+ years with continuous and full Parts A and B enrollment and no Health Maintenance Organization enrollment during the year.

Unweighted counts were multiplied by 20 to produce counts for this table.

**Table 61:** Abdominal Wall Hernia: Ambulatory care visits with first-listed and all-listed diagnoses by age, race, ethnicity, and sex in the United States, 2015

Demographic Characteristics		First-Listed Diagnosis		All-Listed Diagnoses	
		Number in thousands	Rate per 100,000	Number in thousands	Rate per 100,000
**Age (years)**	**0 to 11**	72	148	361	743
	**12 to 24**	128	228	146	261
	**25 to 44**	531	629	903	1,069
	**45 to 54**	711	1,651	1,022	2,372
	**55 to 64**	730	1,789	1,023	2,506
	**65 to 74**	618	2,243	895	3,251
	**75 plus**	421	2,085	781	3,864
**Race**	**White**	2,771	981	4,230	1,521
	**Black**	361	789	722	1,701
	**Other**	79	347	179	755
**Ethnicity**	**Hispanic**	572	1,254	820	1,751
	**Not Hispanic**	2,640	872	4,311	1,450
**Sex**	**Female**	930	525	1,790	1,034
	**Male**	2,282	1,339	3,341	2,004
**Total**		3,212	910	5,131	1,484

Source: National Ambulatory Medical Care Survey (3-year average, 2014–2016).

Rates for age groups are unadjusted and all other rates are age-adjusted.

**Table 62:** Abdominal Wall Hernia: Emergency department visits with first-listed and all-listed diagnoses by age and sex in the United States, 2018

Demographic Characteristics		First-Listed Diagnosis		All-Listed Diagnoses	
		Number in thousands	Rate per 100,000	Number in thousands	Rate per 100,000
**Age (years)**	**0 to 11**	15	31	35	72
	**12 to 24**	12	22	21	38
	**25 to 44**	82	94	159	183
	**45 to 54**	59	141	130	313
	**55 to 64**	60	141	148	351
	**65 to 74**	43	141	121	397
	**75 plus**	48	218	146	665
**Sex**	**Female**	115	64	304	165
	**Male**	203	121	457	269
**Total**		318	92	761	214

Source: Healthcare Cost and Utilization Project Nationwide Emergency Department Sample.

Rates for age groups are unadjusted and all other rates are age-adjusted.

**Table 63:** Abdominal Wall Hernia: Hospital discharges with first-listed and all-listed diagnoses by age, race, ethnicity, and sex in the United States, 2018

Demographic Characteristics		First-Listed Diagnosis		All-Listed Diagnoses	
		Number in thousands	Rate per 100,000	Number in thousands	Rate per 100,000
**Age (years)**	**0 to 11**	2	5	27	56
	**12 to 24**	1	2	4	8
	**25 to 44**	16	18	51	58
	**45 to 54**	22	53	65	155
	**55 to 64**	33	77	100	237
	**65 to 74**	31	100	97	319
	**75 plus**	31	143	108	491
**Race**	**White**	113	37	364	119
	**Black**	17	39	68	153
	**Other**	6	25	23	92
**Ethnicity**	**Hispanic**	17	37	57	122
	**Not Hispanic**	120	36	399	123
**Sex**	**Female**	71	36	210	108
	**Male**	64	36	242	138
**Total**		135	36	452	121

Source: Healthcare Cost and Utilization Project National Inpatient Sample.

Rates for age groups are unadjusted and all other rates are age-adjusted.

**Table 64:** Abdominal Wall Hernia: Deaths with underlying or underlying/other cause and lifetime years of life lost by age, race, ethnicity, and sex in the United States, 2019

Demographic Characteristics		Underlying Cause			Underlying/Other Cause		
		Number of Deaths	Rate per 100,000	Years of potential life lost in thousands	Number of Deaths	Rate per 100,000	Years of potential life lost in thousands
**Age (years)**	**0 to 11**	12	0.0	0.9	22	0.0	1.6
	**12 to 24**	4	0.0	0.2	9	0.0	0.5
	**25 to 44**	34	0.0	1.4	51	0.1	2.1
	**45 to 54**	87	0.2	2.7	154	0.4	4.8
	**55 to 64**	209	0.5	4.7	343	0.8	7.8
	**65 to 74**	328	1.0	5.1	548	1.7	8.6
	**75 plus**	884	3.9	6.0	1,345	6.0	9.2
**Race**	**White**	1,352	0.4	17.8	2,124	0.6	28.9
	**Black**	166	0.4	2.7	272	0.7	4.5
	**Other**	40	0.2	0.7	76	0.3	1.2
**Ethnicity**	**Hispanic**	110	0.3	1.9	183	0.5	3.3
	**Not Hispanic**	1,448	0.4	19.2	2,289	0.6	31.2
**Sex**	**Female**	766	0.3	9.9	1,133	0.5	15.3
	**Male**	792	0.5	11.2	1,339	0.8	19.3
**Total**		1,558	0.4	21.1	2,472	0.6	34.6

Source: Vital Statistics of the United States.

Rates for age groups are unadjusted and all other rates are age-adjusted.

Years of potential life lost is based on U.S. life Table 2006–2018 estimates of life expectancy at age of death.

**Table 65:** Abdominal Wall Hernia: Claims-based prevalence with first-listed and all-listed diagnoses by age, race-ethnicity, sex and region among privately insured enrollees, 2020

Demographic Characteristics		Number of enrollees	First-Listed Diagnosis		All-Listed Diagnoses	
			Number of enrollees with disease	Percent of enrollees with disease	Number of enrollees with disease	Percent of enrollees with disease
**Age (years)**	**0 to 11**	1,101,978	2,295	0.2%	7,404	0.7%
	**12 to 24**	1,451,892	1,172	0.1%	1,650	0.1%
	**25 to 44**	2,656,672	9,438	0.4%	14,243	0.5%
	**45 to 54**	1,452,320	9,486	0.7%	15,840	1.1%
	**55 to 64**	1,640,216	14,120	0.9%	24,223	1.5%
	**65 to 74**	2,840,615	25,399	0.9%	43,577	1.5%
	**75 plus**	2,576,241	21,367	0.8%	40,099	1.6%
**Race-ethnicity**	**White**	8,657,702	58,240	0.7%	98,395	1.1%
	**Black**	1,242,477	7,471	0.6%	13,666	1.1%
	**Hispanic**	1,493,457	8,382	0.6%	15,699	1.1%
	**Asian**	607,284	1,488	0.2%	2,675	0.4%
	**Unknown**	1,719,014	7,696	0.4%	16,601	1.0%
**Sex**	**Female**	7,199,363	25,247	0.4%	48,911	0.7%
	**Male**	6,520,571	58,030	0.9%	98,125	1.5%
**Region**	**Northeast**	1,526,275	10,040	0.7%	17,758	1.2%
	**Midwest**	3,322,846	19,518	0.6%	33,129	1.0%
	**South**	5,619,435	34,651	0.6%	62,930	1.1%
	**West**	3,251,378	19,068	0.6%	33,219	1.0%
**Total**		13,719,934	83,277	0.6%	147,036	1.1%

Source: De-identified Optum Clinformatic ^®^ Data Mart.

Enrollees with full enrollment in a commercial health plan or Medicare during the year.

**Table 66:** Abdominal Wall Hernia: Ambulatory care visits with first-listed and all-listed diagnoses by age, race-ethnicity, sex and region among privately insured enrollees, 2020

Demographic Characteristics		First-Listed Diagnosis		All-Listed Diagnoses	
		Number of events	Event rate per 100,000	Number of events	Event rate per 100,000
**Age (years)**	**0 to 11**	3,646	331	11,690	1,061
	**12 to 24**	2,088	144	2,869	198
	**25 to 44**	17,177	647	26,042	980
	**45 to 54**	17,590	1,211	29,299	2,017
	**55 to 64**	25,941	1,582	44,819	2,733
	**65 to 74**	45,825	1,613	79,931	2,814
	**75 plus**	37,007	1,436	69,688	2,705
**Race-ethnicity**	**White**	105,416	1,218	178,404	2,061
	**Black**	12,994	1,046	24,102	1,940
	**Hispanic**	14,757	988	28,056	1,879
	**Asian**	2,750	453	4,822	794
	**Unknown**	13,357	777	28,954	1,684
**Sex**	**Female**	41,413	575	82,451	1,145
	**Male**	107,861	1,654	181,887	2,789
**Region**	**Northeast**	17,803	1,166	31,852	2,087
	**Midwest**	34,524	1,039	60,305	1,815
	**South**	62,477	1,112	111,778	1,989
	**West**	34,470	1,060	60,403	1,858
**Total**		149,274	1,088	264,338	1,927

Source: De-identified Optum Clinformatic ^®^ Data Mart.

Enrollees with full enrollment in a commercial health plan or Medicare during the year.

**Table 67:** Abdominal Wall Hernia: Emergency department visits with first-listed and all-listed diagnoses by age, race-ethnicity, sex and region among privately insured enrollees, 2020

Demographic Characteristics		First-Listed Diagnosis		All-Listed Diagnoses	
		Number of events	Event rate per 100,000	Number of events	Event rate per 100,000
**Age (years)**	**0 to 11**	14	1	22	2
	**12 to 24**	8	1	10	1
	**25 to 44**	161	6	315	12
	**45 to 54**	225	15	433	30
	**55 to 64**	615	37	1,156	70
	**65 to 74**	1,832	64	3,394	119
	**75 plus**	2,586	100	4,676	182
**Race-ethnicity**	**White**	3,588	41	6,501	75
	**Black**	708	57	1,335	107
	**Hispanic**	591	40	1,176	79
	**Asian**	69	11	121	20
	**Unknown**	485	28	873	51
**Sex**	**Female**	2,042	28	4,208	58
	**Male**	3,399	52	5,798	89
**Region**	**Northeast**	820	54	1,294	85
	**Midwest**	1,155	35	2,020	61
	**South**	2,181	39	4,319	77
	**West**	1,285	40	2,373	73
**Total**		5,441	40	10,006	73

Source: De-identified Optum Clinformatic ^®^ Data Mart.

Enrollees with full enrollment in a commercial health plan or Medicare during the year.

**Table 68:** Abdominal Wall Hernia: Hospital discharges with first-listed and all-listed diagnoses by age, race-ethnicity, sex and region among privately insured enrollees, 2020

Demographic Characteristics		First-Listed Diagnosis		All-Listed Diagnoses	
		Number of events	Event rate per 100,000	Number of events	Event rate per 100,000
**Age (years)**	**0 to 11**	16	1	415	38
	**12 to 24**	10	1	53	4
	**25 to 44**	223	8	871	33
	**45 to 54**	482	33	1,635	113
	**55 to 64**	970	59	3,389	207
	**65 to 74**	1,933	68	7,179	253
	**75 plus**	2,341	91	9,115	354
**Race-ethnicity**	**White**	4,088	47	14,889	172
	**Black**	683	55	2,738	220
	**Hispanic**	671	45	2,621	175
	**Asian**	60	10	277	46
	**Unknown**	473	28	2,132	124
**Sex**	**Female**	3,355	47	10,764	150
	**Male**	2,620	40	11,893	182
**Region**	**Northeast**	798	52	2,969	195
	**Midwest**	1,292	39	4,713	142
	**South**	2,656	47	10,418	185
	**West**	1,229	38	4,557	140
**Total**		5,975	44	22,657	165

Source: De-identified Optum Clinformatic ^®^ Data Mart.

Enrollees with full enrollment in a commercial health plan or Medicare during the year.

**Table 69:** Abdominal Wall Hernia: Claims-based prevalence with first-listed and all-listed diagnoses by age, race, sex and region among fee-for-service, age-eligible Medicare beneficiaries, 2019

Demographic Characteristics		Number of enrollees	First-Listed Diagnosis		All-Listed Diagnoses	
			Number of enrollees with disease	Percent of enrollees with disease	Number of enrollees with disease	Percent of enrollees with disease
**Age (years)**	**65 to 69**	8,066,320	97,040	1.2%	172,600	2.1%
	**70 to 74**	6,936,940	88,460	1.3%	160,700	2.3%
	**75 to 79**	4,894,060	64,960	1.3%	124,660	2.5%
	**80 to 84**	3,335,340	42,360	1.3%	83,440	2.5%
	**85+**	3,578,120	37,000	1.0%	83,860	2.3%
**Race**	**White**	22,023,800	279,360	1.3%	527,980	2.4%
	**Black**	1,861,660	17,580	0.9%	36,220	1.9%
	**Other**	2,925,320	32,880	1.1%	61,060	2.1%
**Sex**	**Female**	14,975,560	104,140	0.7%	218,560	1.5%
	**Male**	11,835,220	225,680	1.9%	406,700	3.4%
**Region**	**Northeast**	4,748,700	61,540	1.3%	121,220	2.6%
	**Midwest**	6,069,800	71,240	1.2%	134,160	2.2%
	**South**	10,595,900	127,460	1.2%	244,080	2.3%
	**West**	5,396,380	69,580	1.3%	125,800	2.3%
**Total**		26,810,780	329,820	1.2%	625,260	2.3%

Source: Centers for Medicare & Medicaid Services, 5% Denominator and Claims Files.

Beneficiaries aged 65+ years with continuous and full Parts A and B enrollment and no Health Maintenance Organization enrollment during the year.

Unweighted counts were multiplied by 20 to produce counts for this table.

**Table 70:** Abdominal Wall Hernia: Ambulatory care visits with first-listed and all-listed diagnoses by age, race, sex and region among fee-for-service, age-eligible Medicare beneficiaries, 2019

Demographic Characteristics		First-Listed Diagnosis		All-Listed Diagnoses	
		Number of events	Event rate per 100,000	Number of events	Event rate per 100,000
**Age (years)**	**65 to 69**	176,900	2,193	313,600	3,888
	**70 to 74**	161,920	2,334	291,860	4,207
	**75 to 79**	116,000	2,370	218,780	4,470
	**80 to 84**	73,800	2,213	144,340	4,328
	**85+**	54,140	1,513	119,120	3,329
**Race**	**White**	496,400	2,254	919,520	4,175
	**Black**	27,700	1,488	57,620	3,095
	**Other**	58,660	2,005	110,560	3,779
**Sex**	**Female**	164,580	1,099	342,560	2,287
	**Male**	418,180	3,533	745,140	6,296
**Region**	**Northeast**	109,020	2,296	211,560	4,455
	**Midwest**	123,680	2,038	231,100	3,807
	**South**	227,320	2,145	416,720	3,933
	**West**	122,740	2,274	228,320	4,231
**Total**		582,760	2,174	1,087,700	4,057

Source: Centers for Medicare & Medicaid Services, 5% Denominator and Claims Files.

Beneficiaries aged 65+ years with continuous and full Parts A and B enrollment and no Health Maintenance Organization enrollment during the year.

Unweighted counts were multiplied by 20 to produce counts for this table.

**Table 71:** Abdominal Wall Hernia: Emergency department visits with first-listed and all-listed diagnoses by age, race, sex and region among fee-for-service, age-eligible Medicare beneficiaries, 2019

Demographic Characteristics		First-Listed Diagnosis		All-Listed Diagnoses	
		Number of events	Event rate per 100,000	Number of events	Event rate per 100,000
**Age (years)**	**65 to 69**	13,280	165	37,340	463
	**70 to 74**	13,900	200	38,220	551
	**75 to 79**	11,300	231	33,340	681
	**80 to 84**	8,900	267	27,800	833
	**85+**	12,180	340	37,160	1,039
**Race**	**White**	48,080	218	141,060	640
	**Black**	5,520	297	15,820	850
	**Other**	5,960	204	16,980	580
**Sex**	**Female**	25,280	169	76,720	512
	**Male**	34,280	290	97,140	821
**Region**	**Northeast**	12,420	262	34,600	729
	**Midwest**	13,420	221	37,060	611
	**South**	22,140	209	69,420	655
	**West**	11,580	215	32,780	607
**Total**		59,560	222	173,860	648

Source: Centers for Medicare & Medicaid Services, 5% Denominator and Claims Files.

Beneficiaries aged 65+ years with continuous and full Parts A and B enrollment and no Health Maintenance Organization enrollment during the year.

Unweighted counts were multiplied by 20 to produce counts for this table.

**Table 72:** Abdominal Wall Hernia: Hospital discharges with first-listed and all-listed diagnoses by age, race, sex and region among fee-for-service, age-eligible Medicare beneficiaries, 2019

Demographic Characteristics		First-Listed Diagnosis		All-Listed Diagnoses	
		Number of events	Event rate per 100,000	Number of events	Event rate per 100,000
**Age (years)**	**65 to 69**	7,840	97	26,380	327
	**70 to 74**	7,780	112	26,140	377
	**75 to 79**	6,560	134	23,440	479
	**80 to 84**	5,140	154	18,140	544
	**85+**	5,640	158	21,720	607
**Race**	**White**	27,220	124	95,320	433
	**Black**	2,540	136	9,500	510
	**Other**	3,200	109	11,000	376
**Sex**	**Female**	18,060	121	55,960	374
	**Male**	14,900	126	59,860	506
**Region**	**Northeast**	6,500	137	21,880	461
	**Midwest**	7,380	122	25,740	424
	**South**	13,520	128	47,640	450
	**West**	5,560	103	20,560	381
**Total**		32,960	123	115,820	432

Source: Centers for Medicare & Medicaid Services, 5% Denominator and Claims Files.

Beneficiaries aged 65+ years with continuous and full Parts A and B enrollment and no Health Maintenance Organization enrollment during the year.

Unweighted counts were multiplied by 20 to produce counts for this table.

**Table 73:** Crohns Disease: Ambulatory care visits with first-listed and all-listed diagnoses by age, race, ethnicity, and sex in the United States, 2015

Demographic Characteristics		First-Listed Diagnosis		All-Listed Diagnoses	
		Number in thousands	Rate per 100,000	Number in thousands	Rate per 100,000
**Age (years)**	**0 to 11**	.	.	1	2
	**12 to 24**	26	46	63	112
	**25 to 44**	148	176	244	288
	**45 to 54**	110	255	257	596
	**55 to 64**	110	269	211	516
	**65 to 74**	144	524	213	774
	**75 plus**	8	41	54	270
**Race**	**White**	445	159	827	294
	**Black**	31	58	105	240
	**Other**	71	289	111	518
**Ethnicity**	**Hispanic**	41	63	92	227
	**Not Hispanic**	506	169	951	318
**Sex**	**Female**	319	169	604	323
	**Male**	228	141	438	276
**Total**		547	156	1,043	300

Source: National Ambulatory Medical Care Survey (3-year average, 2014–2016).

Rates for age groups are unadjusted and all other rates are age-adjusted.

**Table 74:** Crohns Disease: Emergency department visits with first-listed and all-listed diagnoses by age and sex in the United States, 2018

Demographic Characteristics		First-Listed Diagnosis		All-Listed Diagnoses	
		Number in thousands	Rate per 100,000	Number in thousands	Rate per 100,000
**Age (years)**	**0 to 11**	1	2	3	6
	**12 to 24**	15	28	38	68
	**25 to 44**	45	52	143	164
	**45 to 54**	14	34	63	150
	**55 to 64**	10	22	53	124
	**65 to 74**	6	19	41	135
	**75 plus**	3	16	35	160
**Sex**	**Female**	51	31	220	129
	**Male**	44	28	155	95
**Total**		95	30	375	113

Source: Healthcare Cost and Utilization Project Nationwide Emergency Department Sample.

Rates for age groups are unadjusted and all other rates are age-adjusted.

**Table 75:** Crohns Disease: Hospital discharges with first-listed and all-listed diagnoses by age, race, ethnicity, and sex in the United States, 2018

Demographic Characteristics		First-Listed Diagnosis		All-Listed Diagnoses	
		Number in thousands	Rate per 100,000	Number in thousands	Rate per 100,000
**Age (years)**	**0 to 11**	1	2	1	3
	**12 to 24**	10	18	18	32
	**25 to 44**	25	28	60	69
	**45 to 54**	10	23	32	76
	**55 to 64**	7	18	35	83
	**65 to 74**	5	17	34	110
	**75 plus**	3	14	27	124
**Race**	**White**	49	19	177	64
	**Black**	9	20	25	55
	**Other**	3	10	7	27
**Ethnicity**	**Hispanic**	4	8	12	23
	**Not Hispanic**	57	21	197	67
**Sex**	**Female**	33	20	119	66
	**Male**	28	18	88	52
**Total**		61	19	207	59

Source: Healthcare Cost and Utilization Project National Inpatient Sample.

Rates for age groups are unadjusted and all other rates are age-adjusted.

**Table 76:** Crohns Disease: Deaths with underlying or underlying/other cause and lifetime years of life lost by age, race, ethnicity, and sex in the United States, 2019

Demographic Characteristics		Underlying Cause			Underlying/Other Cause		
		Number of Deaths	Rate per 100,000	Years of potential life lost in thousands	Number of Deaths	Rate per 100,000	Years of potential life lost in thousands
**Age (years)**	**0 to 11**	.	.	.	.	.	.
	**12 to 24**	4	0.0	0.2	8	0.0	0.5
	**25 to 44**	57	0.1	2.4	141	0.2	6.0
	**45 to 54**	71	0.2	2.2	201	0.5	6.2
	**55 to 64**	100	0.2	2.3	360	0.8	8.4
	**65 to 74**	183	0.6	2.9	576	1.8	9.1
	**75 plus**	264	1.2	2.1	899	4.0	6.9
**Race**	**White**	625	0.2	10.9	2,002	0.6	33.2
	**Black**	45	0.1	1.1	151	0.4	3.3
	**Other**	9	0.0	0.2	32	0.1	0.6
**Ethnicity**	**Hispanic**	15	0.0	0.3	45	0.1	0.9
	**Not Hispanic**	664	0.2	11.8	2,140	0.6	36.2
**Sex**	**Female**	394	0.2	6.8	1,202	0.5	20.6
	**Male**	285	0.2	5.3	983	0.6	16.5
**Total**		679	0.2	12.1	2,185	0.6	37.1

Source: Vital Statistics of the United States.

Rates for age groups are unadjusted and all other rates are age-adjusted.

Years of potential life lost is based on U.S. life Table 2006–2018 estimates of life expectancy at age of death.

**Table 77:** Crohns Disease: Claims-based prevalence with first-listed and all-listed diagnoses by age, race-ethnicity, sex and region among privately insured enrollees, 2020

Demographic Characteristics		Number of enrollees	First-Listed Diagnosis		All-Listed Diagnoses	
			Number of enrollees with disease	Percent of enrollees with disease	Number of enrollees with disease	Percent of enrollees with disease
**Age (years)**	**0 to 11**	1,101,978	140	0.0%	165	0.0%
	**12 to 24**	1,451,892	2,344	0.2%	2,679	0.2%
	**25 to 44**	2,656,672	6,136	0.2%	7,982	0.3%
	**45 to 54**	1,452,320	3,548	0.2%	5,195	0.4%
	**55 to 64**	1,640,216	3,893	0.2%	6,250	0.4%
	**65 to 74**	2,840,615	5,073	0.2%	9,015	0.3%
	**75 plus**	2,576,241	3,087	0.1%	6,764	0.3%
**Race-ethnicity**	**White**	8,657,702	18,684	0.2%	29,160	0.3%
	**Black**	1,242,477	2,009	0.2%	3,212	0.3%
	**Hispanic**	1,493,457	1,331	0.1%	2,259	0.2%
	**Asian**	607,284	436	0.1%	685	0.1%
	**Unknown**	1,719,014	1,761	0.1%	2,734	0.2%
**Sex**	**Female**	7,199,363	13,261	0.2%	21,422	0.3%
	**Male**	6,520,571	10,960	0.2%	16,628	0.3%
**Region**	**Northeast**	1,526,275	3,252	0.2%	5,026	0.3%
	**Midwest**	3,322,846	6,776	0.2%	10,225	0.3%
	**South**	5,619,435	9,911	0.2%	15,609	0.3%
	**West**	3,251,378	4,282	0.1%	7,190	0.2%
**Total**		13,719,934	24,221	0.2%	38,050	0.3%

Source: De-identified Optum Clinformatic ^®^ Data Mart.

Enrollees with full enrollment in a commercial health plan or Medicare during the year.

**Table 78:** Crohns Disease: Ambulatory care visits with first-listed and all-listed diagnoses by age, race-ethnicity, sex and region among privately insured enrollees, 2020

Demographic Characteristics		First-Listed Diagnosis		All-Listed Diagnoses	
		Number of events	Event rate per 100,000	Number of events	Event rate per 100,000
**Age (years)**	**0 to 11**	440	40	605	55
	**12 to 24**	6,537	450	9,135	629
	**25 to 44**	15,319	577	25,165	947
	**45 to 54**	8,479	584	16,955	1,167
	**55 to 64**	8,688	530	19,693	1,201
	**65 to 74**	10,558	372	25,974	914
	**75 plus**	6,231	242	18,133	704
**Race-ethnicity**	**White**	42,985	496	88,152	1,018
	**Black**	4,734	381	10,239	824
	**Hispanic**	3,188	213	6,921	463
	**Asian**	1,144	188	2,106	347
	**Unknown**	4,201	244	8,242	479
**Sex**	**Female**	31,386	436	67,854	943
	**Male**	24,866	381	47,806	733
**Region**	**Northeast**	7,753	508	15,475	1,014
	**Midwest**	14,393	433	28,482	857
	**South**	24,313	433	50,239	894
	**West**	9,793	301	21,464	660
**Total**		56,252	410	115,660	843

Source: De-identified Optum Clinformatic ^®^ Data Mart.

Enrollees with full enrollment in a commercial health plan or Medicare during the year.

**Table 79:** Crohns Disease: Emergency department visits with first-listed and all-listed diagnoses by age, race-ethnicity, sex and region among privately insured enrollees, 2020

Demographic Characteristics		First-Listed Diagnosis		All-Listed Diagnoses	
		Number of events	Event rate per 100,000	Number of events	Event rate per 100,000
**Age (years)**	**0 to 11**	2	0	2	0
	**12 to 24**	40	3	67	5
	**25 to 44**	308	12	663	25
	**45 to 54**	231	16	421	29
	**55 to 64**	147	9	328	20
	**65 to 74**	170	6	473	17
	**75 plus**	106	4	382	15
**Race-ethnicity**	**White**	738	9	1,761	20
	**Black**	115	9	242	19
	**Hispanic**	59	4	129	9
	**Asian**	13	2	24	4
	**Unknown**	79	5	180	10
**Sex**	**Female**	568	8	1,446	20
	**Male**	436	7	890	14
**Region**	**Northeast**	126	8	368	24
	**Midwest**	231	7	551	17
	**South**	496	9	1,036	18
	**West**	151	5	381	12
**Total**		1,004	7	2,336	17

Source: De-identified Optum Clinformatic ^®^ Data Mart.

Enrollees with full enrollment in a commercial health plan or Medicare during the year.

**Table 80:** Crohns Disease: Hospital discharges with first-listed and all-listed diagnoses by age, race-ethnicity, sex and region among privately insured enrollees, 2020

Demographic Characteristics		First-Listed Diagnosis		All-Listed Diagnoses	
		Number of events	Event rate per 100,000	Number of events	Event rate per 100,000
**Age (years)**	**0 to 11**	18	2	23	2
	**12 to 24**	208	14	378	26
	**25 to 44**	512	19	1,418	53
	**45 to 54**	268	18	1,031	71
	**55 to 64**	286	17	1,536	94
	**65 to 74**	319	11	2,414	85
	**75 plus**	231	9	2,412	94
**Race-ethnicity**	**White**	1,302	15	6,853	79
	**Black**	198	16	988	80
	**Hispanic**	113	8	531	36
	**Asian**	40	7	118	19
	**Unknown**	189	11	722	42
**Sex**	**Female**	1,043	14	5,429	75
	**Male**	799	12	3,783	58
**Region**	**Northeast**	256	17	1,208	79
	**Midwest**	458	14	2,272	68
	**South**	815	15	4,042	72
	**West**	313	10	1,690	52
**Total**		1,842	13	9,212	67

Source: De-identified Optum Clinformatic ^®^ Data Mart.

Enrollees with full enrollment in a commercial health plan or Medicare during the year.

**Table 81:** Crohns Disease: Claims-based prevalence with first-listed and all-listed diagnoses by age, race, sex and region among fee-for-service, age-eligible Medicare beneficiaries, 2019

Demographic Characteristics		Number of enrollees	First-Listed Diagnosis		All-Listed Diagnoses	
			Number of enrollees with disease	Percent of enrollees with disease	Number of enrollees with disease	Percent of enrollees with disease
**Age (years)**	**65 to 69**	8,066,320	21,000	0.3%	33,780	0.4%
	**70 to 74**	6,936,940	17,420	0.3%	28,860	0.4%
	**75 to 79**	4,894,060	11,540	0.2%	20,140	0.4%
	**80 to 84**	3,335,340	6,400	0.2%	12,200	0.4%
	**85+**	3,578,120	3,780	0.1%	8,360	0.2%
**Race**	**White**	22,023,800	53,380	0.2%	92,340	0.4%
	**Black**	1,861,660	2,520	0.1%	4,360	0.2%
	**Other**	2,925,320	4,240	0.1%	6,640	0.2%
**Sex**	**Female**	14,975,560	34,860	0.2%	60,900	0.4%
	**Male**	11,835,220	25,280	0.2%	42,440	0.4%
**Region**	**Northeast**	4,748,700	14,000	0.3%	22,860	0.5%
	**Midwest**	6,069,800	14,120	0.2%	24,700	0.4%
	**South**	10,595,900	22,580	0.2%	39,340	0.4%
	**West**	5,396,380	9,440	0.2%	16,440	0.3%
**Total**		26,810,780	60,140	0.2%	103,340	0.4%

Source: Centers for Medicare & Medicaid Services, 5% Denominator and Claims Files.

Beneficiaries aged 65+ years with continuous and full Parts A and B enrollment and no Health Maintenance Organization enrollment during the year.

Unweighted counts were multiplied by 20 to produce counts for this table.

**Table 82:** Crohns Disease: Ambulatory care visits with first-listed and all-listed diagnoses by age, race, sex and region among fee-for-service, age-eligible Medicare beneficiaries, 2019

Demographic Characteristics		First-Listed Diagnosis		All-Listed Diagnoses	
		Number of events	Event rate per 100,000	Number of events	Event rate per 100,000
**Age (years)**	**65 to 69**	63,380	786	127,640	1,582
	**70 to 74**	53,740	775	109,200	1,574
	**75 to 79**	32,420	662	72,000	1,471
	**80 to 84**	18,900	567	41,500	1,244
	**85+**	9,180	257	26,100	729
**Race**	**White**	157,740	716	335,440	1,523
	**Black**	7,100	381	15,620	839
	**Other**	12,780	437	25,380	868
**Sex**	**Female**	100,300	670	223,340	1,491
	**Male**	77,320	653	153,100	1,294
**Region**	**Northeast**	39,740	837	85,980	1,811
	**Midwest**	39,720	654	84,440	1,391
	**South**	70,520	666	147,480	1,392
	**West**	27,640	512	58,540	1,085
**Total**		177,620	662	376,440	1,404

Source: Centers for Medicare & Medicaid Services, 5% Denominator and Claims Files.

Beneficiaries aged 65+ years with continuous and full Parts A and B enrollment and no Health Maintenance Organization enrollment during the year.

Unweighted counts were multiplied by 20 to produce counts for this table.

**Table 83:** Crohns Disease: Emergency department visits with first-listed and all-listed diagnoses by age, race, sex and region among fee-for-service, age-eligible Medicare beneficiaries, 2019

Demographic Characteristics		First-Listed Diagnosis		All-Listed Diagnoses	
		Number of events	Event rate per 100,000	Number of events	Event rate per 100,000
**Age (years)**	**65 to 69**	2,000	25	13,420	166
	**70 to 74**	1,580	23	12,240	176
	**75 to 79**	1,300	27	10,540	215
	**80 to 84**	960	29	7,500	225
	**85+**	340	10	5,740	160
**Race**	**White**	5,280	24	43,680	198
	**Black**	420	23	2,800	150
	**Other**	480	16	2,960	101
**Sex**	**Female**	4,080	27	30,940	207
	**Male**	2,100	18	18,500	156
**Region**	**Northeast**	1,560	33	11,340	239
	**Midwest**	1,260	21	12,000	198
	**South**	2,520	24	18,820	178
	**West**	840	16	7,280	135
**Total**		6,180	23	49,440	184

Source: Centers for Medicare & Medicaid Services, 5% Denominator and Claims Files.

Beneficiaries aged 65+ years with continuous and full Parts A and B enrollment and no Health Maintenance Organization enrollment during the year.

Unweighted counts were multiplied by 20 to produce counts for this table.

**Table 84:** Crohns Disease: Hospital discharges with first-listed and all-listed diagnoses by age, race, sex and region among fee-for-service, age-eligible Medicare beneficiaries, 2019

Demographic Characteristics		First-Listed Diagnosis		All-Listed Diagnoses	
		Number of events	Event rate per 100,000	Number of events	Event rate per 100,000
**Age (years)**	**65 to 69**	1,500	19	9,540	118
	**70 to 74**	1,280	18	9,600	138
	**75 to 79**	860	18	8,720	178
	**80 to 84**	600	18	5,360	161
	**85+**	380	11	4,500	126
**Race**	**White**	4,000	18	33,520	152
	**Black**	280	15	2,060	111
	**Other**	340	12	2,140	73
**Sex**	**Female**	2,700	18	23,340	156
	**Male**	1,920	16	14,380	122
**Region**	**Northeast**	1,200	25	8,380	176
	**Midwest**	1,000	16	9,760	161
	**South**	1,860	18	14,200	134
	**West**	560	10	5,380	100
**Total**		4,620	17	37,720	141

Source: Centers for Medicare & Medicaid Services, 5% Denominator and Claims Files.

Beneficiaries aged 65+ years with continuous and full Parts A and B enrollment and no Health Maintenance Organization enrollment during the year.

Unweighted counts were multiplied by 20 to produce counts for this table.

**Table 85:** Ulcerative Colitis: Ambulatory care visits with first-listed and all-listed diagnoses by age, race, ethnicity, and sex in the United States, 2015

Demographic Characteristics		First-Listed Diagnosis		All-Listed Diagnoses	
		Number in thousands	Rate per 100,000	Number in thousands	Rate per 100,000
**Age (years)**	**0 to 11**	.	.	.	.
	**12 to 24**	38	67	110	196
	**25 to 44**	64	76	128	152
	**45 to 54**	343	795	372	864
	**55 to 64**	240	587	358	878
	**65 to 74**	65	235	269	976
	**75 plus**	39	195	68	338
**Race**	**White**	565	203	1,011	351
	**Black**	188	385	248	510
	**Other**	36	218	47	288
**Ethnicity**	**Hispanic**	259	598	339	807
	**Not Hispanic**	530	167	968	304
**Sex**	**Female**	418	235	796	431
	**Male**	370	211	510	285
**Total**		789	221	1,306	357

Source: National Ambulatory Medical Care Survey (3-year average, 2014–2016).

Rates for age groups are unadjusted and all other rates are age-adjusted.

**Table 86:** Ulcerative Colitis: Emergency department visits with first-listed and all-listed diagnoses by age and sex in the United States, 2018

Demographic Characteristics		First-Listed Diagnosis		All-Listed Diagnoses	
		Number in thousands	Rate per 100,000	Number in thousands	Rate per 100,000
**Age (years)**	**0 to 11**	1	1	1	3
	**12 to 24**	8	15	16	28
	**25 to 44**	21	24	46	53
	**45 to 54**	7	18	22	54
	**55 to 64**	7	17	25	60
	**65 to 74**	5	17	25	81
	**75 plus**	5	24	29	132
**Sex**	**Female**	30	18	91	50
	**Male**	24	15	74	44
**Total**		55	16	164	47

Source: Healthcare Cost and Utilization Project Nationwide Emergency Department Sample.

Rates for age groups are unadjusted and all other rates are age-adjusted.

**Table 87:** Ulcerative Colitis: Hospital discharges with first-listed and all-listed diagnoses by age, race, ethnicity, and sex in the United States, 2018

Demographic Characteristics		First-Listed Diagnosis		All-Listed Diagnoses	
		Number in thousands	Rate per 100,000	Number in thousands	Rate per 100,000
**Age (years)**	**0 to 11**	1	2	1	2
	**12 to 24**	6	11	11	19
	**25 to 44**	12	14	29	33
	**45 to 54**	5	12	16	38
	**55 to 64**	6	13	21	51
	**65 to 74**	5	15	23	77
	**75 plus**	4	20	27	122
**Race**	**White**	32	12	110	38
	**Black**	4	10	12	27
	**Other**	2	9	6	24
**Ethnicity**	**Hispanic**	5	9	11	22
	**Not Hispanic**	34	12	118	38
**Sex**	**Female**	21	12	68	36
	**Male**	18	11	59	35
**Total**		39	11	128	36

Source: Healthcare Cost and Utilization Project National Inpatient Sample.

Rates for age groups are unadjusted and all other rates are age-adjusted.

**Table 88:** Ulcerative Colitis: Deaths with underlying or underlying/other cause and lifetime years of life lost by age, race, ethnicity, and sex in the United States, 2019

Demographic Characteristics		Underlying Cause			Underlying/Other Cause		
		Number of Deaths	Rate per 100,000	Years of potential life lost in thousands	Number of Deaths	Rate per 100,000	Years of potential life lost in thousands
**Age (years)**	**0 to 11**	.	.	.	.	.	.
	**12 to 24**	3	0.0	0.2	13	0.0	0.8
	**25 to 44**	15	0.0	0.6	55	0.1	2.4
	**45 to 54**	24	0.1	0.8	83	0.2	2.6
	**55 to 64**	54	0.1	1.3	186	0.4	4.3
	**65 to 74**	104	0.3	1.6	329	1.0	5.2
	**75 plus**	224	1.0	1.6	800	3.5	5.8
**Race**	**White**	377	0.1	5.2	1,346	0.4	18.7
	**Black**	36	0.1	0.7	92	0.2	1.8
	**Other**	11	0.0	0.2	28	0.1	0.6
**Ethnicity**	**Hispanic**	12	0.0	0.3	47	0.1	1.2
	**Not Hispanic**	412	0.1	5.8	1,419	0.4	19.8
**Sex**	**Female**	227	0.1	3.3	708	0.3	10.3
	**Male**	197	0.1	2.8	758	0.4	10.7
**Total**		424	0.1	6.1	1,466	0.4	21.0

Source: Vital Statistics of the United States.

Rates for age groups are unadjusted and all other rates are age-adjusted.

Years of potential life lost is based on U.S. life Table 2006–2018 estimates of life expectancy at age of death.

**Table 89:** Ulcerative Colitis: Claims-based prevalence with first-listed and all-listed diagnoses by age, race-ethnicity, sex and region among privately insured enrollees, 2020

Demographic Characteristics		Number of enrollees	First-Listed Diagnosis		All-Listed Diagnoses	
			Number of enrollees with disease	Percent of enrollees with disease	Number of enrollees with disease	Percent of enrollees with disease
**Age (years)**	**0 to 11**	1,101,978	87	0.0%	116	0.0%
	**12 to 24**	1,451,892	1,472	0.1%	1,786	0.1%
	**25 to 44**	2,656,672	6,106	0.2%	8,123	0.3%
	**45 to 54**	1,452,320	3,496	0.2%	5,809	0.4%
	**55 to 64**	1,640,216	4,142	0.3%	7,532	0.5%
	**65 to 74**	2,840,615	6,725	0.2%	13,668	0.5%
	**75 plus**	2,576,241	4,823	0.2%	11,445	0.4%
**Race-ethnicity**	**White**	8,657,702	19,962	0.2%	36,053	0.4%
	**Black**	1,242,477	2,035	0.2%	3,814	0.3%
	**Hispanic**	1,493,457	2,143	0.1%	3,916	0.3%
	**Asian**	607,284	828	0.1%	1,369	0.2%
	**Unknown**	1,719,014	1,883	0.1%	3,327	0.2%
**Sex**	**Female**	7,199,363	14,240	0.2%	26,375	0.4%
	**Male**	6,520,571	12,611	0.2%	22,104	0.3%
**Region**	**Northeast**	1,526,275	3,787	0.2%	6,600	0.4%
	**Midwest**	3,322,846	6,812	0.2%	11,922	0.4%
	**South**	5,619,435	10,843	0.2%	19,811	0.4%
	**West**	3,251,378	5,409	0.2%	10,146	0.3%
**Total**		13,719,934	26,851	0.2%	48,479	0.4%

Source: De-identified Optum Clinformatic ^®^ Data Mart.

Enrollees with full enrollment in a commercial health plan or Medicare during the year.

**Table 90:** Ulcerative Colitis: Ambulatory care visits with first-listed and all-listed diagnoses by age, race-ethnicity, sex and region among privately insured enrollees, 2020

Demographic Characteristics		First-Listed Diagnosis		All-Listed Diagnoses	
		Number of events	Event rate per 100,000	Number of events	Event rate per 100,000
**Age (years)**	**0 to 11**	215	20	318	29
	**12 to 24**	3,718	256	5,202	358
	**25 to 44**	12,849	484	19,957	751
	**45 to 54**	6,578	453	12,739	877
	**55 to 64**	7,490	457	16,090	981
	**65 to 74**	11,765	414	27,889	982
	**75 plus**	8,109	315	23,147	898
**Race-ethnicity**	**White**	37,168	429	77,927	900
	**Black**	3,737	301	8,049	648
	**Hispanic**	4,373	293	8,916	597
	**Asian**	1,728	285	3,145	518
	**Unknown**	3,718	216	7,305	425
**Sex**	**Female**	26,327	366	56,748	788
	**Male**	24,397	374	48,594	745
**Region**	**Northeast**	7,219	473	14,747	966
	**Midwest**	11,940	359	24,443	736
	**South**	21,257	378	43,552	775
	**West**	10,308	317	22,600	695
**Total**		50,724	370	105,342	768

Source: De-identified Optum Clinformatic ^®^ Data Mart.

Enrollees with full enrollment in a commercial health plan or Medicare during the year.

**Table 91:** Ulcerative Colitis: Emergency department visits with first-listed and all-listed diagnoses by age, race-ethnicity, sex and region among privately insured enrollees, 2020

Demographic Characteristics		First-Listed Diagnosis		All-Listed Diagnoses	
		Number of events	Event rate per 100,000	Number of events	Event rate per 100,000
**Age (years)**	**0 to 11**	2	0	2	0
	**12 to 24**	34	2	63	4
	**25 to 44**	232	9	444	17
	**45 to 54**	89	6	157	11
	**55 to 64**	138	8	261	16
	**65 to 74**	249	9	511	18
	**75 plus**	278	11	628	24
**Race-ethnicity**	**White**	727	8	1,470	17
	**Black**	98	8	191	15
	**Hispanic**	86	6	181	12
	**Asian**	24	4	57	9
	**Unknown**	87	5	167	10
**Sex**	**Female**	660	9	1,343	19
	**Male**	362	6	723	11
**Region**	**Northeast**	177	12	310	20
	**Midwest**	228	7	436	13
	**South**	436	8	918	16
	**West**	181	6	402	12
**Total**		1,022	7	2,066	15

Source: De-identified Optum Clinformatic ^®^ Data Mart.

Enrollees with full enrollment in a commercial health plan or Medicare during the year.

**Table 92:** Ulcerative Colitis: Hospital discharges with first-listed and all-listed diagnoses by age, race-ethnicity, sex and region among privately insured enrollees, 2020

Demographic Characteristics		First-Listed Diagnosis		All-Listed Diagnoses	
		Number of events	Event rate per 100,000	Number of events	Event rate per 100,000
**Age (years)**	**0 to 11**	16	1	24	2
	**12 to 24**	125	9	246	17
	**25 to 44**	241	9	873	33
	**45 to 54**	126	9	529	36
	**55 to 64**	181	11	1,064	65
	**65 to 74**	332	12	2,174	77
	**75 plus**	327	13	2,738	106
**Race-ethnicity**	**White**	1,000	12	5,632	65
	**Black**	126	10	724	58
	**Hispanic**	100	7	588	39
	**Asian**	25	4	140	23
	**Unknown**	97	6	564	33
**Sex**	**Female**	789	11	4,382	61
	**Male**	559	9	3,266	50
**Region**	**Northeast**	198	13	1,130	74
	**Midwest**	323	10	1,834	55
	**South**	598	11	3,235	58
	**West**	229	7	1,449	45
**Total**		1,348	10	7,648	56

Source: De-identified Optum Clinformatic ^®^ Data Mart.

Enrollees with full enrollment in a commercial health plan or Medicare during the year.

**Table 93:** Ulcerative Colitis: Claims-based prevalence with first-listed and all-listed diagnoses by age, race, sex and region among fee-for-service, age-eligible Medicare beneficiaries, 2019

Demographic Characteristics		Number of enrollees	First-Listed Diagnosis		All-Listed Diagnoses	
			Number of enrollees with disease	Percent of enrollees with disease	Number of enrollees with disease	Percent of enrollees with disease
**Age (years)**	**65 to 69**	8,066,320	24,820	0.3%	45,580	0.6%
	**70 to 74**	6,936,940	22,580	0.3%	43,040	0.6%
	**75 to 79**	4,894,060	15,320	0.3%	30,560	0.6%
	**80 to 84**	3,335,340	8,620	0.3%	17,900	0.5%
	**85+**	3,578,120	6,700	0.2%	15,300	0.4%
**Race**	**White**	22,023,800	68,360	0.3%	133,360	0.6%
	**Black**	1,861,660	3,260	0.2%	6,920	0.4%
	**Other**	2,925,320	6,420	0.2%	12,100	0.4%
**Sex**	**Female**	14,975,560	43,160	0.3%	85,840	0.6%
	**Male**	11,835,220	34,880	0.3%	66,540	0.6%
**Region**	**Northeast**	4,748,700	17,900	0.4%	34,100	0.7%
	**Midwest**	6,069,800	16,480	0.3%	33,840	0.6%
	**South**	10,595,900	29,640	0.3%	58,060	0.5%
	**West**	5,396,380	14,020	0.3%	26,380	0.5%
**Total**		26,810,780	78,040	0.3%	152,380	0.6%

Source: Centers for Medicare & Medicaid Services, 5% Denominator and Claims Files.

Beneficiaries aged 65+ years with continuous and full Parts A and B enrollment and no Health Maintenance Organization enrollment during the year.

Unweighted counts were multiplied by 20 to produce counts for this table.

**Table 94:** Ulcerative Colitis: Ambulatory care visits with first-listed and all-listed diagnoses by age, race, sex and region among fee-for-service, age-eligible Medicare beneficiaries, 2019

Demographic Characteristics		First-Listed Diagnosis		All-Listed Diagnoses	
		Number of events	Event rate per 100,000	Number of events	Event rate per 100,000
**Age (years)**	**65 to 69**	52,380	649	110,540	1,370
	**70 to 74**	47,280	682	104,680	1,509
	**75 to 79**	31,500	644	71,660	1,464
	**80 to 84**	16,420	492	42,040	1,260
	**85+**	11,820	330	34,120	954
**Race**	**White**	140,240	637	320,340	1,455
	**Black**	5,940	319	14,800	795
	**Other**	13,220	452	27,900	954
**Sex**	**Female**	84,080	561	199,000	1,329
	**Male**	75,320	636	164,040	1,386
**Region**	**Northeast**	36,380	766	82,580	1,739
	**Midwest**	31,420	518	73,800	1,216
	**South**	64,120	605	144,140	1,360
	**West**	27,480	509	62,520	1,159
**Total**		159,400	595	363,040	1,354

Source: Centers for Medicare & Medicaid Services, 5% Denominator and Claims Files.

Beneficiaries aged 65+ years with continuous and full Parts A and B enrollment and no Health Maintenance Organization enrollment during the year.

Unweighted counts were multiplied by 20 to produce counts for this table.

**Table 95:** Ulcerative Colitis: Emergency department visits with first-listed and all-listed diagnoses by age, race, sex and region among fee-for-service, age-eligible Medicare beneficiaries, 2019

Demographic Characteristics		First-Listed Diagnosis		All-Listed Diagnoses	
		Number of events	Event rate per 100,000	Number of events	Event rate per 100,000
**Age (years)**	**65 to 69**	1,900	24	7,820	97
	**70 to 74**	1,760	25	7,500	108
	**75 to 79**	1,440	29	6,980	143
	**80 to 84**	1,180	35	5,680	170
	**85+**	1,160	32	6,420	179
**Race**	**White**	6,400	29	30,080	137
	**Black**	360	19	1,820	98
	**Other**	680	23	2,500	85
**Sex**	**Female**	4,820	32	20,520	137
	**Male**	2,620	22	13,880	117
**Region**	**Northeast**	1,860	39	8,900	187
	**Midwest**	1,620	27	7,800	129
	**South**	2,740	26	12,020	113
	**West**	1,220	23	5,680	105
**Total**		7,440	28	34,400	128

Source: Centers for Medicare & Medicaid Services, 5% Denominator and Claims Files.

Beneficiaries aged 65+ years with continuous and full Parts A and B enrollment and no Health Maintenance Organization enrollment during the year.

Unweighted counts were multiplied by 20 to produce counts for this table.

**Table 96:** Ulcerative Colitis: Hospital discharges with first-listed and all-listed diagnoses by age, race, sex and region among fee-for-service, age-eligible Medicare beneficiaries, 2019

Demographic Characteristics		First-Listed Diagnosis		All-Listed Diagnoses	
		Number of events	Event rate per 100,000	Number of events	Event rate per 100,000
**Age (years)**	**65 to 69**	1,240	15	7,300	90
	**70 to 74**	860	12	6,420	93
	**75 to 79**	920	19	6,900	141
	**80 to 84**	740	22	5,080	152
	**85+**	660	18	5,380	150
**Race**	**White**	3,800	17	27,340	124
	**Black**	300	16	1,560	84
	**Other**	320	11	2,180	75
**Sex**	**Female**	2,720	18	17,840	119
	**Male**	1,700	14	13,240	112
**Region**	**Northeast**	1,000	21	7,440	157
	**Midwest**	1,280	21	8,100	133
	**South**	1,560	15	10,640	100
	**West**	580	11	4,900	91
**Total**		4,420	16	31,080	116

Source: Centers for Medicare & Medicaid Services, 5% Denominator and Claims Files.

Beneficiaries aged 65+ years with continuous and full Parts A and B enrollment and no Health Maintenance Organization enrollment during the year.

Unweighted counts were multiplied by 20 to produce counts for this table.

**Table 97:** Diverticular Disease: Ambulatory care visits with first-listed and all-listed diagnoses by age, race, ethnicity, and sex in the United States, 2015

Demographic Characteristics		First-Listed Diagnosis		All-Listed Diagnoses	
		Number in thousands	Rate per 100,000	Number in thousands	Rate per 100,000
**Age (years)**	**0 to 11**	.	.	.	.
	**12 to 24**	3	6	6	11
	**25 to 44**	71	84	229	271
	**45 to 54**	218	507	482	1,119
	**55 to 64**	609	1,492	1,157	2,836
	**65 to 74**	458	1,666	985	3,579
	**75 plus**	289	1,430	721	3,567
**Race**	**White**	1,365	415	3,184	1,006
	**Black**	250	494	313	645
	**Other**	33	115	85	331
**Ethnicity**	**Hispanic**	266	633	529	1,485
	**Not Hispanic**	1,382	383	3,052	888
**Sex**	**Female**	1,077	498	2,232	1,074
	**Male**	572	311	1,349	765
**Total**		1,649	412	3,581	936

Source: National Ambulatory Medical Care Survey (3-year average, 2014–2016).

Rates for age groups are unadjusted and all other rates are age-adjusted.

**Table 98:** Diverticular Disease: Emergency department visits with first-listed and all-listed diagnoses by age and sex in the United States, 2018

Demographic Characteristics		First-Listed Diagnosis		All-Listed Diagnoses	
		Number in thousands	Rate per 100,000	Number in thousands	Rate per 100,000
**Age (years)**	**0 to 11**	0	0	0	0
	**12 to 24**	4	7	8	14
	**25 to 44**	99	113	165	189
	**45 to 54**	114	274	213	512
	**55 to 64**	130	308	290	686
	**65 to 74**	119	391	329	1,079
	**75 plus**	131	596	471	2,149
**Sex**	**Female**	344	173	844	409
	**Male**	253	151	632	369
**Total**		597	163	1,476	391

Source: Healthcare Cost and Utilization Project Nationwide Emergency Department Sample.

Rates for age groups are unadjusted and all other rates are age-adjusted.

**Table 99:** Diverticular Disease: Hospital discharges with first-listed and all-listed diagnoses by age, race, ethnicity, and sex in the United States, 2018

Demographic Characteristics		First-Listed Diagnosis		All-Listed Diagnoses	
		Number in thousands	Rate per 100,000	Number in thousands	Rate per 100,000
**Age (years)**	**0 to 11**	0	0	0	0
	**12 to 24**	1	2	2	3
	**25 to 44**	34	39	57	65
	**45 to 54**	44	106	90	217
	**55 to 64**	61	144	160	378
	**65 to 74**	64	211	218	716
	**75 plus**	89	407	353	1,608
**Race**	**White**	248	81	745	231
	**Black**	37	90	106	260
	**Other**	13	51	39	164
**Ethnicity**	**Hispanic**	31	71	91	225
	**Not Hispanic**	266	80	800	230
**Sex**	**Female**	165	79	492	228
	**Male**	129	76	387	223
**Total**		294	78	880	227

Source: Healthcare Cost and Utilization Project National Inpatient Sample.

Rates for age groups are unadjusted and all other rates are age-adjusted.

**Table 100:** Diverticular Disease: Deaths with underlying or underlying/other cause and lifetime years of life lost by age, race, ethnicity, and sex in the United States, 2019

Demographic Characteristics		Underlying Cause			Underlying/Other Cause		
		Number of Deaths	Rate per 100,000	Years of potential life lost in thousands	Number of Deaths	Rate per 100,000	Years of potential life lost in thousands
**Age (years)**	**0 to 11**	1	0.0	0.1	1	0.0	0.1
	**12 to 24**	.	.	.	.	.	.
	**25 to 44**	24	0.0	1.0	42	0.0	1.7
	**45 to 54**	72	0.2	2.2	127	0.3	3.9
	**55 to 64**	276	0.7	6.3	445	1.0	10.2
	**65 to 74**	532	1.7	8.4	908	2.9	14.3
	**75 plus**	2,014	8.9	13.6	3,262	14.4	22.0
**Race**	**White**	2,616	0.8	27.7	4,283	1.2	45.9
	**Black**	234	0.6	3.2	385	1.0	4.9
	**Other**	69	0.3	0.8	117	0.5	1.3
**Ethnicity**	**Hispanic**	176	0.5	2.5	299	0.8	4.2
	**Not Hispanic**	2,743	0.7	29.1	4,486	1.2	48.0
**Sex**	**Female**	1,932	0.8	20.4	3,043	1.3	32.0
	**Male**	987	0.6	11.2	1,742	1.0	20.2
**Total**		2,919	0.7	31.6	4,785	1.2	52.1

Source: Vital Statistics of the United States.

Rates for age groups are unadjusted and all other rates are age-adjusted.

Years of potential life lost is based on U.S. life Table 2006–2018 estimates of life expectancy at age of death.

**Table 101:** Diverticular Disease: Claims-based prevalence with first-listed and all-listed diagnoses by age, race-ethnicity, sex and region among privately insured enrollees, 2020

Demographic Characteristics		Number of enrollees	First-Listed Diagnosis		All-Listed Diagnoses	
			Number of enrollees with disease	Percent of enrollees with disease	Number of enrollees with disease	Percent of enrollees with disease
**Age (years)**	**0 to 11**	1,101,978	3	0.0%	5	0.0%
	**12 to 24**	1,451,892	105	0.0%	237	0.0%
	**25 to 44**	2,656,672	4,503	0.2%	9,600	0.4%
	**45 to 54**	1,452,320	7,584	0.5%	32,872	2.3%
	**55 to 64**	1,640,216	13,447	0.8%	69,544	4.2%
	**65 to 74**	2,840,615	30,538	1.1%	163,451	5.8%
	**75 plus**	2,576,241	27,365	1.1%	122,218	4.7%
**Race-ethnicity**	**White**	8,657,702	57,637	0.7%	282,746	3.3%
	**Black**	1,242,477	9,319	0.8%	41,745	3.4%
	**Hispanic**	1,493,457	9,900	0.7%	40,954	2.7%
	**Asian**	607,284	1,439	0.2%	7,557	1.2%
	**Unknown**	1,719,014	5,250	0.3%	24,925	1.4%
**Sex**	**Female**	7,199,363	50,411	0.7%	219,713	3.1%
	**Male**	6,520,571	33,134	0.5%	178,214	2.7%
**Region**	**Northeast**	1,526,275	10,640	0.7%	52,947	3.5%
	**Midwest**	3,322,846	16,723	0.5%	88,908	2.7%
	**South**	5,619,435	39,790	0.7%	179,935	3.2%
	**West**	3,251,378	16,392	0.5%	76,137	2.3%
**Total**		13,719,934	83,545	0.6%	397,927	2.9%

Source: De-identified Optum Clinformatic ^®^ Data Mart.

Enrollees with full enrollment in a commercial health plan or Medicare during the year.

**Table 102:** Diverticular Disease: Ambulatory care visits with first-listed and all-listed diagnoses by age, race-ethnicity, sex and region among privately insured enrollees, 2020

Demographic Characteristics		First-Listed Diagnosis		All-Listed Diagnoses	
		Number of events	Event rate per 100,000	Number of events	Event rate per 100,000
**Age (years)**	**0 to 11**	5	0	6	1
	**12 to 24**	138	10	291	20
	**25 to 44**	7,493	282	15,481	583
	**45 to 54**	11,583	798	44,056	3,033
	**55 to 64**	19,257	1,174	90,523	5,519
	**65 to 74**	39,645	1,396	207,668	7,311
	**75 plus**	33,865	1,315	161,268	6,260
**Race-ethnicity**	**White**	78,162	903	365,328	4,220
	**Black**	11,392	917	54,212	4,363
	**Hispanic**	13,575	909	57,396	3,843
	**Asian**	1,834	302	9,553	1,573
	**Unknown**	7,023	409	32,804	1,908
**Sex**	**Female**	68,669	954	297,017	4,126
	**Male**	43,317	664	222,276	3,409
**Region**	**Northeast**	14,339	939	68,647	4,498
	**Midwest**	22,706	683	109,536	3,296
	**South**	52,688	938	241,917	4,305
	**West**	22,253	684	99,193	3,051
**Total**		111,986	816	519,293	3,785

Source: De-identified Optum Clinformatic ^®^ Data Mart.

Enrollees with full enrollment in a commercial health plan or Medicare during the year.

**Table 103:** Diverticular Disease: Emergency department visits with first-listed and all-listed diagnoses by age, race-ethnicity, sex and region among privately insured enrollees, 2020

Demographic Characteristics		First-Listed Diagnosis		All-Listed Diagnoses	
		Number of events	Event rate per 100,000	Number of events	Event rate per 100,000
**Age (years)**	**0 to 11**	.	.	.	.
	**12 to 24**	2	0	5	0
	**25 to 44**	218	8	444	17
	**45 to 54**	485	33	1,169	80
	**55 to 64**	1,277	78	2,981	182
	**65 to 74**	5,894	207	9,379	330
	**75 plus**	6,125	238	10,503	408
**Race-ethnicity**	**White**	9,583	111	16,888	195
	**Black**	1,659	134	2,816	227
	**Hispanic**	1,639	110	2,792	187
	**Asian**	177	29	357	59
	**Unknown**	943	55	1,628	95
**Sex**	**Female**	9,545	133	15,880	221
	**Male**	4,456	68	8,601	132
**Region**	**Northeast**	2,011	132	3,182	208
	**Midwest**	3,025	91	5,073	153
	**South**	6,073	108	11,059	197
	**West**	2,892	89	5,167	159
**Total**		14,001	102	24,481	178

Source: De-identified Optum Clinformatic ^®^ Data Mart.

Enrollees with full enrollment in a commercial health plan or Medicare during the year.

**Table 104:** Diverticular Disease: Hospital discharges with first-listed and all-listed diagnoses by age, race-ethnicity, sex and region among privately insured enrollees, 2020

Demographic Characteristics		First-Listed Diagnosis		All-Listed Diagnoses	
		Number of events	Event rate per 100,000	Number of events	Event rate per 100,000
**Age (years)**	**0 to 11**	.	.	1	0
	**12 to 24**	4	0	22	2
	**25 to 44**	686	26	1,180	44
	**45 to 54**	1,038	71	2,276	157
	**55 to 64**	1,805	110	5,443	332
	**65 to 74**	4,141	146	15,848	558
	**75 plus**	6,380	248	27,816	1,080
**Race-ethnicity**	**White**	9,550	110	35,948	415
	**Black**	2,045	165	6,972	561
	**Hispanic**	1,416	95	5,495	368
	**Asian**	216	36	738	122
	**Unknown**	827	48	3,433	200
**Sex**	**Female**	8,325	116	30,734	427
	**Male**	5,729	88	21,852	335
**Region**	**Northeast**	1,923	126	7,130	467
	**Midwest**	3,206	96	11,337	341
	**South**	6,374	113	24,165	430
	**West**	2,551	78	9,954	306
**Total**		14,054	102	52,586	383

Source: De-identified Optum Clinformatic ^®^ Data Mart.

Enrollees with full enrollment in a commercial health plan or Medicare during the year.

**Table 105:** Diverticular Disease: Claims-based prevalence with first-listed and all-listed diagnoses by age, race, sex and region among fee-for-service, age-eligible Medicare beneficiaries, 2019

Demographic Characteristics		Number of enrollees	First-Listed Diagnosis		All-Listed Diagnoses	
			Number of enrollees with disease	Percent of enrollees with disease	Number of enrollees with disease	Percent of enrollees with disease
**Age (years)**	**65 to 69**	8,066,320	113,280	1.4%	538,180	6.7%
	**70 to 74**	6,936,940	122,180	1.8%	546,660	7.9%
	**75 to 79**	4,894,060	93,540	1.9%	398,100	8.1%
	**80 to 84**	3,335,340	61,800	1.9%	232,840	7.0%
	**85+**	3,578,120	55,400	1.5%	196,700	5.5%
**Race**	**White**	22,023,800	375,600	1.7%	1,624,300	7.4%
	**Black**	1,861,660	30,260	1.6%	121,680	6.5%
	**Other**	2,925,320	40,340	1.4%	166,500	5.7%
**Sex**	**Female**	14,975,560	281,640	1.9%	1,088,100	7.3%
	**Male**	11,835,220	164,560	1.4%	824,380	7.0%
**Region**	**Northeast**	4,748,700	86,540	1.8%	367,820	7.7%
	**Midwest**	6,069,800	83,660	1.4%	404,820	6.7%
	**South**	10,595,900	193,460	1.8%	822,700	7.8%
	**West**	5,396,380	82,540	1.5%	317,140	5.9%
**Total**		26,810,780	446,200	1.7%	1,912,480	7.1%

Source: Centers for Medicare & Medicaid Services, 5% Denominator and Claims Files.

Beneficiaries aged 65+ years with continuous and full Parts A and B enrollment and no Health Maintenance Organization enrollment during the year.

Unweighted counts were multiplied by 20 to produce counts for this table.

**Table 106:** Diverticular Disease: Ambulatory care visits with first-listed and all-listed diagnoses by age, race, sex and region among fee-for-service, age-eligible Medicare beneficiaries, 2019

Demographic Characteristics		First-Listed Diagnosis		All-Listed Diagnoses	
		Number of events	Event rate per 100,000	Number of events	Event rate per 100,000
**Age (years)**	**65 to 69**	134,900	1,672	675,960	8,380
	**70 to 74**	143,540	2,069	680,380	9,808
	**75 to 79**	101,580	2,076	487,080	9,952
	**80 to 84**	63,460	1,903	268,120	8,039
	**85+**	45,900	1,283	190,400	5,321
**Race**	**White**	417,160	1,894	1,951,200	8,860
	**Black**	28,760	1,545	141,860	7,620
	**Other**	43,460	1,486	208,880	7,140
**Sex**	**Female**	321,180	2,145	1,361,240	9,090
	**Male**	168,200	1,421	940,700	7,948
**Region**	**Northeast**	97,220	2,047	449,880	9,474
	**Midwest**	89,240	1,470	453,980	7,479
	**South**	210,800	1,989	1,018,120	9,609
	**West**	92,120	1,707	379,960	7,041
**Total**		489,380	1,825	2,301,940	8,586

Source: Centers for Medicare & Medicaid Services, 5% Denominator and Claims Files.

Beneficiaries aged 65+ years with continuous and full Parts A and B enrollment and no Health Maintenance Organization enrollment during the year.

Unweighted counts were multiplied by 20 to produce counts for this table.

**Table 107:** Diverticular Disease: Emergency department visits with first-listed and all-listed diagnoses by age, race, sex and region among fee-for-service, age-eligible Medicare beneficiaries, 2019

Demographic Characteristics		First-Listed Diagnosis		All-Listed Diagnoses	
		Number of events	Event rate per 100,000	Number of events	Event rate per 100,000
**Age (years)**	**65 to 69**	40,420	501	96,900	1,201
	**70 to 74**	43,380	625	116,860	1,685
	**75 to 79**	38,260	782	111,600	2,280
	**80 to 84**	29,660	889	96,780	2,902
	**85+**	37,200	1,040	129,400	3,616
**Race**	**White**	155,900	708	457,240	2,076
	**Black**	16,760	900	47,780	2,567
	**Other**	16,260	556	46,520	1,590
**Sex**	**Female**	123,060	822	338,580	2,261
	**Male**	65,860	556	212,960	1,799
**Region**	**Northeast**	36,860	776	102,940	2,168
	**Midwest**	39,420	649	117,540	1,936
	**South**	81,100	765	237,100	2,238
	**West**	31,540	584	93,960	1,741
**Total**		188,920	705	551,540	2,057

Source: Centers for Medicare & Medicaid Services, 5% Denominator and Claims Files.

Beneficiaries aged 65+ years with continuous and full Parts A and B enrollment and no Health Maintenance Organization enrollment during the year.

Unweighted counts were multiplied by 20 to produce counts for this table.

**Table 108:** Diverticular Disease: Hospital discharges with first-listed and all-listed diagnoses by age, race, sex and region among fee-for-service, age-eligible Medicare beneficiaries, 2019

Demographic Characteristics		First-Listed Diagnosis		All-Listed Diagnoses	
		Number of events	Event rate per 100,000	Number of events	Event rate per 100,000
**Age (years)**	**65 to 69**	16,020	199	50,320	624
	**70 to 74**	18,800	271	66,780	963
	**75 to 79**	18,060	369	68,000	1,389
	**80 to 84**	14,980	449	60,960	1,828
	**85+**	20,520	573	81,380	2,274
**Race**	**White**	71,840	326	271,200	1,231
	**Black**	9,940	534	29,520	1,586
	**Other**	6,600	226	26,720	913
**Sex**	**Female**	56,140	375	197,740	1,320
	**Male**	32,240	272	129,700	1,096
**Region**	**Northeast**	17,560	370	61,480	1,295
	**Midwest**	20,160	332	75,940	1,251
	**South**	38,360	362	138,440	1,307
	**West**	12,300	228	51,580	956
**Total**		88,380	330	327,440	1,221

Source: Centers for Medicare & Medicaid Services, 5% Denominator and Claims Files.

Beneficiaries aged 65+ years with continuous and full Parts A and B enrollment and no Health Maintenance Organization enrollment during the year.

Unweighted counts were multiplied by 20 to produce counts for this table.

**Table 109:** Hemorrhoids: Ambulatory care visits with first-listed and all-listed diagnoses by age, race, ethnicity, and sex in the United States, 2015

Demographic Characteristics		First-Listed Diagnosis		All-Listed Diagnoses	
		Number in thousands	Rate per 100,000	Number in thousands	Rate per 100,000
**Age (years)**	**0 to 11**	.	.	4	8
	**12 to 24**	81	144	112	200
	**25 to 44**	421	498	888	1,051
	**45 to 54**	546	1,267	1,079	2,505
	**55 to 64**	418	1,025	789	1,933
	**65 to 74**	306	1,111	660	2,397
	**75 plus**	215	1,063	504	2,492
**Race**	**White**	1,426	522	2,948	1,054
	**Black**	200	452	619	1,392
	**Other**	360	1,503	469	1,966
**Ethnicity**	**Hispanic**	561	1,217	839	1,787
	**Not Hispanic**	1,425	483	3,197	1,064
**Sex**	**Female**	900	503	2,052	1,158
	**Male**	1,086	672	1,983	1,195
**Total**		1,986	584	4,036	1,176

Source: National Ambulatory Medical Care Survey (3-year average, 2014–2016).

Rates for age groups are unadjusted and all other rates are age-adjusted.

**Table 110:** Hemorrhoids: Emergency department visits with first-listed and all-listed diagnoses by age and sex in the United States, 2018

Demographic Characteristics		First-Listed Diagnosis		All-Listed Diagnoses	
		Number in thousands	Rate per 100,000	Number in thousands	Rate per 100,000
**Age (years)**	**0 to 11**	1	2	2	4
	**12 to 24**	23	41	36	65
	**25 to 44**	77	89	140	161
	**45 to 54**	30	72	81	194
	**55 to 64**	22	52	94	221
	**65 to 74**	17	55	100	329
	**75 plus**	23	106	149	681
**Sex**	**Female**	91	53	316	166
	**Male**	102	64	287	170
**Total**		193	58	603	169

Source: Healthcare Cost and Utilization Project Nationwide Emergency Department Sample.

Rates for age groups are unadjusted and all other rates are age-adjusted.

**Table 111:** Hemorrhoids: Hospital discharges with first-listed and all-listed diagnoses by age, race, ethnicity, and sex in the United States, 2018

Demographic Characteristics		First-Listed Diagnosis		All-Listed Diagnoses	
		Number in thousands	Rate per 100,000	Number in thousands	Rate per 100,000
**Age (years)**	**0 to 11**	0	0	1	1
	**12 to 24**	0	0	4	8
	**25 to 44**	4	4	35	40
	**45 to 54**	3	8	40	95
	**55 to 64**	4	10	65	153
	**65 to 74**	5	15	82	268
	**75 plus**	9	40	121	552
**Race**	**White**	19	6	270	86
	**Black**	5	11	58	137
	**Other**	2	9	24	97
**Ethnicity**	**Hispanic**	3	8	40	96
	**Not Hispanic**	22	7	311	92
**Sex**	**Female**	13	6	184	89
	**Male**	12	7	163	93
**Total**		25	7	347	91

Source: Healthcare Cost and Utilization Project National Inpatient Sample.

Rates for age groups are unadjusted and all other rates are age-adjusted.

**Table 112:** Hemorrhoids: Deaths with underlying or underlying/other cause and lifetime years of life lost by age, race, ethnicity, and sex in the United States, 2019

Demographic Characteristics		Underlying Cause			Underlying/Other Cause		
		Number of Deaths	Rate per 100,000	Years of potential life lost in thousands	Number of Deaths	Rate per 100,000	Years of potential life lost in thousands
**Age (years)**	**0 to 11**	.	.	.	.	.	.
	**12 to 24**	.	.	.	.	.	.
	**25 to 44**	4	0.0	0.2	7	0.0	0.3
	**45 to 54**	1	0.0	0.0	5	0.0	0.1
	**55 to 64**	2	0.0	0.0	20	0.0	0.5
	**65 to 74**	6	0.0	0.1	18	0.1	0.3
	**75 plus**	11	0.0	0.1	41	0.2	0.2
**Race**	**White**	19	0.0	0.3	69	0.0	1.0
	**Black**	2	0.0	0.0	12	0.0	0.2
	**Other**	3	0.0	0.1	10	0.0	0.2
**Ethnicity**	**Hispanic**	4	0.0	0.1	11	0.0	0.2
	**Not Hispanic**	20	0.0	0.3	80	0.0	1.2
**Sex**	**Female**	11	0.0	0.2	47	0.0	0.7
	**Male**	13	0.0	0.2	44	0.0	0.7
**Total**		24	0.0	0.4	91	0.0	1.4

Source: Vital Statistics of the United States.

Rates for age groups are unadjusted and all other rates are age-adjusted.

Years of potential life lost is based on U.S. life Table 2006–2018 estimates of life expectancy at age of death.

**Table 113:** Hemorrhoids: Claims-based prevalence with first-listed and all-listed diagnoses by age, race-ethnicity, sex and region among privately insured enrollees, 2020

Demographic Characteristics		Number of enrollees	First-Listed Diagnosis		All-Listed Diagnoses	
			Number of enrollees with disease	Percent of enrollees with disease	Number of enrollees with disease	Percent of enrollees with disease
**Age (years)**	**0 to 11**	1,101,978	188	0.0%	545	0.0%
	**12 to 24**	1,451,892	2,079	0.1%	4,756	0.3%
	**25 to 44**	2,656,672	14,761	0.6%	36,906	1.4%
	**45 to 54**	1,452,320	9,720	0.7%	50,138	3.5%
	**55 to 64**	1,640,216	10,428	0.6%	70,544	4.3%
	**65 to 74**	2,840,615	16,936	0.6%	130,407	4.6%
	**75 plus**	2,576,241	13,195	0.5%	76,797	3.0%
**Race-ethnicity**	**White**	8,657,702	43,330	0.5%	249,109	2.9%
	**Black**	1,242,477	6,838	0.6%	37,975	3.1%
	**Hispanic**	1,493,457	8,416	0.6%	42,185	2.8%
	**Asian**	607,284	3,201	0.5%	14,031	2.3%
	**Unknown**	1,719,014	5,522	0.3%	26,793	1.6%
**Sex**	**Female**	7,199,363	35,481	0.5%	199,197	2.8%
	**Male**	6,520,571	31,826	0.5%	170,896	2.6%
**Region**	**Northeast**	1,526,275	8,670	0.6%	49,981	3.3%
	**Midwest**	3,322,846	13,463	0.4%	81,198	2.4%
	**South**	5,619,435	29,700	0.5%	160,730	2.9%
	**West**	3,251,378	15,474	0.5%	78,184	2.4%
**Total**		13,719,934	67,307	0.5%	370,093	2.7%

Source: De-identified Optum Clinformatic ^®^ Data Mart.

Enrollees with full enrollment in a commercial health plan or Medicare during the year.

**Table 114:** Hemorrhoids: Ambulatory care visits with first-listed and all-listed diagnoses by age, race-ethnicity, sex and region among privately insured enrollees, 2020

Demographic Characteristics		First-Listed Diagnosis		All-Listed Diagnoses	
		Number of events	Event rate per 100,000	Number of events	Event rate per 100,000
**Age (years)**	**0 to 11**	210	19	622	56
	**12 to 24**	2,522	174	6,006	414
	**25 to 44**	20,127	758	50,760	1,911
	**45 to 54**	13,584	935	62,938	4,334
	**55 to 64**	14,470	882	85,964	5,241
	**65 to 74**	23,232	818	153,968	5,420
	**75 plus**	17,528	680	91,249	3,542
**Race-ethnicity**	**White**	59,473	687	299,544	3,460
	**Black**	9,000	724	46,033	3,705
	**Hispanic**	11,336	759	54,596	3,656
	**Asian**	4,478	737	18,241	3,004
	**Unknown**	7,386	430	33,093	1,925
**Sex**	**Female**	47,481	660	242,347	3,366
	**Male**	44,192	678	209,160	3,208
**Region**	**Northeast**	11,352	744	59,390	3,891
	**Midwest**	17,992	541	93,257	2,807
	**South**	41,273	734	202,711	3,607
	**West**	21,056	648	96,149	2,957
**Total**		91,673	668	451,507	3,291

Source: De-identified Optum Clinformatic ^®^ Data Mart.

Enrollees with full enrollment in a commercial health plan or Medicare during the year.

**Table 115:** Hemorrhoids: Emergency department visits with first-listed and all-listed diagnoses by age, race-ethnicity, sex and region among privately insured enrollees, 2020

Demographic Characteristics		First-Listed Diagnosis		All-Listed Diagnoses	
		Number of events	Event rate per 100,000	Number of events	Event rate per 100,000
**Age (years)**	**0 to 11**	.	.	.	.
	**12 to 24**	29	2	110	8
	**25 to 44**	345	13	1,135	43
	**45 to 54**	231	16	1,098	76
	**55 to 64**	346	21	1,600	98
	**65 to 74**	665	23	1,470	52
	**75 plus**	925	36	1,990	77
**Race-ethnicity**	**White**	1,551	18	4,879	56
	**Black**	399	32	946	76
	**Hispanic**	305	20	778	52
	**Asian**	66	11	232	38
	**Unknown**	220	13	568	33
**Sex**	**Female**	1,459	20	4,222	59
	**Male**	1,082	17	3,181	49
**Region**	**Northeast**	362	24	1,030	67
	**Midwest**	513	15	1,602	48
	**South**	1,216	22	3,483	62
	**West**	450	14	1,288	40
**Total**		2,541	19	7,403	54

Source: De-identified Optum Clinformatic ^®^ Data Mart.

Enrollees with full enrollment in a commercial health plan or Medicare during the year.

**Table 116:** Hemorrhoids: Hospital discharges with first-listed and all-listed diagnoses by age, race-ethnicity, sex and region among privately insured enrollees, 2020

Demographic Characteristics		First-Listed Diagnosis		All-Listed Diagnoses	
		Number of events	Event rate per 100,000	Number of events	Event rate per 100,000
**Age (years)**	**0 to 11**	.	.	18	2
	**12 to 24**	2	0	82	6
	**25 to 44**	45	2	648	24
	**45 to 54**	65	4	909	63
	**55 to 64**	91	6	2,225	136
	**65 to 74**	264	9	6,183	218
	**75 plus**	516	20	10,050	390
**Race-ethnicity**	**White**	600	7	13,006	150
	**Black**	154	12	2,988	240
	**Hispanic**	142	10	2,209	148
	**Asian**	28	5	454	75
	**Unknown**	59	3	1,458	85
**Sex**	**Female**	507	7	11,142	155
	**Male**	476	7	8,973	138
**Region**	**Northeast**	156	10	2,860	187
	**Midwest**	189	6	4,323	130
	**South**	427	8	9,160	163
	**West**	211	6	3,772	116
**Total**		983	7	20,115	147

Source: De-identified Optum Clinformatic ^®^ Data Mart.

Enrollees with full enrollment in a commercial health plan or Medicare during the year.

**Table 117:** Hemorrhoids: Claims-based prevalence with first-listed and all-listed diagnoses by age, race, sex and region among fee-for-service, age-eligible Medicare beneficiaries, 2019

Demographic Characteristics		Number of enrollees	First-Listed Diagnosis		All-Listed Diagnoses	
			Number of enrollees with disease	Percent of enrollees with disease	Number of enrollees with disease	Percent of enrollees with disease
**Age (years)**	**65 to 69**	8,066,320	60,360	0.7%	371,360	4.6%
	**70 to 74**	6,936,940	54,280	0.8%	328,060	4.7%
	**75 to 79**	4,894,060	39,600	0.8%	214,980	4.4%
	**80 to 84**	3,335,340	27,400	0.8%	116,980	3.5%
	**85+**	3,578,120	27,880	0.8%	94,840	2.7%
**Race**	**White**	22,023,800	168,740	0.8%	912,080	4.1%
	**Black**	1,861,660	14,040	0.8%	80,880	4.3%
	**Other**	2,925,320	26,740	0.9%	133,260	4.6%
**Sex**	**Female**	14,975,560	123,040	0.8%	637,020	4.3%
	**Male**	11,835,220	86,480	0.7%	489,200	4.1%
**Region**	**Northeast**	4,748,700	40,860	0.9%	227,920	4.8%
	**Midwest**	6,069,800	38,780	0.6%	225,160	3.7%
	**South**	10,595,900	83,420	0.8%	460,680	4.3%
	**West**	5,396,380	46,460	0.9%	212,460	3.9%
**Total**		26,810,780	209,520	0.8%	1,126,220	4.2%

Source: Centers for Medicare & Medicaid Services, 5% Denominator and Claims Files.

Beneficiaries aged 65+ years with continuous and full Parts A and B enrollment and no Health Maintenance Organization enrollment during the year.

Unweighted counts were multiplied by 20 to produce counts for this table.

**Table 118:** Hemorrhoids: Ambulatory care visits with first-listed and all-listed diagnoses by age, race, sex and region among fee-for-service, age-eligible Medicare beneficiaries, 2019

Demographic Characteristics		First-Listed Diagnosis		All-Listed Diagnoses	
		Number of events	Event rate per 100,000	Number of events	Event rate per 100,000
**Age (years)**	**65 to 69**	84,720	1,050	452,640	5,611
	**70 to 74**	75,080	1,082	397,100	5,724
	**75 to 79**	52,700	1,077	254,780	5,206
	**80 to 84**	36,140	1,084	140,780	4,221
	**85+**	31,780	888	110,980	3,102
**Race**	**White**	226,420	1,028	1,090,980	4,954
	**Black**	16,600	892	92,220	4,954
	**Other**	37,400	1,278	173,080	5,917
**Sex**	**Female**	160,360	1,071	772,560	5,159
	**Male**	120,060	1,014	583,720	4,932
**Region**	**Northeast**	53,140	1,119	268,380	5,652
	**Midwest**	50,780	837	254,440	4,192
	**South**	114,000	1,076	566,500	5,346
	**West**	62,500	1,158	266,960	4,947
**Total**		280,420	1,046	1,356,280	5,059

Source: Centers for Medicare & Medicaid Services, 5% Denominator and Claims Files.

Beneficiaries aged 65+ years with continuous and full Parts A and B enrollment and no Health Maintenance Organization enrollment during the year.

Unweighted counts were multiplied by 20 to produce counts for this table.

**Table 119:** Hemorrhoids: Emergency department visits with first-listed and all-listed diagnoses by age, race, sex and region among fee-for-service, age-eligible Medicare beneficiaries, 2019

Demographic Characteristics		First-Listed Diagnosis		All-Listed Diagnoses	
		Number of events	Event rate per 100,000	Number of events	Event rate per 100,000
**Age (years)**	**65 to 69**	4,260	53	22,740	282
	**70 to 74**	4,580	66	26,720	385
	**75 to 79**	4,560	93	28,560	584
	**80 to 84**	4,780	143	26,400	792
	**85+**	6,800	190	33,240	929
**Race**	**White**	19,360	88	107,760	489
	**Black**	2,980	160	15,480	832
	**Other**	2,640	90	14,420	493
**Sex**	**Female**	16,200	108	82,140	548
	**Male**	8,780	74	55,520	469
**Region**	**Northeast**	4,840	102	27,600	581
	**Midwest**	5,440	90	29,720	490
	**South**	10,240	97	56,040	529
	**West**	4,460	83	24,300	450
**Total**		24,980	93	137,660	513

Source: Centers for Medicare & Medicaid Services, 5% Denominator and Claims Files.

Beneficiaries aged 65+ years with continuous and full Parts A and B enrollment and no Health Maintenance Organization enrollment during the year.

Unweighted counts were multiplied by 20 to produce counts for this table.

**Table 120:** Hemorrhoids: Hospital discharges with first-listed and all-listed diagnoses by age, race, sex and region among fee-for-service, age-eligible Medicare beneficiaries, 2019

Demographic Characteristics		First-Listed Diagnosis		All-Listed Diagnoses	
		Number of events	Event rate per 100,000	Number of events	Event rate per 100,000
**Age (years)**	**65 to 69**	1,200	15	18,860	234
	**70 to 74**	1,400	20	22,600	326
	**75 to 79**	1,500	31	25,040	512
	**80 to 84**	1,820	55	22,800	684
	**85+**	2,220	62	26,520	741
**Race**	**White**	6,400	29	91,580	416
	**Black**	1,040	56	12,680	681
	**Other**	700	24	11,560	395
**Sex**	**Female**	5,140	34	67,460	450
	**Male**	3,000	25	48,360	409
**Region**	**Northeast**	1,840	39	22,880	482
	**Midwest**	1,780	29	25,580	421
	**South**	3,420	32	48,020	453
	**West**	1,100	20	19,340	358
**Total**		8,140	30	115,820	432

Source: Centers for Medicare & Medicaid Services, 5% Denominator and Claims Files.

Beneficiaries aged 65+ years with continuous and full Parts A and B enrollment and no Health Maintenance Organization enrollment during the year.

Unweighted counts were multiplied by 20 to produce counts for this table.

**Table 121:** Liver Disease: Ambulatory care visits with first-listed and all-listed diagnoses by age, race, ethnicity, and sex in the United States, 2015

Demographic Characteristics		First-Listed Diagnosis		All-Listed Diagnoses	
		Number in thousands	Rate per 100,000	Number in thousands	Rate per 100,000
**Age (years)**	**0 to 11**	.	.	6	12
	**12 to 24**	19	34	88	157
	**25 to 44**	212	251	729	862
	**45 to 54**	356	826	1,111	2,578
	**55 to 64**	214	523	1,174	2,878
	**65 to 74**	145	527	501	1,819
	**75 plus**	195	963	558	2,759
**Race**	**White**	875	332	3,145	1,112
	**Black**	203	466	670	1,602
	**Other**	62	238	351	1,440
**Ethnicity**	**Hispanic**	78	172	530	1,149
	**Not Hispanic**	1,062	366	3,636	1,195
**Sex**	**Female**	627	370	2,441	1,362
	**Male**	513	312	1,725	1,023
**Total**		1,140	340	4,166	1,196

Source: National Ambulatory Medical Care Survey (3-year average, 2014–2016).

Rates for age groups are unadjusted and all other rates are age-adjusted.

**Table 122:** Liver Disease: Emergency department visits with first-listed and all-listed diagnoses by age and sex in the United States, 2018

Demographic Characteristics		First-Listed Diagnosis		All-Listed Diagnoses	
		Number in thousands	Rate per 100,000	Number in thousands	Rate per 100,000
**Age (years)**	**0 to 11**	1	2	7	14
	**12 to 24**	6	11	53	96
	**25 to 44**	58	66	428	492
	**45 to 54**	76	182	453	1,089
	**55 to 64**	99	234	593	1,404
	**65 to 74**	55	181	398	1,305
	**75 plus**	26	117	265	1,208
**Sex**	**Female**	133	70	1,011	536
	**Male**	187	105	1,187	672
**Total**		320	87	2,198	601

Source: Healthcare Cost and Utilization Project Nationwide Emergency Department Sample.

Rates for age groups are unadjusted and all other rates are age-adjusted.

**Table 123:** Liver Disease: Hospital discharges with first-listed and all-listed diagnoses by age, race, ethnicity, and sex in the United States, 2018

Demographic Characteristics		First-Listed Diagnosis		All-Listed Diagnoses	
		Number in thousands	Rate per 100,000	Number in thousands	Rate per 100,000
**Age (years)**	**0 to 11**	1	2	10	20
	**12 to 24**	2	4	29	53
	**25 to 44**	37	42	262	301
	**45 to 54**	56	134	309	741
	**55 to 64**	81	193	460	1,089
	**65 to 74**	52	170	357	1,171
	**75 plus**	24	107	242	1,105
**Race**	**White**	214	71	1,370	455
	**Black**	23	48	202	439
	**Other**	18	68	115	442
**Ethnicity**	**Hispanic**	45	94	252	528
	**Not Hispanic**	210	65	1,435	442
**Sex**	**Female**	105	53	742	381
	**Male**	148	82	928	517
**Total**		253	67	1,670	446

Source: Healthcare Cost and Utilization Project National Inpatient Sample.

Rates for age groups are unadjusted and all other rates are age-adjusted.

**Table 124:** Liver Disease: Deaths with underlying or underlying/other cause and lifetime years of life lost by age, race, ethnicity, and sex in the United States, 2019

Demographic Characteristics		Underlying Cause			Underlying/Other Cause		
		Number of Deaths	Rate per 100,000	Years of potential life lost in thousands	Number of Deaths	Rate per 100,000	Years of potential life lost in thousands
**Age (years)**	**0 to 11**	58	0.1	4.4	284	0.6	21.8
	**12 to 24**	80	0.1	4.7	274	0.5	16.2
	**25 to 44**	5,395	6.2	229.1	8,317	9.5	353.7
	**45 to 54**	9,669	23.7	300.3	15,314	37.5	474.3
	**55 to 64**	17,669	41.6	409.6	31,960	75.3	735.4
	**65 to 74**	14,466	45.9	233.1	29,011	92.1	464.9
	**75 plus**	10,620	47.0	90.7	22,318	98.9	187.9
**Race**	**White**	50,610	15.8	1,102.0	91,676	28.1	1,911.8
	**Black**	4,771	10.1	102.7	10,688	22.6	222.3
	**Other**	2,576	9.6	67.1	5,114	19.3	120.1
**Ethnicity**	**Hispanic**	7,093	15.3	178.7	12,662	27.7	310.3
	**Not Hispanic**	50,864	14.8	1,093.1	94,816	27.0	1,943.9
**Sex**	**Female**	23,008	11.1	521.6	41,890	19.8	914.5
	**Male**	34,949	18.7	750.3	65,588	34.7	1,339.7
**Total**		57,957	14.7	1,271.8	107,478	26.8	2,254.1

Source: Vital Statistics of the United States.

Rates for age groups are unadjusted and all other rates are age-adjusted.

Years of potential life lost is based on U.S. life Table 2006–2018 estimates of life expectancy at age of death.

**Table 125:** Liver Disease: Claims-based prevalence with first-listed and all-listed diagnoses by age, race-ethnicity, sex and region among privately insured enrollees, 2020

Demographic Characteristics		Number of enrollees	First-Listed Diagnosis		All-Listed Diagnoses	
			Number of enrollees with disease	Percent of enrollees with disease	Number of enrollees with disease	Percent of enrollees with disease
**Age (years)**	**0 to 11**	1,101,978	123	0.0%	386	0.0%
	**12 to 24**	1,451,892	852	0.1%	2,888	0.2%
	**25 to 44**	2,656,672	6,458	0.2%	26,241	1.0%
	**45 to 54**	1,452,320	7,930	0.5%	32,265	2.2%
	**55 to 64**	1,640,216	15,046	0.9%	52,772	3.2%
	**65 to 74**	2,840,615	27,402	1.0%	90,425	3.2%
	**75 plus**	2,576,241	14,230	0.6%	55,117	2.1%
**Race-ethnicity**	**White**	8,657,702	44,576	0.5%	160,260	1.9%
	**Black**	1,242,477	6,693	0.5%	25,368	2.0%
	**Hispanic**	1,493,457	12,177	0.8%	42,511	2.8%
	**Asian**	607,284	2,683	0.4%	10,887	1.8%
	**Unknown**	1,719,014	5,912	0.3%	21,068	1.2%
**Sex**	**Female**	7,199,363	39,176	0.5%	137,573	1.9%
	**Male**	6,520,571	32,865	0.5%	122,521	1.9%
**Region**	**Northeast**	1,526,275	8,884	0.6%	30,224	2.0%
	**Midwest**	3,322,846	13,224	0.4%	47,748	1.4%
	**South**	5,619,435	34,360	0.6%	125,392	2.2%
	**West**	3,251,378	15,573	0.5%	56,730	1.7%
**Total**		13,719,934	72,041	0.5%	260,094	1.9%

Source: De-identified Optum Clinformatic ^®^ Data Mart.

Enrollees with full enrollment in a commercial health plan or Medicare during the year.

**Table 126:** Liver Disease: Ambulatory care visits with first-listed and all-listed diagnoses by age, race-ethnicity, sex and region among privately insured enrollees, 2020

Demographic Characteristics		First-Listed Diagnosis		All-Listed Diagnoses	
		Number of events	Event rate per 100,000	Number of events	Event rate per 100,000
**Age (years)**	**0 to 11**	187	17	566	51
	**12 to 24**	1,236	85	4,298	296
	**25 to 44**	10,065	379	42,212	1,589
	**45 to 54**	14,285	984	59,571	4,102
	**55 to 64**	30,013	1,830	109,754	6,691
	**65 to 74**	52,917	1,863	187,580	6,603
	**75 plus**	26,787	1,040	100,440	3,899
**Race-ethnicity**	**White**	85,369	986	309,883	3,579
	**Black**	11,445	921	44,891	3,613
	**Hispanic**	22,988	1,539	88,304	5,913
	**Asian**	4,117	678	19,051	3,137
	**Unknown**	11,571	673	42,292	2,460
**Sex**	**Female**	71,955	999	269,807	3,748
	**Male**	63,535	974	234,614	3,598
**Region**	**Northeast**	16,417	1,076	57,007	3,735
	**Midwest**	25,280	761	88,765	2,671
	**South**	64,350	1,145	247,770	4,409
	**West**	29,443	906	110,879	3,410
**Total**		135,490	988	504,421	3,677

Source: De-identified Optum Clinformatic ^®^ Data Mart.

Enrollees with full enrollment in a commercial health plan or Medicare during the year.

**Table 127:** Liver Disease: Emergency department visits with first-listed and all-listed diagnoses by age, race-ethnicity, sex and region among privately insured enrollees, 2020

Demographic Characteristics		First-Listed Diagnosis		All-Listed Diagnoses	
		Number of events	Event rate per 100,000	Number of events	Event rate per 100,000
**Age (years)**	**0 to 11**	.	.	2	0
	**12 to 24**	4	0	41	3
	**25 to 44**	129	5	492	19
	**45 to 54**	387	27	1,100	76
	**55 to 64**	1,222	75	3,291	201
	**65 to 74**	2,208	78	6,410	226
	**75 plus**	1,146	44	3,909	152
**Race-ethnicity**	**White**	3,031	35	9,169	106
	**Black**	516	42	1,592	128
	**Hispanic**	861	58	2,550	171
	**Asian**	93	15	258	42
	**Unknown**	595	35	1,676	97
**Sex**	**Female**	2,419	34	7,529	105
	**Male**	2,677	41	7,716	118
**Region**	**Northeast**	613	40	1,698	111
	**Midwest**	911	27	2,749	83
	**South**	2,502	45	7,429	132
	**West**	1,070	33	3,369	104
**Total**		5,096	37	15,245	111

Source: De-identified Optum Clinformatic ^®^ Data Mart.

Enrollees with full enrollment in a commercial health plan or Medicare during the year.

**Table 128:** Liver Disease: Hospital discharges with first-listed and all-listed diagnoses by age, race-ethnicity, sex and region among privately insured enrollees, 2020

Demographic Characteristics		First-Listed Diagnosis		All-Listed Diagnoses	
		Number of events	Event rate per 100,000	Number of events	Event rate per 100,000
**Age (years)**	**0 to 11**	17	2	175	16
	**12 to 24**	42	3	550	38
	**25 to 44**	500	19	4,633	174
	**45 to 54**	983	68	7,118	490
	**55 to 64**	2,425	148	16,633	1,014
	**65 to 74**	3,682	130	30,344	1,068
	**75 plus**	1,969	76	24,973	969
**Race-ethnicity**	**White**	6,073	70	53,614	619
	**Black**	897	72	10,366	834
	**Hispanic**	1,543	103	11,083	742
	**Asian**	156	26	1,774	292
	**Unknown**	949	55	7,589	441
**Sex**	**Female**	4,453	62	40,315	560
	**Male**	5,165	79	44,111	676
**Region**	**Northeast**	1,153	76	9,973	653
	**Midwest**	1,936	58	17,470	526
	**South**	4,774	85	40,528	721
	**West**	1,755	54	16,455	506
**Total**		9,618	70	84,426	615

Source: De-identified Optum Clinformatic ^®^ Data Mart.

Enrollees with full enrollment in a commercial health plan or Medicare during the year.

**Table 129:** Liver Disease: Claims-based prevalence with first-listed and all-listed diagnoses by age, race, sex and region among fee-for-service, age-eligible Medicare beneficiaries, 2019

Demographic Characteristics		Number of enrollees	First-Listed Diagnosis		All-Listed Diagnoses	
			Number of enrollees with disease	Percent of enrollees with disease	Number of enrollees with disease	Percent of enrollees with disease
**Age (years)**	**65 to 69**	8,066,320	114,920	1.4%	336,660	4.2%
	**70 to 74**	6,936,940	89,120	1.3%	272,700	3.9%
	**75 to 79**	4,894,060	52,720	1.1%	173,560	3.5%
	**80 to 84**	3,335,340	28,440	0.9%	101,500	3.0%
	**85+**	3,578,120	18,160	0.5%	78,280	2.2%
**Race**	**White**	22,023,800	237,340	1.1%	761,080	3.5%
	**Black**	1,861,660	19,540	1.0%	65,800	3.5%
	**Other**	2,925,320	46,480	1.6%	135,820	4.6%
**Sex**	**Female**	14,975,560	172,140	1.1%	534,800	3.6%
	**Male**	11,835,220	131,220	1.1%	427,900	3.6%
**Region**	**Northeast**	4,748,700	58,360	1.2%	179,960	3.8%
	**Midwest**	6,069,800	56,460	0.9%	183,960	3.0%
	**South**	10,595,900	125,600	1.2%	402,560	3.8%
	**West**	5,396,380	62,940	1.2%	196,220	3.6%
**Total**		26,810,780	303,360	1.1%	962,700	3.6%

Source: Centers for Medicare & Medicaid Services, 5% Denominator and Claims Files.

Beneficiaries aged 65+ years with continuous and full Parts A and B enrollment and no Health Maintenance Organization enrollment during the year.

Unweighted counts were multiplied by 20 to produce counts for this table.

**Table 130:** Liver Disease: Ambulatory care visits with first-listed and all-listed diagnoses by age, race, sex and region among fee-for-service, age-eligible Medicare beneficiaries, 2019

Demographic Characteristics		First-Listed Diagnosis		All-Listed Diagnoses	
		Number of events	Event rate per 100,000	Number of events	Event rate per 100,000
**Age (years)**	**65 to 69**	221,020	2,740	710,440	8,807
	**70 to 74**	165,860	2,391	567,200	8,177
	**75 to 79**	93,380	1,908	324,020	6,621
	**80 to 84**	52,540	1,575	177,080	5,309
	**85+**	28,500	797	105,940	2,961
**Race**	**White**	439,940	1,998	1,470,640	6,678
	**Black**	30,620	1,645	108,720	5,840
	**Other**	90,740	3,102	305,320	10,437
**Sex**	**Female**	314,120	2,098	1,050,940	7,018
	**Male**	247,180	2,089	833,740	7,045
**Region**	**Northeast**	105,260	2,217	354,740	7,470
	**Midwest**	109,520	1,804	354,700	5,844
	**South**	229,600	2,167	786,040	7,418
	**West**	116,920	2,167	389,200	7,212
**Total**		561,300	2,094	1,884,680	7,030

Source: Centers for Medicare & Medicaid Services, 5% Denominator and Claims Files.

Beneficiaries aged 65+ years with continuous and full Parts A and B enrollment and no Health Maintenance Organization enrollment during the year.

Unweighted counts were multiplied by 20 to produce counts for this table.

**Table 131:** Liver Disease: Emergency department visits with first-listed and all-listed diagnoses by age, race, sex and region among fee-for-service, age-eligible Medicare beneficiaries, 2019

Demographic Characteristics		First-Listed Diagnosis		All-Listed Diagnoses	
		Number of events	Event rate per 100,000	Number of events	Event rate per 100,000
**Age (years)**	**65 to 69**	25,540	317	135,220	1,676
	**70 to 74**	16,360	236	109,040	1,572
	**75 to 79**	11,300	231	78,760	1,609
	**80 to 84**	6,900	207	54,220	1,626
	**85+**	5,340	149	50,800	1,420
**Race**	**White**	48,600	221	328,820	1,493
	**Black**	4,380	235	37,980	2,040
	**Other**	12,460	426	61,240	2,093
**Sex**	**Female**	32,980	220	221,960	1,482
	**Male**	32,460	274	206,080	1,741
**Region**	**Northeast**	11,160	235	73,620	1,550
	**Midwest**	11,960	197	83,540	1,376
	**South**	27,540	260	183,360	1,730
	**West**	14,780	274	87,520	1,622
**Total**		65,440	244	428,040	1,597

Source: Centers for Medicare & Medicaid Services, 5% Denominator and Claims Files.

Beneficiaries aged 65+ years with continuous and full Parts A and B enrollment and no Health Maintenance Organization enrollment during the year.

Unweighted counts were multiplied by 20 to produce counts for this table.

**Table 132:** Liver Disease: Hospital discharges with first-listed and all-listed diagnoses by age, race, sex and region among fee-for-service, age-eligible Medicare beneficiaries, 2019

Demographic Characteristics		First-Listed Diagnosis		All-Listed Diagnoses	
		Number of events	Event rate per 100,000	Number of events	Event rate per 100,000
**Age (years)**	**65 to 69**	17,200	213	111,100	1,377
	**70 to 74**	10,600	153	88,960	1,282
	**75 to 79**	7,040	144	64,720	1,322
	**80 to 84**	4,100	123	44,620	1,338
	**85+**	2,840	79	39,600	1,107
**Race**	**White**	31,240	142	269,620	1,224
	**Black**	2,300	124	30,060	1,615
	**Other**	8,240	282	49,320	1,686
**Sex**	**Female**	20,220	135	172,760	1,154
	**Male**	21,560	182	176,240	1,489
**Region**	**Northeast**	6,300	133	58,420	1,230
	**Midwest**	9,280	153	75,620	1,246
	**South**	17,400	164	145,820	1,376
	**West**	8,800	163	69,140	1,281
**Total**		41,780	156	349,000	1,302

Source: Centers for Medicare & Medicaid Services, 5% Denominator and Claims Files.

Beneficiaries aged 65+ years with continuous and full Parts A and B enrollment and no Health Maintenance Organization enrollment during the year.

Unweighted counts were multiplied by 20 to produce counts for this table.

**Table 133:** Hepatitis A: Ambulatory care visits with first-listed and all-listed diagnoses by age, race, ethnicity, and sex in the United States, 2015

Demographic Characteristics		All-Listed Diagnoses	
		Number in thousands	Rate per 100,000
**Age (years)**	**0 to 11**	.	.
	**12 to 24**	.	.
	**25 to 44**	.	.
	**45 to 54**	.	.
	**55 to 64**	10	25
	**65 to 74**	.	.
	**75 plus**	2	8
**Race**	**White**	11	3
	**Black**	0	1
	**Other**	.	.
**Ethnicity**	**Hispanic**	5	10
	**Not Hispanic**	7	2
**Sex**	**Female**	10	4
	**Male**	2	1
**Total**		12	3

Source: National Ambulatory Medical Care Survey (3-year average, 2014–2016).

Rates for age groups are unadjusted and all other rates are age-adjusted.

**Table 134:** Hepatitis A: Emergency department visits with first-listed and all-listed diagnoses by age and sex in the United States, 2018

Demographic Characteristics		First-Listed Diagnosis		All-Listed Diagnoses	
		Number in thousands	Rate per 100,000	Number in thousands	Rate per 100,000
**Age (years)**	**0 to 11**	0	0	0	0
	**12 to 24**	1	1	1	2
	**25 to 44**	4	5	8	9
	**45 to 54**	1	2	3	7
	**55 to 64**	1	1	3	7
	**65 to 74**	0	1	2	6
	**75 plus**	0	1	2	7
**Sex**	**Female**	3	2	8	5
	**Male**	4	3	10	6
**Total**		7	2	19	6

Source: Healthcare Cost and Utilization Project Nationwide Emergency Department Sample.

Rates for age groups are unadjusted and all other rates are age-adjusted.

**Table 135:** Hepatitis A: Hospital discharges with first-listed and all-listed diagnoses by age, race, ethnicity, and sex in the United States, 2018

Demographic Characteristics		First-Listed Diagnosis		All-Listed Diagnoses	
		Number in thousands	Rate per 100,000	Number in thousands	Rate per 100,000
**Age (years)**	**0 to 11**	0	0	0	0
	**12 to 24**	0	1	1	2
	**25 to 44**	3	4	6	7
	**45 to 54**	1	3	3	6
	**55 to 64**	0	1	2	6
	**65 to 74**	0	1	2	6
	**75 plus**	0	0	1	6
**Race**	**White**	5	2	13	5
	**Black**	0	1	1	3
	**Other**	0	1	1	3
**Ethnicity**	**Hispanic**	0	0	1	2
	**Not Hispanic**	5	2	14	5
**Sex**	**Female**	2	1	6	4
	**Male**	4	2	9	5
**Total**		6	2	15	4

Source: Healthcare Cost and Utilization Project National Inpatient Sample.

Rates for age groups are unadjusted and all other rates are age-adjusted.

**Table 136:** Hepatitis A: Deaths with underlying or underlying/other cause and lifetime years of life lost by age, race, ethnicity, and sex in the United States, 2019

Demographic Characteristics		Underlying Cause			Underlying/Other Cause		
		Number of Deaths	Rate per 100,000	Years of potential life lost in thousands	Number of Deaths	Rate per 100,000	Years of potential life lost in thousands
**Age (years)**	**0 to 11**	.	.	.	.	.	.
	**12 to 24**	.	.	.	.	.	.
	**25 to 44**	14	0.0	0.6	25	0.0	1.0
	**45 to 54**	30	0.1	0.9	56	0.1	1.7
	**55 to 64**	40	0.1	0.9	62	0.1	1.4
	**65 to 74**	26	0.1	0.4	46	0.1	0.7
	**75 plus**	29	0.1	0.2	37	0.2	0.3
**Race**	**White**	131	0.0	2.8	206	0.1	4.7
	**Black**	5	0.0	0.2	13	0.0	0.3
	**Other**	3	0.0	0.1	7	0.0	0.2
**Ethnicity**	**Hispanic**	4	0.0	0.1	11	0.0	0.3
	**Not Hispanic**	135	0.0	3.0	215	0.1	4.9
**Sex**	**Female**	38	0.0	0.9	66	0.0	1.6
	**Male**	101	0.1	2.2	160	0.1	3.6
**Total**		139	0.0	3.1	226	0.1	5.2

Source: Vital Statistics of the United States.

Rates for age groups are unadjusted and all other rates are age-adjusted.

Years of potential life lost is based on U.S. life Table 2006–2018 estimates of life expectancy at age of death.

**Table 137:** Hepatitis A: Claims-based prevalence with first-listed and all-listed diagnoses by age, race-ethnicity, sex and region among privately insured enrollees, 2020

Demographic Characteristics		Number of enrollees	First-Listed Diagnosis		All-Listed Diagnoses	
			Number of enrollees with disease	Percent of enrollees with disease	Number of enrollees with disease	Percent of enrollees with disease
**Age (years)**	**0 to 11**	1,101,978	3	0.0%	6	0.0%
	**12 to 24**	1,451,892	18	0.0%	36	0.0%
	**25 to 44**	2,656,672	57	0.0%	126	0.0%
	**45 to 54**	1,452,320	33	0.0%	122	0.0%
	**55 to 64**	1,640,216	73	0.0%	240	0.0%
	**65 to 74**	2,840,615	80	0.0%	492	0.0%
	**75 plus**	2,576,241	54	0.0%	391	0.0%
**Race-ethnicity**	**White**	8,657,702	190	0.0%	863	0.0%
	**Black**	1,242,477	37	0.0%	156	0.0%
	**Hispanic**	1,493,457	41	0.0%	199	0.0%
	**Asian**	607,284	24	0.0%	72	0.0%
	**Unknown**	1,719,014	26	0.0%	123	0.0%
**Sex**	**Female**	7,199,363	161	0.0%	717	0.0%
	**Male**	6,520,571	157	0.0%	696	0.0%
**Region**	**Northeast**	1,526,275	48	0.0%	209	0.0%
	**Midwest**	3,322,846	41	0.0%	203	0.0%
	**South**	5,619,435	177	0.0%	734	0.0%
	**West**	3,251,378	52	0.0%	267	0.0%
**Total**		13,719,934	318	0.0%	1,413	0.0%

Source: De-identified Optum Clinformatic ^®^ Data Mart.

Enrollees with full enrollment in a commercial health plan or Medicare during the year.

**Table 138:** Hepatitis A: Ambulatory care visits with first-listed and all-listed diagnoses by age, race-ethnicity, sex and region among privately insured enrollees, 2020

Demographic Characteristics		First-Listed Diagnosis		All-Listed Diagnoses	
		Number of events	Event rate per 100,000	Number of events	Event rate per 100,000
**Age (years)**	**0 to 11**	3	0	5	0
	**12 to 24**	20	1	37	3
	**25 to 44**	70	3	158	6
	**45 to 54**	47	3	155	11
	**55 to 64**	89	5	294	18
	**65 to 74**	103	4	607	21
	**75 plus**	66	3	507	20
**Race-ethnicity**	**White**	256	3	1,111	13
	**Black**	38	3	169	14
	**Hispanic**	44	3	263	18
	**Asian**	31	5	91	15
	**Unknown**	29	2	129	8
**Sex**	**Female**	198	3	926	13
	**Male**	200	3	837	13
**Region**	**Northeast**	67	4	310	20
	**Midwest**	61	2	231	7
	**South**	207	4	879	16
	**West**	63	2	343	11
**Total**		398	3	1,763	13

Source: De-identified Optum Clinformatic ^®^ Data Mart.

Enrollees with full enrollment in a commercial health plan or Medicare during the year.

**Table 139:** Hepatitis A: Emergency department visits with first-listed and all-listed diagnoses by age, race-ethnicity, sex and region among privately insured enrollees, 2020

Demographic Characteristics		First-Listed Diagnosis		All-Listed Diagnoses	
		Number of events	Event rate per 100,000	Number of events	Event rate per 100,000
**Age (years)**	**0 to 11**	.	.	.	.
	**12 to 24**	.	.	.	.
	**25 to 44**	2	0	6	0
	**45 to 54**	.	.	3	0
	**55 to 64**	5	0	10	1
	**65 to 74**	5	0	14	0
	**75 plus**	1	0	9	0
**Race-ethnicity**	**White**	8	0	29	0
	**Black**	.	.	2	0
	**Hispanic**	1	0	3	0
	**Asian**	.	.	1	0
	**Unknown**	4	0	7	0
**Sex**	**Female**	5	0	16	0
	**Male**	8	0	26	0
**Region**	**Northeast**	1	0	7	0
	**Midwest**	1	0	8	0
	**South**	9	0	20	0
	**West**	2	0	7	0
**Total**		13	0	42	0

Source: De-identified Optum Clinformatic ^®^ Data Mart.

Enrollees with full enrollment in a commercial health plan or Medicare during the year.

**Table 140:** Hepatitis A: Hospital discharges with first-listed and all-listed diagnoses by age, race-ethnicity, sex and region among privately insured enrollees, 2020

Demographic Characteristics		First-Listed Diagnosis		All-Listed Diagnoses	
		Number of events	Event rate per 100,000	Number of events	Event rate per 100,000
**Age (years)**	**0 to 11**	.	.	1	0
	**12 to 24**	.	.	11	1
	**25 to 44**	15	1	32	1
	**45 to 54**	12	1	39	3
	**55 to 64**	26	2	90	5
	**65 to 74**	18	1	177	6
	**75 plus**	11	0	145	6
**Race-ethnicity**	**White**	55	1	321	4
	**Black**	13	1	59	5
	**Hispanic**	6	0	58	4
	**Asian**	2	0	17	3
	**Unknown**	6	0	40	2
**Sex**	**Female**	37	1	235	3
	**Male**	45	1	260	4
**Region**	**Northeast**	9	1	56	4
	**Midwest**	11	0	82	2
	**South**	48	1	260	5
	**West**	14	0	97	3
**Total**		82	1	495	4

Source: De-identified Optum Clinformatic ^®^ Data Mart.

Enrollees with full enrollment in a commercial health plan or Medicare during the year.

**Table 141:** Hepatitis A: Claims-based prevalence with first-listed and all-listed diagnoses by age, race, sex and region among fee-for-service, age-eligible Medicare beneficiaries, 2019

Demographic Characteristics		Number of enrollees	First-Listed Diagnosis		All-Listed Diagnoses	
			Number of enrollees with disease	Percent of enrollees with disease	Number of enrollees with disease	Percent of enrollees with disease
**Age (years)**	**65 to 69**	8,066,320	300	0.0%	1,660	0.0%
	**70 to 74**	6,936,940	.	.	1,120	0.0%
	**75 to 79**	4,894,060	.	.	880	0.0%
	**80 to 84**	3,335,340	.	.	580	0.0%
	**85+**	3,578,120	.	.	400	0.0%
**Race**	**White**	22,023,800	640	0.0%	3,720	0.0%
	**Black**	1,861,660	.	.	220	0.0%
	**Other**	2,925,320	.	.	700	0.0%
**Sex**	**Female**	14,975,560	500	0.0%	2,540	0.0%
	**Male**	11,835,220	240	0.0%	2,100	0.0%
**Region**	**Northeast**	4,748,700	.	.	620	0.0%
	**Midwest**	6,069,800	.	.	780	0.0%
	**South**	10,595,900	360	0.0%	2,280	0.0%
	**West**	5,396,380	.	.	960	0.0%
**Total**		26,810,780	740	0.0%	4,640	0.0%

Source: Centers for Medicare & Medicaid Services, 5% Denominator and Claims Files.

Beneficiaries aged 65+ years with continuous and full Parts A and B enrollment and no Health Maintenance Organization enrollment during the year.

Unweighted counts were multiplied by 20 to produce counts for this table.

**Table 142:** Hepatitis A: Ambulatory care visits with first-listed and all-listed diagnoses by age, race, sex and region among fee-for-service, age-eligible Medicare beneficiaries, 2019

Demographic Characteristics		First-Listed Diagnosis		All-Listed Diagnoses	
		Number of events	Event rate per 100,000	Number of events	Event rate per 100,000
**Age (years)**	**65 to 69**	400	5	2,060	26
	**70 to 74**	.	.	1,000	14
	**75 to 79**	.	.	1,700	35
	**80 to 84**	.	.	.	.
	**85+**	.	.	.	.
**Race**	**White**	840	4	4,700	21
	**Black**	.	.	.	.
	**Other**	.	.	.	.
**Sex**	**Female**	.	.	2,740	18
	**Male**	.	.	2,880	24
**Region**	**Northeast**	.	.	720	15
	**Midwest**	.	.	480	8
	**South**	520	5	3,780	36
	**West**	.	.	640	12
**Total**		960	4	5,620	21

Source: Centers for Medicare & Medicaid Services, 5% Denominator and Claims Files.

Beneficiaries aged 65+ years with continuous and full Parts A and B enrollment and no Health Maintenance Organization enrollment during the year.

Unweighted counts were multiplied by 20 to produce counts for this table.

**Table 143:** Hepatitis A: Emergency department visits with first-listed and all-listed diagnoses by age, race, sex and region among fee-for-service, age-eligible Medicare beneficiaries, 2019

Demographic Characteristics		First-Listed Diagnosis		All-Listed Diagnoses	
		Number of events	Event rate per 100,000	Number of events	Event rate per 100,000
**Age (years)**	**65 to 69**	.	.	600	7
	**70 to 74**	.	.	480	7
	**75 to 79**	.	.	400	8
	**80 to 84**	.	.	320	10
	**85+**	.	.	260	7
**Race**	**White**	280	1	1,780	8
	**Black**	.	.	.	.
	**Other**	.	.	.	.
**Sex**	**Female**	.	.	1,020	7
	**Male**	.	.	1,040	9
**Region**	**Northeast**	.	.	260	5
	**Midwest**	.	.	420	7
	**South**	.	.	1,040	10
	**West**	.	.	340	6
**Total**		280	1	2,060	8

Source: Centers for Medicare & Medicaid Services, 5% Denominator and Claims Files.

Beneficiaries aged 65+ years with continuous and full Parts A and B enrollment and no Health Maintenance Organization enrollment during the year.

Unweighted counts were multiplied by 20 to produce counts for this table.

**Table 144:** Hepatitis A: Hospital discharges with first-listed and all-listed diagnoses by age, race, sex and region among fee-for-service, age-eligible Medicare beneficiaries, 2019

Demographic Characteristics		First-Listed Diagnosis		All-Listed Diagnoses	
		Number of events	Event rate per 100,000	Number of events	Event rate per 100,000
**Age (years)**	**65 to 69**	.	.	680	8
	**70 to 74**	.	.	520	7
	**75 to 79**	.	.	420	9
	**80 to 84**	.	.	.	.
	**85+**	.	.	.	.
**Race**	**White**	.	.	1,700	8
	**Black**	.	.	.	.
	**Other**	.	.	.	.
**Sex**	**Female**	.	.	900	6
	**Male**	.	.	1,140	10
**Region**	**Northeast**	.	.	.	.
	**Midwest**	.	.	.	.
	**South**	.	.	1,060	10
	**West**	.	.	380	7
**Total**		.	.	2,040	8

Source: Centers for Medicare & Medicaid Services, 5% Denominator and Claims Files.

Beneficiaries aged 65+ years with continuous and full Parts A and B enrollment and no Health Maintenance Organization enrollment during the year.

Unweighted counts were multiplied by 20 to produce counts for this table.

**Table 145:** Hepatitis B: Ambulatory care visits with first-listed and all-listed diagnoses by age, race, ethnicity, and sex in the United States, 2015

Demographic Characteristics		First-Listed Diagnosis		All-Listed Diagnoses	
		Number in thousands	Rate per 100,000	Number in thousands	Rate per 100,000
**Age (years)**	**0 to 11**	.	.	.	.
	**12 to 24**	31	56	31	56
	**25 to 44**	2	2	79	94
	**45 to 54**	100	231	191	444
	**55 to 64**	5	13	111	273
	**65 to 74**	2	9	62	224
	**75 plus**	0	1	289	1,431
**Race**	**White**	6	2	148	45
	**Black**	46	98	64	131
	**Other**	88	350	553	2,863
**Ethnicity**	**Hispanic**	.	.	17	34
	**Not Hispanic**	141	49	747	255
**Sex**	**Female**	4	2	196	123
	**Male**	136	81	568	362
**Total**		141	41	764	228

Source: National Ambulatory Medical Care Survey (3-year average, 2014–2016).

Rates for age groups are unadjusted and all other rates are age-adjusted.

**Table 146:** Hepatitis B: Emergency department visits with first-listed and all-listed diagnoses by age and sex in the United States, 2018

Demographic Characteristics		First-Listed Diagnosis		All-Listed Diagnoses	
		Number in thousands	Rate per 100,000	Number in thousands	Rate per 100,000
**Age (years)**	**0 to 11**	0	0	0	0
	**12 to 24**	0	0	1	3
	**25 to 44**	1	2	20	23
	**45 to 54**	1	2	20	48
	**55 to 64**	0	1	25	59
	**65 to 74**	0	1	14	47
	**75 plus**	0	0	8	35
**Sex**	**Female**	1	1	34	19
	**Male**	2	1	55	31
**Total**		3	1	89	25

Source: Healthcare Cost and Utilization Project Nationwide Emergency Department Sample.

Rates for age groups are unadjusted and all other rates are age-adjusted.

**Table 147:** Hepatitis B: Hospital discharges with first-listed and all-listed diagnoses by age, race, ethnicity, and sex in the United States, 2018

Demographic Characteristics		First-Listed Diagnosis		All-Listed Diagnoses	
		Number in thousands	Rate per 100,000	Number in thousands	Rate per 100,000
**Age (years)**	**0 to 11**	.	.	0	0
	**12 to 24**	0	0	1	3
	**25 to 44**	1	1	22	25
	**45 to 54**	1	2	16	38
	**55 to 64**	0	1	21	50
	**65 to 74**	0	1	14	47
	**75 plus**	0	0	7	34
**Race**	**White**	2	1	43	15
	**Black**	0	1	20	42
	**Other**	0	1	20	75
**Ethnicity**	**Hispanic**	0	0	7	15
	**Not Hispanic**	3	1	75	25
**Sex**	**Female**	1	1	34	19
	**Male**	2	1	48	27
**Total**		3	1	82	23

Source: Healthcare Cost and Utilization Project National Inpatient Sample.

Rates for age groups are unadjusted and all other rates are age-adjusted.

**Table 148:** Hepatitis B: Deaths with underlying or underlying/other cause and lifetime years of life lost by age, race, ethnicity, and sex in the United States, 2019

Demographic Characteristics		Underlying Cause			Underlying/Other Cause		
		Number of Deaths	Rate per 100,000	Years of potential life lost in thousands	Number of Deaths	Rate per 100,000	Years of potential life lost in thousands
**Age (years)**	**0 to 11**	.	.	.	.	.	.
	**12 to 24**	1	0.0	0.0	4	0.0	0.2
	**25 to 44**	42	0.0	1.7	153	0.2	6.3
	**45 to 54**	86	0.2	2.6	256	0.6	7.7
	**55 to 64**	118	0.3	2.7	506	1.2	11.3
	**65 to 74**	113	0.4	1.8	486	1.5	7.7
	**75 plus**	77	0.3	0.7	267	1.2	2.2
**Race**	**White**	249	0.1	5.7	879	0.3	19.0
	**Black**	59	0.1	1.3	299	0.6	6.6
	**Other**	129	0.5	2.6	494	1.8	9.8
**Ethnicity**	**Hispanic**	33	0.1	0.8	84	0.2	1.9
	**Not Hispanic**	404	0.1	8.8	1,588	0.5	33.5
**Sex**	**Female**	128	0.1	2.8	416	0.2	9.4
	**Male**	309	0.2	6.7	1,256	0.7	26.0
**Total**		437	0.1	9.6	1,672	0.4	35.4

Source: Vital Statistics of the United States.

Rates for age groups are unadjusted and all other rates are age-adjusted.

Years of potential life lost is based on U.S. life Table 2006–2018 estimates of life expectancy at age of death.

**Table 149:** Hepatitis B: Claims-based prevalence with first-listed and all-listed diagnoses by age, race-ethnicity, sex and region among privately insured enrollees, 2020

Demographic Characteristics		Number of enrollees	First-Listed Diagnosis		All-Listed Diagnoses	
			Number of enrollees with disease	Percent of enrollees with disease	Number of enrollees with disease	Percent of enrollees with disease
**Age (years)**	**0 to 11**	1,101,978	8	0.0%	27	0.0%
	**12 to 24**	1,451,892	83	0.0%	114	0.0%
	**25 to 44**	2,656,672	1,178	0.0%	1,911	0.1%
	**45 to 54**	1,452,320	1,146	0.1%	2,105	0.1%
	**55 to 64**	1,640,216	1,035	0.1%	2,563	0.2%
	**65 to 74**	2,840,615	1,996	0.1%	5,001	0.2%
	**75 plus**	2,576,241	732	0.0%	2,180	0.1%
**Race-ethnicity**	**White**	8,657,702	1,325	0.0%	3,977	0.0%
	**Black**	1,242,477	614	0.0%	1,617	0.1%
	**Hispanic**	1,493,457	344	0.0%	991	0.1%
	**Asian**	607,284	3,215	0.5%	5,828	1.0%
	**Unknown**	1,719,014	680	0.0%	1,488	0.1%
**Sex**	**Female**	7,199,363	2,806	0.0%	6,211	0.1%
	**Male**	6,520,571	3,372	0.1%	7,690	0.1%
**Region**	**Northeast**	1,526,275	1,253	0.1%	2,465	0.2%
	**Midwest**	3,322,846	736	0.0%	1,621	0.0%
	**South**	5,619,435	2,166	0.0%	5,480	0.1%
	**West**	3,251,378	2,023	0.1%	4,335	0.1%
**Total**		13,719,934	6,178	0.0%	13,901	0.1%

Source: De-identified Optum Clinformatic ^®^ Data Mart.

Enrollees with full enrollment in a commercial health plan or Medicare during the year.

**Table 150:** Hepatitis B: Ambulatory care visits with first-listed and all-listed diagnoses by age, race-ethnicity, sex and region among privately insured enrollees, 2020

Demographic Characteristics		First-Listed Diagnosis		All-Listed Diagnoses	
		Number of events	Event rate per 100,000	Number of events	Event rate per 100,000
**Age (years)**	**0 to 11**	9	1	34	3
	**12 to 24**	107	7	172	12
	**25 to 44**	1,740	65	3,365	127
	**45 to 54**	1,733	119	4,102	282
	**55 to 64**	1,586	97	4,978	303
	**65 to 74**	3,166	111	10,367	365
	**75 plus**	1,175	46	4,360	169
**Race-ethnicity**	**White**	1,992	23	6,877	79
	**Black**	931	75	2,886	232
	**Hispanic**	536	36	1,940	130
	**Asian**	5,005	824	12,783	2,105
	**Unknown**	1,052	61	2,892	168
**Sex**	**Female**	4,282	59	12,101	168
	**Male**	5,234	80	15,277	234
**Region**	**Northeast**	2,049	134	5,761	377
	**Midwest**	999	30	2,647	80
	**South**	3,400	61	10,329	184
	**West**	3,068	94	8,641	266
**Total**		9,516	69	27,378	200

Source: De-identified Optum Clinformatic ^®^ Data Mart.

Enrollees with full enrollment in a commercial health plan or Medicare during the year.

**Table 151:** Hepatitis B: Emergency department visits with first-listed and all-listed diagnoses by age, race-ethnicity, sex and region among privately insured enrollees, 2020

Demographic Characteristics		First-Listed Diagnosis		All-Listed Diagnoses	
		Number of events	Event rate per 100,000	Number of events	Event rate per 100,000
**Age (years)**	**0 to 11**	.	.	.	.
	**12 to 24**	3	0	3	0
	**25 to 44**	37	1	121	5
	**45 to 54**	2	0	16	1
	**55 to 64**	1	0	27	2
	**65 to 74**	1	0	25	1
	**75 plus**	.	.	23	1
**Race-ethnicity**	**White**	10	0	63	1
	**Black**	5	0	26	2
	**Hispanic**	.	.	8	1
	**Asian**	14	2	52	9
	**Unknown**	15	1	66	4
**Sex**	**Female**	43	1	162	2
	**Male**	1	0	53	1
**Region**	**Northeast**	7	0	28	2
	**Midwest**	5	0	49	1
	**South**	21	0	96	2
	**West**	11	0	42	1
**Total**		44	0	215	2

Source: De-identified Optum Clinformatic ^®^ Data Mart.

Enrollees with full enrollment in a commercial health plan or Medicare during the year.

**Table 152:** Hepatitis B: Hospital discharges with first-listed and all-listed diagnoses by age, race-ethnicity, sex and region among privately insured enrollees, 2020

Demographic Characteristics		First-Listed Diagnosis		All-Listed Diagnoses	
		Number of events	Event rate per 100,000	Number of events	Event rate per 100,000
**Age (years)**	**0 to 11**	.	.	4	0
	**12 to 24**	.	.	14	1
	**25 to 44**	8	0	211	8
	**45 to 54**	6	0	225	15
	**55 to 64**	6	0	457	28
	**65 to 74**	7	0	870	31
	**75 plus**	4	0	504	20
**Race-ethnicity**	**White**	17	0	1,030	12
	**Black**	4	0	434	35
	**Hispanic**	7	0	189	13
	**Asian**	2	0	414	68
	**Unknown**	1	0	218	13
**Sex**	**Female**	12	0	900	13
	**Male**	19	0	1,385	21
**Region**	**Northeast**	3	0	354	23
	**Midwest**	3	0	327	10
	**South**	23	0	1,110	20
	**West**	2	0	494	15
**Total**		31	0	2,285	17

Source: De-identified Optum Clinformatic ^®^ Data Mart.

Enrollees with full enrollment in a commercial health plan or Medicare during the year.

**Table 153:** Hepatitis B: Claims-based prevalence with first-listed and all-listed diagnoses by age, race, sex and region among fee-for-service, age-eligible Medicare beneficiaries, 2019

Demographic Characteristics		Number of enrollees	First-Listed Diagnosis		All-Listed Diagnoses	
			Number of enrollees with disease	Percent of enrollees with disease	Number of enrollees with disease	Percent of enrollees with disease
**Age (years)**	**65 to 69**	8,066,320	6,500	0.1%	15,680	0.2%
	**70 to 74**	6,936,940	4,720	0.1%	11,560	0.2%
	**75 to 79**	4,894,060	2,140	0.0%	5,540	0.1%
	**80 to 84**	3,335,340	1,580	0.0%	3,900	0.1%
	**85+**	3,578,120	740	0.0%	2,280	0.1%
**Race**	**White**	22,023,800	4,120	0.0%	14,740	0.1%
	**Black**	1,861,660	1,480	0.1%	4,560	0.2%
	**Other**	2,925,320	10,080	0.3%	19,660	0.7%
**Sex**	**Female**	14,975,560	8,060	0.1%	18,960	0.1%
	**Male**	11,835,220	7,620	0.1%	20,000	0.2%
**Region**	**Northeast**	4,748,700	2,980	0.1%	7,740	0.2%
	**Midwest**	6,069,800	1,780	0.0%	5,080	0.1%
	**South**	10,595,900	4,700	0.0%	12,300	0.1%
	**West**	5,396,380	6,220	0.1%	13,840	0.3%
**Total**		26,810,780	15,680	0.1%	38,960	0.1%

Source: Centers for Medicare & Medicaid Services, 5% Denominator and Claims Files.

Beneficiaries aged 65+ years with continuous and full Parts A and B enrollment and no Health Maintenance Organization enrollment during the year.

Unweighted counts were multiplied by 20 to produce counts for this table.

**Table 154:** Hepatitis B: Ambulatory care visits with first-listed and all-listed diagnoses by age, race, sex and region among fee-for-service, age-eligible Medicare beneficiaries, 2019

Demographic Characteristics		First-Listed Diagnosis		All-Listed Diagnoses	
		Number of events	Event rate per 100,000	Number of events	Event rate per 100,000
**Age (years)**	**65 to 69**	12,000	149	36,920	458
	**70 to 74**	8,980	129	30,120	434
	**75 to 79**	3,700	76	14,260	291
	**80 to 84**	3,060	92	11,340	340
	**85+**	1,180	33	5,700	159
**Race**	**White**	6,900	31	25,840	117
	**Black**	2,660	143	9,540	512
	**Other**	19,360	662	62,960	2,152
**Sex**	**Female**	14,860	99	48,540	324
	**Male**	14,060	119	49,800	421
**Region**	**Northeast**	5,540	117	22,660	477
	**Midwest**	2,960	49	9,700	160
	**South**	8,500	80	26,100	246
	**West**	11,920	221	39,880	739
**Total**		28,920	108	98,340	367

Source: Centers for Medicare & Medicaid Services, 5% Denominator and Claims Files.

Beneficiaries aged 65+ years with continuous and full Parts A and B enrollment and no Health Maintenance Organization enrollment during the year.

Unweighted counts were multiplied by 20 to produce counts for this table.

**Table 155:** Hepatitis B: Emergency department visits with first-listed and all-listed diagnoses by age, race, sex and region among fee-for-service, age-eligible Medicare beneficiaries, 2019

Demographic Characteristics		First-Listed Diagnosis		All-Listed Diagnoses	
		Number of events	Event rate per 100,000	Number of events	Event rate per 100,000
**Age (years)**	**65 to 69**	.	.	3,920	49
	**70 to 74**	.	.	2,860	41
	**75 to 79**	.	.	1,240	25
	**80 to 84**	.	.	1,460	44
	**85+**	.	.	1,020	29
**Race**	**White**	.	.	4,640	21
	**Black**	.	.	2,540	136
	**Other**	.	.	3,320	113
**Sex**	**Female**	.	.	4,400	29
	**Male**	.	.	6,100	52
**Region**	**Northeast**	.	.	1,820	38
	**Midwest**	.	.	2,160	36
	**South**	.	.	3,700	35
	**West**	.	.	2,820	52
**Total**		.	.	10,500	39

Source: Centers for Medicare & Medicaid Services, 5% Denominator and Claims Files.

Beneficiaries aged 65+ years with continuous and full Parts A and B enrollment and no Health Maintenance Organization enrollment during the year.

Unweighted counts were multiplied by 20 to produce counts for this table.

**Table 156:** Hepatitis B: Hospital discharges with first-listed and all-listed diagnoses by age, race, sex and region among fee-for-service, age-eligible Medicare beneficiaries, 2019

Demographic Characteristics		First-Listed Diagnosis		All-Listed Diagnoses	
		Number of events	Event rate per 100,000	Number of events	Event rate per 100,000
**Age (years)**	**65 to 69**	.	.	3,940	49
	**70 to 74**	.	.	3,040	44
	**75 to 79**	.	.	1,440	29
	**80 to 84**	.	.	1,440	43
	**85+**	.	.	880	25
**Race**	**White**	.	.	5,240	24
	**Black**	.	.	2,320	125
	**Other**	.	.	3,180	109
**Sex**	**Female**	.	.	4,500	30
	**Male**	.	.	6,240	53
**Region**	**Northeast**	.	.	2,080	44
	**Midwest**	.	.	1,960	32
	**South**	.	.	3,920	37
	**West**	.	.	2,780	52
**Total**		.	.	10,740	40

Source: Centers for Medicare & Medicaid Services, 5% Denominator and Claims Files.

Beneficiaries aged 65+ years with continuous and full Parts A and B enrollment and no Health Maintenance Organization enrollment during the year.

Unweighted counts were multiplied by 20 to produce counts for this table.

**Table 157:** Hepatitis C: Ambulatory care visits with first-listed and all-listed diagnoses by age, race, ethnicity, and sex in the United States, 2015

Demographic Characteristics		First-Listed Diagnosis		All-Listed Diagnoses	
		Number in thousands	Rate per 100,000	Number in thousands	Rate per 100,000
**Age (years)**	**0 to 11**	.	.	1	2
	**12 to 24**	31	56	48	85
	**25 to 44**	95	112	314	372
	**45 to 54**	151	349	371	861
	**55 to 64**	242	592	831	2,037
	**65 to 74**	61	223	392	1,425
	**75 plus**	.	.	114	565
**Race**	**White**	478	165	1,719	561
	**Black**	97	203	271	567
	**Other**	5	21	82	309
**Ethnicity**	**Hispanic**	15	29	352	818
	**Not Hispanic**	565	176	1,719	525
**Sex**	**Female**	280	138	888	434
	**Male**	300	174	1,184	665
**Total**		580	156	2,072	542

Source: National Ambulatory Medical Care Survey (3-year average, 2014–2016).

Rates for age groups are unadjusted and all other rates are age-adjusted.

**Table 158:** Hepatitis C: Emergency department visits with first-listed and all-listed diagnoses by age and sex in the United States, 2018

Demographic Characteristics		First-Listed Diagnosis		All-Listed Diagnoses	
		Number in thousands	Rate per 100,000	Number in thousands	Rate per 100,000
**Age (years)**	**0 to 11**	0	0	0	1
	**12 to 24**	1	1	15	27
	**25 to 44**	4	4	217	249
	**45 to 54**	1	3	182	436
	**55 to 64**	1	4	300	710
	**65 to 74**	1	2	124	407
	**75 plus**	0	1	26	120
**Sex**	**Female**	3	2	333	181
	**Male**	5	3	531	290
**Total**		8	2	865	234

Source: Healthcare Cost and Utilization Project Nationwide Emergency Department Sample.

Rates for age groups are unadjusted and all other rates are age-adjusted.

**Table 159:** Hepatitis C: Hospital discharges with first-listed and all-listed diagnoses by age, race, ethnicity, and sex in the United States, 2018

Demographic Characteristics		First-Listed Diagnosis		All-Listed Diagnoses	
		Number in thousands	Rate per 100,000	Number in thousands	Rate per 100,000
**Age (years)**	**0 to 11**	.	.	0	0
	**12 to 24**	0	0	13	23
	**25 to 44**	1	2	146	168
	**45 to 54**	1	1	112	270
	**55 to 64**	1	2	209	495
	**65 to 74**	0	1	98	321
	**75 plus**	0	0	21	97
**Race**	**White**	3	1	453	158
	**Black**	0	1	126	250
	**Other**	0	1	29	106
**Ethnicity**	**Hispanic**	0	1	71	140
	**Not Hispanic**	3	1	537	169
**Sex**	**Female**	1	1	233	125
	**Male**	2	1	367	197
**Total**		3	1	600	160

Source: Healthcare Cost and Utilization Project National Inpatient Sample.

Rates for age groups are unadjusted and all other rates are age-adjusted.

**Table 160:** Hepatitis C: Deaths with underlying or underlying/other cause and lifetime years of life lost by age, race, ethnicity, and sex in the United States, 2019

Demographic Characteristics		Underlying Cause			Underlying/Other Cause		
		Number of Deaths	Rate per 100,000	Years of potential life lost in thousands	Number of Deaths	Rate per 100,000	Years of potential life lost in thousands
**Age (years)**	**0 to 11**	.	.	.	.	.	.
	**12 to 24**	2	0.0	0.1	7	0.0	0.4
	**25 to 44**	144	0.2	6.0	643	0.7	27.2
	**45 to 54**	478	1.2	14.6	1,685	4.1	50.9
	**55 to 64**	1,525	3.6	34.8	6,343	14.9	141.7
	**65 to 74**	1,045	3.3	17.1	4,515	14.3	72.6
	**75 plus**	327	1.4	2.8	1,120	5.0	9.6
**Race**	**White**	2,826	0.8	62.4	10,999	3.2	241.5
	**Black**	550	1.1	10.1	2,693	5.2	48.1
	**Other**	145	0.5	2.9	621	2.3	12.9
**Ethnicity**	**Hispanic**	451	0.9	10.7	1,457	3.0	34.1
	**Not Hispanic**	3,070	0.8	64.7	12,856	3.4	268.4
**Sex**	**Female**	1,258	0.6	28.6	4,030	1.8	93.6
	**Male**	2,263	1.1	46.8	10,283	4.9	208.9
**Total**		3,521	0.8	75.4	14,313	3.3	302.5

Source: Vital Statistics of the United States.

Rates for age groups are unadjusted and all other rates are age-adjusted.

Years of potential life lost is based on U.S. life Table 2006–2018 estimates of life expectancy at age of death.

**Table 161:** Hepatitis C: Claims-based prevalence with first-listed and all-listed diagnoses by age, race-ethnicity, sex and region among privately insured enrollees, 2020

Demographic Characteristics		Number of enrollees	First-Listed Diagnosis		All-Listed Diagnoses	
			Number of enrollees with disease	Percent of enrollees with disease	Number of enrollees with disease	Percent of enrollees with disease
**Age (years)**	**0 to 11**	1,101,978	9	0.0%	23	0.0%
	**12 to 24**	1,451,892	78	0.0%	143	0.0%
	**25 to 44**	2,656,672	855	0.0%	1,904	0.1%
	**45 to 54**	1,452,320	788	0.1%	2,502	0.2%
	**55 to 64**	1,640,216	2,851	0.2%	10,497	0.6%
	**65 to 74**	2,840,615	4,838	0.2%	18,032	0.6%
	**75 plus**	2,576,241	923	0.0%	4,272	0.2%
**Race-ethnicity**	**White**	8,657,702	5,560	0.1%	20,564	0.2%
	**Black**	1,242,477	2,102	0.2%	7,171	0.6%
	**Hispanic**	1,493,457	1,197	0.1%	4,562	0.3%
	**Asian**	607,284	276	0.0%	1,047	0.2%
	**Unknown**	1,719,014	1,207	0.1%	4,029	0.2%
**Sex**	**Female**	7,199,363	4,044	0.1%	14,997	0.2%
	**Male**	6,520,571	6,298	0.1%	22,376	0.3%
**Region**	**Northeast**	1,526,275	1,188	0.1%	4,269	0.3%
	**Midwest**	3,322,846	1,356	0.0%	4,930	0.1%
	**South**	5,619,435	5,668	0.1%	20,093	0.4%
	**West**	3,251,378	2,130	0.1%	8,081	0.2%
**Total**		13,719,934	10,342	0.1%	37,373	0.3%

Source: De-identified Optum Clinformatic ^®^ Data Mart.

Enrollees with full enrollment in a commercial health plan or Medicare during the year.

**Table 162:** Hepatitis C: Ambulatory care visits with first-listed and all-listed diagnoses by age, race-ethnicity, sex and region among privately insured enrollees, 2020

Demographic Characteristics		First-Listed Diagnosis		All-Listed Diagnoses	
		Number of events	Event rate per 100,000	Number of events	Event rate per 100,000
**Age (years)**	**0 to 11**	16	1	45	4
	**12 to 24**	126	9	271	19
	**25 to 44**	1,457	55	3,692	139
	**45 to 54**	1,606	111	5,314	366
	**55 to 64**	4,825	294	21,782	1,328
	**65 to 74**	7,997	282	36,232	1,275
	**75 plus**	1,459	57	8,009	311
**Race-ethnicity**	**White**	9,257	107	40,491	468
	**Black**	3,514	283	14,905	1,200
	**Hispanic**	1,946	130	9,346	626
	**Asian**	408	67	1,923	317
	**Unknown**	2,361	137	8,680	505
**Sex**	**Female**	6,530	91	29,672	412
	**Male**	10,956	168	45,673	700
**Region**	**Northeast**	1,902	125	8,348	547
	**Midwest**	2,427	73	9,366	282
	**South**	9,675	172	41,631	741
	**West**	3,482	107	16,000	492
**Total**		17,486	127	75,345	549

Source: De-identified Optum Clinformatic ^®^ Data Mart.

Enrollees with full enrollment in a commercial health plan or Medicare during the year.

**Table 163:** Hepatitis C: Emergency department visits with first-listed and all-listed diagnoses by age, race-ethnicity, sex and region among privately insured enrollees, 2020

Demographic Characteristics		First-Listed Diagnosis		All-Listed Diagnoses	
		Number of events	Event rate per 100,000	Number of events	Event rate per 100,000
**Age (years)**	**0 to 11**	.	.	.	.
	**12 to 24**	.	.	4	0
	**25 to 44**	5	0	65	2
	**45 to 54**	3	0	55	4
	**55 to 64**	19	1	213	13
	**65 to 74**	10	0	234	8
	**75 plus**	3	0	56	2
**Race-ethnicity**	**White**	24	0	347	4
	**Black**	12	1	114	9
	**Hispanic**	2	0	60	4
	**Asian**	.	.	11	2
	**Unknown**	2	0	95	6
**Sex**	**Female**	17	0	228	3
	**Male**	23	0	399	6
**Region**	**Northeast**	1	0	95	6
	**Midwest**	7	0	93	3
	**South**	27	0	315	6
	**West**	5	0	124	4
**Total**		40	0	627	5

Source: De-identified Optum Clinformatic ^®^ Data Mart.

Enrollees with full enrollment in a commercial health plan or Medicare during the year.

**Table 164:** Hepatitis C: Hospital discharges with first-listed and all-listed diagnoses by age, race-ethnicity, sex and region among privately insured enrollees, 2020

Demographic Characteristics		First-Listed Diagnosis		All-Listed Diagnoses	
		Number of events	Event rate per 100,000	Number of events	Event rate per 100,000
**Age (years)**	**0 to 11**	.	.	1	0
	**12 to 24**	.	.	41	3
	**25 to 44**	1	0	436	16
	**45 to 54**	2	0	924	64
	**55 to 64**	6	0	3,968	242
	**65 to 74**	18	1	5,690	200
	**75 plus**	3	0	1,480	57
**Race-ethnicity**	**White**	16	0	6,740	78
	**Black**	5	0	2,771	223
	**Hispanic**	2	0	1,281	86
	**Asian**	.	.	196	32
	**Unknown**	7	0	1,552	90
**Sex**	**Female**	14	0	4,904	68
	**Male**	16	0	7,636	117
**Region**	**Northeast**	1	0	1,405	92
	**Midwest**	5	0	1,764	53
	**South**	18	0	7,127	127
	**West**	6	0	2,244	69
**Total**		30	0	12,540	91

Source: De-identified Optum Clinformatic ^®^ Data Mart.

Enrollees with full enrollment in a commercial health plan or Medicare during the year.

**Table 165:** Hepatitis C: Claims-based prevalence with first-listed and all-listed diagnoses by age, race, sex and region among fee-for-service, age-eligible Medicare beneficiaries, 2019

Demographic Characteristics		Number of enrollees	First-Listed Diagnosis		All-Listed Diagnoses	
			Number of enrollees with disease	Percent of enrollees with disease	Number of enrollees with disease	Percent of enrollees with disease
**Age (years)**	**65 to 69**	8,066,320	23,280	0.3%	69,660	0.9%
	**70 to 74**	6,936,940	8,880	0.1%	28,340	0.4%
	**75 to 79**	4,894,060	3,620	0.1%	11,460	0.2%
	**80 to 84**	3,335,340	1,880	0.1%	6,420	0.2%
	**85+**	3,578,120	920	0.0%	4,920	0.1%
**Race**	**White**	22,023,800	23,820	0.1%	74,000	0.3%
	**Black**	1,861,660	9,280	0.5%	29,360	1.6%
	**Other**	2,925,320	5,480	0.2%	17,440	0.6%
**Sex**	**Female**	14,975,560	15,740	0.1%	46,860	0.3%
	**Male**	11,835,220	22,840	0.2%	73,940	0.6%
**Region**	**Northeast**	4,748,700	6,700	0.1%	24,040	0.5%
	**Midwest**	6,069,800	5,660	0.1%	18,840	0.3%
	**South**	10,595,900	14,940	0.1%	46,500	0.4%
	**West**	5,396,380	11,280	0.2%	31,420	0.6%
**Total**		26,810,780	38,580	0.1%	120,800	0.5%

Source: Centers for Medicare & Medicaid Services, 5% Denominator and Claims Files.

Beneficiaries aged 65+ years with continuous and full Parts A and B enrollment and no Health Maintenance Organization enrollment during the year.

Unweighted counts were multiplied by 20 to produce counts for this table.

**Table 166:** Hepatitis C: Ambulatory care visits with first-listed and all-listed diagnoses by age, race, sex and region among fee-for-service, age-eligible Medicare beneficiaries, 2019

Demographic Characteristics		First-Listed Diagnosis		All-Listed Diagnoses	
		Number of events	Event rate per 100,000	Number of events	Event rate per 100,000
**Age (years)**	**65 to 69**	42,580	528	154,180	1,911
	**70 to 74**	16,240	234	63,940	922
	**75 to 79**	6,720	137	25,720	526
	**80 to 84**	4,420	133	14,380	431
	**85+**	1,840	51	9,300	260
**Race**	**White**	41,560	189	154,420	701
	**Black**	18,460	992	70,440	3,784
	**Other**	11,780	403	42,660	1,458
**Sex**	**Female**	29,940	200	102,340	683
	**Male**	41,860	354	165,180	1,396
**Region**	**Northeast**	12,240	258	51,280	1,080
	**Midwest**	9,460	156	38,660	637
	**South**	28,120	265	107,020	1,010
	**West**	21,980	407	70,560	1,308
**Total**		71,800	268	267,520	998

Source: Centers for Medicare & Medicaid Services, 5% Denominator and Claims Files.

Beneficiaries aged 65+ years with continuous and full Parts A and B enrollment and no Health Maintenance Organization enrollment during the year.

Unweighted counts were multiplied by 20 to produce counts for this table.

**Table 167:** Hepatitis C: Emergency department visits with first-listed and all-listed diagnoses by age, race, sex and region among fee-for-service, age-eligible Medicare beneficiaries, 2019

Demographic Characteristics		First-Listed Diagnosis		All-Listed Diagnoses	
		Number of events	Event rate per 100,000	Number of events	Event rate per 100,000
**Age (years)**	**65 to 69**	240	3	36,740	455
	**70 to 74**	.	.	14,120	204
	**75 to 79**	.	.	6,280	128
	**80 to 84**	.	.	2,920	88
	**85+**	.	.	2,940	82
**Race**	**White**	.	.	34,500	157
	**Black**	.	.	20,620	1,108
	**Other**	.	.	7,880	269
**Sex**	**Female**	.	.	23,300	156
	**Male**	.	.	39,700	335
**Region**	**Northeast**	.	.	12,780	269
	**Midwest**	.	.	10,660	176
	**South**	.	.	23,600	223
	**West**	.	.	15,960	296
**Total**		400	1	63,000	235

Source: Centers for Medicare & Medicaid Services, 5% Denominator and Claims Files.

Beneficiaries aged 65+ years with continuous and full Parts A and B enrollment and no Health Maintenance Organization enrollment during the year.

Unweighted counts were multiplied by 20 to produce counts for this table.

**Table 168:** Hepatitis C: Hospital discharges with first-listed and all-listed diagnoses by age, race, sex and region among fee-for-service, age-eligible Medicare beneficiaries, 2019

Demographic Characteristics		First-Listed Diagnosis		All-Listed Diagnoses	
		Number of events	Event rate per 100,000	Number of events	Event rate per 100,000
**Age (years)**	**65 to 69**	.	.	30,660	380
	**70 to 74**	.	.	11,920	172
	**75 to 79**	.	.	5,460	112
	**80 to 84**	.	.	2,500	75
	**85+**	.	.	2,520	70
**Race**	**White**	.	.	30,080	137
	**Black**	.	.	16,160	868
	**Other**	.	.	6,820	233
**Sex**	**Female**	.	.	19,280	129
	**Male**	.	.	33,780	285
**Region**	**Northeast**	.	.	11,400	240
	**Midwest**	.	.	8,940	147
	**South**	.	.	20,100	190
	**West**	.	.	12,620	234
**Total**		.	.	53,060	198

Source: Centers for Medicare & Medicaid Services, 5% Denominator and Claims Files.

Beneficiaries aged 65+ years with continuous and full Parts A and B enrollment and no Health Maintenance Organization enrollment during the year.

Unweighted counts were multiplied by 20 to produce counts for this table.

**Table 169:** Gallstones: Ambulatory care visits with first-listed and all-listed diagnoses by age, race, ethnicity, and sex in the United States, 2015

Demographic Characteristics		First-Listed Diagnosis		All-Listed Diagnoses	
		Number in thousands	Rate per 100,000	Number in thousands	Rate per 100,000
**Age (years)**	**0 to 11**	2	4	2	4
	**12 to 24**	123	219	148	263
	**25 to 44**	383	453	565	669
	**45 to 54**	233	542	467	1,084
	**55 to 64**	328	804	414	1,015
	**65 to 74**	172	624	389	1,415
	**75 plus**	113	558	218	1,080
**Race**	**White**	1,139	426	1,901	691
	**Black**	147	313	172	380
	**Other**	67	235	131	519
**Ethnicity**	**Hispanic**	491	1,029	600	1,254
	**Not Hispanic**	863	305	1,604	554
**Sex**	**Female**	899	546	1,466	870
	**Male**	454	255	738	418
**Total**		1,354	397	2,204	642

Source: National Ambulatory Medical Care Survey (3-year average, 2014–2016).

Rates for age groups are unadjusted and all other rates are age-adjusted.

**Table 170:** Gallstones: Emergency department visits with first-listed and all-listed diagnoses by age and sex in the United States, 2018

Demographic Characteristics		First-Listed Diagnosis		All-Listed Diagnoses	
		Number in thousands	Rate per 100,000	Number in thousands	Rate per 100,000
**Age (years)**	**0 to 11**	1	2	2	5
	**12 to 24**	71	128	97	175
	**25 to 44**	238	273	335	385
	**45 to 54**	97	234	163	390
	**55 to 64**	88	208	179	423
	**65 to 74**	71	234	169	553
	**75 plus**	67	305	205	935
**Sex**	**Female**	437	260	734	417
	**Male**	196	117	416	243
**Total**		633	188	1,149	328

Source: Healthcare Cost and Utilization Project Nationwide Emergency Department Sample.

Rates for age groups are unadjusted and all other rates are age-adjusted.

**Table 171:** Gallstones: Hospital discharges with first-listed and all-listed diagnoses by age, race, ethnicity, and sex in the United States, 2018

Demographic Characteristics		First-Listed Diagnosis		All-Listed Diagnoses	
		Number in thousands	Rate per 100,000	Number in thousands	Rate per 100,000
**Age (years)**	**0 to 11**	1	1	2	4
	**12 to 24**	18	32	29	52
	**25 to 44**	67	78	114	131
	**45 to 54**	38	91	77	186
	**55 to 64**	45	107	108	256
	**65 to 74**	46	149	121	397
	**75 plus**	53	243	164	746
**Race**	**White**	222	78	502	169
	**Black**	27	61	72	165
	**Other**	21	82	48	188
**Ethnicity**	**Hispanic**	58	112	112	233
	**Not Hispanic**	213	69	510	158
**Sex**	**Female**	166	92	353	186
	**Male**	102	59	263	151
**Total**		268	75	615	167

Source: Healthcare Cost and Utilization Project National Inpatient Sample.

Rates for age groups are unadjusted and all other rates are age-adjusted.

**Table 172:** Gallstones: Deaths with underlying or underlying/other cause and lifetime years of life lost by age, race, ethnicity, and sex in the United States, 2019

Demographic Characteristics		Underlying Cause			Underlying/Other Cause		
		Number of Deaths	Rate per 100,000	Years of potential life lost in thousands	Number of Deaths	Rate per 100,000	Years of potential life lost in thousands
**Age (years)**	**0 to 11**	.	.	.	.	.	.
	**12 to 24**	1	0.0	0.1	3	0.0	0.2
	**25 to 44**	21	0.0	0.9	61	0.1	2.6
	**45 to 54**	38	0.1	1.2	66	0.2	2.1
	**55 to 64**	99	0.2	2.3	190	0.4	4.4
	**65 to 74**	181	0.6	2.8	307	1.0	4.8
	**75 plus**	710	3.1	4.4	1,169	5.2	7.4
**Race**	**White**	892	0.3	9.8	1,517	0.4	17.6
	**Black**	77	0.2	1.0	154	0.4	2.4
	**Other**	81	0.4	0.9	125	0.5	1.5
**Ethnicity**	**Hispanic**	99	0.3	1.4	157	0.4	2.5
	**Not Hispanic**	951	0.3	10.3	1,639	0.4	19.0
**Sex**	**Female**	564	0.2	5.9	959	0.4	10.9
	**Male**	486	0.3	5.7	837	0.5	10.5
**Total**		1,050	0.3	11.7	1,796	0.4	21.5

Source: Vital Statistics of the United States.

Rates for age groups are unadjusted and all other rates are age-adjusted.

Years of potential life lost is based on U.S. life Table 2006–2018 estimates of life expectancy at age of death.

**Table 173:** Gallstones: Claims-based prevalence with first-listed and all-listed diagnoses by age, race-ethnicity, sex and region among privately insured enrollees, 2020

Demographic Characteristics		Number of enrollees	First-Listed Diagnosis		All-Listed Diagnoses	
			Number of enrollees with disease	Percent of enrollees with disease	Number of enrollees with disease	Percent of enrollees with disease
**Age (years)**	**0 to 11**	1,101,978	36	0.0%	82	0.0%
	**12 to 24**	1,451,892	1,211	0.1%	1,547	0.1%
	**25 to 44**	2,656,672	8,239	0.3%	10,843	0.4%
	**45 to 54**	1,452,320	5,509	0.4%	8,212	0.6%
	**55 to 64**	1,640,216	6,963	0.4%	12,254	0.7%
	**65 to 74**	2,840,615	13,714	0.5%	26,749	0.9%
	**75 plus**	2,576,241	12,840	0.5%	30,192	1.2%
**Race-ethnicity**	**White**	8,657,702	31,245	0.4%	57,469	0.7%
	**Black**	1,242,477	4,746	0.4%	9,305	0.7%
	**Hispanic**	1,493,457	7,159	0.5%	12,949	0.9%
	**Asian**	607,284	1,460	0.2%	3,019	0.5%
	**Unknown**	1,719,014	3,902	0.2%	7,137	0.4%
**Sex**	**Female**	7,199,363	30,418	0.4%	52,778	0.7%
	**Male**	6,520,571	18,094	0.3%	37,101	0.6%
**Region**	**Northeast**	1,526,275	5,166	0.3%	10,209	0.7%
	**Midwest**	3,322,846	10,772	0.3%	18,438	0.6%
	**South**	5,619,435	22,296	0.4%	41,418	0.7%
	**West**	3,251,378	10,278	0.3%	19,814	0.6%
**Total**		13,719,934	48,512	0.4%	89,879	0.7%

Source: De-identified Optum Clinformatic ^®^ Data Mart.

Enrollees with full enrollment in a commercial health plan or Medicare during the year.

**Table 174:** Gallstones: Ambulatory care visits with first-listed and all-listed diagnoses by age, race-ethnicity, sex and region among privately insured enrollees, 2020

Demographic Characteristics		First-Listed Diagnosis		All-Listed Diagnoses	
		Number of events	Event rate per 100,000	Number of events	Event rate per 100,000
**Age (years)**	**0 to 11**	51	5	115	10
	**12 to 24**	2,211	152	2,883	199
	**25 to 44**	14,812	558	20,158	759
	**45 to 54**	9,409	648	14,121	972
	**55 to 64**	10,982	670	19,041	1,161
	**65 to 74**	19,796	697	37,835	1,332
	**75 plus**	16,815	653	38,397	1,490
**Race-ethnicity**	**White**	47,672	551	84,416	975
	**Black**	7,180	578	13,137	1,057
	**Hispanic**	11,029	738	19,525	1,307
	**Asian**	2,152	354	4,656	767
	**Unknown**	6,043	352	10,816	629
**Sex**	**Female**	48,371	672	82,723	1,149
	**Male**	25,705	394	49,827	764
**Region**	**Northeast**	7,416	486	14,458	947
	**Midwest**	16,514	497	27,831	838
	**South**	34,550	615	61,224	1,090
	**West**	15,596	480	29,037	893
**Total**		74,076	540	132,550	966

Source: De-identified Optum Clinformatic ^®^ Data Mart.

Enrollees with full enrollment in a commercial health plan or Medicare during the year.

**Table 175:** Gallstones: Emergency department visits with first-listed and all-listed diagnoses by age, race-ethnicity, sex and region among privately insured enrollees, 2020

Demographic Characteristics		First-Listed Diagnosis		All-Listed Diagnoses	
		Number of events	Event rate per 100,000	Number of events	Event rate per 100,000
**Age (years)**	**0 to 11**	.	.	.	.
	**12 to 24**	33	2	52	4
	**25 to 44**	328	12	542	20
	**45 to 54**	301	21	503	35
	**55 to 64**	693	42	1,215	74
	**65 to 74**	2,783	98	5,048	178
	**75 plus**	2,979	116	6,000	233
**Race-ethnicity**	**White**	4,534	52	8,558	99
	**Black**	789	64	1,515	122
	**Hispanic**	1,016	68	1,814	121
	**Asian**	172	28	340	56
	**Unknown**	606	35	1,133	66
**Sex**	**Female**	4,215	59	7,754	108
	**Male**	2,902	45	5,606	86
**Region**	**Northeast**	921	60	1,545	101
	**Midwest**	1,499	45	2,706	81
	**South**	2,981	53	5,800	103
	**West**	1,716	53	3,309	102
**Total**		7,117	52	13,360	97

Source: De-identified Optum Clinformatic ^®^ Data Mart.

Enrollees with full enrollment in a commercial health plan or Medicare during the year.

**Table 176:** Gallstones: Hospital discharges with first-listed and all-listed diagnoses by age, race-ethnicity, sex and region among privately insured enrollees, 2020

Demographic Characteristics		First-Listed Diagnosis		All-Listed Diagnoses	
		Number of events	Event rate per 100,000	Number of events	Event rate per 100,000
**Age (years)**	**0 to 11**	3	0	29	3
	**12 to 24**	132	9	259	18
	**25 to 44**	830	31	1,766	66
	**45 to 54**	670	46	1,716	118
	**55 to 64**	1,109	68	3,526	215
	**65 to 74**	2,847	100	9,549	336
	**75 plus**	4,128	160	15,044	584
**Race-ethnicity**	**White**	6,342	73	20,616	238
	**Black**	926	75	3,565	287
	**Hispanic**	1,441	96	4,412	295
	**Asian**	264	43	837	138
	**Unknown**	746	43	2,459	143
**Sex**	**Female**	5,441	76	16,727	232
	**Male**	4,278	66	15,162	233
**Region**	**Northeast**	1,320	86	4,145	272
	**Midwest**	2,059	62	6,564	198
	**South**	4,204	75	14,278	254
	**West**	2,136	66	6,902	212
**Total**		9,719	71	31,889	232

Source: De-identified Optum Clinformatic ^®^ Data Mart.

Enrollees with full enrollment in a commercial health plan or Medicare during the year.

**Table 177:** Gallstones: Claims-based prevalence with first-listed and all-listed diagnoses by age, race, sex and region among fee-for-service, age-eligible Medicare beneficiaries, 2019

Demographic Characteristics		Number of enrollees	First-Listed Diagnosis		All-Listed Diagnoses	
			Number of enrollees with disease	Percent of enrollees with disease	Number of enrollees with disease	Percent of enrollees with disease
**Age (years)**	**65 to 69**	8,066,320	56,600	0.7%	105,120	1.3%
	**70 to 74**	6,936,940	53,220	0.8%	105,220	1.5%
	**75 to 79**	4,894,060	41,060	0.8%	84,680	1.7%
	**80 to 84**	3,335,340	32,620	1.0%	70,320	2.1%
	**85+**	3,578,120	34,720	1.0%	77,220	2.2%
**Race**	**White**	22,023,800	178,220	0.8%	359,020	1.6%
	**Black**	1,861,660	13,680	0.7%	29,380	1.6%
	**Other**	2,925,320	26,320	0.9%	54,160	1.9%
**Sex**	**Female**	14,975,560	121,980	0.8%	237,800	1.6%
	**Male**	11,835,220	96,240	0.8%	204,760	1.7%
**Region**	**Northeast**	4,748,700	37,900	0.8%	80,840	1.7%
	**Midwest**	6,069,800	46,320	0.8%	91,140	1.5%
	**South**	10,595,900	90,360	0.9%	181,020	1.7%
	**West**	5,396,380	43,640	0.8%	89,560	1.7%
**Total**		26,810,780	218,220	0.8%	442,560	1.7%

Source: Centers for Medicare & Medicaid Services, 5% Denominator and Claims Files.

Beneficiaries aged 65+ years with continuous and full Parts A and B enrollment and no Health Maintenance Organization enrollment during the year.

Unweighted counts were multiplied by 20 to produce counts for this table.

**Table 178:** Gallstones: Ambulatory care visits with first-listed and all-listed diagnoses by age, race, sex and region among fee-for-service, age-eligible Medicare beneficiaries, 2019

Demographic Characteristics		First-Listed Diagnosis		All-Listed Diagnoses	
		Number of events	Event rate per 100,000	Number of events	Event rate per 100,000
**Age (years)**	**65 to 69**	67,100	832	132,220	1,639
	**70 to 74**	60,220	868	127,760	1,842
	**75 to 79**	44,580	911	98,400	2,011
	**80 to 84**	32,480	974	75,720	2,270
	**85+**	29,720	831	73,600	2,057
**Race**	**White**	192,640	875	410,820	1,865
	**Black**	12,440	668	29,220	1,570
	**Other**	29,020	992	67,660	2,313
**Sex**	**Female**	135,260	903	286,460	1,913
	**Male**	98,840	835	221,240	1,869
**Region**	**Northeast**	41,460	873	99,840	2,102
	**Midwest**	49,200	811	101,620	1,674
	**South**	98,260	927	203,340	1,919
	**West**	45,180	837	102,900	1,907
**Total**		234,100	873	507,700	1,894

Source: Centers for Medicare & Medicaid Services, 5% Denominator and Claims Files.

Beneficiaries aged 65+ years with continuous and full Parts A and B enrollment and no Health Maintenance Organization enrollment during the year.

Unweighted counts were multiplied by 20 to produce counts for this table.

**Table 179:** Gallstones: Emergency department visits with first-listed and all-listed diagnoses by age, race, sex and region among fee-for-service, age-eligible Medicare beneficiaries, 2019

Demographic Characteristics		First-Listed Diagnosis		All-Listed Diagnoses	
		Number of events	Event rate per 100,000	Number of events	Event rate per 100,000
**Age (years)**	**65 to 69**	25,800	320	53,680	665
	**70 to 74**	25,340	365	55,220	796
	**75 to 79**	20,860	426	47,900	979
	**80 to 84**	17,760	532	43,640	1,308
	**85+**	23,180	648	58,780	1,643
**Race**	**White**	90,480	411	206,440	937
	**Black**	8,400	451	21,460	1,153
	**Other**	14,060	481	31,320	1,071
**Sex**	**Female**	60,720	405	134,780	900
	**Male**	52,220	441	124,440	1,051
**Region**	**Northeast**	19,600	413	44,740	942
	**Midwest**	23,120	381	54,360	896
	**South**	46,140	435	107,020	1,010
	**West**	24,080	446	53,100	984
**Total**		112,940	421	259,220	967

Source: Centers for Medicare & Medicaid Services, 5% Denominator and Claims Files.

Beneficiaries aged 65+ years with continuous and full Parts A and B enrollment and no Health Maintenance Organization enrollment during the year.

Unweighted counts were multiplied by 20 to produce counts for this table.

**Table 180:** Gallstones: Hospital discharges with first-listed and all-listed diagnoses by age, race, sex and region among fee-for-service, age-eligible Medicare beneficiaries, 2019

Demographic Characteristics		First-Listed Diagnosis		All-Listed Diagnoses	
		Number of events	Event rate per 100,000	Number of events	Event rate per 100,000
**Age (years)**	**65 to 69**	10,680	132	30,300	376
	**70 to 74**	11,620	168	33,480	483
	**75 to 79**	10,120	207	29,180	596
	**80 to 84**	8,980	269	28,700	860
	**85+**	12,040	336	37,040	1,035
**Race**	**White**	43,540	198	126,060	572
	**Black**	3,220	173	12,360	664
	**Other**	6,680	228	20,280	693
**Sex**	**Female**	27,600	184	82,140	548
	**Male**	25,840	218	76,560	647
**Region**	**Northeast**	9,700	204	27,380	577
	**Midwest**	12,480	206	35,660	587
	**South**	21,100	199	64,320	607
	**West**	10,160	188	31,340	581
**Total**		53,440	199	158,700	592

Source: Centers for Medicare & Medicaid Services, 5% Denominator and Claims Files.

Beneficiaries aged 65+ years with continuous and full Parts A and B enrollment and no Health Maintenance Organization enrollment during the year.

Unweighted counts were multiplied by 20 to produce counts for this table.

**Table 181:** Acute Pancreatitis: Ambulatory care visits with first-listed and all-listed diagnoses by age, race, ethnicity, and sex in the United States, 2015

Demographic Characteristics		First-Listed Diagnosis		All-Listed Diagnoses	
		Number in thousands	Rate per 100,000	Number in thousands	Rate per 100,000
**Age (years)**	**0 to 11**	2	5	2	5
	**12 to 24**	25	45	32	57
	**25 to 44**	113	133	202	239
	**45 to 54**	165	383	234	543
	**55 to 64**	86	212	236	578
	**65 to 74**	32	117	125	453
	**75 plus**	21	103	66	325
**Race**	**White**	391	158	720	275
	**Black**	47	111	161	333
	**Other**	7	31	16	78
**Ethnicity**	**Hispanic**	73	140	125	265
	**Not Hispanic**	371	144	772	269
**Sex**	**Female**	239	155	484	282
	**Male**	206	125	413	250
**Total**		445	140	897	264

Source: National Ambulatory Medical Care Survey (3-year average, 2014–2016).

Rates for age groups are unadjusted and all other rates are age-adjusted.

**Table 182:** Acute Pancreatitis: Emergency department visits with first-listed and all-listed diagnoses by age and sex in the United States, 2018

Demographic Characteristics		First-Listed Diagnosis		All-Listed Diagnoses	
		Number in thousands	Rate per 100,000	Number in thousands	Rate per 100,000
**Age (years)**	**0 to 11**	2	4	3	6
	**12 to 24**	20	36	31	55
	**25 to 44**	123	141	177	203
	**45 to 54**	82	197	122	294
	**55 to 64**	73	173	115	273
	**65 to 74**	44	144	74	243
	**75 plus**	33	152	62	284
**Sex**	**Female**	172	98	274	154
	**Male**	206	125	310	187
**Total**		377	111	585	170

Source: Healthcare Cost and Utilization Project Nationwide Emergency Department Sample.

Rates for age groups are unadjusted and all other rates are age-adjusted.

**Table 183:** Acute Pancreatitis: Hospital discharges with first-listed and all-listed diagnoses by age, race, ethnicity, and sex in the United States, 2018

Demographic Characteristics		First-Listed Diagnosis		All-Listed Diagnoses	
		Number in thousands	Rate per 100,000	Number in thousands	Rate per 100,000
**Age (years)**	**0 to 11**	2	3	3	6
	**12 to 24**	14	25	22	40
	**25 to 44**	88	101	126	145
	**45 to 54**	60	144	88	212
	**55 to 64**	56	133	89	211
	**65 to 74**	35	116	63	207
	**75 plus**	29	131	56	256
**Race**	**White**	221	82	351	127
	**Black**	48	108	71	159
	**Other**	17	65	29	112
**Ethnicity**	**Hispanic**	40	76	66	130
	**Not Hispanic**	247	86	386	131
**Sex**	**Female**	131	74	213	118
	**Male**	153	92	235	140
**Total**		283	83	448	129

Source: Healthcare Cost and Utilization Project National Inpatient Sample.

Rates for age groups are unadjusted and all other rates are age-adjusted.

**Table 184:** Acute Pancreatitis: Deaths with underlying or underlying/other cause and lifetime years of life lost by age, race, ethnicity, and sex in the United States, 2019

Demographic Characteristics		Underlying Cause			Underlying/Other Cause		
		Number of Deaths	Rate per 100,000	Years of potential life lost in thousands	Number of Deaths	Rate per 100,000	Years of potential life lost in thousands
**Age (years)**	**0 to 11**	2	0.0	0.1	4	0.0	0.3
	**12 to 24**	39	0.1	2.3	67	0.1	3.9
	**25 to 44**	398	0.5	17.2	721	0.8	31.1
	**45 to 54**	335	0.8	10.3	698	1.7	21.5
	**55 to 64**	586	1.4	13.4	1,186	2.8	27.1
	**65 to 74**	599	1.9	9.4	1,216	3.9	19.2
	**75 plus**	861	3.8	6.4	1,802	8.0	13.2
**Race**	**White**	2,368	0.8	48.2	4,724	1.5	94.0
	**Black**	342	0.8	8.0	720	1.6	16.4
	**Other**	110	0.4	2.8	250	1.0	5.8
**Ethnicity**	**Hispanic**	178	0.4	4.7	409	0.9	10.4
	**Not Hispanic**	2,642	0.8	54.3	5,285	1.6	105.9
**Sex**	**Female**	1,120	0.5	21.3	2,314	1.1	44.0
	**Male**	1,700	1.0	37.7	3,380	1.9	72.3
**Total**		2,820	0.7	59.0	5,694	1.5	116.3

Source: Vital Statistics of the United States.

Rates for age groups are unadjusted and all other rates are age-adjusted.

Years of potential life lost is based on U.S. life Table 2006–2018 estimates of life expectancy at age of death.

**Table 185:** Acute Pancreatitis: Claims-based prevalence with first-listed and all-listed diagnoses by age, race-ethnicity, sex and region among privately insured enrollees, 2020

Demographic Characteristics		Number of enrollees	First-Listed Diagnosis		All-Listed Diagnoses	
			Number of enrollees with disease	Percent of enrollees with disease	Number of enrollees with disease	Percent of enrollees with disease
**Age (years)**	**0 to 11**	1,101,978	38	0.0%	60	0.0%
	**12 to 24**	1,451,892	319	0.0%	452	0.0%
	**25 to 44**	2,656,672	2,242	0.1%	3,163	0.1%
	**45 to 54**	1,452,320	2,140	0.1%	3,168	0.2%
	**55 to 64**	1,640,216	3,022	0.2%	4,874	0.3%
	**65 to 74**	2,840,615	4,546	0.2%	7,676	0.3%
	**75 plus**	2,576,241	3,895	0.2%	7,019	0.3%
**Race-ethnicity**	**White**	8,657,702	10,530	0.1%	17,037	0.2%
	**Black**	1,242,477	2,157	0.2%	3,495	0.3%
	**Hispanic**	1,493,457	1,891	0.1%	3,202	0.2%
	**Asian**	607,284	384	0.1%	613	0.1%
	**Unknown**	1,719,014	1,240	0.1%	2,065	0.1%
**Sex**	**Female**	7,199,363	8,071	0.1%	13,427	0.2%
	**Male**	6,520,571	8,131	0.1%	12,985	0.2%
**Region**	**Northeast**	1,526,275	1,682	0.1%	2,835	0.2%
	**Midwest**	3,322,846	3,583	0.1%	5,519	0.2%
	**South**	5,619,435	7,728	0.1%	12,685	0.2%
	**West**	3,251,378	3,209	0.1%	5,373	0.2%
**Total**		13,719,934	16,202	0.1%	26,412	0.2%

Source: De-identified Optum Clinformatic ^®^ Data Mart.

Enrollees with full enrollment in a commercial health plan or Medicare during the year.

**Table 186:** Acute Pancreatitis: Ambulatory care visits with first-listed and all-listed diagnoses by age, race-ethnicity, sex and region among privately insured enrollees, 2020

Demographic Characteristics		First-Listed Diagnosis		All-Listed Diagnoses	
		Number of events	Event rate per 100,000	Number of events	Event rate per 100,000
**Age (years)**	**0 to 11**	57	5	103	9
	**12 to 24**	500	34	747	51
	**25 to 44**	3,239	122	5,445	205
	**45 to 54**	2,949	203	5,122	353
	**55 to 64**	3,706	226	7,100	433
	**65 to 74**	4,545	160	9,265	326
	**75 plus**	3,875	150	8,178	317
**Race-ethnicity**	**White**	12,681	146	24,018	277
	**Black**	2,193	177	4,229	340
	**Hispanic**	2,106	141	4,104	275
	**Asian**	458	75	847	139
	**Unknown**	1,433	83	2,762	161
**Sex**	**Female**	9,178	127	17,955	249
	**Male**	9,693	149	18,005	276
**Region**	**Northeast**	1,800	118	3,619	237
	**Midwest**	4,655	140	8,089	243
	**South**	8,586	153	16,873	300
	**West**	3,830	118	7,379	227
**Total**		18,871	138	35,960	262

Source: De-identified Optum Clinformatic ^®^ Data Mart.

Enrollees with full enrollment in a commercial health plan or Medicare during the year.

**Table 187:** Acute Pancreatitis: Emergency department visits with first-listed and all-listed diagnoses by age, race-ethnicity, sex and region among privately insured enrollees, 2020

Demographic Characteristics		First-Listed Diagnosis		All-Listed Diagnoses	
		Number of events	Event rate per 100,000	Number of events	Event rate per 100,000
**Age (years)**	**0 to 11**	3	0	3	0
	**12 to 24**	13	1	16	1
	**25 to 44**	290	11	410	15
	**45 to 54**	537	37	848	58
	**55 to 64**	1,177	72	1,799	110
	**65 to 74**	2,678	94	4,235	149
	**75 plus**	2,302	89	3,788	147
**Race-ethnicity**	**White**	4,478	52	7,023	81
	**Black**	1,051	85	1,684	136
	**Hispanic**	764	51	1,257	84
	**Asian**	135	22	199	33
	**Unknown**	572	33	936	54
**Sex**	**Female**	3,632	50	5,779	80
	**Male**	3,368	52	5,320	82
**Region**	**Northeast**	869	57	1,222	80
	**Midwest**	1,420	43	2,083	63
	**South**	3,372	60	5,478	97
	**West**	1,339	41	2,316	71
**Total**		7,000	51	11,099	81

Source: De-identified Optum Clinformatic ^®^ Data Mart.

Enrollees with full enrollment in a commercial health plan or Medicare during the year.

**Table 188:** Acute Pancreatitis: Hospital discharges with first-listed and all-listed diagnoses by age, race-ethnicity, sex and region among privately insured enrollees, 2020

Demographic Characteristics		First-Listed Diagnosis		All-Listed Diagnoses	
		Number of events	Event rate per 100,000	Number of events	Event rate per 100,000
**Age (years)**	**0 to 11**	24	2	47	4
	**12 to 24**	160	11	275	19
	**25 to 44**	1,408	53	2,214	83
	**45 to 54**	1,351	93	2,183	150
	**55 to 64**	1,774	108	3,260	199
	**65 to 74**	2,562	90	5,308	187
	**75 plus**	2,139	83	5,008	194
**Race-ethnicity**	**White**	6,064	70	11,760	136
	**Black**	1,338	108	2,444	197
	**Hispanic**	1,061	71	2,222	149
	**Asian**	207	34	407	67
	**Unknown**	748	44	1,462	85
**Sex**	**Female**	4,548	63	9,065	126
	**Male**	4,870	75	9,230	142
**Region**	**Northeast**	948	62	2,008	132
	**Midwest**	2,218	67	4,034	121
	**South**	4,494	80	8,618	153
	**West**	1,758	54	3,635	112
**Total**		9,418	69	18,295	133

Source: De-identified Optum Clinformatic ^®^ Data Mart.

Enrollees with full enrollment in a commercial health plan or Medicare during the year.

**Table 189:** Acute Pancreatitis: Claims-based prevalence with first-listed and all-listed diagnoses by age, race, sex and region among fee-for-service, age-eligible Medicare beneficiaries, 2019

Demographic Characteristics		Number of enrollees	First-Listed Diagnosis		All-Listed Diagnoses	
			Number of enrollees with disease	Percent of enrollees with disease	Number of enrollees with disease	Percent of enrollees with disease
**Age (years)**	**65 to 69**	8,066,320	16,500	0.2%	26,020	0.3%
	**70 to 74**	6,936,940	15,740	0.2%	25,420	0.4%
	**75 to 79**	4,894,060	9,940	0.2%	16,660	0.3%
	**80 to 84**	3,335,340	7,680	0.2%	13,300	0.4%
	**85+**	3,578,120	8,300	0.2%	13,740	0.4%
**Race**	**White**	22,023,800	47,040	0.2%	75,520	0.3%
	**Black**	1,861,660	4,440	0.2%	7,920	0.4%
	**Other**	2,925,320	6,680	0.2%	11,700	0.4%
**Sex**	**Female**	14,975,560	30,500	0.2%	50,440	0.3%
	**Male**	11,835,220	27,660	0.2%	44,700	0.4%
**Region**	**Northeast**	4,748,700	8,820	0.2%	14,740	0.3%
	**Midwest**	6,069,800	13,340	0.2%	21,160	0.3%
	**South**	10,595,900	24,840	0.2%	40,080	0.4%
	**West**	5,396,380	11,160	0.2%	19,160	0.4%
**Total**		26,810,780	58,160	0.2%	95,140	0.4%

Source: Centers for Medicare & Medicaid Services, 5% Denominator and Claims Files.

Beneficiaries aged 65+ years with continuous and full Parts A and B enrollment and no Health Maintenance Organization enrollment during the year.

Unweighted counts were multiplied by 20 to produce counts for this table.

**Table 190:** Acute Pancreatitis: Ambulatory care visits with first-listed and all-listed diagnoses by age, race, sex and region among fee-for-service, age-eligible Medicare beneficiaries, 2019

Demographic Characteristics		First-Listed Diagnosis		All-Listed Diagnoses	
		Number of events	Event rate per 100,000	Number of events	Event rate per 100,000
**Age (years)**	**65 to 69**	14,000	174	30,100	373
	**70 to 74**	14,300	206	29,240	422
	**75 to 79**	9,320	190	18,500	378
	**80 to 84**	6,620	198	14,180	425
	**85+**	7,920	221	14,780	413
**Race**	**White**	44,560	202	88,520	402
	**Black**	2,500	134	6,360	342
	**Other**	5,100	174	11,920	407
**Sex**	**Female**	25,920	173	53,540	358
	**Male**	26,240	222	53,260	450
**Region**	**Northeast**	9,060	191	18,140	382
	**Midwest**	12,320	203	25,220	415
	**South**	21,280	201	43,080	407
	**West**	9,500	176	20,360	377
**Total**		52,160	195	106,800	398

Source: Centers for Medicare & Medicaid Services, 5% Denominator and Claims Files.

Beneficiaries aged 65+ years with continuous and full Parts A and B enrollment and no Health Maintenance Organization enrollment during the year.

Unweighted counts were multiplied by 20 to produce counts for this table.

**Table 191:** Acute Pancreatitis: Emergency department visits with first-listed and all-listed diagnoses by age, race, sex and region among fee-for-service, age-eligible Medicare beneficiaries, 2019

Demographic Characteristics		First-Listed Diagnosis		All-Listed Diagnoses	
		Number of events	Event rate per 100,000	Number of events	Event rate per 100,000
**Age (years)**	**65 to 69**	15,480	192	23,280	289
	**70 to 74**	13,920	201	21,100	304
	**75 to 79**	8,920	182	14,560	298
	**80 to 84**	6,900	207	11,640	349
	**85+**	7,700	215	12,660	354
**Race**	**White**	42,480	193	65,660	298
	**Black**	4,480	241	7,440	400
	**Other**	5,960	204	10,140	347
**Sex**	**Female**	27,700	185	43,820	293
	**Male**	25,220	213	39,420	333
**Region**	**Northeast**	8,060	170	12,620	266
	**Midwest**	11,940	197	17,860	294
	**South**	22,640	214	36,080	341
	**West**	10,280	190	16,680	309
**Total**		52,920	197	83,240	310

Source: Centers for Medicare & Medicaid Services, 5% Denominator and Claims Files.

Beneficiaries aged 65+ years with continuous and full Parts A and B enrollment and no Health Maintenance Organization enrollment during the year.

Unweighted counts were multiplied by 20 to produce counts for this table.

**Table 192:** Acute Pancreatitis: Hospital discharges with first-listed and all-listed diagnoses by age, race, sex and region among fee-for-service, age-eligible Medicare beneficiaries, 2019

Demographic Characteristics		First-Listed Diagnosis		All-Listed Diagnoses	
		Number of events	Event rate per 100,000	Number of events	Event rate per 100,000
**Age (years)**	**65 to 69**	9,840	122	17,340	215
	**70 to 74**	9,160	132	16,200	234
	**75 to 79**	5,600	114	10,920	223
	**80 to 84**	4,480	134	8,860	266
	**85+**	5,360	150	10,420	291
**Race**	**White**	28,480	129	51,340	233
	**Black**	2,460	132	4,900	263
	**Other**	3,500	120	7,500	256
**Sex**	**Female**	17,760	119	33,120	221
	**Male**	16,680	141	30,620	259
**Region**	**Northeast**	5,100	107	9,820	207
	**Midwest**	8,660	143	15,020	247
	**South**	14,700	139	26,540	250
	**West**	5,980	111	12,360	229
**Total**		34,440	128	63,740	238

Source: Centers for Medicare & Medicaid Services, 5% Denominator and Claims Files.

Beneficiaries aged 65+ years with continuous and full Parts A and B enrollment and no Health Maintenance Organization enrollment during the year.

Unweighted counts were multiplied by 20 to produce counts for this table.

**Table 193:** Chronic Pancreatitis: Ambulatory care visits with first-listed and all-listed diagnoses by age, race, ethnicity, and sex in the United States, 2015

Demographic Characteristics		First-Listed Diagnosis		All-Listed Diagnoses	
		Number in thousands	Rate per 100,000	Number in thousands	Rate per 100,000
**Age (years)**	**0 to 11**	.	.	.	.
	**12 to 24**	.	.	3	5
	**25 to 44**	16	19	54	64
	**45 to 54**	40	92	86	199
	**55 to 64**	9	22	49	119
	**65 to 74**	31	114	36	129
	**75 plus**	1	7	19	93
**Race**	**White**	86	30	228	81
	**Black**	11	27	16	37
	**Other**	.	.	2	7
**Ethnicity**	**Hispanic**	74	168	91	206
	**Not Hispanic**	23	9	154	52
**Sex**	**Female**	40	28	107	67
	**Male**	57	30	138	80
**Total**		97	28	245	71

Source: National Ambulatory Medical Care Survey (3-year average, 2014–2016).

Rates for age groups are unadjusted and all other rates are age-adjusted.

**Table 194:** Chronic Pancreatitis: Emergency department visits with first-listed and all-listed diagnoses by age and sex in the United States, 2018

Demographic Characteristics		First-Listed Diagnosis		All-Listed Diagnoses	
		Number in thousands	Rate per 100,000	Number in thousands	Rate per 100,000
**Age (years)**	**0 to 11**	0	0	1	2
	**12 to 24**	1	2	6	10
	**25 to 44**	15	17	78	90
	**45 to 54**	10	25	69	165
	**55 to 64**	8	19	62	147
	**65 to 74**	2	8	29	94
	**75 plus**	1	4	19	85
**Sex**	**Female**	16	10	114	65
	**Male**	22	13	149	89
**Total**		38	12	263	77

Source: Healthcare Cost and Utilization Project Nationwide Emergency Department Sample.

Rates for age groups are unadjusted and all other rates are age-adjusted.

**Table 195:** Chronic Pancreatitis: Hospital discharges with first-listed and all-listed diagnoses by age, race, ethnicity, and sex in the United States, 2018

Demographic Characteristics		First-Listed Diagnosis		All-Listed Diagnoses	
		Number in thousands	Rate per 100,000	Number in thousands	Rate per 100,000
**Age (years)**	**0 to 11**	0	0	1	2
	**12 to 24**	1	1	5	8
	**25 to 44**	3	4	51	58
	**45 to 54**	3	7	47	112
	**55 to 64**	2	5	47	112
	**65 to 74**	1	4	25	83
	**75 plus**	1	3	17	76
**Race**	**White**	8	3	143	52
	**Black**	2	5	42	93
	**Other**	1	2	9	34
**Ethnicity**	**Hispanic**	1	2	18	35
	**Not Hispanic**	10	4	176	60
**Sex**	**Female**	5	3	84	47
	**Male**	6	3	109	64
**Total**		11	3	192	55

Source: Healthcare Cost and Utilization Project National Inpatient Sample.

Rates for age groups are unadjusted and all other rates are age-adjusted.

**Table 196:** Chronic Pancreatitis: Deaths with underlying or underlying/other cause and lifetime years of life lost by age, race, ethnicity, and sex in the United States, 2019

Demographic Characteristics		Underlying Cause			Underlying/Other Cause		
		Number of Deaths	Rate per 100,000	Years of potential life lost in thousands	Number of Deaths	Rate per 100,000	Years of potential life lost in thousands
**Age (years)**	**0 to 11**	.	.	.	4	0.0	0.3
	**12 to 24**	4	0.0	0.2	10	0.0	0.6
	**25 to 44**	44	0.1	1.9	193	0.2	8.2
	**45 to 54**	66	0.2	2.0	274	0.7	8.4
	**55 to 64**	118	0.3	2.7	458	1.1	10.5
	**65 to 74**	75	0.2	1.2	317	1.0	5.0
	**75 plus**	112	0.5	0.8	337	1.5	2.5
**Race**	**White**	324	0.1	6.7	1,259	0.4	28.0
	**Black**	86	0.2	1.8	291	0.6	6.3
	**Other**	9	0.0	0.2	43	0.2	1.2
**Ethnicity**	**Hispanic**	23	0.0	0.6	64	0.1	1.7
	**Not Hispanic**	396	0.1	8.2	1,529	0.5	33.9
**Sex**	**Female**	156	0.1	3.3	640	0.3	14.8
	**Male**	263	0.1	5.5	953	0.5	20.7
**Total**		419	0.1	8.8	1,593	0.4	35.5

Source: Vital Statistics of the United States.

Rates for age groups are unadjusted and all other rates are age-adjusted.

Years of potential life lost is based on U.S. life Table 2006–2018 estimates of life expectancy at age of death.

**Table 197:** Chronic Pancreatitis: Claims-based prevalence with first-listed and all-listed diagnoses by age, race-ethnicity, sex and region among privately insured enrollees, 2020

Demographic Characteristics		Number of enrollees	First-Listed Diagnosis		All-Listed Diagnoses	
			Number of enrollees with disease	Percent of enrollees with disease	Number of enrollees with disease	Percent of enrollees with disease
**Age (years)**	**0 to 11**	1,101,978	9	0.0%	15	0.0%
	**12 to 24**	1,451,892	54	0.0%	110	0.0%
	**25 to 44**	2,656,672	484	0.0%	1,168	0.0%
	**45 to 54**	1,452,320	669	0.0%	1,704	0.1%
	**55 to 64**	1,640,216	1,157	0.1%	3,288	0.2%
	**65 to 74**	2,840,615	1,530	0.1%	4,823	0.2%
	**75 plus**	2,576,241	963	0.0%	4,092	0.2%
**Race-ethnicity**	**White**	8,657,702	3,269	0.0%	9,973	0.1%
	**Black**	1,242,477	705	0.1%	2,201	0.2%
	**Hispanic**	1,493,457	404	0.0%	1,520	0.1%
	**Asian**	607,284	131	0.0%	368	0.1%
	**Unknown**	1,719,014	357	0.0%	1,138	0.1%
**Sex**	**Female**	7,199,363	2,364	0.0%	7,456	0.1%
	**Male**	6,520,571	2,502	0.0%	7,744	0.1%
**Region**	**Northeast**	1,526,275	532	0.0%	1,579	0.1%
	**Midwest**	3,322,846	1,101	0.0%	3,151	0.1%
	**South**	5,619,435	2,312	0.0%	7,271	0.1%
	**West**	3,251,378	921	0.0%	3,199	0.1%
**Total**		13,719,934	4,866	0.0%	15,200	0.1%

Source: De-identified Optum Clinformatic ^®^ Data Mart.

Enrollees with full enrollment in a commercial health plan or Medicare during the year.

**Table 198:** Chronic Pancreatitis: Ambulatory care visits with first-listed and all-listed diagnoses by age, race-ethnicity, sex and region among privately insured enrollees, 2020

Demographic Characteristics		First-Listed Diagnosis		All-Listed Diagnoses	
		Number of events	Event rate per 100,000	Number of events	Event rate per 100,000
**Age (years)**	**0 to 11**	9	1	25	2
	**12 to 24**	84	6	211	15
	**25 to 44**	1,006	38	2,884	109
	**45 to 54**	1,429	98	4,514	311
	**55 to 64**	2,269	138	7,853	479
	**65 to 74**	2,453	86	9,100	320
	**75 plus**	1,468	57	6,576	255
**Race-ethnicity**	**White**	5,832	67	20,509	237
	**Black**	1,253	101	4,667	376
	**Hispanic**	711	48	2,906	195
	**Asian**	242	40	703	116
	**Unknown**	680	40	2,378	138
**Sex**	**Female**	4,342	60	15,938	221
	**Male**	4,376	67	15,225	233
**Region**	**Northeast**	866	57	2,994	196
	**Midwest**	2,044	62	6,668	201
	**South**	4,187	75	14,983	267
	**West**	1,621	50	6,518	200
**Total**		8,718	64	31,163	227

Source: De-identified Optum Clinformatic ^®^ Data Mart.

Enrollees with full enrollment in a commercial health plan or Medicare during the year.

**Table 199:** Chronic Pancreatitis: Emergency department visits with first-listed and all-listed diagnoses by age, race-ethnicity, sex and region among privately insured enrollees, 2020

Demographic Characteristics		First-Listed Diagnosis		All-Listed Diagnoses	
		Number of events	Event rate per 100,000	Number of events	Event rate per 100,000
**Age (years)**	**0 to 11**	1	0	1	0
	**12 to 24**	.	.	1	0
	**25 to 44**	58	2	119	4
	**45 to 54**	114	8	264	18
	**55 to 64**	202	12	455	28
	**65 to 74**	155	5	395	14
	**75 plus**	50	2	177	7
**Race-ethnicity**	**White**	330	4	862	10
	**Black**	146	12	294	24
	**Hispanic**	41	3	116	8
	**Asian**	5	1	14	2
	**Unknown**	58	3	126	7
**Sex**	**Female**	249	3	631	9
	**Male**	331	5	781	12
**Region**	**Northeast**	52	3	157	10
	**Midwest**	121	4	270	8
	**South**	335	6	760	14
	**West**	72	2	225	7
**Total**		580	4	1,412	10

Source: De-identified Optum Clinformatic ^®^ Data Mart.

Enrollees with full enrollment in a commercial health plan or Medicare during the year.

**Table 200:** Chronic Pancreatitis: Hospital discharges with first-listed and all-listed diagnoses by age, race-ethnicity, sex and region among privately insured enrollees, 2020

Demographic Characteristics		First-Listed Diagnosis		All-Listed Diagnoses	
		Number of events	Event rate per 100,000	Number of events	Event rate per 100,000
**Age (years)**	**0 to 11**	1	0	9	1
	**12 to 24**	4	0	58	4
	**25 to 44**	34	1	746	28
	**45 to 54**	51	4	1,046	72
	**55 to 64**	78	5	1,893	115
	**65 to 74**	75	3	2,211	78
	**75 plus**	22	1	1,611	63
**Race-ethnicity**	**White**	169	2	4,784	55
	**Black**	54	4	1,369	110
	**Hispanic**	23	2	700	47
	**Asian**	3	0	117	19
	**Unknown**	16	1	604	35
**Sex**	**Female**	140	2	3,521	49
	**Male**	125	2	4,053	62
**Region**	**Northeast**	28	2	819	54
	**Midwest**	62	2	1,726	52
	**South**	125	2	3,708	66
	**West**	50	2	1,321	41
**Total**		265	2	7,574	55

Source: De-identified Optum Clinformatic ^®^ Data Mart.

Enrollees with full enrollment in a commercial health plan or Medicare during the year.

**Table 201:** Chronic Pancreatitis: Claims-based prevalence with first-listed and all-listed diagnoses by age, race, sex and region among fee-for-service, age-eligible Medicare beneficiaries, 2019

Demographic Characteristics		Number of enrollees	First-Listed Diagnosis		All-Listed Diagnoses	
			Number of enrollees with disease	Percent of enrollees with disease	Number of enrollees with disease	Percent of enrollees with disease
**Age (years)**	**65 to 69**	8,066,320	5,540	0.1%	16,180	0.2%
	**70 to 74**	6,936,940	4,700	0.1%	13,000	0.2%
	**75 to 79**	4,894,060	2,940	0.1%	9,740	0.2%
	**80 to 84**	3,335,340	1,780	0.1%	6,740	0.2%
	**85+**	3,578,120	1,640	0.0%	6,260	0.2%
**Race**	**White**	22,023,800	13,880	0.1%	41,980	0.2%
	**Black**	1,861,660	1,380	0.1%	5,160	0.3%
	**Other**	2,925,320	1,340	0.0%	4,780	0.2%
**Sex**	**Female**	14,975,560	7,920	0.1%	26,220	0.2%
	**Male**	11,835,220	8,680	0.1%	25,700	0.2%
**Region**	**Northeast**	4,748,700	2,600	0.1%	9,020	0.2%
	**Midwest**	6,069,800	3,240	0.1%	11,400	0.2%
	**South**	10,595,900	7,660	0.1%	22,200	0.2%
	**West**	5,396,380	3,100	0.1%	9,300	0.2%
**Total**		26,810,780	16,600	0.1%	51,920	0.2%

Source: Centers for Medicare & Medicaid Services, 5% Denominator and Claims Files.

Beneficiaries aged 65+ years with continuous and full Parts A and B enrollment and no Health Maintenance Organization enrollment during the year.

Unweighted counts were multiplied by 20 to produce counts for this table.

**Table 202:** Chronic Pancreatitis: Ambulatory care visits with first-listed and all-listed diagnoses by age, race, sex and region among fee-for-service, age-eligible Medicare beneficiaries, 2019

Demographic Characteristics		First-Listed Diagnosis		All-Listed Diagnoses	
		Number of events	Event rate per 100,000	Number of events	Event rate per 100,000
**Age (years)**	**65 to 69**	9,360	116	30,540	379
	**70 to 74**	7,120	103	23,280	336
	**75 to 79**	5,100	104	18,880	386
	**80 to 84**	2,380	71	11,140	334
	**85+**	1,840	51	7,660	214
**Race**	**White**	21,900	99	74,580	339
	**Black**	1,760	95	8,920	479
	**Other**	2,140	73	8,000	273
**Sex**	**Female**	12,360	83	47,000	314
	**Male**	13,440	114	44,500	376
**Region**	**Northeast**	3,600	76	14,200	299
	**Midwest**	6,040	100	21,100	348
	**South**	11,420	108	40,020	378
	**West**	4,740	88	16,180	300
**Total**		25,800	96	91,500	341

Source: Centers for Medicare & Medicaid Services, 5% Denominator and Claims Files.

Beneficiaries aged 65+ years with continuous and full Parts A and B enrollment and no Health Maintenance Organization enrollment during the year.

Unweighted counts were multiplied by 20 to produce counts for this table.

**Table 203:** Chronic Pancreatitis: Emergency department visits with first-listed and all-listed diagnoses by age, race, sex and region among fee-for-service, age-eligible Medicare beneficiaries, 2019

Demographic Characteristics		First-Listed Diagnosis		All-Listed Diagnoses	
		Number of events	Event rate per 100,000	Number of events	Event rate per 100,000
**Age (years)**	**65 to 69**	1,380	17	10,100	125
	**70 to 74**	680	10	6,300	91
	**75 to 79**	.	.	4,820	98
	**80 to 84**	.	.	3,100	93
	**85+**	260	7	3,760	105
**Race**	**White**	1,840	8	21,300	97
	**Black**	.	.	4,560	245
	**Other**	.	.	2,220	76
**Sex**	**Female**	1,380	9	13,960	93
	**Male**	1,400	12	14,120	119
**Region**	**Northeast**	240	5	4,460	94
	**Midwest**	580	10	7,040	116
	**South**	1,340	13	11,360	107
	**West**	620	11	5,220	97
**Total**		2,780	10	28,080	105

Source: Centers for Medicare & Medicaid Services, 5% Denominator and Claims Files.

Beneficiaries aged 65+ years with continuous and full Parts A and B enrollment and no Health Maintenance Organization enrollment during the year.

Unweighted counts were multiplied by 20 to produce counts for this table.

**Table 204:** Chronic Pancreatitis: Hospital discharges with first-listed and all-listed diagnoses by age, race, sex and region among fee-for-service, age-eligible Medicare beneficiaries, 2019

Demographic Characteristics		First-Listed Diagnosis		All-Listed Diagnoses	
		Number of events	Event rate per 100,000	Number of events	Event rate per 100,000
**Age (years)**	**65 to 69**	360	4	8,280	103
	**70 to 74**	280	4	5,940	86
	**75 to 79**	.	.	4,800	98
	**80 to 84**	.	.	2,480	74
	**85+**	.	.	3,000	84
**Race**	**White**	640	3	19,100	87
	**Black**	.	.	3,520	189
	**Other**	.	.	1,880	64
**Sex**	**Female**	540	4	12,340	82
	**Male**	340	3	12,160	103
**Region**	**Northeast**	.	.	3,880	82
	**Midwest**	.	.	6,480	107
	**South**	440	4	10,040	95
	**West**	.	.	4,100	76
**Total**		880	3	24,500	91

Source: Centers for Medicare & Medicaid Services, 5% Denominator and Claims Files.

Beneficiaries aged 65+ years with continuous and full Parts A and B enrollment and no Health Maintenance Organization enrollment during the year.

Unweighted counts were multiplied by 20 to produce counts for this table.

**Table 205:** Celiac Disease: Ambulatory care visits with first-listed and all-listed diagnoses by age, race, ethnicity, and sex in the United States, 2015

Demographic Characteristics		First-Listed Diagnosis		All-Listed Diagnoses	
		Number in thousands	Rate per 100,000	Number in thousands	Rate per 100,000
**Age (years)**	**0 to 11**	5	9	122	251
	**12 to 24**	11	19	37	65
	**25 to 44**	2	2	138	164
	**45 to 54**	18	42	80	186
	**55 to 64**	13	31	192	470
	**65 to 74**	8	30	19	70
	**75 plus**	7	35	22	109
**Race**	**White**	63	23	507	199
	**Black**	.	.	44	80
	**Other**	.	.	59	257
**Ethnicity**	**Hispanic**	3	6	118	195
	**Not Hispanic**	60	21	493	180
**Sex**	**Female**	47	27	390	247
	**Male**	16	10	220	126
**Total**		63	18	611	187

Source: National Ambulatory Medical Care Survey (3-year average, 2014–2016).

Rates for age groups are unadjusted and all other rates are age-adjusted.

**Table 206:** Celiac Disease: Emergency department visits with first-listed and all-listed diagnoses by age and sex in the United States, 2018

Demographic Characteristics		First-Listed Diagnosis		All-Listed Diagnoses	
		Number in thousands	Rate per 100,000	Number in thousands	Rate per 100,000
**Age (years)**	**0 to 11**	0	0	3	5
	**12 to 24**	0	1	10	18
	**25 to 44**	0	0	18	21
	**45 to 54**	0	0	9	21
	**55 to 64**	0	0	9	22
	**65 to 74**	0	0	8	26
	**75 plus**	0	1	11	51
**Sex**	**Female**	1	1	50	29
	**Male**	0	0	18	11
**Total**		1	0	68	20

Source: Healthcare Cost and Utilization Project Nationwide Emergency Department Sample.

Rates for age groups are unadjusted and all other rates are age-adjusted.

**Table 207:** Celiac Disease: Hospital discharges with first-listed and all-listed diagnoses by age, race, ethnicity, and sex in the United States, 2018

Demographic Characteristics		First-Listed Diagnosis		All-Listed Diagnoses	
		Number in thousands	Rate per 100,000	Number in thousands	Rate per 100,000
**Age (years)**	**0 to 11**	0	0	1	2
	**12 to 24**	0	0	4	8
	**25 to 44**	0	0	10	11
	**45 to 54**	0	0	5	12
	**55 to 64**	0	0	6	15
	**65 to 74**	0	0	7	23
	**75 plus**	0	0	9	41
**Race**	**White**	1	0	40	14
	**Black**	0	0	2	3
	**Other**	0	0	1	4
**Ethnicity**	**Hispanic**	0	0	2	4
	**Not Hispanic**	1	0	40	13
**Sex**	**Female**	0	0	30	16
	**Male**	0	0	13	7
**Total**		1	0	42	12

Source: Healthcare Cost and Utilization Project National Inpatient Sample.

Rates for age groups are unadjusted and all other rates are age-adjusted.

**Table 208:** Celiac Disease: Deaths with underlying or underlying/other cause and lifetime years of life lost by age, race, ethnicity, and sex in the United States, 2019

Demographic Characteristics		Underlying Cause			Underlying/Other Cause		
		Number of Deaths	Rate per 100,000	Years of potential life lost in thousands	Number of Deaths	Rate per 100,000	Years of potential life lost in thousands
**Age (years)**	**0 to 11**	.	.	.	1	0.0	0.1
	**12 to 24**	.	.	.	.	.	.
	**25 to 44**	2	0.0	0.1	4	0.0	0.2
	**45 to 54**	1	0.0	0.0	5	0.0	0.2
	**55 to 64**	3	0.0	0.1	26	0.1	0.6
	**65 to 74**	10	0.0	0.2	44	0.1	0.7
	**75 plus**	28	0.1	0.2	128	0.6	0.8
**Race**	**White**	42	0.0	0.5	202	0.1	2.4
	**Black**	1	0.0	0.0	2	0.0	0.0
	**Other**	1	0.0	0.0	4	0.0	0.1
**Ethnicity**	**Hispanic**	2	0.0	0.1	6	0.0	0.1
	**Not Hispanic**	42	0.0	0.5	202	0.1	2.4
**Sex**	**Female**	25	0.0	0.3	130	0.1	1.5
	**Male**	19	0.0	0.3	78	0.0	1.1
**Total**		44	0.0	0.6	208	0.1	2.6

Source: Vital Statistics of the United States.

Rates for age groups are unadjusted and all other rates are age-adjusted.

Years of potential life lost is based on U.S. life Table 2006–2018 estimates of life expectancy at age of death.

**Table 209:** Celiac Disease: Claims-based prevalence with first-listed and all-listed diagnoses by age, race-ethnicity, sex and region among privately insured enrollees, 2020

Demographic Characteristics		Number of enrollees	First-Listed Diagnosis		All-Listed Diagnoses	
			Number of enrollees with disease	Percent of enrollees with disease	Number of enrollees with disease	Percent of enrollees with disease
**Age (years)**	**0 to 11**	1,101,978	492	0.0%	904	0.1%
	**12 to 24**	1,451,892	1,012	0.1%	2,476	0.2%
	**25 to 44**	2,656,672	1,072	0.0%	3,555	0.1%
	**45 to 54**	1,452,320	554	0.0%	2,163	0.1%
	**55 to 64**	1,640,216	564	0.0%	2,299	0.1%
	**65 to 74**	2,840,615	954	0.0%	3,555	0.1%
	**75 plus**	2,576,241	587	0.0%	2,909	0.1%
**Race-ethnicity**	**White**	8,657,702	4,083	0.0%	14,136	0.2%
	**Black**	1,242,477	203	0.0%	710	0.1%
	**Hispanic**	1,493,457	351	0.0%	1,178	0.1%
	**Asian**	607,284	89	0.0%	298	0.0%
	**Unknown**	1,719,014	509	0.0%	1,539	0.1%
**Sex**	**Female**	7,199,363	3,588	0.0%	12,546	0.2%
	**Male**	6,520,571	1,647	0.0%	5,315	0.1%
**Region**	**Northeast**	1,526,275	976	0.1%	2,948	0.2%
	**Midwest**	3,322,846	1,353	0.0%	4,605	0.1%
	**South**	5,619,435	1,710	0.0%	5,957	0.1%
	**West**	3,251,378	1,196	0.0%	4,351	0.1%
**Total**		13,719,934	5,235	0.0%	17,861	0.1%

Source: De-identified Optum Clinformatic ^®^ Data Mart.

Enrollees with full enrollment in a commercial health plan or Medicare during the year.

**Table 210:** Celiac Disease: Ambulatory care visits with first-listed and all-listed diagnoses by age, race-ethnicity, sex and region among privately insured enrollees, 2020

Demographic Characteristics		First-Listed Diagnosis		All-Listed Diagnoses	
		Number of events	Event rate per 100,000	Number of events	Event rate per 100,000
**Age (years)**	**0 to 11**	710	64	1,666	151
	**12 to 24**	1,405	97	4,473	308
	**25 to 44**	1,448	55	5,917	223
	**45 to 54**	713	49	3,727	257
	**55 to 64**	743	45	3,947	241
	**65 to 74**	1,296	46	6,340	223
	**75 plus**	786	31	5,350	208
**Race-ethnicity**	**White**	5,516	64	24,758	286
	**Black**	280	23	1,268	102
	**Hispanic**	488	33	2,251	151
	**Asian**	128	21	460	76
	**Unknown**	689	40	2,683	156
**Sex**	**Female**	4,857	67	22,158	308
	**Male**	2,244	34	9,262	142
**Region**	**Northeast**	1,328	87	5,253	344
	**Midwest**	1,798	54	7,704	232
	**South**	2,338	42	10,788	192
	**West**	1,637	50	7,675	236
**Total**		7,101	52	31,420	229

Source: De-identified Optum Clinformatic ^®^ Data Mart.

Enrollees with full enrollment in a commercial health plan or Medicare during the year.

**Table 211:** Celiac Disease: Emergency department visits with first-listed and all-listed diagnoses by age, race-ethnicity, sex and region among privately insured enrollees, 2020

Demographic Characteristics		First-Listed Diagnosis		All-Listed Diagnoses	
		Number of events	Event rate per 100,000	Number of events	Event rate per 100,000
**Age (years)**	**0 to 11**	2	0	3	0
	**12 to 24**	5	0	25	2
	**25 to 44**	12	0	81	3
	**45 to 54**	2	0	9	1
	**55 to 64**	5	0	15	1
	**65 to 74**	5	0	43	2
	**75 plus**	4	0	27	1
**Race-ethnicity**	**White**	29	0	171	2
	**Black**	3	0	6	0
	**Hispanic**	2	0	6	0
	**Asian**	.	.	3	0
	**Unknown**	1	0	17	1
**Sex**	**Female**	28	0	163	2
	**Male**	7	0	40	1
**Region**	**Northeast**	8	1	42	3
	**Midwest**	8	0	50	2
	**South**	9	0	55	1
	**West**	10	0	56	2
**Total**		35	0	203	1

Source: De-identified Optum Clinformatic ^®^ Data Mart.

Enrollees with full enrollment in a commercial health plan or Medicare during the year.

**Table 212:** Celiac Disease: Hospital discharges with first-listed and all-listed diagnoses by age, race-ethnicity, sex and region among privately insured enrollees, 2020

Demographic Characteristics		First-Listed Diagnosis		All-Listed Diagnoses	
		Number of events	Event rate per 100,000	Number of events	Event rate per 100,000
**Age (years)**	**0 to 11**	2	0	12	1
	**12 to 24**	3	0	157	11
	**25 to 44**	.	.	302	11
	**45 to 54**	2	0	139	10
	**55 to 64**	4	0	276	17
	**65 to 74**	7	0	459	16
	**75 plus**	3	0	769	30
**Race-ethnicity**	**White**	14	0	1,691	20
	**Black**	.	.	122	10
	**Hispanic**	2	0	113	8
	**Asian**	.	.	29	5
	**Unknown**	5	0	159	9
**Sex**	**Female**	11	0	1,480	21
	**Male**	10	0	634	10
**Region**	**Northeast**	3	0	310	20
	**Midwest**	7	0	609	18
	**South**	6	0	674	12
	**West**	5	0	521	16
**Total**		21	0	2,114	15

Source: De-identified Optum Clinformatic ^®^ Data Mart.

Enrollees with full enrollment in a commercial health plan or Medicare during the year.

**Table 213:** Celiac Disease: Claims-based prevalence with first-listed and all-listed diagnoses by age, race, sex and region among fee-for-service, age-eligible Medicare beneficiaries, 2019

Demographic Characteristics		Number of enrollees	First-Listed Diagnosis		All-Listed Diagnoses	
			Number of enrollees with disease	Percent of enrollees with disease	Number of enrollees with disease	Percent of enrollees with disease
**Age (years)**	**65 to 69**	8,066,320	3,400	0.0%	13,660	0.2%
	**70 to 74**	6,936,940	3,200	0.0%	12,720	0.2%
	**75 to 79**	4,894,060	2,720	0.1%	9,500	0.2%
	**80 to 84**	3,335,340	1,840	0.1%	7,220	0.2%
	**85+**	3,578,120	940	0.0%	4,880	0.1%
**Race**	**White**	22,023,800	11,320	0.1%	44,340	0.2%
	**Black**	1,861,660	.	.	680	0.0%
	**Other**	2,925,320	.	.	2,960	0.1%
**Sex**	**Female**	14,975,560	8,560	0.1%	32,980	0.2%
	**Male**	11,835,220	3,540	0.0%	15,000	0.1%
**Region**	**Northeast**	4,748,700	3,380	0.1%	12,320	0.3%
	**Midwest**	6,069,800	2,540	0.0%	11,100	0.2%
	**South**	10,595,900	4,100	0.0%	15,760	0.1%
	**West**	5,396,380	2,080	0.0%	8,800	0.2%
**Total**		26,810,780	12,100	0.0%	47,980	0.2%

Source: Centers for Medicare & Medicaid Services, 5% Denominator and Claims Files.

Beneficiaries aged 65+ years with continuous and full Parts A and B enrollment and no Health Maintenance Organization enrollment during the year.

Unweighted counts were multiplied by 20 to produce counts for this table.

**Table 214:** Celiac Disease: Ambulatory care visits with first-listed and all-listed diagnoses by age, race, sex and region among fee-for-service, age-eligible Medicare beneficiaries, 2019

Demographic Characteristics}		First-Listed Diagnosis}		All-Listed Diagnoses}	
		Number of events}	Event rate per 100,000}	Number of events}	Event rate per 100,000}
**Age (years)**	**65 to 69**	4,720	59	25,400	315
	**70 to 74**	4,900	71	23,900	345
	**75 to 79**	3,620	74	17,200	351
	**80 to 84**	2,680	80	12,480	374
	**85+**	1,300	36	10,320	288
**Race**	**White**	16,060	73	82,580	375
	**Black**	.	.	1,420	76
	**Other**	.	.	5,300	181
**Sex**	**Female**	12,440	83	62,380	417
	**Male**	4,780	40	26,920	227
**Region**	**Northeast**	4,800	101	23,200	489
	**Midwest**	3,380	56	18,980	313
	**South**	5,960	56	31,380	296
	**West**	3,080	57	15,740	292
**Total**		17,220	64	89,300	333

Source: Centers for Medicare & Medicaid Services, 5% Denominator and Claims Files.

Beneficiaries aged 65+ years with continuous and full Parts A and B enrollment and no Health Maintenance Organization enrollment during the year.

Unweighted counts were multiplied by 20 to produce counts for this table.

**Table 215:** Celiac Disease: Emergency department visits with first-listed and all-listed diagnoses by age, race, sex and region among fee-for-service, age-eligible Medicare beneficiaries, 2019

Demographic Characteristics		First-Listed Diagnosis		All-Listed Diagnoses	
		Number of events	Event rate per 100,000	Number of events	Event rate per 100,000
**Age (years)**	**65 to 69**	.	.	2,580	32
	**70 to 74**	.	.	3,140	45
	**75 to 79**	.	.	3,300	67
	**80 to 84**	.	.	2,780	83
	**85+**	.	.	2,400	67
**Race**	**White**	.	.	13,260	60
	**Black**	.	.	.	.
	**Other**	.	.	.	.
**Sex**	**Female**	.	.	10,240	68
	**Male**	.	.	3,960	33
**Region**	**Northeast**	.	.	3,540	75
	**Midwest**	.	.	2,940	48
	**South**	.	.	5,100	48
	**West**	.	.	2,620	49
**Total**		.	.	14,200	53

Source: Centers for Medicare & Medicaid Services, 5% Denominator and Claims Files.

Beneficiaries aged 65+ years with continuous and full Parts A and B enrollment and no Health Maintenance Organization enrollment during the year.

Unweighted counts were multiplied by 20 to produce counts for this table.

**Table 216:** Celiac Disease: Hospital discharges with first-listed and all-listed diagnoses by age, race, sex and region among fee-for-service, age-eligible Medicare beneficiaries, 2019

Demographic Characteristics		First-Listed Diagnosis		All-Listed Diagnoses	
		Number of events	Event rate per 100,000	Number of events	Event rate per 100,000
**Age (years)**	**65 to 69**	.	.	2,160	27
	**70 to 74**	.	.	2,560	37
	**75 to 79**	.	.	2,560	52
	**80 to 84**	.	.	1,940	58
	**85+**	.	.	2,140	60
**Race**	**White**	.	.	10,580	48
	**Black**	.	.	.	.
	**Other**	.	.	.	.
**Sex**	**Female**	.	.	7,880	53
	**Male**	.	.	3,480	29
**Region**	**Northeast**	.	.	2,960	62
	**Midwest**	.	.	2,420	40
	**South**	.	.	3,820	36
	**West**	.	.	2,160	40
**Total**		.	.	11,360	42

Source: Centers for Medicare & Medicaid Services, 5% Denominator and Claims Files.

Beneficiaries aged 65+ years with continuous and full Parts A and B enrollment and no Health Maintenance Organization enrollment during the year.

Unweighted counts were multiplied by 20 to produce counts for this table.

**Table 217:** Gastrointestinal Infections (exc. C. difficile): Ambulatory care visits with first-listed and all-listed diagnoses by age, race, ethnicity, and sex in the United States, 2015

Demographic Characteristics		First-Listed Diagnosis		All-Listed Diagnoses	
		Number in thousands	Rate per 100,000	Number in thousands	Rate per 100,000
**Age (years)**	**0 to 11**	339	696	468	962
	**12 to 24**	323	576	377	671
	**25 to 44**	270	320	318	377
	**45 to 54**	84	196	123	286
	**55 to 64**	119	291	167	410
	**65 to 74**	37	135	108	393
	**75 plus**	77	382	81	400
**Race**	**White**	940	394	1,257	519
	**Black**	286	627	354	786
	**Other**	24	104	31	136
**Ethnicity**	**Hispanic**	217	394	316	551
	**Not Hispanic**	1,033	422	1,327	528
**Sex**	**Female**	692	455	952	608
	**Male**	557	368	690	454
**Total**		1,250	408	1,642	527

Source: National Ambulatory Medical Care Survey (3-year average, 2014–2016).

Rates for age groups are unadjusted and all other rates are age-adjusted.

**Table 218:** Gastrointestinal Infections (exc. C. difficile): Emergency department visits with first-listed and all-listed diagnoses by age and sex in the United States, 2018

Demographic Characteristics		First-Listed Diagnosis		All-Listed Diagnoses	
		Number in thousands	Rate per 100,000	Number in thousands	Rate per 100,000
**Age (years)**	**0 to 11**	174	360	208	429
	**12 to 24**	83	149	101	182
	**25 to 44**	108	124	143	164
	**45 to 54**	40	97	60	144
	**55 to 64**	38	90	63	149
	**65 to 74**	32	106	57	185
	**75 plus**	35	161	67	304
**Sex**	**Female**	287	179	394	239
	**Male**	224	144	304	193
**Total**		510	162	698	217

Source: Healthcare Cost and Utilization Project Nationwide Emergency Department Sample.

Rates for age groups are unadjusted and all other rates are age-adjusted.

**Table 219:** Gastrointestinal Infections (exc. C. difficile): Hospital discharges with first-listed and all-listed diagnoses by age, race, ethnicity, and sex in the United States, 2018

Demographic Characteristics		First-Listed Diagnosis		All-Listed Diagnoses	
		Number in thousands	Rate per 100,000	Number in thousands	Rate per 100,000
**Age (years)**	**0 to 11**	16	32	31	64
	**12 to 24**	7	12	14	25
	**25 to 44**	16	18	36	42
	**45 to 54**	13	30	28	68
	**55 to 64**	16	39	39	92
	**65 to 74**	17	55	40	133
	**75 plus**	21	97	51	231
**Race**	**White**	85	30	190	67
	**Black**	14	32	35	78
	**Other**	7	29	17	66
**Ethnicity**	**Hispanic**	16	30	34	65
	**Not Hispanic**	91	31	208	69
**Sex**	**Female**	63	34	137	74
	**Male**	43	26	102	61
**Total**		106	30	240	68

Source: Healthcare Cost and Utilization Project National Inpatient Sample.

Rates for age groups are unadjusted and all other rates are age-adjusted.

**Table 220:** Gastrointestinal Infections (exc. C. difficile): Deaths with underlying or underlying/other cause and lifetime years of life lost by age, race, ethnicity, and sex in the United States, 2019

Demographic Characteristics		Underlying Cause			Underlying/Other Cause		
		Number of Deaths	Rate per 100,000	Years of potential life lost in thousands	Number of Deaths	Rate per 100,000	Years of potential life lost in thousands
**Age (years)**	**0 to 11**	215	0.4	16.5	279	0.6	21.4
	**12 to 24**	9	0.0	0.6	19	0.0	1.2
	**25 to 44**	70	0.1	3.1	146	0.2	6.4
	**45 to 54**	112	0.3	3.5	235	0.6	7.3
	**55 to 64**	313	0.7	7.2	632	1.5	14.6
	**65 to 74**	537	1.7	8.5	1,060	3.4	16.8
	**75 plus**	1,580	7.0	10.3	2,905	12.9	19.4
**Race**	**White**	2,398	0.7	36.9	4,519	1.3	66.9
	**Black**	332	0.8	10.5	566	1.3	16.1
	**Other**	106	0.4	2.2	191	0.8	4.0
**Ethnicity**	**Hispanic**	237	0.6	6.3	402	1.0	9.7
	**Not Hispanic**	2,599	0.7	43.3	4,874	1.4	77.2
**Sex**	**Female**	1,701	0.8	27.5	3,103	1.4	48.1
	**Male**	1,135	0.7	22.1	2,173	1.3	38.9
**Total**		2,836	0.7	49.6	5,276	1.3	87.0

Source: Vital Statistics of the United States.

Rates for age groups are unadjusted and all other rates are age-adjusted.

Years of potential life lost is based on U.S. life Table 2006–2018 estimates of life expectancy at age of death.

**Table 221:** Gastrointestinal Infections (exc. C. difficile): Claims-based prevalence with first-listed and all-listed diagnoses by age, race-ethnicity, sex and region among privately insured enrollees, 2020

Demographic Characteristics		Number of enrollees	First-Listed Diagnosis		All-Listed Diagnoses	
			Number of enrollees with disease	Percent of enrollees with disease	Number of enrollees with disease	Percent of enrollees with disease
**Age (years)**	**0 to 11**	1,101,978	6,863	0.6%	9,069	0.8%
	**12 to 24**	1,451,892	4,595	0.3%	6,335	0.4%
	**25 to 44**	2,656,672	7,506	0.3%	11,473	0.4%
	**45 to 54**	1,452,320	3,713	0.3%	6,703	0.5%
	**55 to 64**	1,640,216	4,038	0.2%	7,891	0.5%
	**65 to 74**	2,840,615	6,251	0.2%	13,250	0.5%
	**75 plus**	2,576,241	5,661	0.2%	12,428	0.5%
**Race-ethnicity**	**White**	8,657,702	21,074	0.2%	37,052	0.4%
	**Black**	1,242,477	3,801	0.3%	7,231	0.6%
	**Hispanic**	1,493,457	5,039	0.3%	9,396	0.6%
	**Asian**	607,284	1,370	0.2%	2,421	0.4%
	**Unknown**	1,719,014	7,343	0.4%	11,049	0.6%
**Sex**	**Female**	7,199,363	21,975	0.3%	38,875	0.5%
	**Male**	6,520,571	16,652	0.3%	28,274	0.4%
**Region**	**Northeast**	1,526,275	4,349	0.3%	7,660	0.5%
	**Midwest**	3,322,846	8,389	0.3%	13,719	0.4%
	**South**	5,619,435	19,018	0.3%	33,725	0.6%
	**West**	3,251,378	6,871	0.2%	12,045	0.4%
**Total**		13,719,934	38,627	0.3%	67,149	0.5%

Source: De-identified Optum Clinformatic ^®^ Data Mart.

Enrollees with full enrollment in a commercial health plan or Medicare during the year.

**Table 222:** Gastrointestinal Infections (exc. C. difficile): Ambulatory care visits with first-listed and all-listed diagnoses by age, race-ethnicity, sex and region among privately insured enrollees, 2020

Demographic Characteristics		First-Listed Diagnosis		All-Listed Diagnoses	
		Number of events	Event rate per 100,000	Number of events	Event rate per 100,000
**Age (years)**	**0 to 11**	7,126	647	9,592	870
	**12 to 24**	4,791	330	6,796	468
	**25 to 44**	8,008	301	12,919	486
	**45 to 54**	3,852	265	7,267	500
	**55 to 64**	4,017	245	8,065	492
	**65 to 74**	6,298	222	13,223	465
	**75 plus**	5,716	222	11,860	460
**Race-ethnicity**	**White**	21,410	247	37,151	429
	**Black**	3,880	312	7,281	586
	**Hispanic**	5,365	359	10,559	707
	**Asian**	1,466	241	2,765	455
	**Unknown**	7,687	447	11,966	696
**Sex**	**Female**	22,588	314	40,674	565
	**Male**	17,220	264	29,048	445
**Region**	**Northeast**	4,512	296	8,008	525
	**Midwest**	8,538	257	13,571	408
	**South**	19,559	348	34,967	622
	**West**	7,199	221	13,176	405
**Total**		39,808	290	69,722	508

Source: De-identified Optum Clinformatic ^®^ Data Mart.

Enrollees with full enrollment in a commercial health plan or Medicare during the year.

**Table 223:** Gastrointestinal Infections (exc. C. difficile): Emergency department visits with first-listed and all-listed diagnoses by age, race-ethnicity, sex and region among privately insured enrollees, 2020

Demographic Characteristics		First-Listed Diagnosis		All-Listed Diagnoses	
		Number of events	Event rate per 100,000	Number of events	Event rate per 100,000
**Age (years)**	**0 to 11**	23	2	33	3
	**12 to 24**	16	1	35	2
	**25 to 44**	117	4	205	8
	**45 to 54**	135	9	225	15
	**55 to 64**	265	16	458	28
	**65 to 74**	553	19	1,042	37
	**75 plus**	550	21	1,085	42
**Race-ethnicity**	**White**	1,027	12	1,888	22
	**Black**	243	20	445	36
	**Hispanic**	200	13	412	28
	**Asian**	31	5	55	9
	**Unknown**	158	9	283	16
**Sex**	**Female**	1,104	15	2,067	29
	**Male**	555	9	1,016	16
**Region**	**Northeast**	190	12	306	20
	**Midwest**	444	13	720	22
	**South**	804	14	1,593	28
	**West**	221	7	464	14
**Total**		1,659	12	3,083	22

Source: De-identified Optum Clinformatic ^®^ Data Mart.

Enrollees with full enrollment in a commercial health plan or Medicare during the year.

**Table 224:** Gastrointestinal Infections (exc. C. difficile): Hospital discharges with first-listed and all-listed diagnoses by age, race-ethnicity, sex and region among privately insured enrollees, 2020

Demographic Characteristics		First-Listed Diagnosis		All-Listed Diagnoses	
		Number of events	Event rate per 100,000	Number of events	Event rate per 100,000
**Age (years)**	**0 to 11**	105	10	222	20
	**12 to 24**	69	5	194	13
	**25 to 44**	151	6	550	21
	**45 to 54**	213	15	741	51
	**55 to 64**	366	22	1,511	92
	**65 to 74**	744	26	3,331	117
	**75 plus**	1,132	44	4,530	176
**Race-ethnicity**	**White**	1,839	21	7,104	82
	**Black**	347	28	1,535	124
	**Hispanic**	263	18	1,216	81
	**Asian**	51	8	231	38
	**Unknown**	280	16	993	58
**Sex**	**Female**	1,818	25	6,754	94
	**Male**	962	15	4,325	66
**Region**	**Northeast**	366	24	1,571	103
	**Midwest**	603	18	2,423	73
	**South**	1,352	24	5,246	93
	**West**	459	14	1,839	57
**Total**		2,780	20	11,079	81

Source: De-identified Optum Clinformatic ^®^ Data Mart.

Enrollees with full enrollment in a commercial health plan or Medicare during the year.

**Table 225:** Gastrointestinal Infections (exc. C. difficile): Claims-based prevalence with first-listed and all-listed diagnoses by age, race, sex and region among fee-for-service, age-eligible Medicare beneficiaries, 2019

Demographic Characteristics		Number of enrollees	First-Listed Diagnosis		All-Listed Diagnoses	
			Number of enrollees with disease	Percent of enrollees with disease	Number of enrollees with disease	Percent of enrollees with disease
**Age (years)**	**65 to 69**	8,066,320	30,860	0.4%	56,600	0.7%
	**70 to 74**	6,936,940	30,000	0.4%	54,300	0.8%
	**75 to 79**	4,894,060	22,540	0.5%	41,700	0.9%
	**80 to 84**	3,335,340	17,660	0.5%	30,960	0.9%
	**85+**	3,578,120	18,360	0.5%	33,000	0.9%
**Race**	**White**	22,023,800	96,880	0.4%	174,260	0.8%
	**Black**	1,861,660	7,740	0.4%	14,300	0.8%
	**Other**	2,925,320	14,800	0.5%	28,000	1.0%
**Sex**	**Female**	14,975,560	77,640	0.5%	139,240	0.9%
	**Male**	11,835,220	41,780	0.4%	77,320	0.7%
**Region**	**Northeast**	4,748,700	22,820	0.5%	41,000	0.9%
	**Midwest**	6,069,800	24,620	0.4%	43,700	0.7%
	**South**	10,595,900	50,380	0.5%	90,780	0.9%
	**West**	5,396,380	21,600	0.4%	41,080	0.8%
**Total**		26,810,780	119,420	0.4%	216,560	0.8%

Source: Centers for Medicare & Medicaid Services, 5% Denominator and Claims Files.

Beneficiaries aged 65+ years with continuous and full Parts A and B enrollment and no Health Maintenance Organization enrollment during the year.

Unweighted counts were multiplied by 20 to produce counts for this table.

**Table 226:** Gastrointestinal Infections (exc. C. difficile): Ambulatory care visits with first-listed and all-listed diagnoses by age, race, sex and region among fee-for-service, age-eligible Medicare beneficiaries, 2019

Demographic Characteristics		First-Listed Diagnosis		All-Listed Diagnoses	
		Number of events	Event rate per 100,000	Number of events	Event rate per 100,000
**Age (years)**	**65 to 69**	24,660	306	52,760	654
	**70 to 74**	23,740	342	49,320	711
	**75 to 79**	18,760	383	38,600	789
	**80 to 84**	13,260	398	26,480	794
	**85+**	14,620	409	28,760	804
**Race**	**White**	76,520	347	154,820	703
	**Black**	5,620	302	12,840	690
	**Other**	12,900	441	28,260	966
**Sex**	**Female**	61,700	412	126,620	846
	**Male**	33,340	282	69,300	586
**Region**	**Northeast**	18,980	400	37,820	796
	**Midwest**	17,460	288	34,960	576
	**South**	40,720	384	84,140	794
	**West**	17,880	331	39,000	723
**Total**		95,040	354	195,920	731

Source: Centers for Medicare & Medicaid Services, 5% Denominator and Claims Files.

Beneficiaries aged 65+ years with continuous and full Parts A and B enrollment and no Health Maintenance Organization enrollment during the year.

Unweighted counts were multiplied by 20 to produce counts for this table.

**Table 227:** Gastrointestinal Infections (exc. C. difficile): Emergency department visits with first-listed and all-listed diagnoses by age, race, sex and region among fee-for-service, age-eligible Medicare beneficiaries, 2019

Demographic Characteristics		First-Listed Diagnosis		All-Listed Diagnoses	
		Number of events	Event rate per 100,000	Number of events	Event rate per 100,000
**Age (years)**	**65 to 69**	9,920	123	17,340	215
	**70 to 74**	10,220	147	17,520	253
	**75 to 79**	7,380	151	13,760	281
	**80 to 84**	7,100	213	12,960	389
	**85+**	8,380	234	16,180	452
**Race**	**White**	35,460	161	63,880	290
	**Black**	3,060	164	5,680	305
	**Other**	4,480	153	8,200	280
**Sex**	**Female**	28,900	193	49,900	333
	**Male**	14,100	119	27,860	235
**Region**	**Northeast**	8,280	174	15,520	327
	**Midwest**	10,420	172	18,340	302
	**South**	17,240	163	30,540	288
	**West**	7,060	131	13,360	248
**Total**		43,000	160	77,760	290

Source: Centers for Medicare & Medicaid Services, 5% Denominator and Claims Files.

Beneficiaries aged 65+ years with continuous and full Parts A and B enrollment and no Health Maintenance Organization enrollment during the year.

Unweighted counts were multiplied by 20 to produce counts for this table.

**Table 228:** Gastrointestinal Infections (exc. C. difficile): Hospital discharges with first-listed and all-listed diagnoses by age, race, sex and region among fee-for-service, age-eligible Medicare beneficiaries, 2019

Demographic Characteristics		First-Listed Diagnosis		All-Listed Diagnoses	
		Number of events	Event rate per 100,000	Number of events	Event rate per 100,000
**Age (years)**	**65 to 69**	4,660	58	11,600	144
	**70 to 74**	5,380	78	12,060	174
	**75 to 79**	3,920	80	10,040	205
	**80 to 84**	4,080	122	9,520	285
	**85+**	4,880	136	12,040	336
**Race**	**White**	18,920	86	45,520	207
	**Black**	1,680	90	3,940	212
	**Other**	2,320	79	5,800	198
**Sex**	**Female**	15,200	101	34,760	232
	**Male**	7,720	65	20,500	173
**Region**	**Northeast**	5,020	106	12,000	253
	**Midwest**	5,500	91	13,160	217
	**South**	8,720	82	20,580	194
	**West**	3,680	68	9,520	176
**Total**		22,920	85	55,260	206

Source: Centers for Medicare & Medicaid Services, 5% Denominator and Claims Files.

Beneficiaries aged 65+ years with continuous and full Parts A and B enrollment and no Health Maintenance Organization enrollment during the year.

Unweighted counts were multiplied by 20 to produce counts for this table.

**Table 229:** Clostridium difficile: Ambulatory care visits with first-listed and all-listed diagnoses by age, race, ethnicity, and sex in the United States, 2015

Demographic Characteristics		First-Listed Diagnosis		All-Listed Diagnoses	
		Number in thousands	Rate per 100,000	Number in thousands	Rate per 100,000
**Age (years)**	**0 to 11**	16	34	18	38
	**12 to 24**	3	5	3	5
	**25 to 44**	15	17	20	24
	**45 to 54**	21	48	27	62
	**55 to 64**	49	119	56	136
	**65 to 74**	28	100	67	245
	**75 plus**	39	195	59	291
**Race**	**White**	140	47	211	71
	**Black**	7	18	14	42
	**Other**	23	80	24	83
**Ethnicity**	**Hispanic**	23	49	26	56
	**Not Hispanic**	147	48	223	70
**Sex**	**Female**	104	50	166	80
	**Male**	66	39	83	50
**Total**		170	45	249	67

Source: National Ambulatory Medical Care Survey (3-year average, 2014–2016).

Rates for age groups are unadjusted and all other rates are age-adjusted.

**Table 230:** Clostridium difficile: Emergency department visits with first-listed and all-listed diagnoses by age and sex in the United States, 2018

Demographic Characteristics		First-Listed Diagnosis		All-Listed Diagnoses	
		Number in thousands	Rate per 100,000	Number in thousands	Rate per 100,000
**Age (years)**	**0 to 11**	3	6	5	11
	**12 to 24**	4	8	9	16
	**25 to 44**	14	16	32	37
	**45 to 54**	12	29	31	75
	**55 to 64**	18	43	52	123
	**65 to 74**	21	68	63	208
	**75 plus**	36	162	100	454
**Sex**	**Female**	70	35	173	85
	**Male**	39	23	120	69
**Total**		108	29	293	78

Source: Healthcare Cost and Utilization Project Nationwide Emergency Department Sample.

Rates for age groups are unadjusted and all other rates are age-adjusted.

**Table 231:** Clostridium difficile: Hospital discharges with first-listed and all-listed diagnoses by age, race, ethnicity, and sex in the United States, 2018

Demographic Characteristics		First-Listed Diagnosis		All-Listed Diagnoses	
		Number in thousands	Rate per 100,000	Number in thousands	Rate per 100,000
**Age (years)**	**0 to 11**	2	4	5	10
	**12 to 24**	3	5	8	15
	**25 to 44**	9	10	29	33
	**45 to 54**	9	21	30	73
	**55 to 64**	15	35	57	135
	**65 to 74**	19	63	71	233
	**75 plus**	33	149	106	485
**Race**	**White**	77	25	254	81
	**Black**	10	23	40	93
	**Other**	3	15	15	63
**Ethnicity**	**Hispanic**	7	18	27	63
	**Not Hispanic**	82	25	282	84
**Sex**	**Female**	57	28	175	85
	**Male**	32	19	132	75
**Total**		89	23	307	80

Source: Healthcare Cost and Utilization Project National Inpatient Sample.

Rates for age groups are unadjusted and all other rates are age-adjusted.

**Table 232:** Clostridium difficile: Deaths with underlying or underlying/other cause and lifetime years of life lost by age, race, ethnicity, and sex in the United States, 2019

Demographic Characteristics		Underlying Cause			Underlying/Other Cause		
		Number of Deaths	Rate per 100,000	Years of potential life lost in thousands	Number of Deaths	Rate per 100,000	Years of potential life lost in thousands
**Age (years)**	**0 to 11**	5	0.0	0.4	9	0.0	0.7
	**12 to 24**	2	0.0	0.1	13	0.0	0.7
	**25 to 44**	66	0.1	2.8	144	0.2	6.1
	**45 to 54**	135	0.3	4.2	287	0.7	8.9
	**55 to 64**	444	1.0	10.2	916	2.2	21.0
	**65 to 74**	949	3.0	15.0	1,814	5.8	28.4
	**75 plus**	2,940	13.0	20.0	4,956	22.0	34.6
**Race**	**White**	3,956	1.2	44.2	7,095	2.1	84.6
	**Black**	441	1.1	6.7	803	1.9	12.6
	**Other**	144	0.6	1.8	241	1.0	3.3
**Ethnicity**	**Hispanic**	308	0.9	4.5	509	1.4	8.0
	**Not Hispanic**	4,233	1.1	48.1	7,630	2.1	92.4
**Sex**	**Female**	2,644	1.1	30.8	4,571	2.0	56.7
	**Male**	1,897	1.1	21.8	3,568	2.1	43.8
**Total**		4,541	1.1	52.6	8,139	2.0	100.5

Source: Vital Statistics of the United States.

Rates for age groups are unadjusted and all other rates are age-adjusted.

Years of potential life lost is based on U.S. life Table 2006–2018 estimates of life expectancy at age of death.

**Table 233:** Clostridium difficile: Claims-based prevalence with first-listed and all-listed diagnoses by age, race-ethnicity, sex and region among privately insured enrollees, 2020

Demographic Characteristics		Number of enrollees	First-Listed Diagnosis		All-Listed Diagnoses	
			Number of enrollees with disease	Percent of enrollees with disease	Number of enrollees with disease	Percent of enrollees with disease
**Age (years)**	**0 to 11**	1,101,978	56	0.0%	121	0.0%
	**12 to 24**	1,451,892	161	0.0%	308	0.0%
	**25 to 44**	2,656,672	507	0.0%	965	0.0%
	**45 to 54**	1,452,320	526	0.0%	1,083	0.1%
	**55 to 64**	1,640,216	1,128	0.1%	2,503	0.2%
	**65 to 74**	2,840,615	2,483	0.1%	5,627	0.2%
	**75 plus**	2,576,241	4,530	0.2%	9,292	0.4%
**Race-ethnicity**	**White**	8,657,702	6,991	0.1%	14,293	0.2%
	**Black**	1,242,477	866	0.1%	2,182	0.2%
	**Hispanic**	1,493,457	728	0.0%	1,656	0.1%
	**Asian**	607,284	147	0.0%	353	0.1%
	**Unknown**	1,719,014	659	0.0%	1,415	0.1%
**Sex**	**Female**	7,199,363	6,141	0.1%	12,325	0.2%
	**Male**	6,520,571	3,250	0.0%	7,574	0.1%
**Region**	**Northeast**	1,526,275	1,251	0.1%	2,631	0.2%
	**Midwest**	3,322,846	2,262	0.1%	4,806	0.1%
	**South**	5,619,435	3,892	0.1%	8,361	0.1%
	**West**	3,251,378	1,986	0.1%	4,101	0.1%
**Total**		13,719,934	9,391	0.1%	19,899	0.1%

Source: De-identified Optum Clinformatic ^®^ Data Mart.

Enrollees with full enrollment in a commercial health plan or Medicare during the year.

**Table 234:** Clostridium difficile: Ambulatory care visits with first-listed and all-listed diagnoses by age, race-ethnicity, sex and region among privately insured enrollees, 2020

Demographic Characteristics		First-Listed Diagnosis		All-Listed Diagnoses	
		Number of events	Event rate per 100,000	Number of events	Event rate per 100,000
**Age (years)**	**0 to 11**	63	6	147	13
	**12 to 24**	222	15	454	31
	**25 to 44**	744	28	1,533	58
	**45 to 54**	765	53	1,670	115
	**55 to 64**	1,771	108	4,123	251
	**65 to 74**	4,218	148	10,143	357
	**75 plus**	9,474	368	21,614	839
**Race-ethnicity**	**White**	13,316	154	29,790	344
	**Black**	1,376	111	3,631	292
	**Hispanic**	1,206	81	3,107	208
	**Asian**	221	36	573	94
	**Unknown**	1,138	66	2,583	150
**Sex**	**Female**	11,239	156	25,525	355
	**Male**	6,018	92	14,159	217
**Region**	**Northeast**	2,588	170	5,918	388
	**Midwest**	3,967	119	9,593	289
	**South**	6,684	119	14,887	265
	**West**	4,018	124	9,286	286
**Total**		17,257	126	39,684	289

Source: De-identified Optum Clinformatic ^®^ Data Mart.

Enrollees with full enrollment in a commercial health plan or Medicare during the year.

**Table 235:** Clostridium difficile: Emergency department visits with first-listed and all-listed diagnoses by age, race-ethnicity, sex and region among privately insured enrollees, 2020

Demographic Characteristics		First-Listed Diagnosis		All-Listed Diagnoses	
		Number of events	Event rate per 100,000	Number of events	Event rate per 100,000
**Age (years)**	**0 to 11**	1	0	1	0
	**12 to 24**	4	0	6	0
	**25 to 44**	24	1	39	1
	**45 to 54**	28	2	70	5
	**55 to 64**	105	6	226	14
	**65 to 74**	290	10	612	22
	**75 plus**	527	20	1,117	43
**Race-ethnicity**	**White**	763	9	1,586	18
	**Black**	73	6	174	14
	**Hispanic**	69	5	149	10
	**Asian**	13	2	24	4
	**Unknown**	61	4	138	8
**Sex**	**Female**	640	9	1,324	18
	**Male**	339	5	747	11
**Region**	**Northeast**	151	10	290	19
	**Midwest**	298	9	594	18
	**South**	334	6	752	13
	**West**	196	6	435	13
**Total**		979	7	2,071	15

Source: De-identified Optum Clinformatic ^®^ Data Mart.

Enrollees with full enrollment in a commercial health plan or Medicare during the year.

**Table 236:** Clostridium difficile: Hospital discharges with first-listed and all-listed diagnoses by age, race-ethnicity, sex and region among privately insured enrollees, 2020

Demographic Characteristics		First-Listed Diagnosis		All-Listed Diagnoses	
		Number of events	Event rate per 100,000	Number of events	Event rate per 100,000
**Age (years)**	**0 to 11**	14	1	65	6
	**12 to 24**	33	2	133	9
	**25 to 44**	90	3	393	15
	**45 to 54**	156	11	662	46
	**55 to 64**	371	23	1,904	116
	**65 to 74**	841	30	4,305	152
	**75 plus**	1,595	62	6,788	263
**Race-ethnicity**	**White**	2,240	26	9,845	114
	**Black**	336	27	1,869	150
	**Hispanic**	269	18	1,297	87
	**Asian**	37	6	227	37
	**Unknown**	218	13	1,012	59
**Sex**	**Female**	2,053	29	8,320	116
	**Male**	1,047	16	5,930	91
**Region**	**Northeast**	403	26	1,895	124
	**Midwest**	683	21	3,449	104
	**South**	1,446	26	6,209	110
	**West**	568	17	2,697	83
**Total**		3,100	23	14,250	104

Source: De-identified Optum Clinformatic ^®^ Data Mart.

Enrollees with full enrollment in a commercial health plan or Medicare during the year.

**Table 237:** Clostridium difficile: Claims-based prevalence with first-listed and all-listed diagnoses by age, race, sex and region among fee-for-service, age-eligible Medicare beneficiaries, 2019

Demographic Characteristics		Number of enrollees	First-Listed Diagnosis		All-Listed Diagnoses	
			Number of enrollees with disease	Percent of enrollees with disease	Number of enrollees with disease	Percent of enrollees with disease
**Age (years)**	**65 to 69**	8,066,320	10,820	0.1%	23,040	0.3%
	**70 to 74**	6,936,940	13,600	0.2%	26,500	0.4%
	**75 to 79**	4,894,060	14,440	0.3%	25,760	0.5%
	**80 to 84**	3,335,340	10,100	0.3%	20,060	0.6%
	**85+**	3,578,120	16,520	0.5%	29,880	0.8%
**Race**	**White**	22,023,800	57,120	0.3%	106,640	0.5%
	**Black**	1,861,660	4,020	0.2%	8,940	0.5%
	**Other**	2,925,320	4,340	0.1%	9,660	0.3%
**Sex**	**Female**	14,975,560	42,500	0.3%	78,120	0.5%
	**Male**	11,835,220	22,980	0.2%	47,120	0.4%
**Region**	**Northeast**	4,748,700	12,040	0.3%	23,480	0.5%
	**Midwest**	6,069,800	16,480	0.3%	30,080	0.5%
	**South**	10,595,900	26,420	0.2%	50,320	0.5%
	**West**	5,396,380	10,540	0.2%	21,360	0.4%
**Total**		26,810,780	65,480	0.2%	125,240	0.5%

Source: Centers for Medicare & Medicaid Services, 5% Denominator and Claims Files.

Beneficiaries aged 65+ years with continuous and full Parts A and B enrollment and no Health Maintenance Organization enrollment during the year.

Unweighted counts were multiplied by 20 to produce counts for this table.

**Table 238:** Clostridium difficile: Ambulatory care visits with first-listed and all-listed diagnoses by age, race, sex and region among fee-for-service, age-eligible Medicare beneficiaries, 2019

Demographic Characteristics		First-Listed Diagnosis		All-Listed Diagnoses	
		Number of events	Event rate per 100,000	Number of events	Event rate per 100,000
**Age (years)**	**65 to 69**	16,700	207	41,420	513
	**70 to 74**	19,480	281	49,680	716
	**75 to 79**	23,980	490	52,540	1,074
	**80 to 84**	18,820	564	42,920	1,287
	**85+**	31,020	867	70,900	1,981
**Race**	**White**	96,780	439	225,020	1,022
	**Black**	6,340	341	15,880	853
	**Other**	6,880	235	16,560	566
**Sex**	**Female**	74,480	497	169,100	1,129
	**Male**	35,520	300	88,360	747
**Region**	**Northeast**	22,440	473	55,240	1,163
	**Midwest**	25,820	425	59,400	979
	**South**	43,440	410	98,420	929
	**West**	18,300	339	44,400	823
**Total**		110,000	410	257,460	960

Source: Centers for Medicare & Medicaid Services, 5% Denominator and Claims Files.

Beneficiaries aged 65+ years with continuous and full Parts A and B enrollment and no Health Maintenance Organization enrollment during the year.

Unweighted counts were multiplied by 20 to produce counts for this table.

**Table 239:** Clostridium difficile: Emergency department visits with first-listed and all-listed diagnoses by age, race, sex and region among fee-for-service, age-eligible Medicare beneficiaries, 2019

Demographic Characteristics		First-Listed Diagnosis		All-Listed Diagnoses	
		Number of events	Event rate per 100,000	Number of events	Event rate per 100,000
**Age (years)**	**65 to 69**	5,200	64	15,920	197
	**70 to 74**	6,680	96	18,140	261
	**75 to 79**	7,000	143	18,040	369
	**80 to 84**	5,140	154	14,560	437
	**85+**	8,800	246	22,140	619
**Race**	**White**	28,300	128	73,460	334
	**Black**	2,180	117	7,980	429
	**Other**	2,340	80	7,360	252
**Sex**	**Female**	21,560	144	54,480	364
	**Male**	11,260	95	34,320	290
**Region**	**Northeast**	6,260	132	17,020	358
	**Midwest**	8,080	133	21,580	356
	**South**	13,400	126	34,560	326
	**West**	5,080	94	15,640	290
**Total**		32,820	122	88,800	331

Source: Centers for Medicare & Medicaid Services, 5% Denominator and Claims Files.

Beneficiaries aged 65+ years with continuous and full Parts A and B enrollment and no Health Maintenance Organization enrollment during the year.

Unweighted counts were multiplied by 20 to produce counts for this table.

**Table 240:** Clostridium difficile: Hospital discharges with first-listed and all-listed diagnoses by age, race, sex and region among fee-for-service, age-eligible Medicare beneficiaries, 2019

Demographic Characteristics		First-Listed Diagnosis		All-Listed Diagnoses	
		Number of events	Event rate per 100,000	Number of events	Event rate per 100,000
**Age (years)**	**65 to 69**	4,420	55	18,420	228
	**70 to 74**	5,400	78	19,940	287
	**75 to 79**	5,900	121	19,660	402
	**80 to 84**	4,240	127	15,420	462
	**85+**	7,740	216	22,320	624
**Race**	**White**	24,140	110	78,640	357
	**Black**	1,860	100	8,900	478
	**Other**	1,700	58	8,220	281
**Sex**	**Female**	18,220	122	57,100	381
	**Male**	9,480	80	38,660	327
**Region**	**Northeast**	4,880	103	17,520	369
	**Midwest**	7,220	119	23,820	392
	**South**	11,840	112	38,560	364
	**West**	3,760	70	15,860	294
**Total**		27,700	103	95,760	357

Source: Centers for Medicare & Medicaid Services, 5% Denominator and Claims Files.

Beneficiaries aged 65+ years with continuous and full Parts A and B enrollment and no Health Maintenance Organization enrollment during the year.

Unweighted counts were multiplied by 20 to produce counts for this table.

**Table 241:** Esophageal Cancer: Incidence rates by age, race, ethnicity, and sex, 2016

Demographic Characteristics		Number of cases	Incidence per 100,000
**Age (years)**	**0 to 11**	.	.
	**12 to 24**	.	.
	**25 to 44**	324	0.4
	**45 to 54**	1,347	3.2
	**55 to 64**	4,323	10.2
	**65 to 74**	5,692	18.7
	**75 plus**	5,350	24.4
**Race**	**White**	14,984	4.6
	**Black**	1,395	3.3
	**Other**	686	2.5
**Ethnicity**	**Hispanic**	842	2.0
	**Not Hispanic**	15,065	4.3
**Sex**	**Female**	3,791	1.8
	**Male**	12,562	7.0
**Total**		16,376	4.2

Source: Surveillance, Epidemiology, and End Results (SEER) Program.

Rates for age groups are unadjusted and all other rates are age-adjusted.

**Table 242:** Esophageal Cancer: Hospital discharges with first-listed and all-listed diagnoses by age, race, ethnicity, and sex in the United States, 2018

Demographic Characteristics		First-Listed Diagnosis		All-Listed Diagnoses	
		Number in thousands	Rate per 100,000	Number in thousands	Rate per 100,000
**Age (years)**	**0 to 11**	.	.	.	.
	**12 to 24**	.	.	0	0
	**25 to 44**	0	0	1	1
	**45 to 54**	2	4	4	10
	**55 to 64**	4	10	11	27
	**65 to 74**	4	14	13	43
	**75 plus**	3	15	10	48
**Race**	**White**	12	4	34	10
	**Black**	1	3	4	8
	**Other**	1	3	2	8
**Ethnicity**	**Hispanic**	1	2	3	6
	**Not Hispanic**	13	4	38	10
**Sex**	**Female**	3	1	8	4
	**Male**	11	6	32	17
**Total**		14	3	40	10

Source: Healthcare Cost and Utilization Project National Inpatient Sample.

Rates for age groups are unadjusted and all other rates are age-adjusted.

**Table 243:** Esophageal Cancer: Deaths with underlying or underlying/other cause and lifetime years of life lost by age, race, ethnicity, and sex in the United States, 2019

Demographic Characteristics		Underlying Cause			Underlying/Other Cause		
		Number of Deaths	Rate per 100,000	Years of potential life lost in thousands	Number of Deaths	Rate per 100,000	Years of potential life lost in thousands
**Age (years)**	**0 to 11**	.	.	.	.	.	.
	**12 to 24**	6	0.0	0.3	6	0.0	0.3
	**25 to 44**	283	0.3	11.4	298	0.3	12.0
	**45 to 54**	1,122	2.7	33.1	1,197	2.9	35.3
	**55 to 64**	3,817	9.0	83.9	4,081	9.6	89.7
	**65 to 74**	5,200	16.5	79.4	5,702	18.1	87.0
	**75 plus**	5,557	24.6	43.8	6,221	27.6	49.0
**Race**	**White**	14,295	4.1	223.7	15,631	4.5	242.3
	**Black**	1,264	2.8	21.2	1,410	3.1	23.4
	**Other**	426	1.7	7.1	464	1.8	7.7
**Ethnicity**	**Hispanic**	681	1.7	12.9	753	1.8	14.2
	**Not Hispanic**	15,304	4.1	239.1	16,752	4.5	259.2
**Sex**	**Female**	3,145	1.4	50.8	3,466	1.5	55.5
	**Male**	12,840	6.8	201.2	14,039	7.4	217.9
**Total**		15,985	3.8	252.0	17,505	4.2	273.4

Source: Vital Statistics of the United States.

Rates for age groups are unadjusted and all other rates are age-adjusted.

Years of potential life lost is based on U.S. life Table 2006–2018 estimates of life expectancy at age of death.

**Table 244:** Gastric Cancer: Incidence rates by age, race, ethnicity, and sex, 2016

Demographic Characteristics		Number of cases	Incidence per 100,000
**Age (years)**	**0 to 11**	.	.
	**12 to 24**	.	0.2
	**25 to 44**	1,346	1.5
	**45 to 54**	2,262	5.4
	**55 to 64**	5,412	12.8
	**65 to 74**	7,184	23.6
	**75 plus**	8,093	36.9
**Race**	**White**	17,077	5.4
	**Black**	3,483	8.5
	**Other**	2,422	9.1
**Ethnicity**	**Hispanic**	3,486	8.6
	**Not Hispanic**	19,874	5.9
**Sex**	**Female**	9,182	4.5
	**Male**	14,343	8.2
**Total**		23,539	6.2

Source: Surveillance, Epidemiology, and End Results (SEER) Program.

Rates for age groups are unadjusted and all other rates are age-adjusted.

**Table 245:** Gastric Cancer: Hospital discharges with first-listed and all-listed diagnoses by age, race, ethnicity, and sex in the United States, 2018

Demographic Characteristics		First-Listed Diagnosis		All-Listed Diagnoses	
		Number in thousands	Rate per 100,000	Number in thousands	Rate per 100,000
**Age (years)**	**0 to 11**	.	.	0	0
	**12 to 24**	0	0	0	0
	**25 to 44**	1	2	3	4
	**45 to 54**	2	5	5	12
	**55 to 64**	5	11	10	24
	**65 to 74**	5	16	11	37
	**75 plus**	5	24	12	53
**Race**	**White**	13	4	29	9
	**Black**	3	7	7	16
	**Other**	3	11	5	22
**Ethnicity**	**Hispanic**	4	8	7	17
	**Not Hispanic**	15	4	34	10
**Sex**	**Female**	7	3	15	7
	**Male**	12	7	26	14
**Total**		19	5	41	11

Source: Healthcare Cost and Utilization Project National Inpatient Sample.

Rates for age groups are unadjusted and all other rates are age-adjusted.

**Table 246:** Gastric Cancer: Deaths with underlying or underlying/other cause and lifetime years of life lost by age, race, ethnicity, and sex in the United States, 2019

Demographic Characteristics		Underlying Cause			Underlying/Other Cause		
		Number of Deaths	Rate per 100,000	Years of potential life lost in thousands	Number of Deaths	Rate per 100,000	Years of potential life lost in thousands
**Age (years)**	**0 to 11**	.	.	.	.	.	.
	**12 to 24**	21	0.0	1.2	23	0.0	1.3
	**25 to 44**	559	0.6	24.0	583	0.7	25.1
	**45 to 54**	1,021	2.5	32.1	1,055	2.6	33.2
	**55 to 64**	2,074	4.9	47.2	2,208	5.2	50.2
	**65 to 74**	2,925	9.3	45.7	3,149	10.0	49.1
	**75 plus**	4,513	20.0	34.3	5,039	22.3	38.2
**Race**	**White**	8,137	2.5	136.3	8,829	2.7	145.4
	**Black**	1,941	4.6	30.5	2,114	5.0	32.9
	**Other**	1,035	4.1	17.9	1,114	4.5	18.8
**Ethnicity**	**Hispanic**	1,709	4.1	38.4	1,828	4.4	40.5
	**Not Hispanic**	9,404	2.6	146.3	10,229	2.8	156.7
**Sex**	**Female**	4,478	2.1	79.1	4,827	2.2	84.1
	**Male**	6,635	3.7	105.6	7,230	4.0	113.1
**Total**		11,113	2.8	184.7	12,057	3.0	197.2

Source: Vital Statistics of the United States.

Rates for age groups are unadjusted and all other rates are age-adjusted.

Years of potential life lost is based on U.S. life Table 2006–2018 estimates of life expectancy at age of death.

**Table 247:** Cancer of Small Intestine: Incidence rates by age, race, ethnicity, and sex, 2016

Demographic Characteristics		Number of cases	Incidence per 100,000
**Age (years)**	**0 to 11**	.	.
	**12 to 24**	.	0.0
	**25 to 44**	577	0.7
	**45 to 54**	1,168	2.8
	**55 to 64**	2,081	4.9
	**65 to 74**	2,873	9.4
	**75 plus**	2,630	12.0
**Race**	**White**	7,256	2.3
	**Black**	1,607	3.8
	**Other**	455	1.7
**Ethnicity**	**Hispanic**	674	1.6
	**Not Hispanic**	8,157	2.5
**Sex**	**Female**	4,247	2.1
	**Male**	4,795	2.7
**Total**		9,044	2.4

Source: Surveillance, Epidemiology, and End Results (SEER) Program.

Rates for age groups are unadjusted and all other rates are age-adjusted.

**Table 248:** Cancer of Small Intestine: Hospital discharges with first-listed and all-listed diagnoses by age, race, ethnicity, and sex in the United States, 2018

Demographic Characteristics		First-Listed Diagnosis		All-Listed Diagnoses	
		Number in thousands	Rate per 100,000	Number in thousands	Rate per 100,000
**Age (years)**	**0 to 11**	.	.	.	.
	**12 to 24**	0	0	0	0
	**25 to 44**	0	0	0	1
	**45 to 54**	0	1	1	3
	**55 to 64**	1	2	2	5
	**65 to 74**	1	4	3	9
	**75 plus**	1	6	3	13
**Race**	**White**	3	1	7	2
	**Black**	1	2	2	4
	**Other**	0	1	1	3
**Ethnicity**	**Hispanic**	0	1	1	2
	**Not Hispanic**	4	1	9	2
**Sex**	**Female**	2	1	4	2
	**Male**	2	1	5	3
**Total**		4	1	9	2

Source: Healthcare Cost and Utilization Project National Inpatient Sample.

Rates for age groups are unadjusted and all other rates are age-adjusted.

**Table 249:** Cancer of Small Intestine: Deaths with underlying or underlying/other cause and lifetime years of life lost by age, race, ethnicity, and sex in the United States, 2019

Demographic Characteristics		Underlying Cause			Underlying/Other Cause		
		Number of Deaths	Rate per 100,000	Years of potential life lost in thousands	Number of Deaths	Rate per 100,000	Years of potential life lost in thousands
**Age (years)**	**0 to 11**	.	.	.	.	.	.
	**12 to 24**	3	0.0	0.2	3	0.0	0.2
	**25 to 44**	51	0.1	2.1	54	0.1	2.3
	**45 to 54**	107	0.3	3.3	119	0.3	3.6
	**55 to 64**	286	0.7	6.5	315	0.7	7.1
	**65 to 74**	490	1.6	7.7	542	1.7	8.5
	**75 plus**	771	3.4	5.9	883	3.9	6.8
**Race**	**White**	1,362	0.4	20.1	1,528	0.4	22.3
	**Black**	277	0.6	4.6	308	0.7	5.2
	**Other**	69	0.3	1.0	80	0.3	1.1
**Ethnicity**	**Hispanic**	100	0.3	1.8	120	0.3	2.2
	**Not Hispanic**	1,608	0.4	23.9	1,796	0.5	26.3
**Sex**	**Female**	782	0.3	12.4	867	0.4	13.6
	**Male**	926	0.5	13.3	1,049	0.6	14.9
**Total**		1,708	0.4	25.7	1,916	0.5	28.5

Source: Vital Statistics of the United States.

Rates for age groups are unadjusted and all other rates are age-adjusted.

Years of potential life lost is based on U.S. life Table 2006–2018 estimates of life expectancy at age of death.

**Table 250:** Colorectal Cancer: Incidence rates by age, race, ethnicity, and sex, 2016

Demographic Characteristics		Number of cases	Incidence per 100,000
**Age (years)**	**0 to 11**	.	0.0
	**12 to 24**	877	1.6
	**25 to 44**	8,814	10.1
	**45 to 54**	19,593	47.1
	**55 to 64**	29,710	70.3
	**65 to 74**	33,730	110.6
	**75 plus**	43,342	197.6
**Race**	**White**	106,708	34.3
	**Black**	16,878	40.6
	**Other**	9,409	34.7
**Ethnicity**	**Hispanic**	13,543	33.0
	**Not Hispanic**	115,855	35.3
**Sex**	**Female**	63,289	31.2
	**Male**	68,385	39.4
**Total**		131,692	35.0

Source: Surveillance, Epidemiology, and End Results (SEER) Program.

Rates for age groups are unadjusted and all other rates are age-adjusted.

**Table 251:** Colorectal Cancer: Hospital discharges with first-listed and all-listed diagnoses by age, race, ethnicity, and sex in the United States, 2018

Demographic Characteristics		First-Listed Diagnosis		All-Listed Diagnoses	
		Number in thousands	Rate per 100,000	Number in thousands	Rate per 100,000
**Age (years)**	**0 to 11**	0	0	0	0
	**12 to 24**	0	0	0	1
	**25 to 44**	7	8	16	18
	**45 to 54**	18	44	36	87
	**55 to 64**	29	68	59	139
	**65 to 74**	33	108	66	215
	**75 plus**	38	175	73	333
**Race**	**White**	103	32	202	63
	**Black**	15	34	32	74
	**Other**	9	36	18	71
**Ethnicity**	**Hispanic**	12	29	25	59
	**Not Hispanic**	114	33	227	66
**Sex**	**Female**	59	28	117	56
	**Male**	66	37	133	74
**Total**		126	32	250	64

Source: Healthcare Cost and Utilization Project National Inpatient Sample.

Rates for age groups are unadjusted and all other rates are age-adjusted.

**Table 252:** Colorectal Cancer: Deaths with underlying or underlying/other cause and lifetime years of life lost by age, race, ethnicity, and sex in the United States, 2019

Demographic Characteristics		Underlying Cause			Underlying/Other Cause		
		Number of Deaths	Rate per 100,000	Years of potential life lost in thousands	Number of Deaths	Rate per 100,000	Years of potential life lost in thousands
**Age (years)**	**0 to 11**	.	.	.	.	.	.
	**12 to 24**	38	0.1	2.2	42	0.1	2.4
	**25 to 44**	1,708	1.9	71.0	1,772	2.0	73.7
	**45 to 54**	4,734	11.6	146.4	4,988	12.2	154.1
	**55 to 64**	10,048	23.7	229.2	10,845	25.5	247.1
	**65 to 74**	12,684	40.3	199.8	14,354	45.6	225.4
	**75 plus**	22,494	99.6	164.0	27,798	123.1	199.0
**Race**	**White**	42,411	12.6	654.6	49,354	14.6	728.6
	**Black**	7,024	16.3	117.8	7,907	18.5	129.6
	**Other**	2,271	9.0	40.1	2,538	10.1	43.4
**Ethnicity**	**Hispanic**	3,654	8.8	74.4	4,063	9.9	80.6
	**Not Hispanic**	48,052	13.3	738.1	55,736	15.3	821.1
**Sex**	**Female**	24,124	10.7	377.1	27,678	12.2	415.7
	**Male**	27,582	15.2	435.4	32,121	17.8	486.0
**Total**		51,706	12.8	812.5	59,799	14.7	901.6

Source: Vital Statistics of the United States.

Rates for age groups are unadjusted and all other rates are age-adjusted.

Years of potential life lost is based on U.S. life Table 2006–2018 estimates of life expectancy at age of death.

**Table 253:** Primary Liver Cancer: Incidence rates by age, race, ethnicity, and sex, 2016

Demographic Characteristics		Number of cases	Incidence per 100,000
**Age (years)**	**0 to 11**	179	0.4
	**12 to 24**	.	0.2
	**25 to 44**	385	0.4
	**45 to 54**	1,852	4.4
	**55 to 64**	8,495	20.1
	**65 to 74**	9,980	32.7
	**75 plus**	6,524	29.7
**Race**	**White**	18,768	5.5
	**Black**	4,365	9.5
	**Other**	2,726	9.9
**Ethnicity**	**Hispanic**	4,316	10.8
	**Not Hispanic**	22,065	6.1
**Sex**	**Female**	6,640	3.1
	**Male**	19,823	10.4
**Total**		26,497	6.5

Source: Surveillance, Epidemiology, and End Results (SEER) Program.

Rates for age groups are unadjusted and all other rates are age-adjusted.

**Table 254:** Primary Liver Cancer: Hospital discharges with first-listed and all-listed diagnoses by age, race, ethnicity, and sex in the United States, 2018

Demographic Characteristics		First-Listed Diagnosis		All-Listed Diagnoses	
		Number in thousands	Rate per 100,000	Number in thousands	Rate per 100,000
**Age (years)**	**0 to 11**	0	1	2	4
	**12 to 24**	0	0	0	1
	**25 to 44**	0	0	2	2
	**45 to 54**	1	3	6	14
	**55 to 64**	6	13	23	54
	**65 to 74**	5	16	21	67
	**75 plus**	3	14	13	58
**Race**	**White**	11	3	49	14
	**Black**	2	5	10	20
	**Other**	2	9	7	28
**Ethnicity**	**Hispanic**	3	6	11	26
	**Not Hispanic**	13	4	55	15
**Sex**	**Female**	4	2	18	8
	**Male**	11	6	47	24
**Total**		16	4	65	16

Source: Healthcare Cost and Utilization Project National Inpatient Sample.

Rates for age groups are unadjusted and all other rates are age-adjusted.

**Table 255:** Primary Liver Cancer: Deaths with underlying or underlying/other cause and lifetime years of life lost by age, race, ethnicity, and sex in the United States, 2019

Demographic Characteristics		Underlying Cause			Underlying/Other Cause		
		Number of Deaths	Rate per 100,000	Years of potential life lost in thousands	Number of Deaths	Rate per 100,000	Years of potential life lost in thousands
**Age (years)**	**0 to 11**	28	0.1	2.1	32	0.1	2.4
	**12 to 24**	29	0.1	1.7	30	0.1	1.8
	**25 to 44**	233	0.3	9.6	260	0.3	10.7
	**45 to 54**	963	2.4	28.4	1,110	2.7	32.8
	**55 to 64**	5,547	13.1	121.0	6,235	14.7	136.2
	**65 to 74**	7,041	22.4	110.0	7,957	25.3	124.2
	**75 plus**	6,353	28.1	51.1	7,051	31.2	56.7
**Race**	**White**	15,745	4.4	250.6	17,711	4.9	283.0
	**Black**	2,971	6.2	49.2	3,314	6.9	54.9
	**Other**	1,478	5.8	24.2	1,650	6.5	26.9
**Ethnicity**	**Hispanic**	2,410	5.8	44.9	2,722	6.5	51.3
	**Not Hispanic**	17,784	4.6	279.1	19,953	5.1	313.6
**Sex**	**Female**	5,513	2.4	85.9	6,118	2.6	95.9
	**Male**	14,681	7.4	238.1	16,557	8.3	269.0
**Total**		20,194	4.7	324.0	22,675	5.3	364.9

Source: Vital Statistics of the United States.

Rates for age groups are unadjusted and all other rates are age-adjusted.

Years of potential life lost is based on U.S. life Table 2006–2018 estimates of life expectancy at age of death.

**Table 256:** Bile Duct Cancer: Incidence rates by age, race, ethnicity, and sex, 2016

Demographic Characteristics		Number of cases	Incidence per 100,000
**Age (years)**	**0 to 11**	.	0.0
	**12 to 24**	.	.
	**25 to 44**	425	0.5
	**45 to 54**	936	2.2
	**55 to 64**	2,889	6.8
	**65 to 74**	4,056	13.3
	**75 plus**	4,932	22.5
**Race**	**White**	10,361	3.2
	**Black**	1,217	3.0
	**Other**	1,090	4.0
**Ethnicity**	**Hispanic**	1,542	4.1
	**Not Hispanic**	11,007	3.2
**Sex**	**Female**	5,878	2.8
	**Male**	6,840	3.9
**Total**		12,720	3.3

Source: Surveillance, Epidemiology, and End Results (SEER) Program.

Rates for age groups are unadjusted and all other rates are age-adjusted.

**Table 257:** Bile Duct Cancer: Hospital discharges with first-listed and all-listed diagnoses by age, race, ethnicity, and sex in the United States, 2018

Demographic Characteristics		First-Listed Diagnosis		All-Listed Diagnoses	
		Number in thousands	Rate per 100,000	Number in thousands	Rate per 100,000
**Age (years)**	**0 to 11**	.	.	0	0
	**12 to 24**	0	0	0	0
	**25 to 44**	0	0	2	2
	**45 to 54**	1	3	3	8
	**55 to 64**	3	7	8	19
	**65 to 74**	4	12	11	35
	**75 plus**	3	16	10	44
**Race**	**White**	9	3	27	8
	**Black**	1	3	3	8
	**Other**	1	5	4	14
**Ethnicity**	**Hispanic**	1	4	4	10
	**Not Hispanic**	10	3	30	8
**Sex**	**Female**	5	2	16	7
	**Male**	6	3	18	10
**Total**		11	3	34	8

Source: Healthcare Cost and Utilization Project National Inpatient Sample.

Rates for age groups are unadjusted and all other rates are age-adjusted.

**Table 258:** Bile Duct Cancer: Deaths with underlying or underlying/other cause and lifetime years of life lost by age, race, ethnicity, and sex in the United States, 2019

Demographic Characteristics		Underlying Cause			Underlying/Other Cause		
		Number of Deaths	Rate per 100,000	Years of potential life lost in thousands	Number of Deaths	Rate per 100,000	Years of potential life lost in thousands
**Age (years)**	**0 to 11**	.	.	.	.	.	.
	**12 to 24**	10	0.0	0.6	11	0.0	0.6
	**25 to 44**	247	0.3	10.5	267	0.3	11.4
	**45 to 54**	617	1.5	19.3	664	1.6	20.8
	**55 to 64**	1,963	4.6	45.1	2,142	5.0	49.1
	**65 to 74**	3,130	9.9	49.8	3,422	10.9	54.4
	**75 plus**	3,871	17.1	31.4	4,250	18.8	34.5
**Race**	**White**	8,175	2.4	129.5	8,942	2.6	141.2
	**Black**	946	2.2	15.8	1,045	2.4	17.5
	**Other**	717	2.9	11.4	769	3.1	12.0
**Ethnicity**	**Hispanic**	909	2.2	18.2	1,004	2.4	20.0
	**Not Hispanic**	8,929	2.4	138.5	9,752	2.6	150.9
**Sex**	**Female**	4,756	2.1	78.4	5,147	2.3	84.7
	**Male**	5,082	2.7	78.3	5,609	3.0	86.2
**Total**		9,838	2.4	156.7	10,756	2.6	170.8

Source: Vital Statistics of the United States.

Rates for age groups are unadjusted and all other rates are age-adjusted.

Years of potential life lost is based on U.S. life Table 2006–2018 estimates of life expectancy at age of death.

**Table 259:** Gallbladder Cancer: Incidence rates by age, race, ethnicity, and sex, 2016

Demographic Characteristics		Number of cases	Incidence per 100,000
**Age (years)**	**0 to 11**	.	.
	**12 to 24**	.	.
	**25 to 44**	.	0.1
	**45 to 54**	326	0.8
	**55 to 64**	604	1.4
	**65 to 74**	1,315	4.3
	**75 plus**	2,009	9.2
**Race**	**White**	2,886	0.9
	**Black**	748	1.9
	**Other**	433	1.6
**Ethnicity**	**Hispanic**	583	1.6
	**Not Hispanic**	3,551	1.1
**Sex**	**Female**	2,923	1.4
	**Male**	1,254	0.8
**Total**		4,173	1.1

Source: Surveillance, Epidemiology, and End Results (SEER) Program.

Rates for age groups are unadjusted and all other rates are age-adjusted.

**Table 260:** Gallbladder Cancer: Hospital discharges with first-listed and all-listed diagnoses by age, race, ethnicity, and sex in the United States, 2018

Demographic Characteristics		First-Listed Diagnosis		All-Listed Diagnoses	
		Number in thousands	Rate per 100,000	Number in thousands	Rate per 100,000
**Age (years)**	**0 to 11**	.	.	0	0
	**12 to 24**	.	.	.	.
	**25 to 44**	0	0	0	0
	**45 to 54**	0	1	1	2
	**55 to 64**	1	2	2	4
	**65 to 74**	1	3	2	8
	**75 plus**	1	4	3	11
**Race**	**White**	2	1	5	2
	**Black**	1	1	1	3
	**Other**	0	1	1	4
**Ethnicity**	**Hispanic**	0	1	1	3
	**Not Hispanic**	2	1	7	2
**Sex**	**Female**	2	1	5	2
	**Male**	1	0	2	1
**Total**		3	1	8	2

Source: Healthcare Cost and Utilization Project National Inpatient Sample.

Rates for age groups are unadjusted and all other rates are age-adjusted.

**Table 261:** Gallbladder Cancer: Deaths with underlying or underlying/other cause and lifetime years of life lost by age, race, ethnicity, and sex in the United States, 2019

Demographic Characteristics		Underlying Cause			Underlying/Other Cause		
		Number of Deaths	Rate per 100,000	Years of potential life lost in thousands	Number of Deaths	Rate per 100,000	Years of potential life lost in thousands
**Age (years)**	**0 to 11**	.	.	.	.	.	.
	**12 to 24**	.	.	.	.	.	.
	**25 to 44**	18	0.0	0.8	21	0.0	0.9
	**45 to 54**	138	0.3	4.4	147	0.4	4.7
	**55 to 64**	404	1.0	9.5	429	1.0	10.1
	**65 to 74**	628	2.0	10.2	671	2.1	10.9
	**75 plus**	952	4.2	7.7	1,036	4.6	8.3
**Race**	**White**	1,614	0.5	24.4	1,732	0.5	26.1
	**Black**	374	0.9	5.9	404	0.9	6.3
	**Other**	152	0.6	2.3	168	0.7	2.6
**Ethnicity**	**Hispanic**	289	0.7	5.8	312	0.8	6.4
	**Not Hispanic**	1,851	0.5	26.8	1,992	0.5	28.6
**Sex**	**Female**	1,436	0.6	22.9	1,537	0.7	24.4
	**Male**	704	0.4	9.8	767	0.4	10.6
**Total**		2,140	0.5	32.6	2,304	0.6	34.9

Source: Vital Statistics of the United States.

Rates for age groups are unadjusted and all other rates are age-adjusted.

Years of potential life lost is based on U.S. life Table 2006–2018 estimates of life expectancy at age of death.

**Table 262:** Pancreatic Cancer: Incidence rates by age, race, ethnicity, and sex, 2016

Demographic Characteristics		Number of cases	Incidence per 100,000
**Age (years)**	**0 to 11**	.	0.0
	**12 to 24**	.	0.1
	**25 to 44**	1,194	1.4
	**45 to 54**	3,851	9.3
	**55 to 64**	11,352	26.9
	**65 to 74**	17,152	56.3
	**75 plus**	20,125	91.7
**Race**	**White**	43,366	13.3
	**Black**	6,430	15.6
	**Other**	3,001	11.1
**Ethnicity**	**Hispanic**	4,925	12.8
	**Not Hispanic**	45,662	13.3
**Sex**	**Female**	24,496	11.5
	**Male**	27,063	15.4
**Total**		51,567	13.3

Source: Surveillance, Epidemiology, and End Results (SEER) Program.

Rates for age groups are unadjusted and all other rates are age-adjusted.

**Table 263:** Pancreatic Cancer: Hospital discharges with first-listed and all-listed diagnoses by age, race, ethnicity, and sex in the United States, 2018

Demographic Characteristics		First-Listed Diagnosis		All-Listed Diagnoses	
		Number in thousands	Rate per 100,000	Number in thousands	Rate per 100,000
**Age (years)**	**0 to 11**	0	0	0	0
	**12 to 24**	0	0	0	0
	**25 to 44**	1	1	3	3
	**45 to 54**	3	8	10	23
	**55 to 64**	9	22	27	65
	**65 to 74**	12	39	37	122
	**75 plus**	12	53	33	152
**Race**	**White**	30	9	89	26
	**Black**	5	12	16	35
	**Other**	3	12	7	30
**Ethnicity**	**Hispanic**	3	8	10	24
	**Not Hispanic**	34	9	102	28
**Sex**	**Female**	19	9	54	24
	**Male**	18	10	57	30
**Total**		37	9	110	27

Source: Healthcare Cost and Utilization Project National Inpatient Sample.

Rates for age groups are unadjusted and all other rates are age-adjusted.

**Table 264:** Pancreatic Cancer: Deaths with underlying or underlying/other cause and lifetime years of life lost by age, race, ethnicity, and sex in the United States, 2019

Demographic Characteristics		Underlying Cause			Underlying/Other Cause		
		Number of Deaths	Rate per 100,000	Years of potential life lost in thousands	Number of Deaths	Rate per 100,000	Years of potential life lost in thousands
**Age (years)**	**0 to 11**	1	0.0	0.1	1	0.0	0.1
	**12 to 24**	7	0.0	0.4	8	0.0	0.5
	**25 to 44**	534	0.6	21.5	565	0.6	22.7
	**45 to 54**	2,454	6.0	74.7	2,551	6.2	77.7
	**55 to 64**	9,068	21.4	205.6	9,456	22.3	214.4
	**65 to 74**	14,448	45.9	227.7	15,173	48.2	238.9
	**75 plus**	19,444	86.1	155.9	20,570	91.1	164.7
**Race**	**White**	38,345	11.0	566.0	40,315	11.6	593.2
	**Black**	5,810	13.3	92.0	6,123	14.1	96.7
	**Other**	1,801	7.3	27.8	1,886	7.6	29.0
**Ethnicity**	**Hispanic**	2,941	7.4	53.4	3,087	7.8	56.0
	**Not Hispanic**	43,015	11.4	632.4	45,237	12.0	662.8
**Sex**	**Female**	22,176	9.6	333.4	23,224	10.1	348.3
	**Male**	23,780	12.7	352.4	25,100	13.4	370.5
**Total**		45,956	11.0	685.8	48,324	11.6	718.8

Source: Vital Statistics of the United States.

Rates for age groups are unadjusted and all other rates are age-adjusted.

Years of potential life lost is based on U.S. life Table 2006–2018 estimates of life expectancy at age of death.
